# Enhancement of efferocytosis through biased FPR2 signaling attenuates intestinal inflammation

**DOI:** 10.15252/emmm.202317815

**Published:** 2023-11-22

**Authors:** Ming‐Yue Wu, Yun‐Jun Ge, Er‐Jin Wang, Qi‐Wen Liao, Zheng‐Yu Ren, Yang Yu, Guoyuan Zhu, Chun‐Ping Liu, Meng‐Ni Zhang, Huanxing Su, Han‐Ming Shen, Ye Chen, Lei Wang, Yi‐Tao Wang, Min Li, Zhaoxiang Bian, Jin Chai, Richard D Ye, Jia‐Hong Lu

**Affiliations:** ^1^ State Key Laboratory of Quality Research in Chinese Medicine, Institute of Chinese Medical Sciences University of Macau Macau SAR China; ^2^ Department of Gastroenterology, The First Affiliated Hospital (Southwest Hospital) Third Military Medical University (Army Medical University) Chongqing China; ^3^ Kobilka Institute of Innovative Drug Discovery, School of Medicine The Chinese University of Hong Kong Shenzhen China; ^4^ Department of Basic Medical Science, Wuxi School of Medicine Jiangnan University Wuxi China; ^5^ Engineering Research Center of Cell and Therapeutic Antibody Medicine, Ministry of Education, School of Pharmacy Shanghai Jiao Tong University Shanghai China; ^6^ State Key Laboratory of Quality Research in Chinese Medicine, Macau Institute for Applied Research in Medicine and Health Macau University of Science and Technology Macau SAR China; ^7^ Department of Cardiovascular Medicine The Second Affiliated Hospital of Guangzhou University of Chinese Medicine Guangzhou China; ^8^ Guangdong‐Hong Kong‐Macau Joint Lab on Chinese Medicine and Immune Disease Research University of Macau Macau SAR China; ^9^ Faculty of Health Sciences University of Macau Macau SAR China; ^10^ Integrative Microecology Center, Department of Gastroenterology, Shenzhen Hospital Southern Medical University Shenzhen, Guangzhou China; ^11^ School of Chinese Medicine Hong Kong Baptist University Hongkong SAR China; ^12^ The Second Affiliated Hospital, School of Medicine The Chinese University of Hong Kong Shenzhen China

**Keywords:** columbamine, FPR2, inflammatory bowel disease, LC3‐associated efferocytosis, Autophagy & Cell Death, Digestive System, Immunology

## Abstract

Efficient clearance of dying cells (efferocytosis) is an evolutionarily conserved process for tissue homeostasis. Genetic enhancement of efferocytosis exhibits therapeutic potential for inflammation resolution and tissue repair. However, pharmacological approaches to enhance efferocytosis remain sparse due to a lack of targets for modulation. Here, we report the identification of columbamine (COL) which enhances macrophage‐mediated efferocytosis and attenuates intestinal inflammation in a murine colitis model. COL enhances efferocytosis by promoting LC3‐associated phagocytosis (LAP), a non‐canonical form of autophagy. Transcriptome analysis and pharmacological characterization revealed that COL is a biased agonist that occupies a part of the ligand binding pocket of formyl peptide receptor 2 (FPR2), a G‐protein coupled receptor involved in inflammation regulation. Genetic ablation of the *Fpr2* gene or treatment with an FPR2 antagonist abolishes COL‐induced efferocytosis, anti‐colitis activity and LAP. Taken together, our study identifies FPR2 as a potential target for modulating LC3‐associated efferocytosis to alleviate intestinal inflammation and highlights the therapeutic value of COL, a natural and biased agonist of FPR2, in the treatment of inflammatory bowel disease.

## Graphical abstract

Enhancement of efferocytosis has been regarded as an emerging strategy for inflammatory diseases, while pharmacological approaches to modulate efferocytosis are poorly defined. Our study identified a natural compound, columbamine (COL), that can activate LC3‐associated efferocytosis and attenuate DSS‐induced colitis by biasedly targeting FPR2 on macrophages. This study provides a novel therapeutic strategy for inflammatory diseases, including colitis, via enhancing FPR2‐mediated efferocytosis.

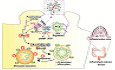

**The paper explained**

**Problem**

Efferocytosis (apoptotic cells clearance) enhancement has been regarded as a strategy to resolve inflammation. However, pharmacological approaches to modulate efferocytosis are poorly developed due to the limited drug targets being identified.

**Results**

We established a screening model and identified a natural compound, columbamine (COL), that can enhance LC3‐associated efferocytosis and resolve intestinal inflammation in mice. Mechanistic analysis demonstrates that COL directly bind to and biasedly activates formyl peptide receptor 2 (FPR2) to resolve intestinal inflammation and promote efferocytosis.

**Impact**

This study identifies FPR2 as a potential target for pharmacological modulation of efferocytosis to alleviate inflammation and highlights the therapeutic value of COL, a natural efferocytosis enhancer, in the treatment of inflammatory diseases including inflammatory bowel disease.

## Introduction

Approximately 200–300 billion dead cells are produced daily in an adult (Mehrotra & Ravichandran, 2022). Due to the efficient dead cell clearance process termed efferocytosis, the dead cells are rapidly removed and rarely visible in tissues. Dead cells release damage‐associated molecular patterns (DAMPs) including DNA and ATP that trigger innate immune response if not cleared efficiently; hence, efferocytosis is an intrinsical anti‐inflammatory mechanism. Recent studies have revealed that efferocytosis actively inhibits inflammation by inducing metabolic reprogramming of phagocytic cells (Medina *et al*, 2020; Yurdagul *et al*, 2020). Impairment of efferocytosis has been implicated in a wide range of diseases including autoimmune diseases, atherosclerosis, cancer, and infections. Regulation of efferocytosis may offer new opportunities for therapeutic intervention of these diseases.

Inflammatory bowel disease (IBD) is a chronic relapsing and remitting intestinal inflammatory disease that can be categorized into two forms, Crohn's disease (CD) and ulcerative colitis (UC) (Mulder *et al*, 2014). Previous studies suggest that efferocytosis mediates colonic inflammatory response and tissue repair. Deficiency in LSECtin (Clec4g)‐dependent corpse clearance by macrophages and lack of NRBF2‐mediated efferocytosis result in serious epithelium damage and inflammatory response in an experimental colitis model (Yang *et al*, 2018; Wu *et al*, 2020). Furthermore, genetic enhancement of efferocytosis protects against intestinal inflammation (Lee *et al*, 2016). These results indicate that efferocytosis is a potent mechanism for intestinal inflammation resolution.

Several molecules have been reported to enhance efferocytosis and mitigate inflammation. Lovastatin, glucocorticoids, N‐acetylcysteine, resolvin D1 and sulfated glycosphingolipid enhance efferocytosis and resolve inflammation with varying mechanisms (Morimoto *et al*, 2006; Popovic *et al*, 2007; Moon *et al*, 2010; Garabuczi *et al*, 2015; Luo *et al*, 2016), and most of them display protective effects in IBD models (Weylandt *et al*, 2007; Guijarro *et al*, 2008; Dubois‐Camacho *et al*, 2017). Because of the multiple mechanisms involved, the relative importance of efferocytosis is unclear.

Formyl peptide receptor 2 (FPR2), a promising anti‐inflammatory therapeutic target, is highly expressed in phagocytic cells as a member of the G protein‐coupled receptors (GPCRs) (Qin *et al*, 2022; Dahlgren *et al*, 2023). Different ligands bind to FPR2 and elicit distinct signal cascades for modulation of pro‐inflammatory or pro‐resolving functions (Vong *et al*, 2012; Balzola *et al*, 2013; Kim *et al*, 2013; Leoni *et al*, 2013). Interestingly, several endogenous efferocytosis enhancers such as Annexin A1 (ANXA1), N‐terminal peptide of Annexin A1 (Ac2‐26), Lipoxin A4 (LXA4), and Resolvin D family members (RvD1, RvD3, RvD5) are all reported to be FPR2 agonists (Arienti *et al*, 2019). These results suggest that FPR2 ligands may actively regulate intestinal inflammation through efferocytosis. However, a causal relationship between FPR2 signaling and efferocytosis has not been established.

Natural compounds are promising sources for lead compound identification. Several natural compounds, such as quercetin (Sotnikova *et al*, 2013), curcumin (Burge *et al*, 2019), and berberine (Habtemariam, 2016), have been consistently found effective for IBD treatment. However, the anti‐colitis mechanisms of these natural compounds are poorly understood. Here we report the identification of columbamine (COL), a natural alkaloid, as an LAP‐associated efferocytosis booster to lessen colonic inflammation by triggering biased signaling through FPR2. These findings unveil a novel role for FPR2 in mediating LC3‐associated efferocytosis to alleviate colonic inflammation and suggest the therapeutic potential of COL as a biased agonist of FPR2 for colitis treatment.

## Results

### Identification of COL as an efferocytosis enhancer

**Figure 1:**
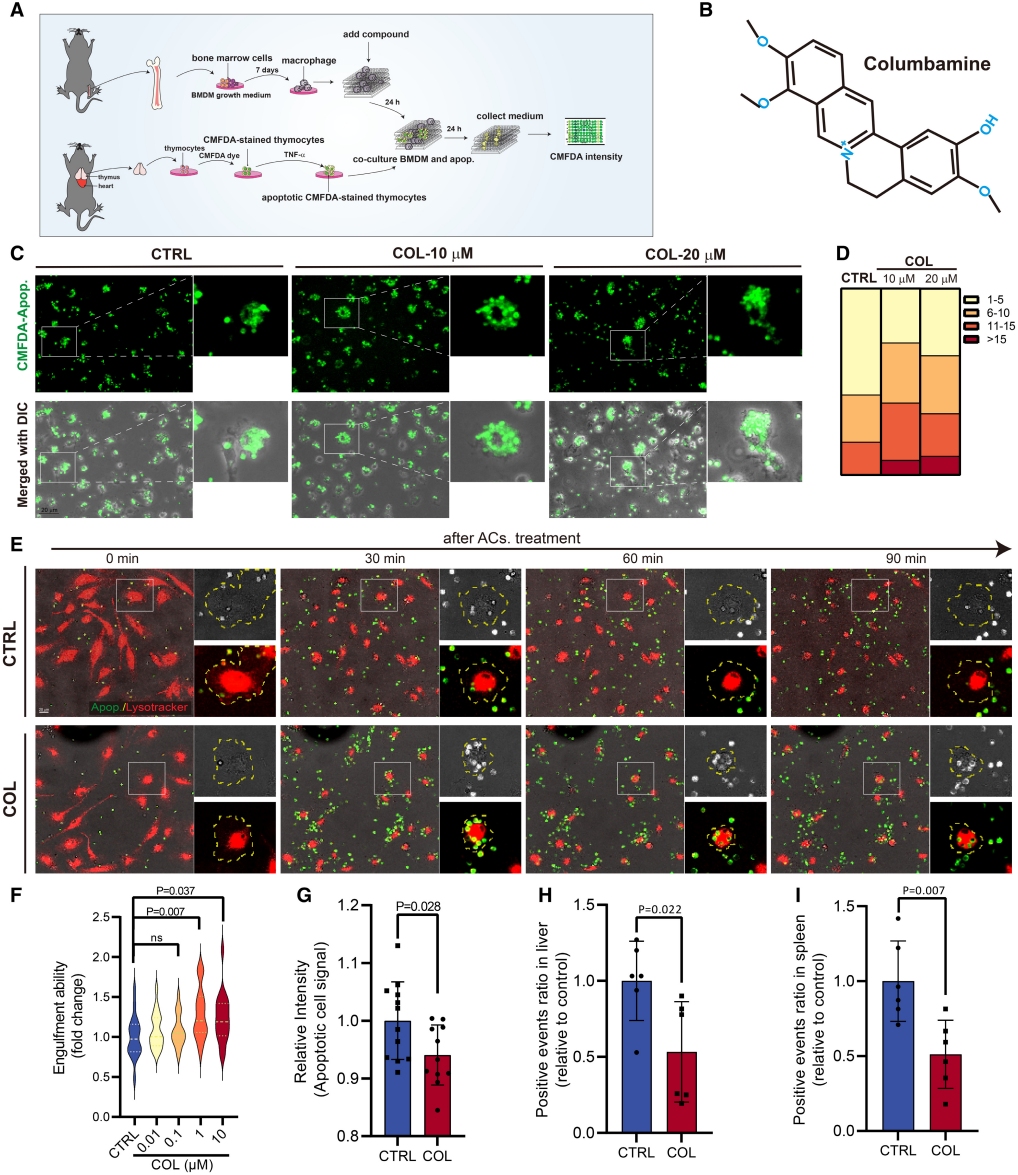
Identification of COL as an efferocytosis enhancer ASchematic overview of functional assay to monitor efferocytosis.BChemical structure of columbamine (COL).CRepresentative images of BMDMs after co‐culturing CMFDA‐stained ACs with BMDMs (with or without treatment of COL) for 24 h (Green: CMFDA‐labeled‐ACs). Scale bar, 20 μm.DQuantification of BMDMs based on the number of engulfed ACs (1–5 ACs, 6–10 ACs, 11–15 ACs, above 15 ACs).EBMDMs were pretreated with vehicle or COL (10 μM) for 24 h, and the representative time‐lapse images at indicated time points were captured after treating with ACs (Green: CMFDA‐labeled‐ACs, Red: LysoTracker). Scale bar, 20 μm.FQuantification of relative engulfment ability for BMDMs with or without pre‐treatment of COL (10 mg/kg body weight) (*n* = 3).GRelative CMFDA intensity after co‐culturing BMDMs (with or without COL pretreatment) with CMFDA‐labeled ACs (*n* = 11 or 12).H, IRelative positive CMFDA events in livers and spleens in mice with or without COL treatment after injecting CMFDA‐ACs (*n* = 6). Data information: Data are shown as mean ± SD. Multiple *t*‐test followed by Bonferroni correction was used in (F). Unpaired 2‐sided *t*‐test was used in (G–I). *P*‐values are indicated in the figures. Experimental numbers indicate biological replicates. Experiments from (C–I) replicated for at least three times. Source data are available online for this figure.

We established an *in vitro* assay for screening of efferocytosis enhancers by incubating TNF‐α‐induced apoptotic thymocytes (CMFDA staining) with primary mouse bone marrow‐derived macrophages (BMDMs) and analyzing the efferocytosis capacity by detecting unphagocytosed CMFDA‐derived fluorescence intensity in culture medium (Fig 1A). A library containing 392 natural compounds with diverse structures were screened, and several compounds, including columbamine (COL) (Fig 1B), were identified as potentially being able to accelerate efferocytosis (Appendix Fig S1A, Appendix Table S1). BMDMs treated with COL engulfed more apoptotic cells (ACs) than control group after 24 h of incubation (Fig 1C and D) without showing obvious toxicity (Appendix Fig S1B), indicating an enhanced phagocytosis capacity. Furthermore, time‐lapse imaging of COL‐treated BMDMs co‐cultured with ACs showed quick recognition of ACs and more ACs were engulfed in a 2‐h period (Fig 1E) (Movies EV1 and EV2). The engulfed ACs were subsequently acidified in lysosomes, as we obtained more mature AC‐containing phagosomes in COL‐treated BMDMs than control group (Appendix Fig S1C and D). We also found that BMDMs started to display enhanced efferocytosis at the COL concentration of 10 nM, and to significantly boost efferocytosis *in vitro* at 1 μM of COL (Fig 1F and G). *In vivo* study showed that COL administration in mice accelerated clearance of ACs in liver and spleen (Fig 1H and I, Appendix Fig S1E–I). Collectively, COL was identified as an efferocytosis enhancer that stimulates the capacity of BMDMs to ingest and degrade ACs *in vitro* and *in vivo*.

### COL activates efferocytosis by boosting LC3‐associated phagocytosis

**Figure 2:**
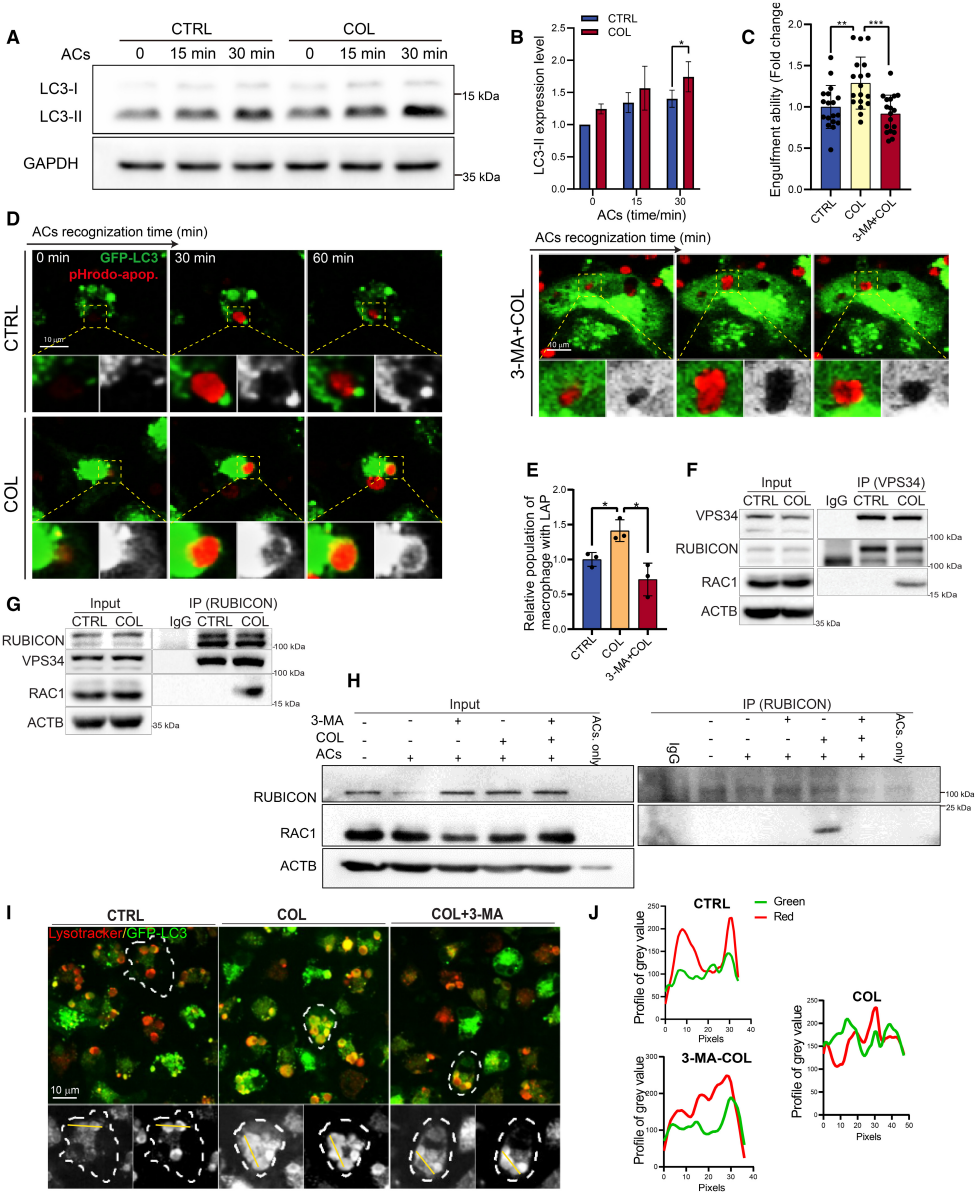
COL induces LC3‐associated phagocytosis ARepresentative WB images of LC3 in BMDMs after AC treatment for the indicated times.BQuantification of LC3‐II expression (*n* = 3 biological replicates).CComparison of engulfment ability of macrophages after pre‐treating with 3‐MA (5 mM) or not. In the COL‐treated group, COL (1 μM) was added for 2 h followed by addition of ACs (*n* = 18).DRepresentative images of LC3‐associated phagocytosis after pHrodo‐SE labeled‐ACs incubated with GFP‐LC3 BMDMs at the indicated time points. The 3‐MA (5 mM) were used for pre‐treatment for 30 min, and COL (1 μM) was added for 2 h followed by addition of ACs. Green: GFP‐LC3; Red: pHrodo‐SE. Scale bar, 10 μm.EQuantification of LAP efficiency in GFP‐LC3‐BMDMs after different treatment (*n* = 3).F, GCell lysates of BMDMs (pre‐treated with 1 μM COL or not) treated with ACs were subjected to co‐IP by a Vps34 or Rubicon antibody and followed by WB analysis using the indicated antibodies.HCell lysates of ACs and BMDMs that treated with or without ACs were subjected to co‐IP by a Rubicon antibody and followed by WB analysis using the indicated antibodies. The BMDM were pre‐treated with vehicle, COL (1 μM), or 3‐MA (5 mM).IRepresentative fluorescent images of GFP‐LC3 BMDMs treated with ACs. The BMDMs were pre‐stained with LysoTracker Red for 30 min, then treated with vehicle or 3‐MA (5 mM) for 30 min. The COL (1 μM) was added for another 2 h.JLine profiles of intensity of GFP and LysoTracker Red fluorescent value along the yellow line in (I). Data information: Data are shown as mean ± SD. *P < 0.05, **P < 0.01, ***P < 0.01. Multiple *t*‐test are followed by Bonferroni correction unpaired *t*‐test. Experimental numbers indicate biological replicates. Experiments have been replicated for at least three times. Source data are available online for this figure.

LC3‐asscoiated phagocytosis (LAP) is the phagocytic process characterized by LC3 accumulation around phagosomes, which induces phagosome maturation and enhances efferocytosis (Martinez *et al*, 2011). AC incubation with BMDMs induced a fast increase of LC3 lipidation, a marker for LC3 membrane recruitment. Our results showed that COL treatment enhanced AC‐induced LC3 lipidation (Fig 2A and B). FYVE is a PI3P effector protein domain that has been used to determine PI3KC3 activity (Gillooly *et al*, 2000). We transfected RAW 264.7 cells with GFP‐2xFYVE plasmid and detected FYVE puncta formation during phagocytosis. COL‐treated macrophage engulfed microspheres quickly (within 30 min) and also colocalized well with GFP‐FYVE compared to the non‐treated group (Appendix Fig S2). Blocking LAP by 3‐methyladenine (3‐MA) (Pick, 2014) inhibited the COL‐induced ACs engulfment promotion in macrophage (Fig 2C). To directly observe the LAP alteration, we isolated BMDMs from GFP‐LC3 transgenic mice and incubated them with ACs. LC3‐associated efferocytosis was captured by time‐lapse imaging. We observed that COL treatment quickly and strongly enhanced the GFP‐LC3 recruitment to engulfed ACs in BMDMs, while 3‐MA treatment totally blocked the recruitment (Fig 2D and E) (Movies [Link], [Link]). VPS34‐RUBICON formation is necessary for LAP initiation (Pick, 2014). RAC1, a small GTPase that mediate membrane ruffle dynamics, is also involved in LAP by acting with the RUBICON‐NOX2 complex (Martinez *et al*, 2015). To determine whether COL‐induced LAP is associated with the VPS34‐RUBICON complex, we used co‐IP to detect VPS34 and RUBICON‐binding proteins during efferocytosis. Interestingly, we found that COL triggered VPS34‐RUBICON‐RAC1 formation (Fig 2F and G), which can be totally blocked by addition of 3‐MA (Fig 2H). To monitor the LAP maturation process, we pre‐stained GFP‐LC3 BMDMs with lysotracker. The data showed that, once LAP formed, phagosomes containing ACs became red quickly, indicating an efficient maturation of AC containing phagosomes after COL treatment. Meanwhile, COL induced increased GFP‐LC3 recruitment to phagosomes, which can be blocked by 3‐MA (Fig 2I and J). These results infer that COL induced LAP‐associated efferocytosis by enhancing RUBICON‐VPS34‐RAC1 interactions.

### COL administration attenuates DSS‐induced colitis in mice in a macrophage‐dependent manner

**Figure 3:**
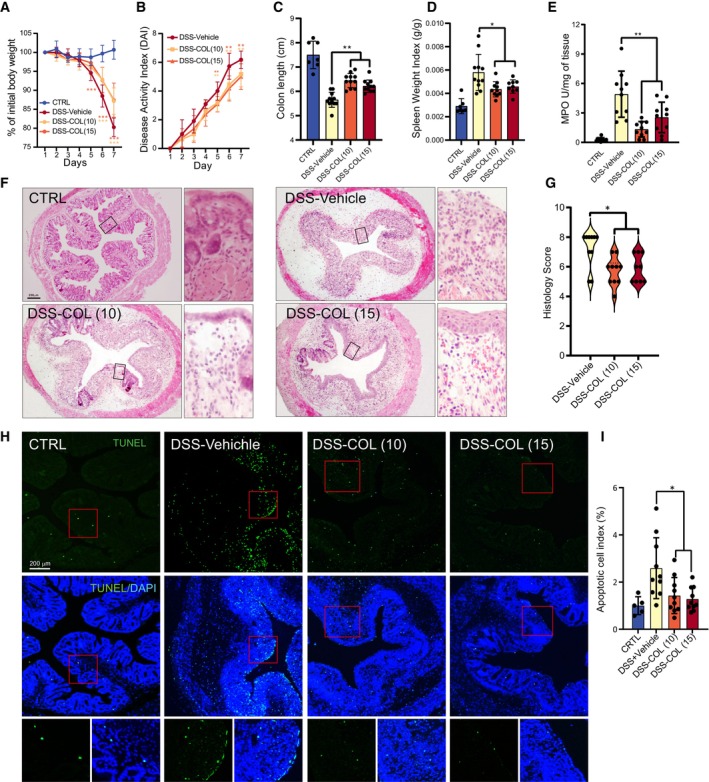
COL administration attenuates DSS‐induced colitis in mice A, BMice were treated with vehicle, 10 mg/kg COL (COL (10)), or 15 mg/kg COL (COL (15)), and quantification of daily body weight and DAI alteration during DSS treatment were recorded (*n* = 7–11).C, DQuantification of colon length and spleen weight after indicated treatments (*n* = 7–11).EMPO activity determination in different groups (*n* = 7–10).FRepresentative images of HE staining. Scale bar, 200 μm.GComparison of histology score based on the observations of HE staining images (*n* = 9–10).HRepresentative images of TUNEL staining of colon tissue in different groups. Green: TUNEL positive area; Blue: DAPI staining. Scale bar, 200 μm.IApoptotic cell index analysis (*n* = 5 in CTRL, *n* = 10 in other groups). Data information: Data are shown as mean ± SEM in (A, B), and as mean ± SD in (C–E) and (I). Multiple *t*‐test was followed by Bonferroni correction. *P < 0.05, **P < 0.01, ***P < 0.001. Experimental numbers indicate biological replicates. The experiments have been replicated for at least three times. Source data are available online for this figure.

Efferocytosis enhancement has been shown to alleviate inflammation in an inflammatory bowel disease (IBD) model (Lee *et al*, 2016). To investigate whether the efferocytosis enhancer COL exerts anti‐inflammation properties in an IBD model, we injected COL daily into mice with DSS‐induced colitis. The results showed that COL administration alleviated DSS‐induced body weight reduction, colon length shortening, as well as increase in Disease Activity Index (DAI), spleen weight and MPO activity (Fig 3A–E). Histology examination showed an overall improved morphology of colon tissue in COL‐treated groups (Fig 3F and G). TUNEL staining of the colon tissue sections from COL‐treated mice showed a dramatic reduction in the number of ACs (Fig 3H and I). These results indicate that COL can attenuate DSS‐induced colitis and AC accumulation in mice.

To determine whether LAP was involved in the COL‐induced colitis amelioration, we used 3‐MA to block LAP in a colitis mouse model (Macias‐Ceja *et al*, 2017). As expected, 3‐MA treatment largely blocked the COL induced‐colonic efferocytosis enhancement and anti‐colitis effects (Appendix Fig S3A–H). Collectively, LAP mediated anti‐colitis activity of COL.

**Figure 4:**
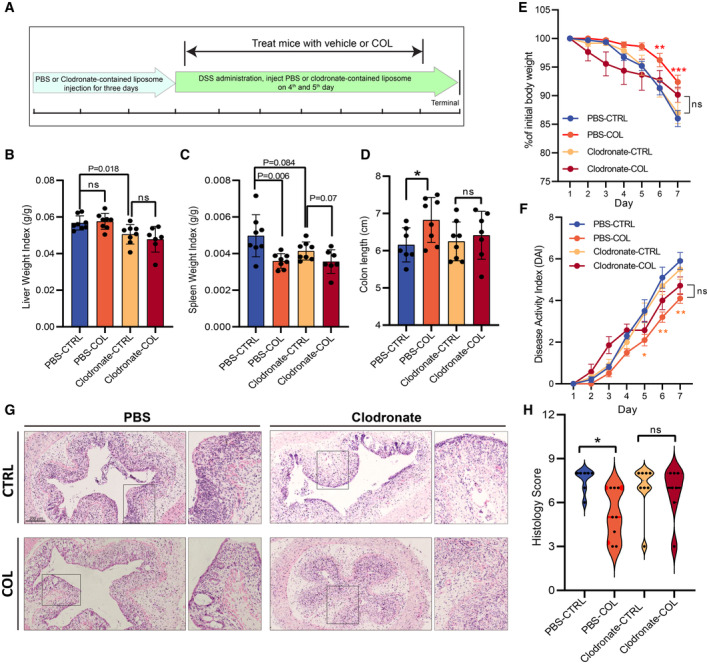
Macrophage depletion inhibited the protective effects of COL on DSS‐induced mouse colitis ASchematic overview of macrophages depletion *in vivo* via clodronate‐liposome injection (*n* = 7–8). Mice were injected with PBS‐ or clodronate‐liposome for 3 days before DSS administration. After DSS treatment, liposomes were injected at the 4^th^ and 5^th^ days, and COL or vehicle were injected daily via i.p.B, CComparison of liver and spleen weight index (*n* = 7–8).D, EComparison of colon length and body weight, respectively, during DSS treatment (*n* = 7–8).FComparison of DAI alteration after different treatment (*n* = 7–8).G, HHistological studies of colon tissue. Data information: (G) Representative images after HE staining; Scale bar, 200 μm. (H) Comparison of histology score based on the observations of HE staining images (*n* = 7–8). Data are shown as mean ± SD in (B–D), and mean ± SEM in (E) and (F). Multiple *t*‐test followed by Bonferroni correction. *P < 0.05, **P < 0.01, ***P < 0.001 or *P*‐values are indicated in the figures; ns, not significant. Experimental numbers indicate biological replicates. Source data are available online for this figure.

Intestines contain the largest number of macrophages, the professional phagocytes that primarily execute efferocytosis, in the body (Heinsbroek & Gordon, 2009). To assess whether macrophages are required for the anti‐colitis effect of COL, we depleted macrophages by clodronate‐contained liposome (Clo‐Lipo DOTAP) (Fig 4A). The efficacy of Clo‐Lipo DOTAP on macrophage depletion had been confirmed before usage in a mouse colitis model (Appendix Fig S4B). After DSS‐treatment, mice with macrophage deletion displayed lower spleen and liver weight compared to controls (Fig 4B and C), which was consistent with previous studies (Bu *et al*, 2013; Bader *et al*, 2018). Colonic CD68 expression analysis showed that Clo‐Lipo DOTAP injection depleted about 50% colonic macrophages (Appendix Fig S4C and D). Our results showed that the anti‐colitis activity of COL was largely abolished (Fig 4D–F). In addition, mice with macrophage depletion did not show attenuated effects in colonic epithelium disruption and inflammatory cell infiltration after COL treatment (Fig 4G and H). Colonic *in situ* efferocytosis efficiency is higher in COL‐treated mice, but the difference was eliminated after depletion of macrophage (Appendix Fig S4A). These results suggested that macrophage is a target cell type of COL in alleviating colitis injury.

### Formyl peptide receptor 2 (FPR2) is involved in intestinal inflammation regulation and required for COL‐induced efferocytosis

**Figure 5:**
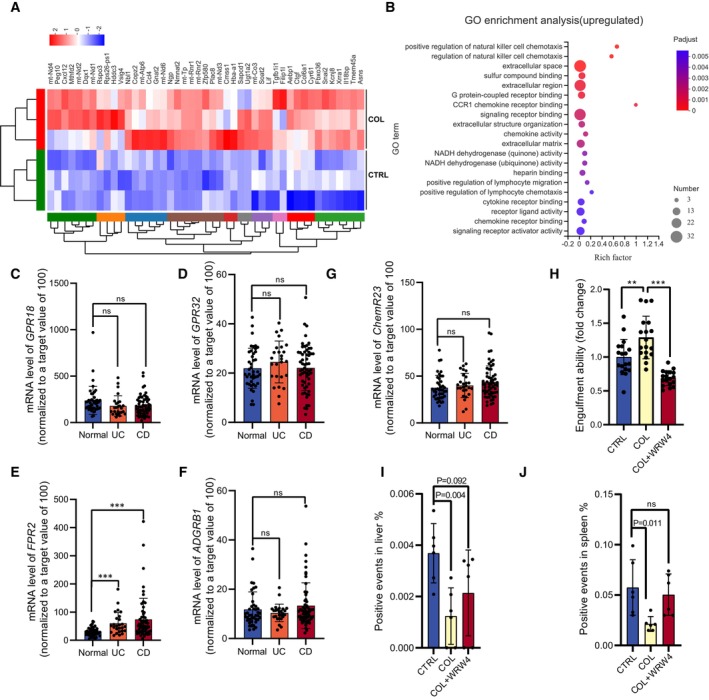
FPR2 is associated with IBD and required for COL‐induced efferocytosis A, BTranscriptomic analysis of macrophages with or without COL treatment. (A) Heat map analysis of the up‐regulated genes in COL‐treated macrophages. (B) Gene Ontology (GO) enrichment analysis of COL‐induced upregulated genes in macrophages.C–GComparison of selected mRNA expression levels in IBD patients' peripheral blood mononuclear cells from the GEO profiles of GDS1615/210773, GDS1615/210279, GDS1615/221469, GDS1615/206083, and GDS1615/210659 (*n* = 26–59).HComparison of efferocytosis ability in macrophages with or without FPR2 antagonist (WRW4) treatment (*n* = 6, three images were selected in each sample). WRW4 (30 μM) was given for 30 min, followed by COL (1 μM) treatment for another 2 h. Three replicated experiments.I, JPositive CMFDA events in livers (I) and spleens (J) of mice with or without COL treatment after injecting CMFDA‐ACs (*n* = 6). WRW4 was injected 30 min prior to COL administration and 6 h after ACs injection. Data information: Multiple *t*‐test was followed by Bonferroni correction. ***P < 0.001 or indicated in the figures; ns, not significant. Data are shown as mean ± SD. Experimental numbers indicate biological replicates. Source data are available online for this figure.

To reveal how COL worked on macrophages, we analyzed the transcriptome data from COL‐treated macrophages (Fig 5A). GO enrichment analysis indicates that COL increased the expressions of genes associated with chemotaxis, receptor binding, extracellular organization, NADH activity, and G protein‐coupled receptor (GPCR) bindings (Fig 5B). GPCRs are the most intensively studied drug targets, because they are involved in diverse physiological functions and are accessible on the cell surface (Hauser *et al*, 2017). We hypothesize that GPCR is a potential target of COL. We compared mRNA levels of phagocytosis‐related GPCRs (Ravichandran, 2010; Arienti *et al*, 2019) in peripheral blood mononuclear cells from UC or CD patients according to their gene expression omnibus (GEO) profiles (Burczynski *et al*, 2006). We found that *FPR2* is the only differentially expressed gene in peripheral blood mononuclear cells from IBD patients (Fig 5C–G), indicating a high correlation between FPR2 and IBD. To confirm whether FPR2 is required for COL‐induced biological functions, we blocked FPR2 signal transduction by pre‐treatment with WRW4 peptide, a specific FPR2 antagonist, before adding COL. We found that blocking FPR2 prevented COL‐induced efferocytosis enhancement both *in vitro* (Fig 5H) and *in vivo* (Fig 5I and J). These results verified our hypothesis that FPR2 is necessary for COL‐induced efferocytosis.

### FPR2 is required for the anti‐colitis activity of COL

To evaluate whether FPR2 is the target of COL for its anti‐colitis effects, we further administered WRW4 to colitis mice. The results showed that blocking FPR2 prevented COL‐induced amelioration of colitis. Whereas the mice treated only with COL exhibited mild body weight reduction and raising level of DAI, mice with WRW4 treatment before COL administration did not show improvement in body weight reduction and DAI (Appendix Fig S4E and F). In addition, WRW4 abrogated the efficacy of COL in maintaining colon length and decreasing MPO activity (Appendix Fig S4G and H). Colon histology analysis also revealed dampen epithelial structure and inflammatory cell infiltration after treatment with WRW4 compared to the COL only group of mice (Appendix Fig S4I and J).

**Figure 6:**
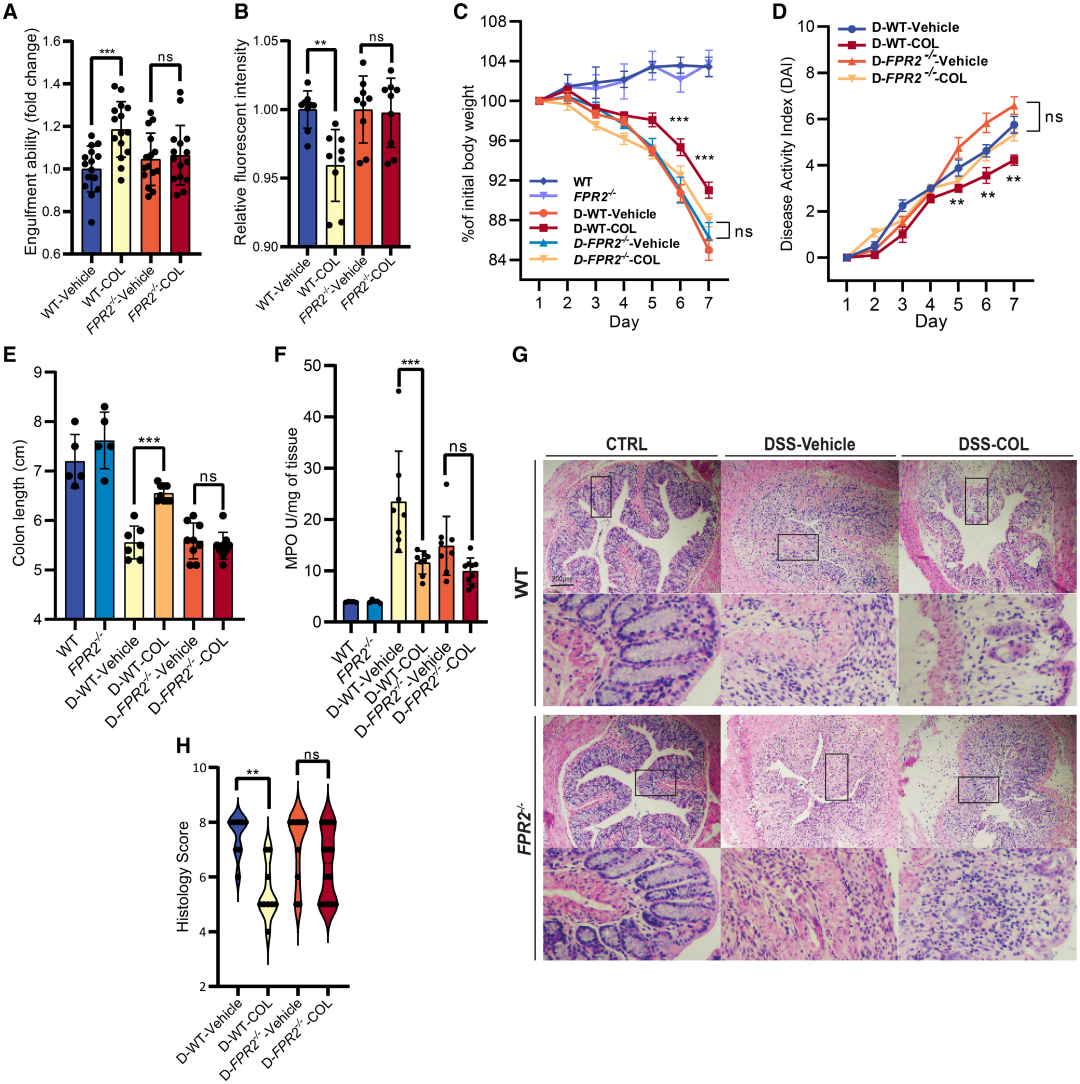
Loss of FPR2 abolished COL‐induced colitis attenuation and efferocytosis enhancement AComparison of engulfment ability of macrophages in different groups (*n* = 15).BRelative CMFDA intensity after co‐culturing BMDMs with CMFDA‐labeled ACs. BMDMs were pretreated with 1 μM COL for 2 h (*n* = 8–9).C, DComparison of daily body weight and DAI alteration, respectively, during DSS treatment in different groups (*n* = 5 in control groups, *n* = 7–9 in model groups).EComparison of colon length after different treatment (*n* = 5 in control groups, *n* = 7–9 in model groups).FComparison of myeloperoxidase (MPO) activity in tissues among groups (*n* = 5 in control groups, *n* = 7–9 in model groups).GRepresentative images after HE staining; Scale bar, 200 μm.HComparison of histology score based on the HE staining images (*n* = 8–9). Data information: Multiple *t*‐test was followed by Bonferroni correction. **P < 0.01, ***P < 0.001; ns, not significant. Data are shown as mean ± SEM in (C, D) and mean ± SD in (A, B, E, F, H). Experimental numbers indicate biological replicates. Source data are available online for this figure.

To confirm that FPR2 mediates the effect of COL, we used the *FPR2* knockout (KO) mice (Appendix Fig S5A). Consistent with the data obtained with WRW4 treatment, COL‐induced efferocytosis was totally abolished in *FPR2* knockout BMDMs (Fig 6A and B). In the colitis model, *FPR2* ^−/−^ mice did not exhibit obvious body weight reduction, but showed a higher DAI compared to the wild type (WT) mice after DSS treatment (Fig 6C and D), indicating that FPR2 partially regulated the DSS‐induced colitis development, which is consistent with a previous report (Balzola *et al*, 2013). As expected, deletion of *FPR2* abolished the anti‐colitis activity of COL in ameliorating body weight reduction, DAI increase, and colon shrinkage (Fig 6C–E). However, MPO activity increase was inhibited in inflammatory colonic tissue from *FPR2* ^−/−^ mice (Fig 6F), probably because of the involvement of FPR2 in chemotaxis and neutrophil degranulation (Sun & Ye, 2012). In addition, histological analysis showed that severe DSS‐induced damage of epithelial layers, and inflammatory cell infiltration could not be alleviated by COL in *FPR2* ^−/−^ mice (Fig 6G and H). Colonic *in situ* efferocytosis efficiency is higher in COL‐treated mice, but the difference was eliminated in FPR2 deficiency mice (Appendix Fig S5B). Collectively, these data indicate that FPR2 is required for COL‐induced attenuation of mouse colitis.

### COL induces biased signaling through FPR2 to promote efferocytosis

**Figure 7:**
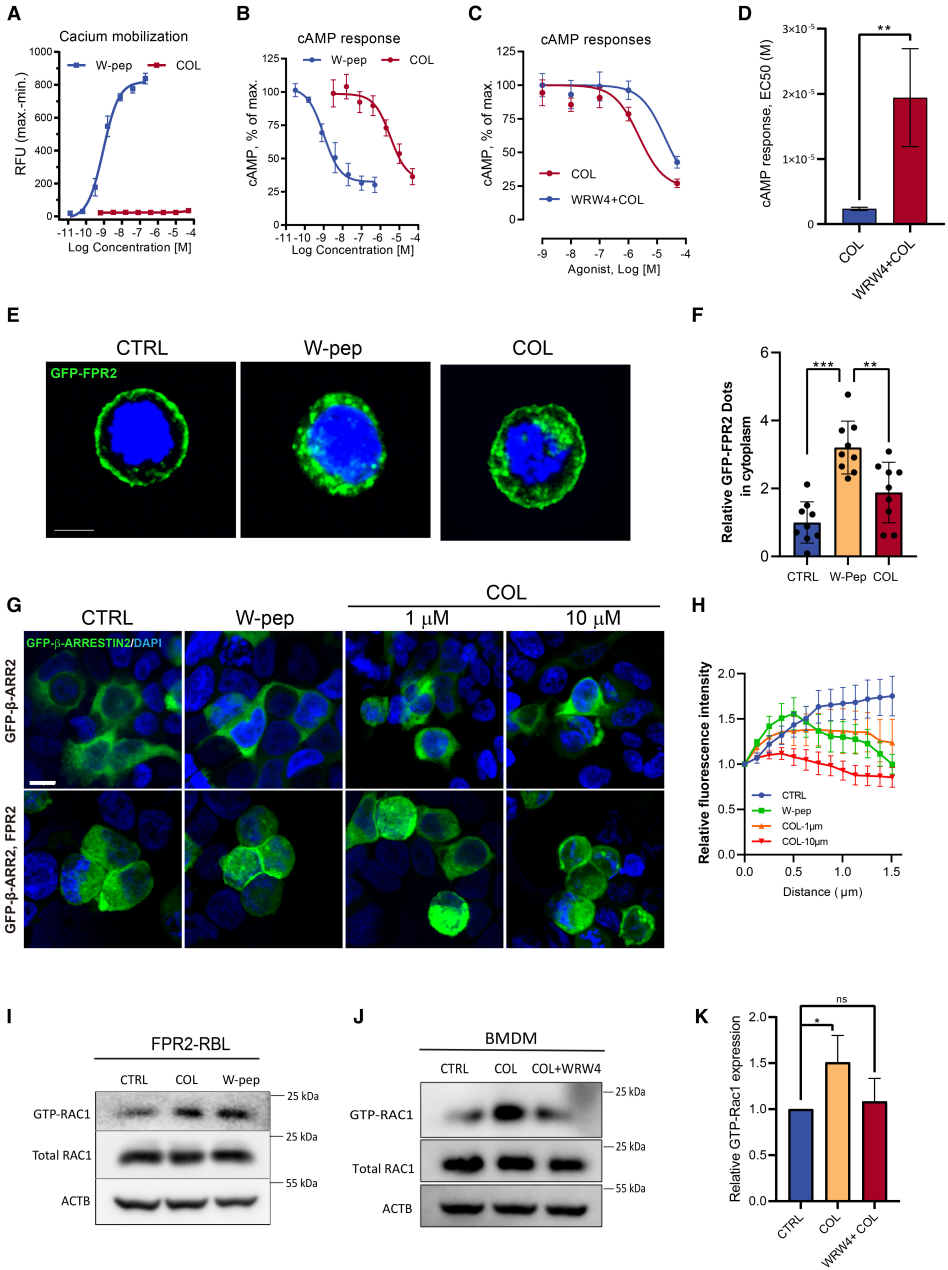
Determination of COL‐induced FPR2 signaling The dose‐response curves of COL and WKYMVm (W‐pep)‐induced calcium mobilization in FPR2‐RBL cells (*n* = 3).The dose‐response curves of COL and W‐pep‐induced inhibition of forskolin‐stimulated cAMP accumulation in FPR2‐expressing cells (*n* = 3).The dose‐response curves of COL and COL plus WRW4‐induced inhibition of forskolin‐stimulated cAMP accumulation in FPR2‐expressing cells (*n* = 3). WRW4 (30 μM) was added 30 min prior to COL treatment.Effect of COL for inhibition of cAMP responses with or without WRW4 treatment (*n* = 3). EC_50_ were calculated by Prism software.Representative GFP‐FPR2 images after treating GFP‐FPR2‐RBL cells with COL (10 μM) or W‐pep (0.1 μM) (*n* = 9). Scale bar, 5 μm.Quantification of GFP‐FPR2 dots that distributed in the cytoplasm.Representative images to show GFP‐β‐arrestin recruitment in HEK293 cells transfected with GFP‐β‐arrestin plasmid or GFP‐β‐arrestin combined with FPR2 plasmids after W‐pep (0.1 μM) or COL treatment for 30 min. Scale bar, 20 μm.Relative intensity of GFP‐β‐arrestin based on the plot profile (*n* = 6–8). Higher peak intensity enriched in a short distance indicates stronger membrane recruitment capacity.GTP‐RAC1 levels in FPR2‐RBL cells after COL (1 μM) or W‐pep (0.1 μM) treatment for 10 min.Levels of GTP‐RAC1 in BMDMs after COL (1 μM) treatment for 10 min with or without WRW4 pre‐treatment (30 μM, 30 min).Quantification of Western blot analysis of GTP‐RAC1 level (*n* = 4). Data information: Data are shown as mean ± SD. *P < 0.05, **P < 0.01, ***P < 0.001, ns, not significant. Unpaired *t*‐test in (D); Multiple *t*‐test are followed by Bonferroni correction in (F); one sample *t*‐test versus hypothetical mean 1 in (K). Experimental numbers indicate biological replicates. Source data are available online for this figure.

Our data demonstrated that COL is dependent on FPR2 to promote efferocytosis and alleviation of DSS‐induced colonic damage and inflammation. However, whether COL directly targets FPR2 and how the FPR2‐associated signaling is provoked remained unclear. WKYMVm (W‐peptide) is a potent FPR2 agonist that activates FPR2 and induces calcium mobilization, membrane recruitment of β‐arrestin, FPR2 internalization, cAMP response and classic phosphorylation pathways without bias (Ge *et al*, 2020b). To understand the effect of COL on FPR2 signaling, we carefully characterized the signaling events downstream of FPR2. Surprisingly, we found that COL failed to trigger calcium mobilization directly in mouse and human FPR2‐overexpressing RBL cells (Fig 7A, Appendix Fig S5C and D), but pre‐treatment with COL potentiated W‐peptide‐induced calcium mobilization, which was abolished by WRW4 (Appendix Fig S5E and F). We also found that COL stimulated a dose‐dependent cAMP response (reduction in cytosolic cAMP accumulation, Fig 7B), which was a function of the Gαi proteins coupled to FPR2 and could be inhibited by WRW4 (Fig 7C and D). These biased signaling triggered by COL was also observed in hFPR1‐overexpressing cells (Appendix Fig S5G and H). COL at 10 μM induced membrane recruitment of β‐arrestin in hFPR2‐expressing cells (Fig 7G and H). COL stimulated hFPR2 internalization at 1 μM (Fig 7E and F), further confirming that it is a ligand of FPR2. To investigate whether COL could induce conventional GPCR‐mediated phosphorylation signals, we detected phosphorylated ERK1/2, p38, JNK, AKT, and PKA in hFPR2‐expressing RBL cells. However, none of them showed obvious phosphorylation after COL stimulation (Appendix Fig S5I and J). Interestingly, we found that COL induced pseudopodium shrinkage within 5 min of treatment in cells expressing a GFP‐hFPR2 construct, and the effect was stronger than that of W‐peptide (Appendix Fig S5K). The above data imply that COL selectively activates FPR2‐mediated cellular activities and is therefore a biased agonist of FPR2.

RAC1 is a crucial factor for regulation of phagocytosis through actin cytoskeleton rearrangement (Cox *et al*, 1997), which can be activated by FPR2 agonists (Leoni *et al*, 2013; Liu *et al*, 2019). We determined RAC1 binding of GTP in the COL‐treated macrophages and hFPR2‐expressed RBL cells. Our data showed that COL increased the level of GTP‐bound RAC1 in both cell types, and this effect was abolished by the FPR2 antagonist WRW4 (Fig 7I–K). Taken together, these results suggest that COL enhances efferocytosis of macrophages through biased signaling through FPR2 that involves the RAC1 and cAMP signaling pathways.

### COL directly binds to FPR2 and alters its conformation for biased signaling

**Figure 8:**
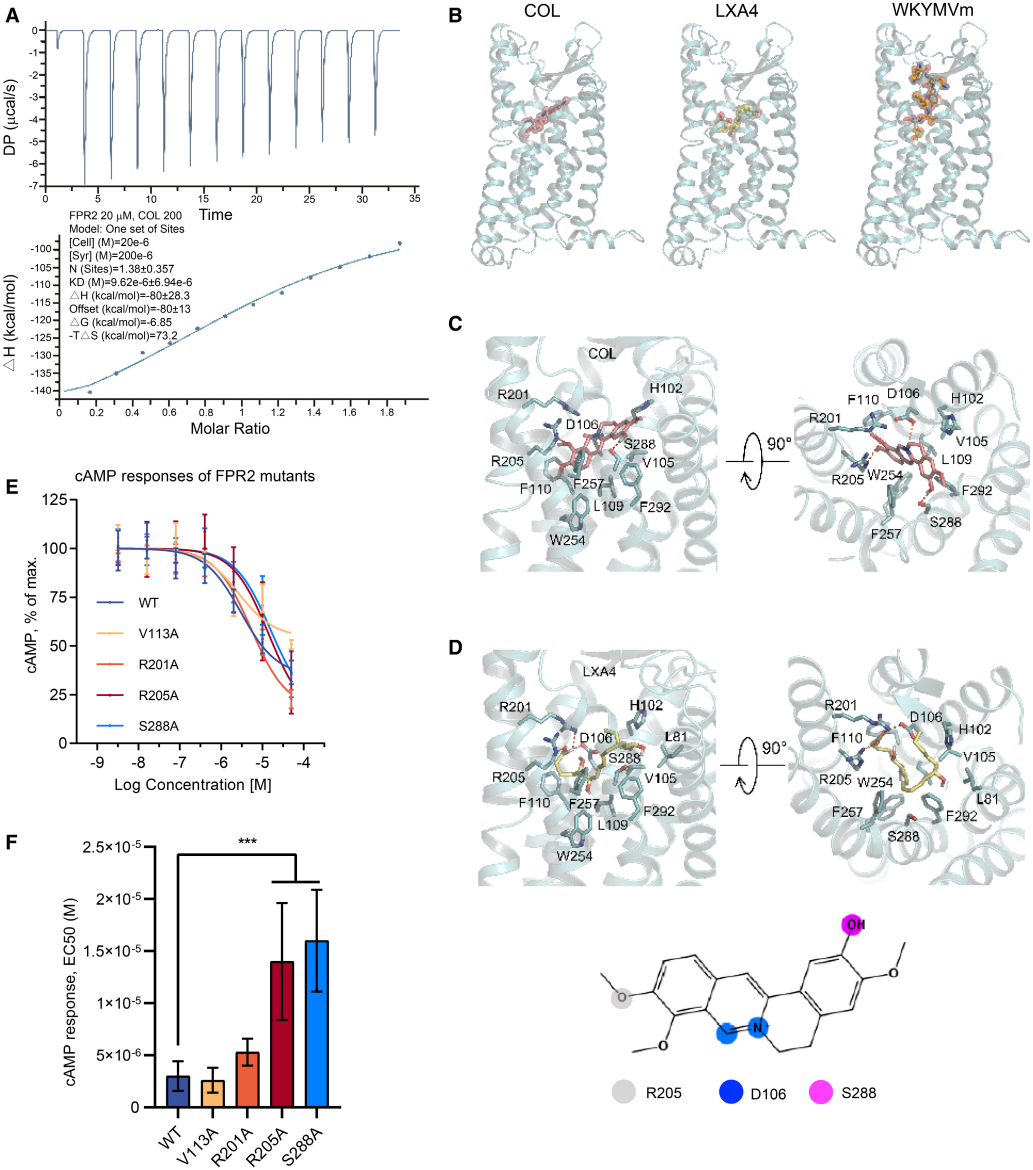
Characterization of binding between COL and FPR2 Measurement of the binding between COL (200 μM) and FPR2 (20 μM) by Isothermal titration calorimetry.Comparison of ligand binding poses in FPR2 including COL (left, docking prediction), LXA4 (middle, docking prediction), and WKYMVm (right, PDB ID: 6LW5). COL occupied a horizonal pose in FPR2 similar to LXA4 but different from the vertical pose of WKYMVm. The ligands are shown in sphere and sticks. FPR2 is shown as cyan ribbons.Detailed interactions between COL and amino acids in the FPR2 binding pocket, viewed from transmembrane side (left, side view) and extracellular side (right, top view). The polar connections between COL and FPR2 are composed of D106, R205, and S288, and the hydrophobic interactions involve H102, V105, L109, F110, W254, F257, F292, and the guanidino group of R205.Detailed interactions between LXA4 and amino acids in the FPR2 binding pocket. Hydrophobic interactions between LXA4 and FPR2 involve L81, H102, V105, L109, F110, W254, F257, F292. Besides the hydrogen network of D106‐R201‐R205, S288 is important for recognition of LXA4 by FPR2. The polar interactions are indicated as red dashed lines. The atoms of COL participated in polar interaction with FPR2 were highlighted and colored inset on the chemical structure.Dose–response curve of COL‐induced inhibition of cAMP production in HEK293 cells expressing different FPR2 mutants (*n* = 3).Median effective concentration of COL for inhibition of cAMP responses in cells expressing the FPR2 mutants (*n* = 3). Data information: Experimental numbers in (F) indicate biological replicates. Data are shown as mean ± SD in (E and F). ***P < 0.001. Source data are available online for this figure.

Our data indicate that COL targets and activates FPR2, but whether COL directly binds to FPR2 is unknown. We used isothermal titration calorimetry (ITC) to access the direct binding between COL and FPR2. The dissociation constant (Kd) was determined to be 9.62 μM with a binding stoichiometry (*n*) of 1.38 at 25°C (Fig 8A).

To investigate the binding mode by which COL binds to FPR2, molecular docking of COL‐FPR2 binding was performed based on the recently published 3‐dimensional structure of FPR2 (Zhuang *et al*, 2020). Unlike WKYMVm which inserts itself deeply into the FPR2 transmembrane helical bundle, COL binds with FPR2 in a relatively shallow and transversal pattern, similar to lipoxin A4 (LXA4), a well‐known anti‐inflammatory FPR2 ligand (Maderna *et al*, 2010) (Fig 8B). Calculation revealed that R205, D106, and S288 are the potential COL‐FPR2 binding sites (Fig 8C), of which R205 and D106 have been identified for W‐pep binding (Chen *et al*, 2020), and S288 is the COL‐specific binding site not previously been shown to interact with W‐pep. These binding sites are largely similar to the predicted LXA4‐FPR2 interaction (Fig 8D), in which D106, R205 and S288 are also involved. To verify the functional binding sites, we established cell lines expressing mutant FPR2 with alanine substitutions (R205A and S288A). Of note, alanine substitution at D106 disrupted the FPR2 conformation and was not prepared here (Chen *et al*, 2020). cAMP responses assay was utilized for functional measurement. The results showed that R205A and S288A mutations significantly increased EC_50_ of COL, indicating their critical roles in mediating COL activity (Fig 8E and F). The locations of R205 and S288 are shown in Fig 8C and D.

### FPR2 is required in COL‐induced LAP‐associated efferocytosis

**Figure 9:**
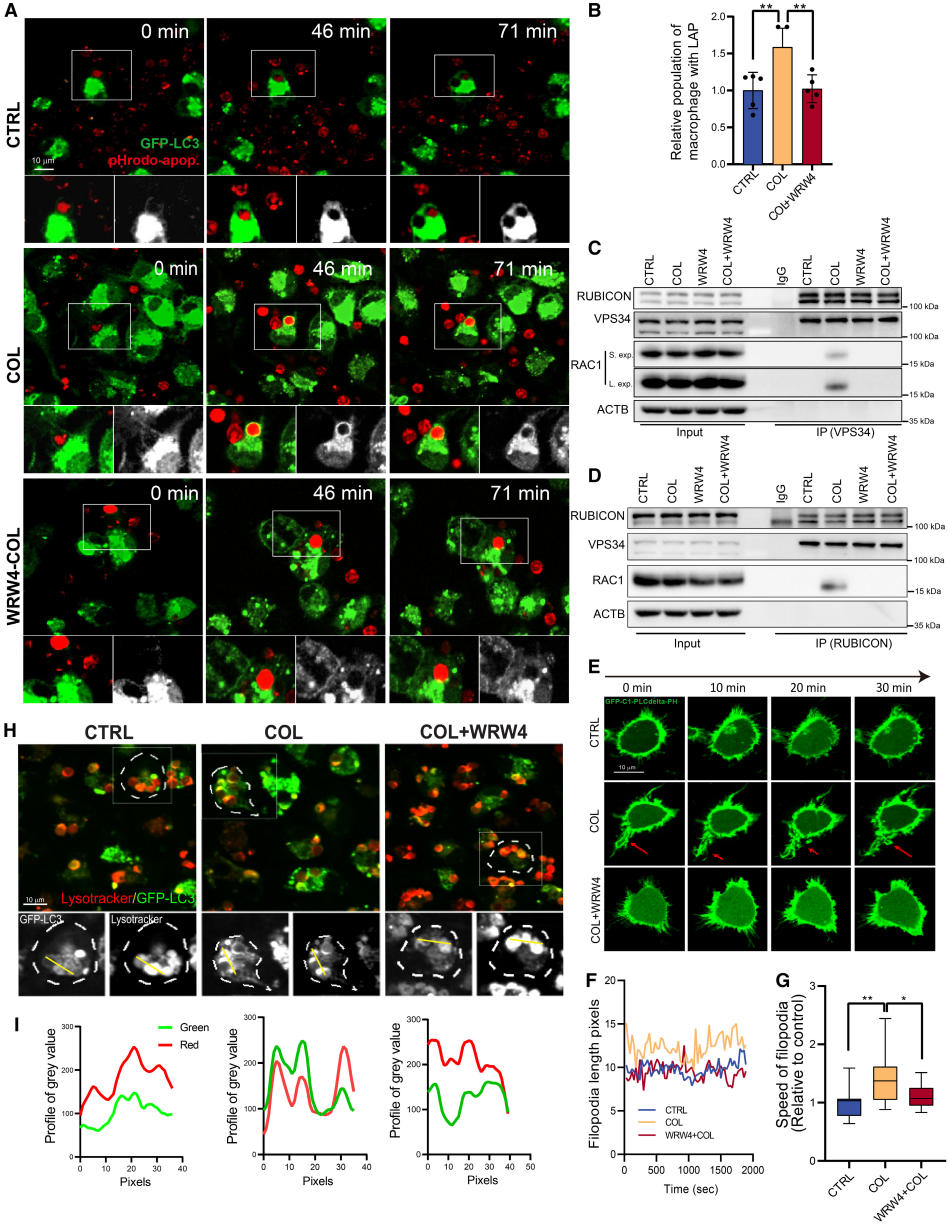
COL stimulates RUBICON‐VPS34‐RAC1 formation and accelerates LC3‐associated efferocytosis ARepresentative images of LC3‐associated phagocytosis after pHrodo‐SE labeled‐ACs incubated with GFP‐LC3 BMDMs at the indicated time points. BMDMs were pre‐treated with 3‐MA (5 mM) or not for 30 min, and COL (1 μM) was added for another 2 h followed by addition of ACs. Green: GFP‐LC3; Red: pHrodo‐SE. Scale bar, 10 μm.BQuantification of LAP efficiency in GFP‐LC3‐BMDMs after different treatment (*n* = 5).C, DCell lysates of BMDMs treated with ACs were subjected to co‐IP by a VPS34 or RUBICON antibody and followed by WB analysis using the indicated antibodies, respectively. BMDM were pre‐treated with WRW4 (30 μM) or COL (1 μM).ERepresentative images of RAW 264.7 cells that transiently transfected with GFP‐C1‐PLCdelta‐PH after treating with COL (1 μM) or not. In WRW4 treated groups, WRW4 (30 μM) were treated for 30 min before adding COL. Red arrow indicates the pseudopodia with high dynamics.FQuantification of filopodia length alteration in different groups using Image J.GQuantification of relative speed of filopodia movement in different groups using Image J. Values are shown in box‐and‐whisker plots and the line in the box corresponds to the median. The boxes go from the upper to the bottom quartiles, and the whiskers go from the minimum to the maximum value (*n* = 14–19).HRepresentative images of GFP‐LC3 BMDMs treated with ACs. BMDMs were pre‐stained with LysoTracker Red for 30 min and pre‐treated with WRW4 (30 μM) for another 30 min in WRW4‐treated groups. ACs were added after COL (1 μM) were treated for another 2 h.ILine profiles of intensity of GFP and LysoTracker Red fluorescent value along the yellow line in (H). Data information: Data are shown as mean ± SD. *P < 0.05, **P < 0.01. Multiple *t*‐test are followed by Bonferroni correction. Experimental numbers indicate biological replicates. Source data are available online for this figure.

To determine whether FPR2 is involved in COL‐induced LAP, we used WRW4 to block FPR2 signaling pathway. We found the enhanced LAP in the COL treatment group was totally blocked by addition of WRW4 (Fig 9A and B) (Movies [Link], [Link] and EV6). In addition, WRW4 treatment abolished the interactions between VPS34 and RAC1 or RUBICON and RAC1 (Fig 9C and D). These results indicate that FPR2 is an essential receptor to activate COL‐induced LAP formation. As RAC1 is responsible for cell membrane ruffle dynamic activity during engulfment (Castellano *et al*, 2000), we applied PLCdelta‐PH plasmid to track cellular pseudopodium dynamic activity after COL treatment. We found that COL increased PLCdelta‐PH‐labeled pseudopodium dynamic activity, which was blocked by WRW4 (Fig 9E–G). Furthermore, the enhancement of GFP‐LC3 recruitment to phagosome after COL treatment was also blocked by WRW4 (Fig 9H and I). Collectively, these data indicate that FPR2 is indispensable for COL‐induced LC3‐associated efferocytosis by facilitating RAC1 activation and RAC1‐RUBICON‐VPS34 interaction.

## Discussion

Aiming to discover a new pharmacological tool to regulate efferocytosis for alleviation of inflammation, we identified COL as a natural compound to enhance macrophage efferocytosis and alleviate colitis in a mouse model. Mechanistic study reveals that COL enhances efferocytosis by directly binding to FPR2, leading to biased signaling. Our findings not only establish the link between FPR2 and efferocytosis, but also strongly support the notion that pharmacological enhancement of efferocytosis is a therapeutic strategy for treatment of inflammatory diseases.

FPR2 is a dual‐function GPCR mediating both pro‐inflammatory and anti‐inflammatory responses, by binding with diverse ligands (Zhang *et al*, 2020). Several FPR2 agonists have been shown to exhibit anti‐inflammation effects on a variety of disease models (Vong *et al*, 2012; Kim *et al*, 2013; Leoni *et al*, 2013; Birkl *et al*, 2019). However, most of these agonists are either peptides or lipids with poor stability and bioavailability. There have been efforts in finding small molecular FPR2 ligands with anti‐inflammatory and pro‐resolving activities (Qin *et al*, 2017, 2022).

Our study has identified COL as a unique small molecule biased agonist of FPR2 which selectively inhibited adenylyl cyclase to reduce cellular cAMP without affecting calcium signaling. Recent structural data revealed the binding pattern of FPR2 with its ligands, providing the structural basis for activation of FPR2 toward pro‐inflammatory or anti‐inflammatory signaling (Chen *et al*, 2020; Zhuang *et al*, 2020). Molecular docking data indicate that COL binds to the functional residues R205 and S288. This binding pattern only partially overlaps with the binding pattern of classic agonist WKYMVm (R205, R201). Unlike WKYMVm which deeply inserts into the FPR2 transmembrane helical bundle, COL displays a relatively shallow and transversal binding pattern. This binding is strong enough to trigger conformational change of FPR2 for a cAMP response, but does not trigger calcium signaling. Therefore, we speculate that the relatively shallow binding pattern of COL may contribute to the biased signaling through FPR2 toward anti‐inflammatory signaling. This new working model may expand our understanding regarding the mechanisms for FPR2 activation. Of note, when docking analysis was conducted with LXA4, a ligand of FPR2 known for its anti‐inflammatory activity (Maderna *et al*, 2010), a similar transversal pose in the FPR2 binding pocket was also observed (Ge *et al*, 2020a). In this regard, COL and LXA4 may share the anti‐inflammatory mechanism through biased signaling at FPR2 including selective activation of the Giα proteins for inhibition of cAMP production but not calcium signaling (Ge *et al*, 2020b).

Previous studies have revealed that FPR2 agonists are involved in the regulation of phagocytosis (Scannell *et al*, 2007; Maderna *et al*, 2010; Tzelepis *et al*, 2015). However, a convincing connection between FPR2 and efferocytosis has not been established. Using FPR2 antagonist and *Fpr2* knockout mice, we showed that COL‐induced FPR2 activation enhanced efferocytosis both *in vitro* and *in vivo*. In searching for the mechanism by which FPR2 regulates efferocytosis, we found that activation of FPR2 by COL activates RAC1 and promotes RAC1‐RUBICON‐VPS34 complex formation to trigger LC3 recruitment on the AC‐containing phagosomes (efferocytosome), thereby facilitating efferocytosome maturation and degradation. This novel mechanism for FPR2 signaling may explain, at least in part, how certain FPR2 ligands can act as anti‐inflammatory agents.

Columbamine is an isoquinoline alkaloid firstly found in calumba (*Jateorhiza palmata* (Lam.) Miers), but can also be found in other plants, such as *Coptis chinensis* Franch., *Fibraurea tinetoria* Lour and *Berberis thunbergia* DC. These plants were traditionally used for alleviating inflammatory symptoms in traditional Chinese medicine and widely applied for therapy of gastrointestinal diseases. However, the anti‐inflammatory mechanism remained unclear. A previous study has found that COL inhibits anti‐inflammatory signal in RAW264.7 cells (Liu *et al*, 2010), but the effective dose used in the study was 25 to 100 μM, which is far beyond the concentration (1 μM) in this study. Furthermore, according to the PK/PD study of COL in a previous report (Xie *et al*, 2020), COL can be effective for at least 12 h after oral administration (10 mg/kg) or intravenous injection (1 mg/kg). Our study has identified a novel anti‐inflammation target of COL with a lower effective concentration and a potential value for clinical application.

Our data here suggests that pharmacologically enhancing macrophage efferocytosis alleviates colitis symptoms, supporting a new therapeutic strategy for IBD, a condition affecting millions of people. Intestine contains the largest population of macrophages which play a central role in maintaining immune homeostasis. Increasing evidence supports a causal link between macrophage dysregulation and IBD development, implying that macrophages can be a new therapeutic target for IBD. In accordance with this link, our data show that depletion of macrophages abolishes the anti‐IBD activity of COL. Though efferocytosis capacity in tissue is huge, accumulation of ACs in colon tissue correlates with disease severity, suggesting that aberrant efferocytosis is associated with disease progression. Enhancing macrophage efferocytosis not only prevents secondary pro‐inflammatory necrotic cell death, but also triggers differentiation of macrophages into anti‐inflammatory phenotype to promote inflammation resolution and tissue repair. Collectively, our data, together with previous reports, suggest that IBD is one of the diseases suited for efferocytosis enhancement therapy.

## Materials and Methods

### Animals

Wild‐type male C57BL/6J mice were purchased from the animal facility of the Faculty of Health Sciences of University of Macau. The *FPR2* ^−/−^ mice were generated by BRL Medicine Inc. (Shanghai, China) and their WT littermate controls derived from interbreeding of heterozygous C57BL/6J mice were genotyped by PCR with a forward primer (5′‐GTCTCAATCCGATGCTC‐3′) and reverse primer (5′‐GGTGAAGTAGAACTGGTGC‐3′). GFP‐LC3 mice in C57BL/6J background were established (Helfand *et al*, 2003) and provided by N. Mizushima (Tokyo Medical and Dental University, Japan). Mouse genotypes were determined by PCR. Mice were housed under controlled temperature (25°C) on 12 h light–dark cycles, with *ad libitum* access to food and water. All animal procedures were conducted at the University of Macau using guidelines approved by the University's Animal Ethics Committee (UMARE‐023‐2021).

### Reagents, antibodies, and plasmids

Columbamine was purchased from Chengdu Must Bio‐Technology Co. Ltd (Chengdu, China) or synthesized by Prof. Guoyuan Zhu in Macau University of Science and Technology (Tao *et al*, 2020). *O*‐dianisidine dihydrochloride (D3893) and disodium clodronate tetrahydrate (88416‐50‐6) were purchased from TCI (Shanghai) Development Co. Ltd. (Shanghai, China). RNAiso Plus (NO.9108) was purchased from Takara (Kusatsu, Shiga, Japan). LysoTracker Red (L7528), pHrodo red AM (P35372), Lipofectamine™ 3000 (L3000015), Active RAC1 Pull‐Down and Detection kit (16118), FlAsH tetracysteine tag detection kit (T34561), and FluoSpheres™ Fluorescent Microspheres (F8887) were purchased from ThermoFisher Scientific (MA, USA). Hexadecyltrimethylammonium bromide (HTAB) was purchased from Sigma‐Aldrich (St. Louis, MO, USA). CytoTrace™ Green CMFDA was purchased from AAT BioQest (Sunnyvale, CA, USA). cAMP detection kit was purchased from Cisbio (Codolet, France). Calcium 5 assay kit was purchased from Molecular Device (San Jose, CA, USA). WKYMVm and WRW4 were obtained from ChinaPeptide Co., Ltd. (Shanghai, China). 3‐Methyladenine (3‐MA) was purchased from Xianding Biotechnology Co., Ltd (Shanghai, China). DAB kit (SK‐4100) was obtained from Vector Laboratories (Burlingame, CA, USA).

Goat anti‐Mouse IgG cross‐adsorbed secondary antibody (1:200, A21422), anti‐RAC1 (1:1,000, MA5‐37658), and anti‐F4/80 (1:1,000, 14‐4801‐82) antibodies were purchased from ThermoFisher Scientific (MA, USA). Anti‐P‐p38 (1:1,000, 4511), anti‐p38 (1:1,000, 54470), anti‐P‐ERK1/2 (1:1,000, 4370), anti‐ERK1/2 (1:1,000, 4695), anti‐P‐JNK (1:1,000, 9255), anti‐P‐AKT (1:1,000, 4060), anti‐rubicon for WB (1:1,000, 8465) and anti‐P‐PKA (1:1,000, 9621) antibodies were purchased from Cell Signaling Technology (MA, USA). Anti‐GAPDH (1:3,000, AC001), anti‐β‐Actin (1:3,000, AC026), HRP Goat anti‐Rabbit IgG (1:10,000, AS014), and HRP Goat anti‐Mouse IgG (1:10,000, AS003) antibodies were purchased from ABclonal Technology (Wuhan, China). Anti‐CD68 (1:50, sc‐20060), anti‐FPR2 (1:50, sc‐57141), anti‐VPS34 (1:200, sc‐11427), anti‐Rab7 (1:200, A12308), and anti‐NOX2 (1:200, sc‐130543) antibodies were purchased from Santa Cruz Technology (CA, USA). Anti‐LC3 (1:5,000, NB100‐2220) antibody was purchased from Novus Biologicals (CO, USA). Anti‐RUBICON (GTX129096) for IP was obtained from GeneTex (1:1,000, Irvine, CA, USA).

### Plasmids

Plasmids coding for hFPR1, GFP‐FPR2, hFPR2(WT), mFPR2 were described previously (Ge *et al*, 2020b). GFP‐C1‐PLCdelta‐PH was a gift from Tobias Meyer (Addgene plasmid #21179; http://n2t.net/addgene:21179; RRID:Addgene_21179) (Stauffer *et al*, 1998). GFP‐2xFYVE was a gift from Harald Stenmark (Addgene plasmid # 140047; http://n2t.net/addgene:140047; RRID:Addgene_140047) (Gillooly *et al*, 2000).

### Cell culture

Human embryonic kidney cell line HEK293 cell (CRL‐1573), human cervical epithelial carcinoma cell line HeLa cell (CCL‐2), murine macrophage cell line RAW264.7 cell (TIB‐71) and rat basophilic leukemic cell line RBL‐2H3 cell (CRL‐2256) are purchased from ATCC. hFPR2‐RBL, mFPR2‐RBL, hFPR2‐HEK293, and hFPR2‐GFP‐RBL stable cell lines were established through transfecting related plasmids into RBL‐2H3 cells followed by G418 selection for 2 months (Ge *et al*, 2020b). FPR2 expression was confirmed by RT–PCR. The stable cell lines, HEK293 cell, HeLa cell, RAW264.7 cells and RBL cells were maintained in Dulbecco's modified Eagle's medium (DMEM, Gibco) supplemented with 10% fetal bovine serum (Gibco) and 50 μg/ml penicillin and streptomycin (Gibco). DNA transfection was performed using Lipofectamine 3000 kit according to the manufacturer's manual.

### Screening of efferocytosis enhancers

Bone marrow‐derived macrophages were plates in 96‐wells and pre‐treated with compounds (10 μM) in library. On the same day, thymocytes were isolated from 4 to 6 week‐old mice and stained with CMFDA for 30 min. Then apoptosis was induced by adding 20 ng/ml TNF‐α. After 24 h, BMDMs were co‐cultured with CMFDA‐labeled apoptotic thymocytes at a ratio of 1:10 (BMDMs: thymoctyes). After another 24 h, the medium in each of the 96‐ wells were transferred to a 96‐well black bottom plate. Another 50 μl PBS were added into the original 96‐well plate to wash out any non‐ingested apoptotic cells (ACs) and transfer them into the 96‐well black bottom plate. Finally, the fluorescence intensity was detected with a plate reader (Molecular Devices) (Excitation: 492 nm, emission: 517 nm).

### Cell viability determination

Bone marrow‐derived macrophage (BMDM) were treated with columbamine (COL) at different concentration. The cell viability was evaluated by using Cell Counting Kit‐8 (CCK‐8) with the experimental conditions as the CCK‐8 manual.

### Phagosome maturation detection

ACs were pre‐stained with pHrodo‐SE and added to BMDMs that were pretreated with vehicle or COL for 2 h. Red fluorescence signal emitted from ACs while maturing was captured by fluorescence microscopy every 30 min. Quantification of fluorescent signal was analyzed by Images J.

### DSS‐induced mouse colitis model

Male C57BL/6J mice (littermates, 8‐week‐old) were administrated with 3% DSS‐contained drinking water for 5 days, followed by normal drinking water for another 2 days. Body weight, fecal blood, and stool consistency were measured daily. DAI score was defined as follows: weight loss: 0 (no loss), 1 (1–5%), 2 (5–10%), 3 (10–20%), and 4 (> 20%); bleeding: 0 (no blood), 1 (hemoccult positive), 2 (hemoccult positive and visual pellet bleeding), and 4 (gross bleeding); stool consistency: 0 (normal), 2 (loose stool), and 4 (diarrhea). On the day of sacrifice, mice were anesthetized and blood was collected via cardiac puncture. The colon and spleen were collected and the length and weight measured. Each colon was divided into four parts. A 0.5 cm segment of distal colon was placed into 4% paraformaldehyde; the remaining colon was divided into three parts, one for MPO analysis, and the remaining two parts were stored at −80°C for further analysis.

### Histology

Colon tissue was fixed overnight in 4% paraformaldehyde, embedded in paraffin, and sectioned into 4‐μm‐thick slices. Slides were stained separately with HE. The histological score represents the sum of the epithelial damage and inflammatory cell infiltration scores, and these scores were evaluated as described previously (Kim *et al*, 2012). For epithelial damage, the scores were calculated as: 0, normal morphology; 1, loss of goblet cells; 2, loss of crypts; and 4, large areas with crypt loss. For inflammatory cell infiltration, the scores were calculated as: 0, no infiltration; 1, infiltration around crypt bases; 2, infiltration reaching the lamina muscularis mucosae; 3, extensive infiltration reaching the lamina muscularis mucosae and thickening of the mucosa with abundant edema; and 4, infiltration of the lamina submucosa.

### Colonic myeloperoxidase (MPO) assay

Myeloperoxidase assay was performed according to a previous study (Kim *et al*, 2012). Tissue samples were thoroughly washed and homogenized in 0.5% hexadecyltrimethyl ammonium bromide in 50 mM PBS at pH 6.0, and centrifuged. MPO was detected in the supernatant by adding 1 mg/ml of o‐dianisidine dihydrochloride and 5 × 10^−4^% H_2_O_2_; the change in optical density at 450 nm was calculated. One unit of MPO activity was defined as the amount that degraded 1.0 mol of peroxide/min at 25°C.

### TUNEL staining

The paraffin‐embedded distal colon region was cut by a microtome at 4 μm. After dewaxing and rehydrating tissue sections according to the standard protocols, the sections were immersed in 0.1 M citrate buffer (pH 6.0), microwaved at 350 W for 5 min, mounted on sides, and incubated with TUNEL reaction mixture for 1 h at 37°C. The TUNEL reaction mixture was made based on the manufacturer's instructions. The nuclei were counterstained with Hoechst 33342. Stained images were captured under Zeiss Axio Observer. For each mouse, 3–5 random fields of the colon were captured per section. TUNEL‐positive areas were quantitatively analyzed by Image J software (NIH). The apoptotic index was determined by calculating the TUNEL‐positive area normalized to the total nuclear area in each field.

### Immunoblotting and co‐immunoprecipitation

Tissue or cells were lysed with RIPA buffer (50 mM Tris–HCl, 1% NP40, 0.35% DOC, 150 mM NaCl, 1 mM EDTA, and 1 mM EGTA, supplemented with protease and phosphatase inhibitor cocktails). The lysates were denatured in 1 × sample loading buffer and resolved by SDS–PAGE, and then the proteins were transferred to a polyvinylidene difluoride membrane. After blocking with 5% non‐fat milk in Tris‐buffered saline containing 0.1% Tween‐20 (TBST), the membranes were incubated with different primary antibodies overnight at 4°C. The proteins were detected by chemiluminescence using an HRP substrate (GE Healthcare) after incubating the membrane with an HRP‐conjugated secondary antibody and washing with TBST. Western blotting images were quantified using Imaging LabTM software (Bio‐Rad).

For co‐immunoprecipitation (IP), cells were homogenized in IP lysis buffer (10 mM Tris–HCl, pH 7.5, 2 mM EDTA, 1% NP40, 150 mM NaCl supplemented with protease and phosphates inhibitors). The lysates were incubated with specific antibodies overnight, followed by incubation with Dynabeads protein G for 4 h. The beads were washed 3–5 times with IP lysis buffer and denatured in 1 × sample loading buffer before the immunoprecipitated proteins were resolved by SDS–PAGE.

### Immunofluorescence staining (IF)

Immunofluorescence staining of colon biopsies was performed similar to IHC staining. The hydrated slides were firstly subjected to antigen retrieval using 0.05 M citrate buffer (pH 6.0) at 100°C for 15 min, and then the sections were blocked and incubated with the appropriate primary antibody overnight at 4°C, followed by incubation with secondary antibodies (Alexa Fluor 488 or 555 conjugated) for 1 h at room temperature. Fluorescent images were captured by using fluorescence microscopy Zeiss Axio Observer system. The positive staining index was determined by calculating the positive staining area normalized to the total nuclear area in each field via ImageJ.

### Immunohistochemistry (IHC)

For immunohistochemistry studies, the slides were deparaffinized and rehydrated. Antigen retrieval was performed by using 0.05 M citrate buffer (pH 6.0) at 100°C for 15 min. After the slides were blocked with 3% H_2_O_2_, the sections were incubated with primary antibody overnight at 4°C, followed by incubation with an HRP‐conjugated anti‐rabbit IgG, and reacted with a peroxidase substrate kit for DAB. Colon tissue was fixed overnight in 4% paraformaldehyde, embedded in paraffin, and processed.

### BMDMs

Cells were obtained by flushing the femur and tibia from male wild type or *FPR2* knockout C57BL/6 mice (8–12 weeks), and the isolated cells were cultured in culture medium (Dulbecco's modified Eagle's medium (DMEM) supplemented with 10% fetal bovine serum (FBS), 10% L929‐conditioned medium, and 1% penicillin and streptomycin). The culture medium was changed every 3 days and cells were collected on day 6–7.

### Efferocytosis detection *in vitro* and *in vivo*

*In vitro*, BMDMs were plated in 24‐well plates and pre‐treated with COL at different concentrations for at least 2 h. Then BMDMs are cultured with CMFDA‐labeled apoptotic thymocytes at a ratio of 1:10 (BMDMs: thymoctyes). After another 24 h, the medium in each well was transferred to a centrifuge tube. Then another 200 μl PBS was added into the original 24‐wells to wash out any non‐ingested ACs. The mixed supernatant and PBS were transferred to the 96‐well black bottom plate and the fluorescence intensity was recorded with a plate reader (Molecular Devices) (Excitation: 492 nm, emission: 517 nm).

*In vivo* AC clearance was determined as previously described with minor modification (Martinez *et al*, 2016). Male mice in WRW4 groups were intraperitoneally (i.p.) injected with 4 mg/kg WRW4. Then COL treatment groups were i.p. injected with 10 mg/kg COL. Subsequently, CMFDA‐labeled apoptotic thymocytes (3 × 10^6^ cells in 100 μl PBS) were injected intravenously into mice. Mice were deprived of drinking water after AC injection. WRW4 was injected again 6 h after AC injections to strengthen FPR2 blocking effects. The mice were finally sacrificed after another 6 h. Mice spleen and liver were dissected out. The number of CMFDA‐positive events in spleen and liver was measured by LSR Fortessa flow cytometry (BD Biosciences).

### Determination of *in situ* efferocytosis efficiency *in vivo*

Efferocytosis was determined as previous described (Proto *et al*, 2018). Macrophages in tissues were stained by F4/80 or CD11b followed IF staining. ACs were labeled by TUNEL staining. Images were captured by using a Leica DMI8 and analyzed using ImageJ software. The ACs that were co‐localized with or were adjacent to macrophages were count as efferocytosis events. The ratio of associated ACs per tissue section were quantified as efferocytosis efficiency.

### Engulfment ability determination

ACs were pre‐stained with pHrodo‐SE or CMFDA and added to BMDMs or lysotracker‐stained BMDM that had been pretreated with COL for 2 or 24 h. Non‐engulfed ACs were washed out, and images were captured by Opera Phenix Plus High‐content screening system (PerkinElmer) or Zeiss Axio Observer. The engulfment ability was determined by counting the numbers of BMDM and ACs that had been ingested in the cell bodies of BMDMs (Merged ACs‐matched fluorescent channel and bright‐field images) in each field, and the ratio of ingested ACs was taken as representative of engulfment ability.

### Time‐lapse imaging microscopy and analysis

Live‐imaging was performed using Opera Phenix Plus High‐content screening system (PerkinElmer), Leica SP8 (Leica), and Nikon A1R (Nikon) confocal imaging microscopes. Images were captured in an environmental chamber that maintained the temperature at 37°C and provided a humidified stream of 5% CO_2_ air. For the live fusion images of Lysotracker‐stained lysosomes and apoptotic thymocytes, BMDMs were pre‐stained with Lysotracker red DND‐99, and Lysotracker was in the culture medium during the image capture. CMFDA‐labeled apoptotic thymocytes were added to the BMDMs, and images were taken every 30 s for 2–3 h. To track LAP formation, GFP‐LC3 overexpressing BMDMs were co‐cultured with pHrodo‐SE stained ACs. The Nikon A1R system was applied for tracking LAP formation. LAP population was calculated by counting cells with LAP versus total cells.

To track fusions of LAP and lysosome, GFP‐LC3‐expressing BMDMs were pre‐stained with Lysotracker and incubated with apoptotic cells. The time‐lapse images were taken by Opera Phenix Plus High‐content screening system for 2 h. GFP‐LC3 fluorescent intensity of phagosome after 2 h was analyzed by Image J, and the data were normalized with control.

For HeLa cells, Live‐imaging was performed using Leica SP8 (Leica) confocal imaging microscopes. Images were captured under an environmental chamber that maintained the temperature at 37°C and provided a humidified stream of 5% CO_2_ air. GFP‐FPR2 overexpressing HeLa cells were treated with W‐p or COL. Images were taken every 30 s for 2–3 h. In the experiments to track 2xFYVE‐GFP puncta formation in RAW264.7 cells, images were taken every 5 min by Opera Phenix Plus High‐content screening system.

### Macrophage depletion *in vivo*

PBS‐liposome and clodronate liposome were prepared by thin‐film hydration method (Van Rooijen *et al*, 1996). 5 mg/ml clodronate‐liposome were produced for macrophage depletion. The *in vivo* macrophage depletion was performed as previous description with minor modification (Weisser *et al*, 2012). Briefly, clodronate or PBS liposomes (200 μl) were i.p. injected into mice 3 days before DSS administration. Then, clodronate‐ or PBS‐liposome were continuously injected on day 4 and 5 after start DSS administration.

### RNA isolation and RNA‐sequencing

Total RNA from BMDM and COL‐treated BMDMs were extracted using RNAiso Plus according to the manufacture's protocol. RNAs were detected, sequenced, and analyzed by Majorbio (Shanghai, China).

### Gene expression omnibus (GEO) analysis

The data file of GDS1615 in GEO profiles from NCBI was retrieved to analyze the phagocytosis‐related GPCR mRNA level alteration in IBD patients (Burczynski *et al*, 2006). The data were derived from peripheral blood mononuclear cells of 42 healthy persons, 26 UC patients, and 59 CD patients. The expression values were subjected to GraphPad Prism 9.0 analysis.

### Ca^2+^ mobilization and cAMP measurement

FPR2‐RBL cells were loaded with the Ca^2+^ sensitive dye Calcium 5 in HBSS plus 20 mM HEPES. To detect direct agonistic activities, the cells were stimulated with WKYMVm (10 nM) or COL in different concentrations. Ca^2+^ responses were detected using a Flexstation III microplate reader (Molecular Devices).

For cAMP measurement, FPR2‐HEK293 cells were suspended in assay buffer (HBSS plus 5 mM HEPES, 0.1% BSA (w/v) and 0.5 mM isobutylmethylxanthine) and seeded into 384‐well plates. WKYMVm or COL were applied together with 5 μM forskolin to the cells for 30 min. cAMP level was detected with the Cisbio cAMP detection kit according to the manufacturer's instructions.

### FPR2 internalization and β‐arrestin recruitment

For FPR2 internalization detection, FPR2‐GFP‐RBL cells were cultured in confocal dishes. Cells were fixed by adding 4% PFA for 15 min after treatment of WKYMVm or COL for 1 h. For β‐arrestin recruitment, HEK293 cells were transfected with FPR2 or β‐arrestin‐2‐GFP plasmids and cultured in confocal dish. Cells were fixed by adding 4% PFA after treatment of WKYMVm and COL for 15 min. Cell nuclei were stained with Hoechst. Distribution of GFP‐FPR2 and β‐arrestin‐2‐GFP were monitored by using Leica TCS SP8 laser scanning confocal microscope. GFP‐FPR2 internalization was analyzed by counting the GFP‐FPR2 puncta localized in the cytoplasm. β‐arrestin‐2‐GFP recruitment was analyzed by tracking the fluorescence intensity profile (drawing lines from membrane to cytoplasm). 0–1.5 μm represent the distance to the edge of the membrane. All the intensity was normalized to the initial value.

### Detection of active RAC1

Endogenous active guanosine triphosphate (GTP)‐bound RAC1 was pulled down using Active RAC1 Pull‐down and Detection Kit (Thermo Scientific) according to the manufacturer's protocol. FPR2‐RBL or BMDMs was treated with COL (1 μM) for 10 min, and cells were harvested and proteins were extracted in lysis buffer. Extracted proteins were incubated with glutathione resin bound with GST‐human Pak1‐PBD in 4°C for 4 h with gentle rocking. Active RAC1 was eluted by adding reducing sample buffer and then subjected to Western blot analysis.

### Isothermal titration calorimetry (ITC)

Isothermal titration calorimetry measurements were performed using the Microcal‐PEAQ‐ITC (Malvern). Human FPR2 protein was purified as previously described (Chen *et al*, 2020). 280 μl PBS containing 20 μM FPR2 protein was added into each sample chamber. 200 μM COL was titrated into the sample chamber at 25°C. The data were evaluated with the Malvern software using the single set of independent binding sites model.

### Molecular docking analysis

The cryo‐EM structure of FPR2 (PDB ID: 6OMM) was retrieved for docking analysis using the AutoDock Tool (Allouche, 2012). Hydrogen atoms were added to the receptor before running docking analysis in the Python Molecular Viewer (PMV, v1.5.7). The 3D structures of WKYMVm were generated and optimized using Avogadro platform. After ligand preparation, docking of these ligands to FPR2 was performed with the AutoDock tool. The docking parameters were applied as previously described (Chen *et al*, 2022). After analysis, the binding pose chosen from the optimal conformations was presented for the binding sites of the ligands and FPR2.

### Monitoring pseudopodium dynamic activity

RAW264.7 cells were transiently transfected with plasmids coding GFP‐C1‐PLCdelta‐PH. Then 3‐MA or WRW4 were added in indicated dishes for 30 min before COL treatment. A1R confocal laser scanning system (Nikon) was used to monitor the GFP‐C1‐PLCdelta‐PH enriched pseudopodium dynamic activity. Filopodia length and speed were analyzed by FiloQuant plugin in ImageJ (Jacquemet *et al*, 2017, 2019).

### Statistical analysis

All statistical analysis was performed using GraphPad Prism9 software. Randomization was done for the animal grouping. Histology scoring and any images analysis based on personal observation are verified by different observers. There are no exclusion criteria, except absence of dead mice data. The experiments were not blinded. *n* indicates biological replicates throughout the study. The values were expressed as mean ± SEM or SD and *P* < 0.05 were considered significant. To analyze the differences between two groups, unpaired *t*‐test was used. For data normalized with control group (control is 1), one sample *t*‐test against hypothetical value 1 was used to compare. For multiple group comparison, multiple *t*‐test followed by Bonferroni correction or one‐way analysis of variance was used.

## Author contributions

**Jia‐Hong Lu:** Supervision; funding acquisition; project administration; writing – review and editing. **Richard D Ye:** Funding acquisition; project administration; writing – review and editing. **Ming‐Yue Wu:** Data curation; software; formal analysis; validation; methodology; writing – original draft. **Yun‐Jun Ge:** Resources; formal analysis; validation; methodology. **Er‐Jin Wang:** Data curation; validation; visualization; methodology. **Qi‐Wen Liao:** Resources; software. **Zheng‐Yu Ren:** Visualization; methodology. **Yang Yu:** Resources. **Guoyuan Zhu:** Resources. **Chun‐Ping Liu:** Resources. **Huanxing Su:** Writing – review and editing. **Han‐Ming Shen:** Writing – review and editing. **Ye Chen:** Writing – review and editing. **Lei Wang:** Software. **Jin Chai:** Resources. **Yi‐Tao Wang:** Writing – review and editing. **Min Li:** Writing – review and editing. **Zhaoxiang Bian:** Methodology; writing – review and editing. **Meng‐Ni Zhang:** Validation.

## Disclosure and competing interests statement

The authors declare that they have no conflict of interest.

## Supporting information



Appendix

Movie EV1

Movie EV2

Movie EV3

Movie EV4

Movie EV5

Movie EV6

Source Data for Appendix

Source Data for Figure 1

Source Data for Figure 2

Source Data for Figure 3

Source Data for Figure 4

Source Data for Figure 5

Source Data for Figure 6

Source Data for Figure 7

Source Data for Figure 8

Source Data for Figure 9

## Data Availability

RNA‐Seq (Mouse) data generated in this study: Sequence Read Archive (SRA) data (accession: PRJNA1023710) [URL: https://www.ncbi.nlm.nih.gov/sra/?term=PRJNA1023710].
