# Supplementary material for: Enhancement of efferocytosis through biased FPR2 signaling attenuates intestinal inflammation
Source: EMBO Mol Med. 2023 Nov 22;15(12):e17815. doi: 10.15252/emmm.202317815 (PMC10701612; doi:10.15252/emmm.202317815)
Supplement: Supplementary file 1 — Appendix [file EMMM-15-e17815-s003.pdf]

APPENDIX

Enhancement of efferocytosis through biased FPR2 signaling attenuates intestinal inflammation

Ming-Yue Wu[1, 3†], Yun-Jun Ge[2, 11†], Er-Jin Wang[1†], Qi-Wen Liao[2], Zheng-Yu Ren[1], Yang Yu[4], Guoyuan Zhu[5], Chun-ping Liu[1, 6, 7], Meng-Ni Zhang[3], Huanxing Su[1], Han-Ming Shen[8], Ye Chen[9], Lei Wang[6], Yi-Tao Wang[1], Min Li[10], Zhaoxiang Bian[10], Jin Chai[3], Richard D. Ye[2*], Jia-Hong Lu[1, 7*]

Table of Contents

Appendix Table S1. List of top 10 compounds screened from library....2
Appendix Figure S1. Phagosome maturation detection and in situ efferocytosis detection. 3
Appendix Figure S2. Representative images of GFP-2xFYVE expressing RAW264.7 cells after co-cultured with microspheres beads. 5
Appendix Figure S3. Effects of COL on DSS-induced colitis model after blocking LAP by adding 3-MA in vivo. 6
Appendix Figure S4. Confirmation of clodronate-induced macrophage depletion and determination of protective effects of COL on DSS-induced colitis in the presence of FPR2 antagonist. 8
Appendix Figure S5. Measurements of biased signaling through FPR2. 10

Appendix Table S1. List of top 10 compounds screened from library.

List	Chemical	Z-Score	Structure
1	2-(9-bromo-4-methoxy-6-methyl-5,6,7,8-tetrahydro-[1,3]dioxolo[4,5-g]isoquinolin-5-yl)-1-(3-methoxy-phenyl)-ethanone	-1.6	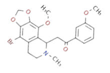
2	Columbamine	-1.46	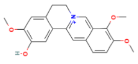
3	8,8-dimethyl-10h-pyrano[2,3-f]chromene-2,9-dione	-1.31	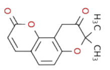
4	n-homoveratroylhomoveratrylamine	-1.3	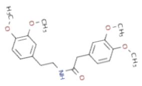
5	1,2,3,4-tetrahydro-6,7-dimethoxy-1-methylisoquinolinyl-8,12dihydro-5,6-epoxy-2-hydroxyalantolactone	-1.24	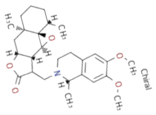
6	7,8-dimethoxy-benzo[d][1,2]oxazin-1-one	-1.22	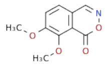
7	5-[2-(6-hydroxy-4,7-dimethoxy-benzofuran-5-yl)-2-oxo-ethyl]-4-methoxy-6,6-dimethyl-5,6,7,8-tetrahydro-[1,3]dioxolo[4,5-g]isoquinolin-6-ium	-1.2	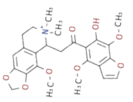
8	brassinolide	-1.17	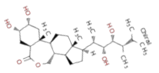
9	chloramphenicol	-1.16	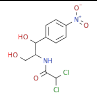
10	allomatrine N1-oxide	-1.16	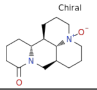

Appendix Figure S1

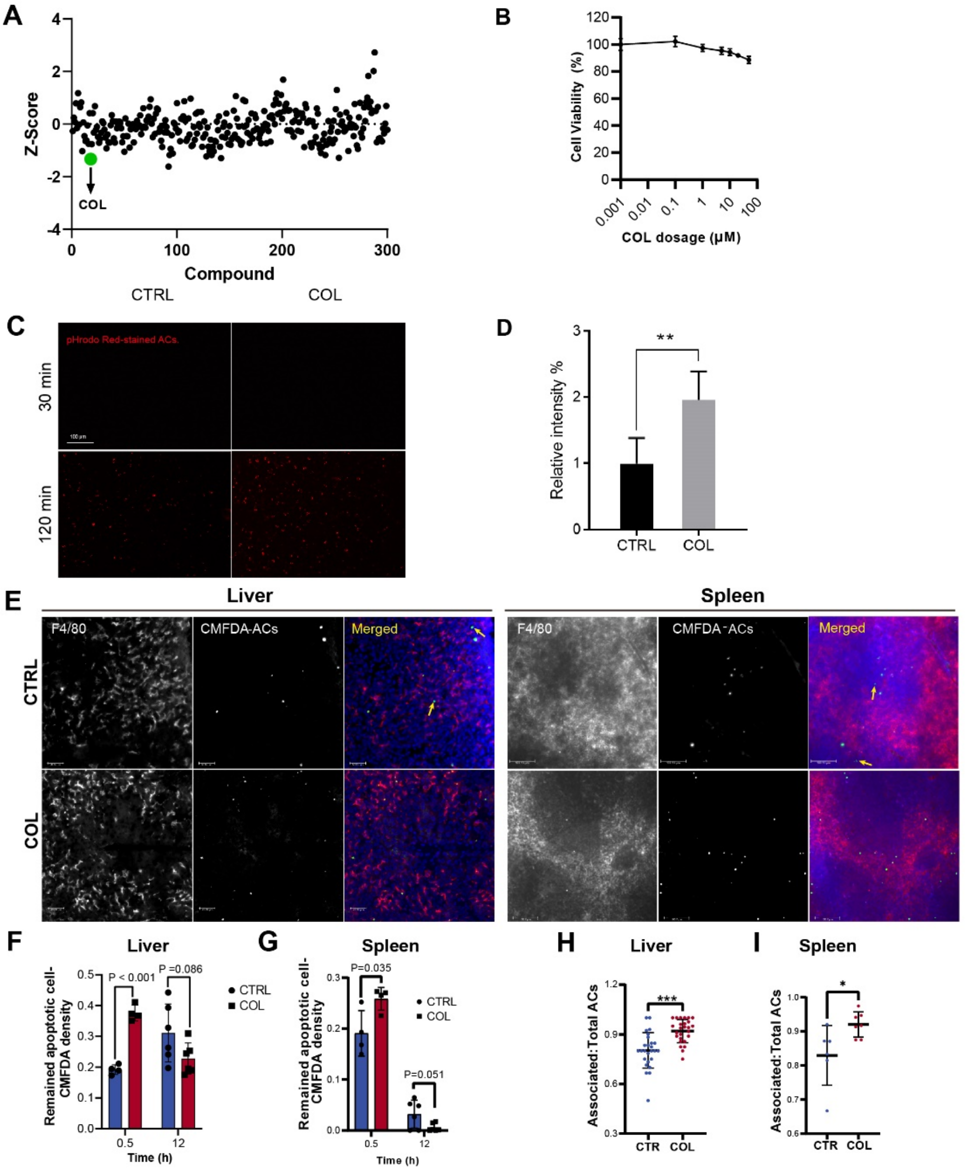

Appendix Figure S1. Phagosome maturation detection and in situ efferocytosis detection.

A. Comparison of compounds-induced efferocytosis capacity as described in Fig. 1A. The COL-treated group was labeled in green color (black arrow).

B. Determination of cell viability after COL treatment by using CCK8.

C. Representative images after co-culturing pHrodo SE-stained ACs and BMDMs for indicated time. Red: pHrodo SE fluorescent signal. BMDMs was pretreated with COL (10 μM) for 24 h. Scale bar, 100 μm.

D. Quantification data of pHrodo SE intensity according to the images in Fig. EV1C (n = 6).

E. Representative images of liver and spleen after injected mice with CMFDA-ACs for 30 min. Red: F4/80, Green: CMFDA-ACs, Blue: DAPI. Arrow: Free ACs. Mice were pretreated with COL (10 mg/kg) or not (*n* = 3), Scale bar, 50 μm (liver), 100 μm (spleen).

F, G. Comparison of CMFDA-ACs intensity in liver and spleen lysates tissue after injected mice with CMFDA-ACs for 30 min and 12 h (495/520). Mice were pretreated with COL (10 mg/kg) or not (*n* = 3).

H, I. Analysis of macrophage-associated ACs and total ACs in liver and spleen slides after staining macrophages with F4/80. Images (2-12 images per mice) were captured and analyzed after injected CMFDA-ACs for 30 min.

Appendix Figure S2

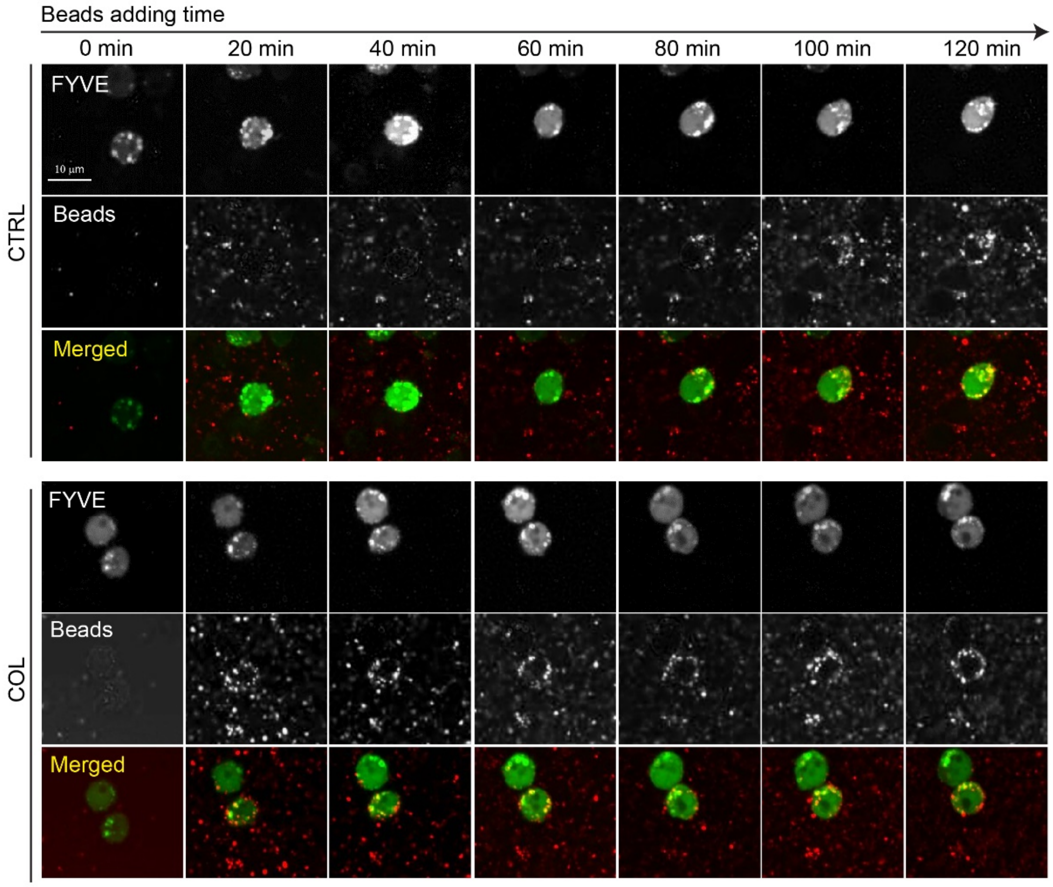

Appendix Figure S2. Representative images of GFP-2xFYVE expressing RAW264.7 cells after co-cultured with microspheres beads.

COL were added into RAW264.7 cells for 2 h, followed by adding microsphere beads (0.1 μm). Green: GFP-2 x FYVE, Red: microsphere beads (0.1 μm) with red fluorescence (580/605).

Appendix Figure S3

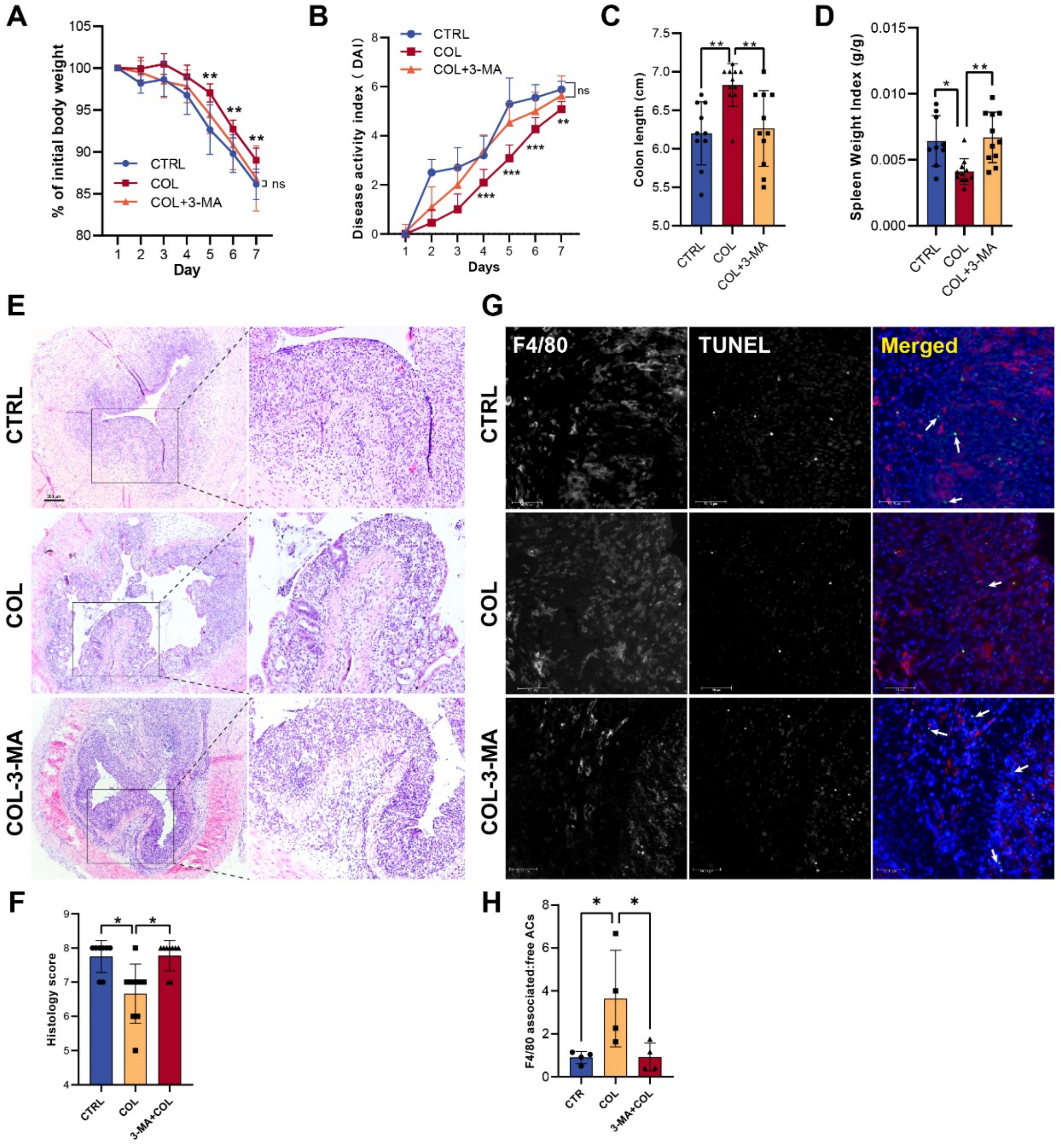

Appendix Figure S3. Effects of COL on DSS-induced colitis model after blocking LAP by adding 3-MA in vivo.

A, B. Comparison of daily body weight and DAI alteration during DSS treatment in different groups (*n* = 9-11). COL (10 mg/kg) or COL plus 3-MA (10 mg/kg) were injected (i.p.) daily, respectively.

C, D. Comparison of colon length and spleen weight after different treatment (*n* = 9-11).

E, F. Comparison of representative HE staining images and histology score based on the observations of HE staining images (*n* = 8-9). Scale bar, 200 µm.

G, H. Comparison of representative fluorescence images (Red: F4/80, Green: TUNEL, Blue: DAPI) and analysis results of F4/80-assocated ACs ratio in different groups (*n* = 4). Scale bar, 50 µm.

Data information: Data are shown as mean ± SD. *$P<0.05$, **$P<0.01$, ***$P<0.001$ or indicated the figures; ns, not significant. Multiple *t*-test are followed by Bonferroni correction.

Appendix Figure S4

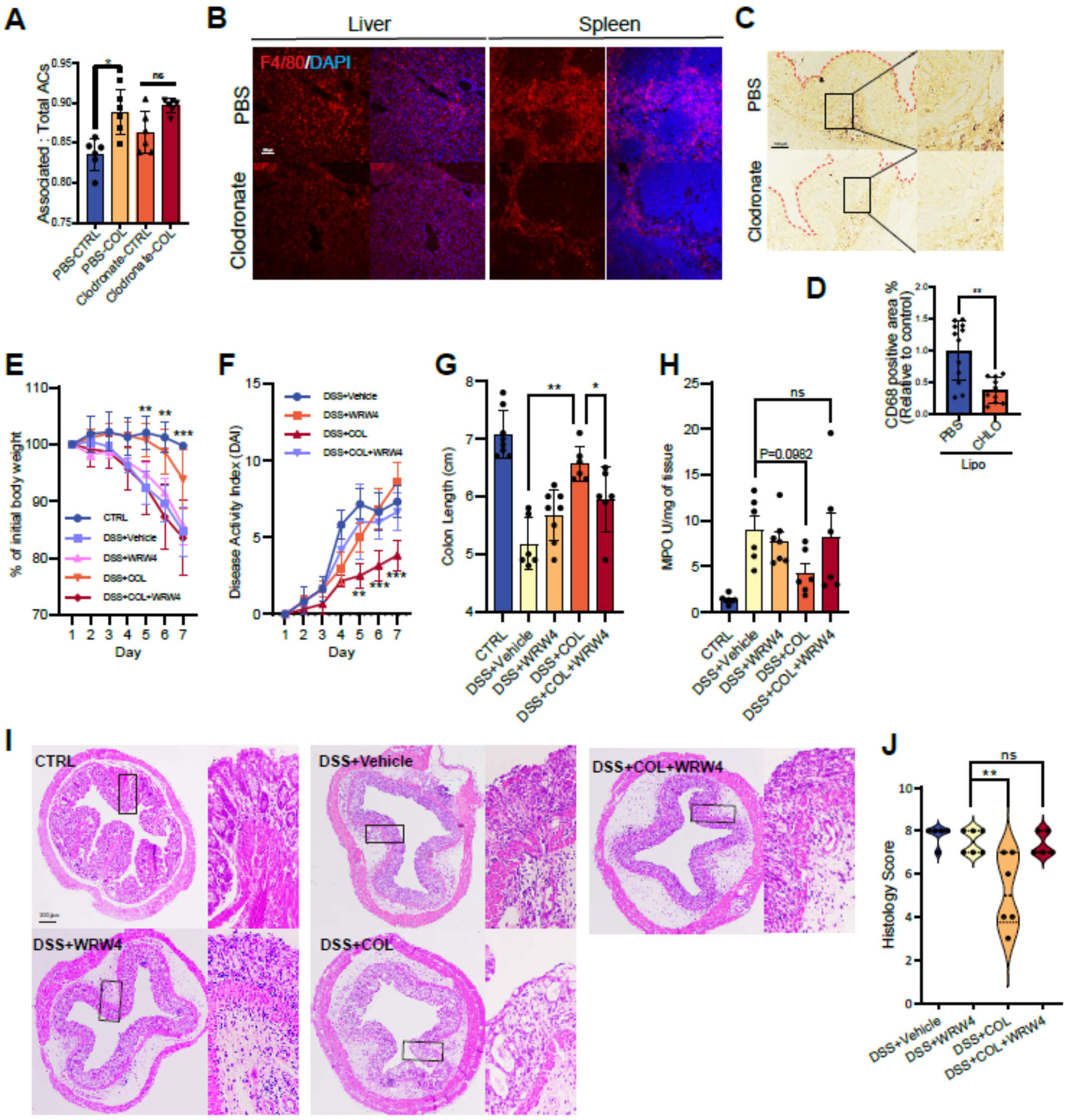

Appendix Figure S4. Confirmation of clodronate-induced macrophage depletion and determination of protective effects of COL on DSS-induced colitis in the presence of FPR2 antagonist.

A. Colonic in situ efferocytosis efficiency in colitis mice with or without clodronate-liposome treatment (10 mg/kg COL treated or not) (*n* > 6 images in each group were analysis).

B. Representative immunofluorescent images of colon slides from mice with PBS-liposome or clodronate-liposome injection (i.v.) for 24 h. Red: F4/80 positive area; Blue, DAPI positive. Scale bar, 100 μm.

C. Representative immunohistochemistry images of colon slides from mice in after DSS treatment with PBS-liposome or clodronate-liposome injection. Brown color: CD68 positive area.

D. Quantification analysis of CD68 positive area (*n* = 10-12).

E, F. Comparison of daily body weight and DAI alteration during DSS treatment in different groups (*n* = 6). COL (10 mg/kg) and WRW4 (4 mg/kg) were injected (i.p.) daily, respectively.

G. Comparison of colon length after different treatment (*n* = 6).

H. Comparison of myeloperoxidase (MPO) activity in tissues among groups (*n* = 6).

I. Representative images after HE staining.

J. Comparison of histology score based on the observations of HE staining images (*n* = 6). Scale bar, 200 μm.

Data information: Data are shown as mean ± SD. $^{*}P<0.05$, $^{**}P<0.01$, $^{***}P<0.001$ or indicated the figures; ns, not significant. Multiple *t*-test are followed by Bonferroni correction.

Appendix Figure S5

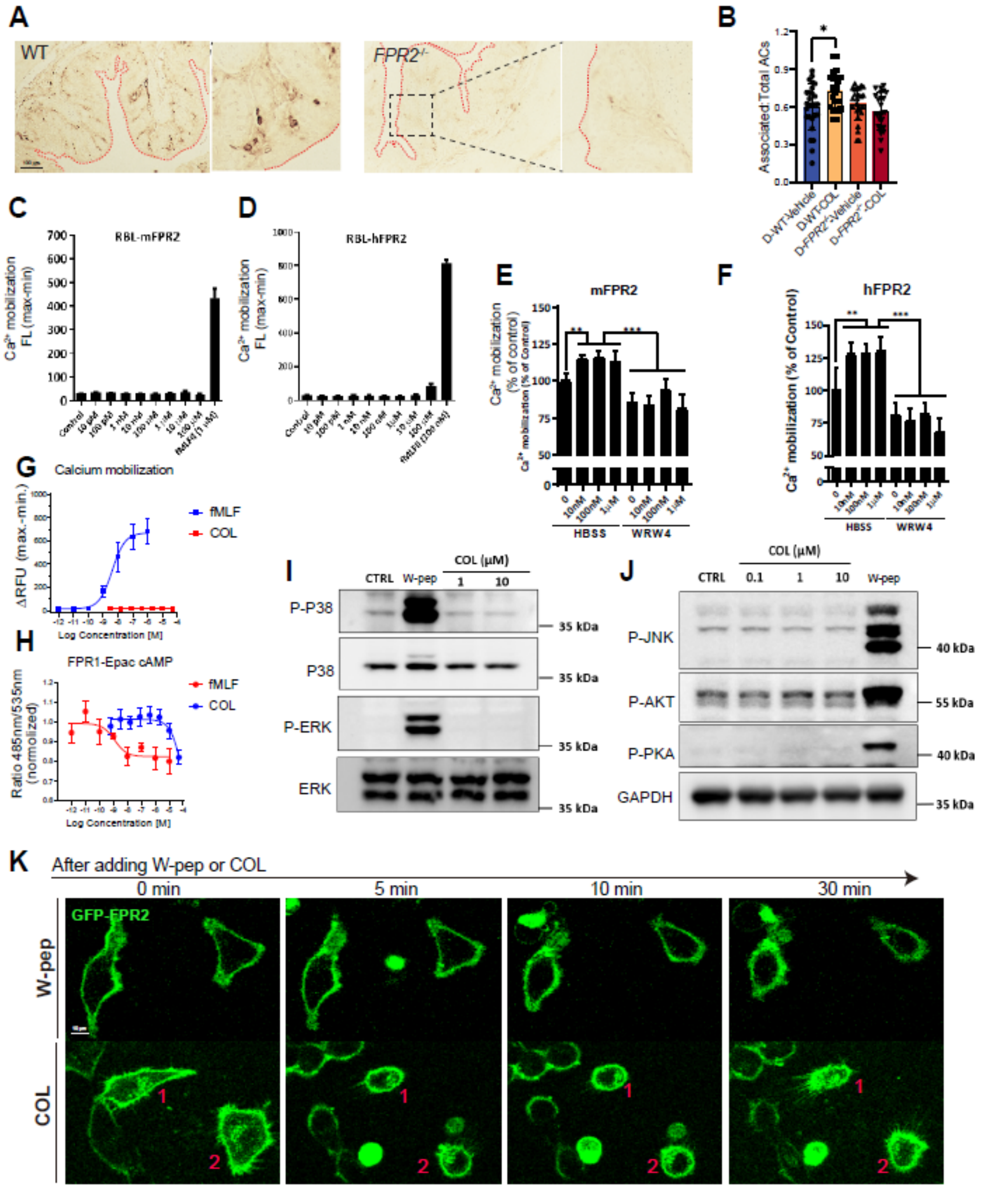

Appendix Figure S5. Measurements of biased signaling through FPR2.

A. Representative immunohistochemistry staining images of colon in wild type (WT) and FPR2$^{-/-}$ mice. Brown: FPR2 positive staining.

B. Colonic in situ efferocytosis efficiency in WT and FPR2$^{-/-}$ mice (10 mg/kg COL treated or not) after DSS treatment (n > 20 images in each group were analysis).

C, D. Calcium mobilization results after treating RBL cells with COL in different concentration (n = 6). RBL cells were stably expressing mouse FPR2 (mFPR2) or human FPR2 (hFPR2).

E, F. W-pep. induced calcium mobilization results after preincubated with mFPR2 or hFPR2

overexpressing RBL cells with COL or COL plus WRW4 (30 μM) in different concentrations for 30 min (*n* = 6).

G, H. Determination of dose-dependent calcium mobilization and dose dependent inhibition of forskolin-stimulated cAMP accumulation in FPR1-overexpressing cell lines (*n* = 6). fMLF is the agonist of FPR1.

I, J . Phosphorylation levels of different protein kinases determination in FPR2-RBL cells. W-pep. (0.1 μM) and COL were added to FPR2-RBL cells for 10 min.

K. Representative images of GFP-FPR2-expressing Hela cells after treating cells with W-pep. (0.1 μM) or COL (1 μM) at indicated time points. Green: GFP-FPR2; Number in red color: cells with strong morphology change.

Data information: Data are shown as mean ± SD. **P<0.01, ***P<0.001; ns, not significant. Multiple t-test are followed by Bonferroni correction.
