# Supplementary figures and images for: Enhancement of efferocytosis through biased FPR2 signaling attenuates intestinal inflammation (part 1 of 2)
Source: EMBO Mol Med. 2023 Nov 22;15(12):e17815. doi: 10.15252/emmm.202317815 (PMC10701612; doi:10.15252/emmm.202317815)

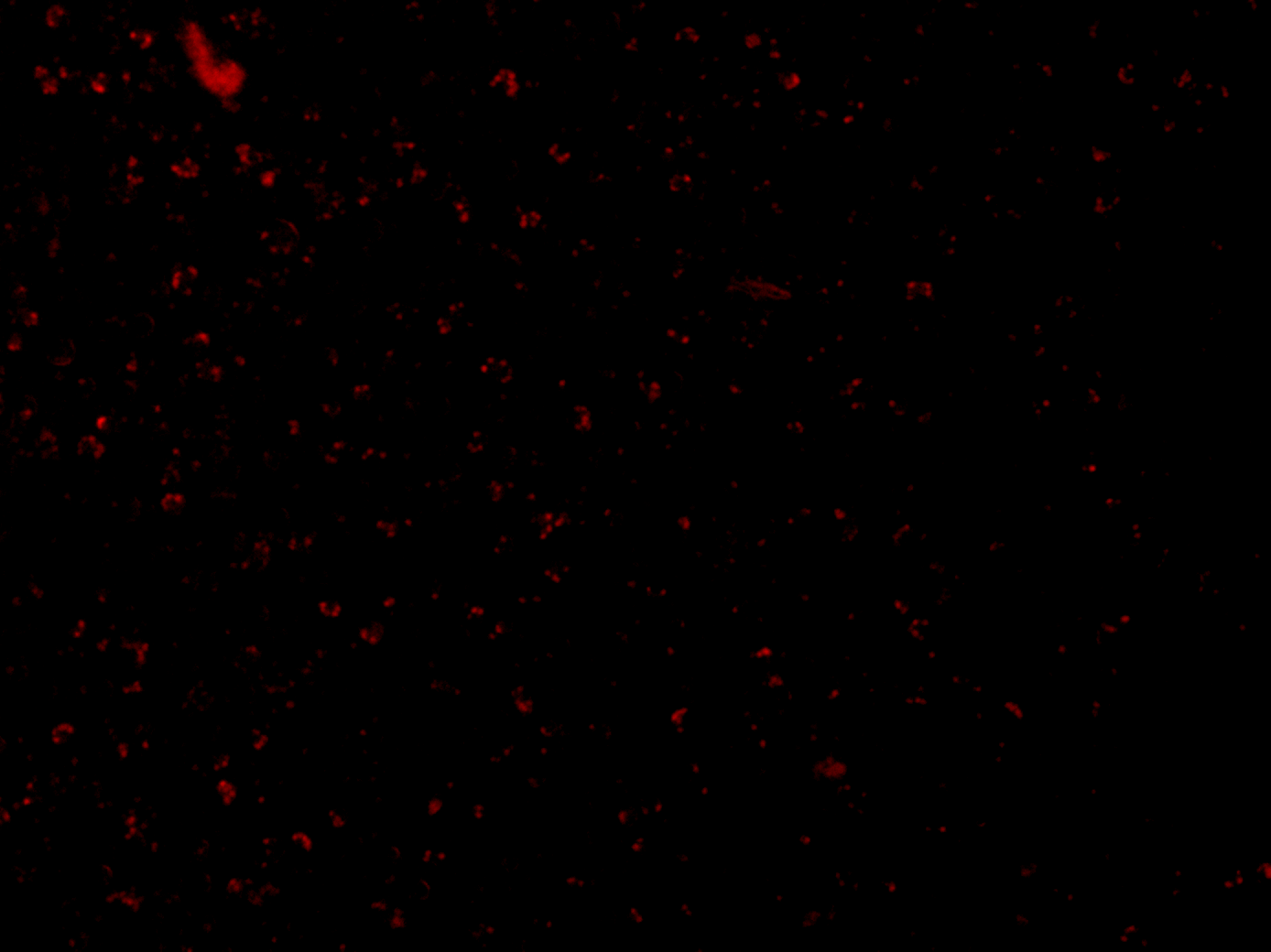

Supplement: Supplementary file 8 — Source Data for Appendix [file EMMM-15-e17815-s004.zip › Appendix_FigS1D/120min/col10-120min.tif]

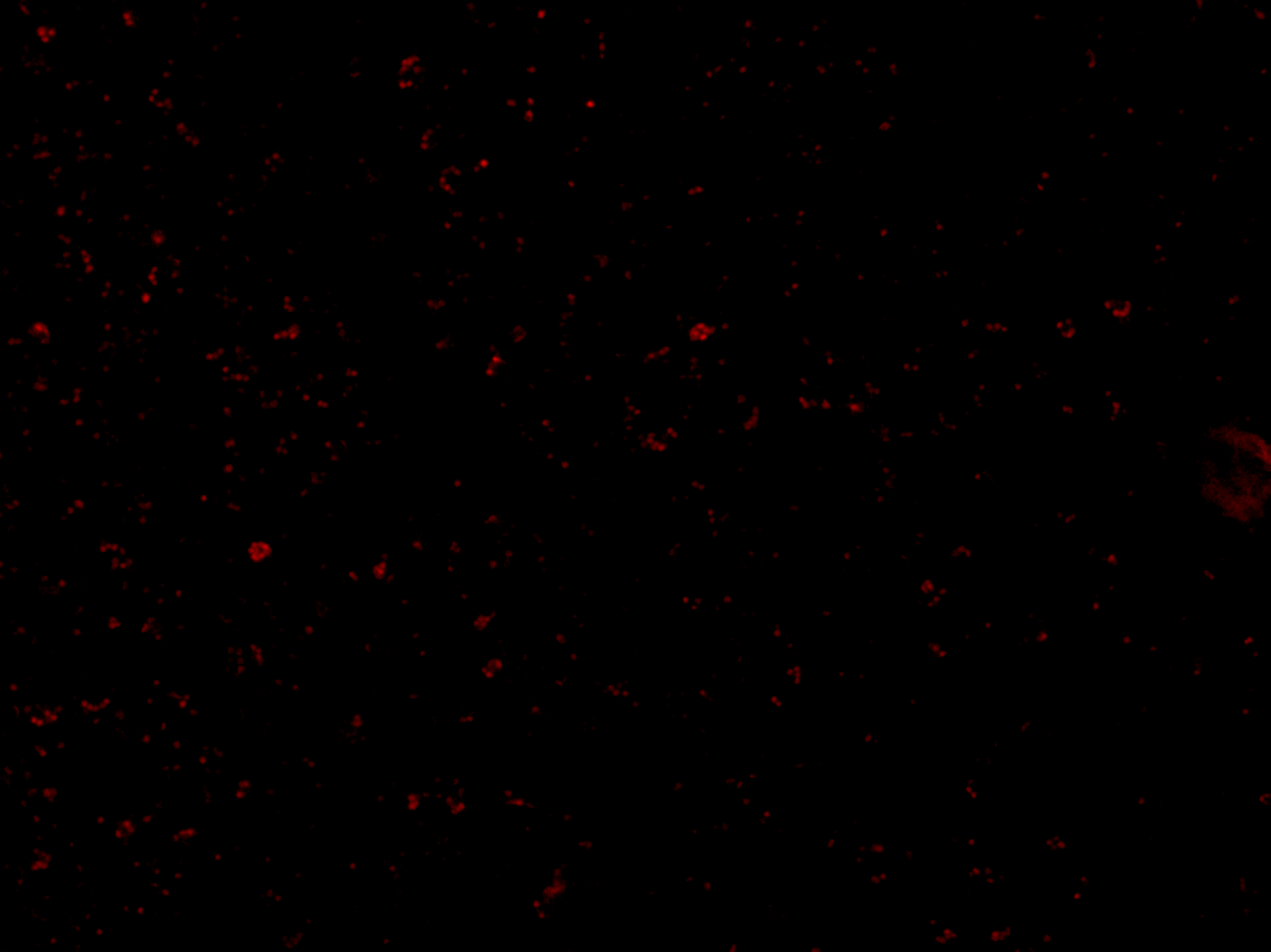

Supplement: Supplementary file 8 — Source Data for Appendix [file EMMM-15-e17815-s004.zip › Appendix_FigS1D/120min/ctrl-120min.tif]

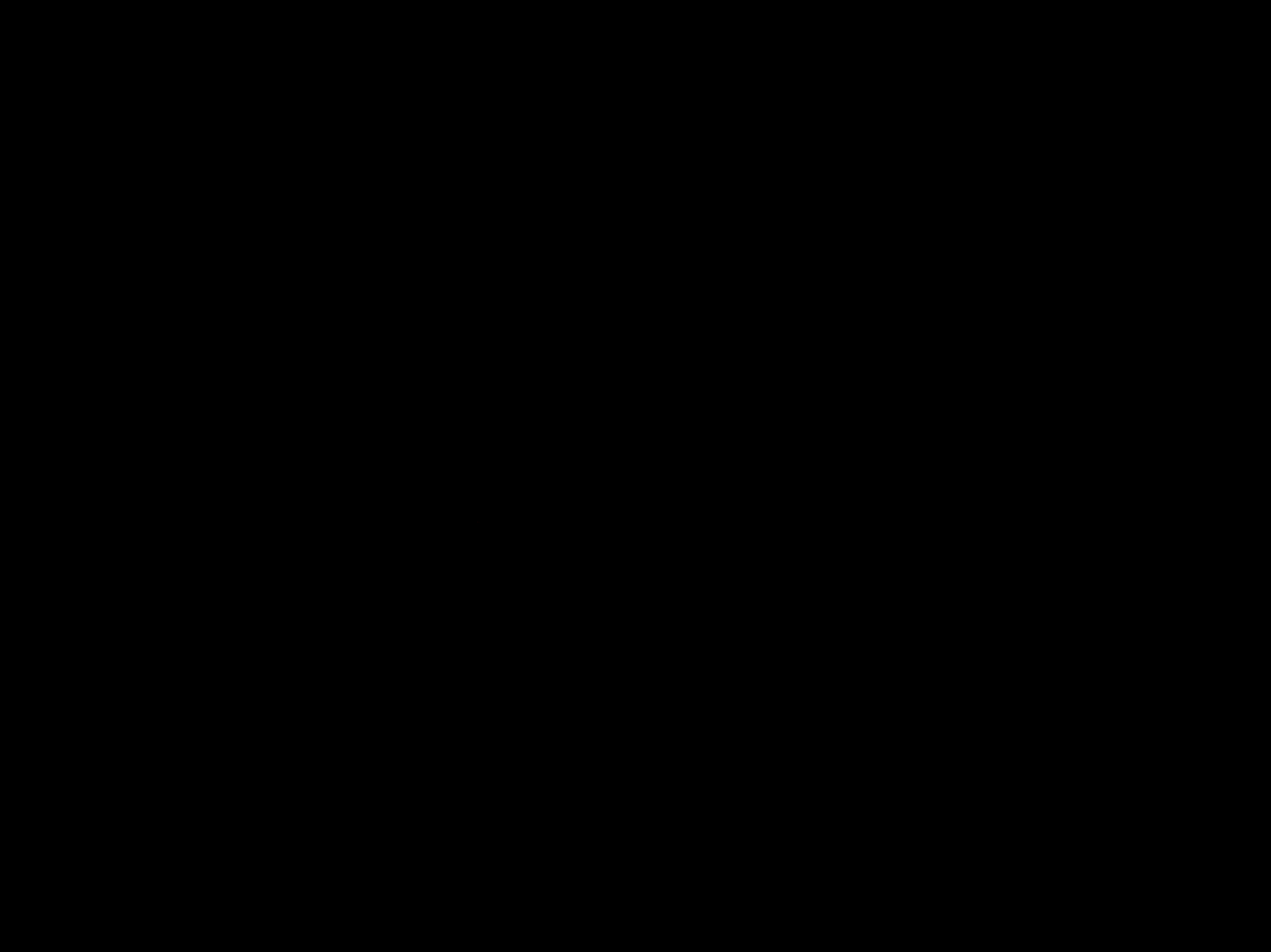

Supplement: Supplementary file 8 — Source Data for Appendix [file EMMM-15-e17815-s004.zip › Appendix_FigS1D/30min/col-10-30min.tif]

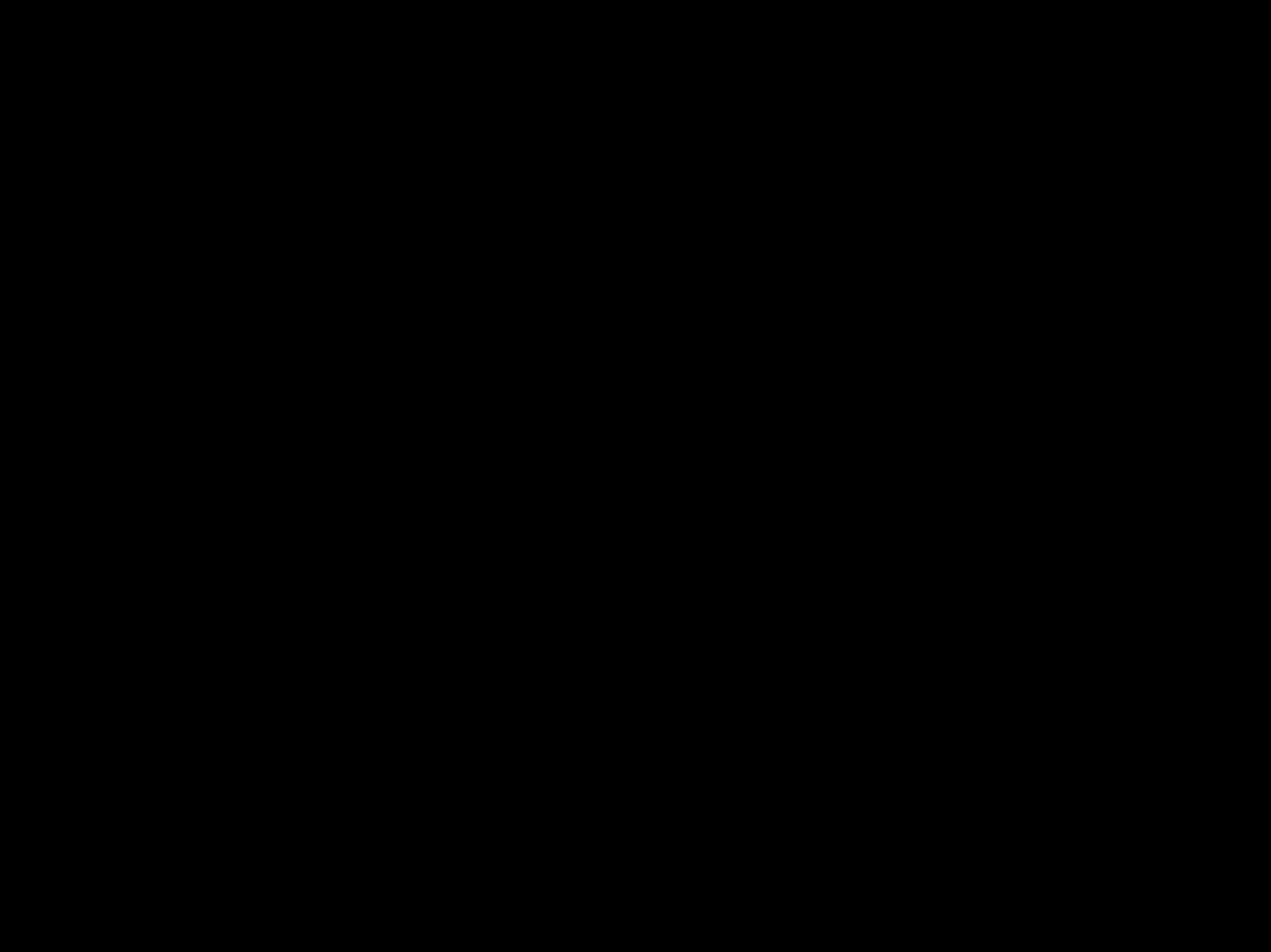

Supplement: Supplementary file 8 — Source Data for Appendix [file EMMM-15-e17815-s004.zip › Appendix_FigS1D/30min/ctr-30min.tif]

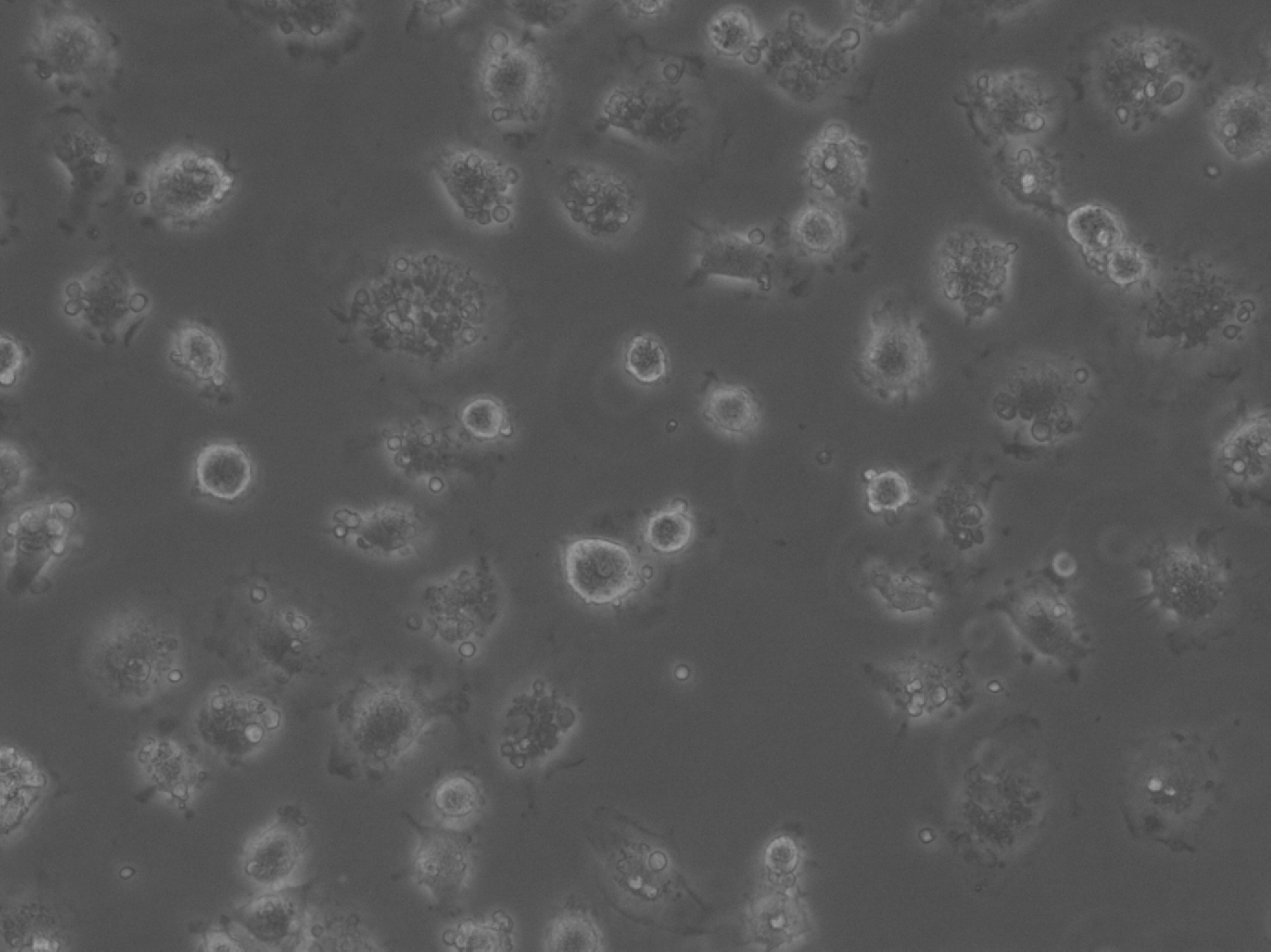

Supplement: Supplementary file 9 — Source Data for Figure 1 [file EMMM-15-e17815-s002.zip › Figure_1/1C/COL-10_uM/bright-BMDM.tif]

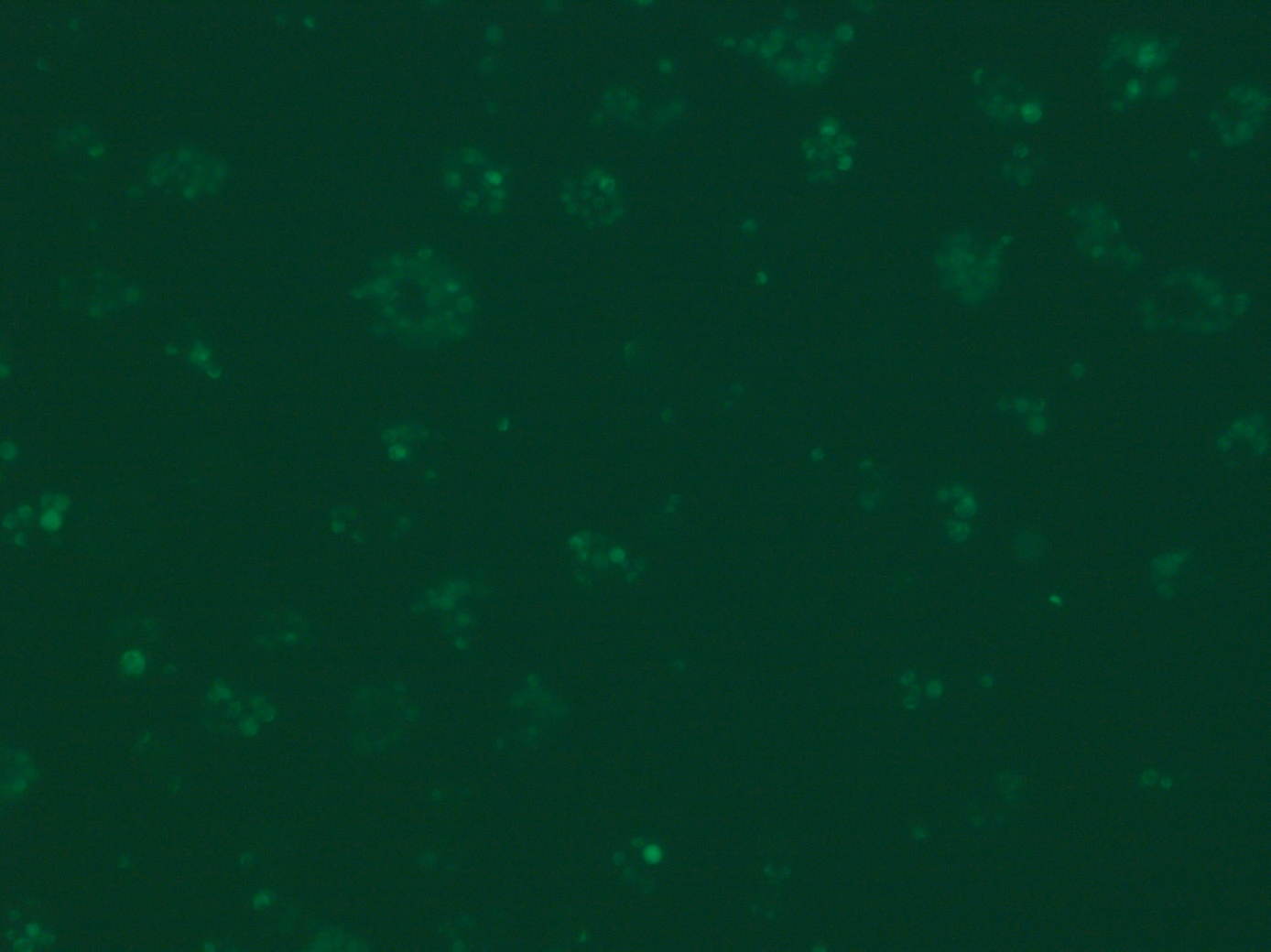

Supplement: Supplementary file 9 — Source Data for Figure 1 [file EMMM-15-e17815-s002.zip › Figure_1/1C/COL-10_uM/Fluo._image-CMFDA-ACs.jpg]

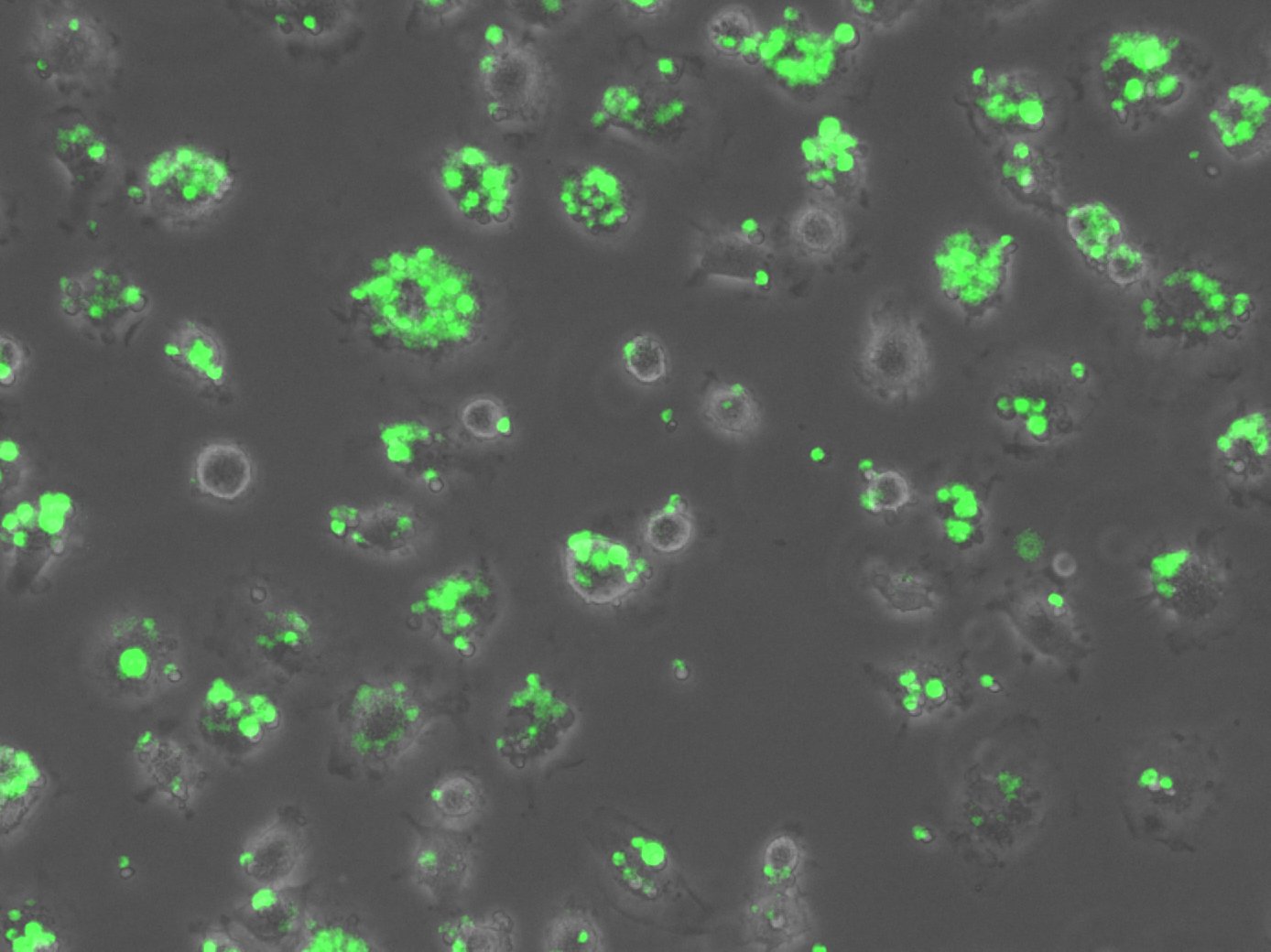

Supplement: Supplementary file 9 — Source Data for Figure 1 [file EMMM-15-e17815-s002.zip › Figure_1/1C/COL-10_uM/merged.jpg]

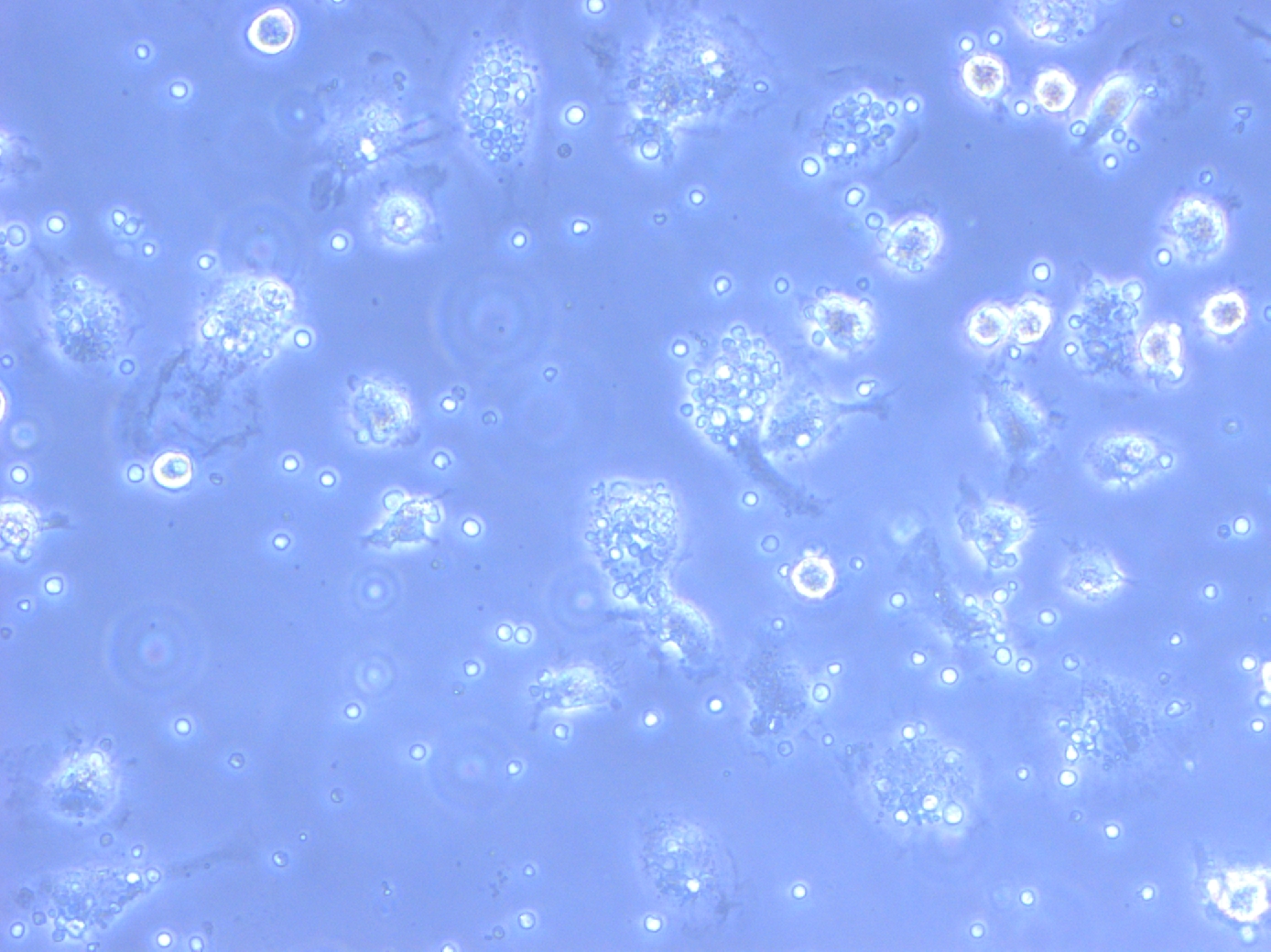

Supplement: Supplementary file 9 — Source Data for Figure 1 [file EMMM-15-e17815-s002.zip › Figure_1/1C/COL-20_uM/bright-BMDM.jpg]

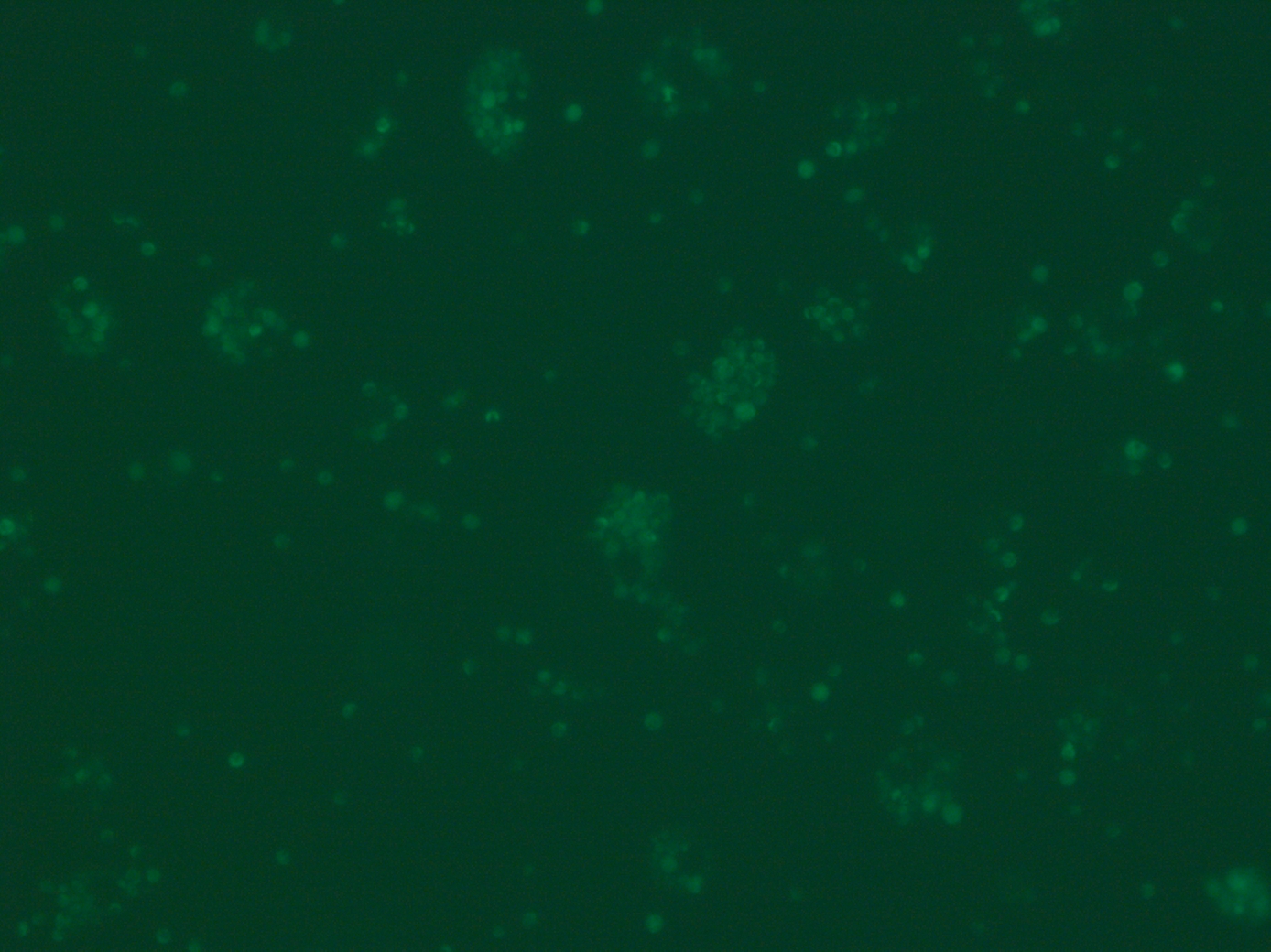

Supplement: Supplementary file 9 — Source Data for Figure 1 [file EMMM-15-e17815-s002.zip › Figure_1/1C/COL-20_uM/Fluo._image-CMFDA-ACs.jpg]

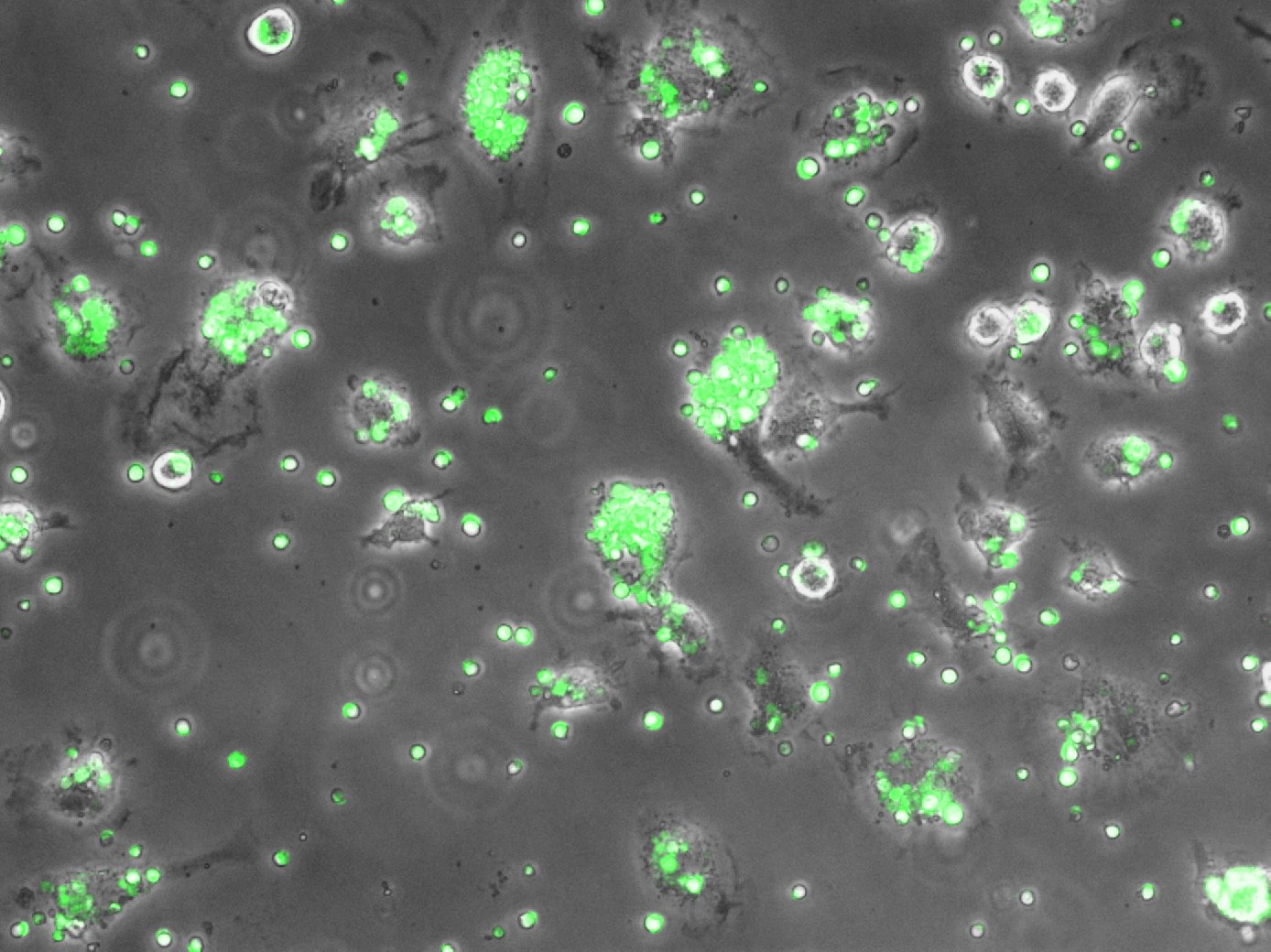

Supplement: Supplementary file 9 — Source Data for Figure 1 [file EMMM-15-e17815-s002.zip › Figure_1/1C/COL-20_uM/merged.jpg]

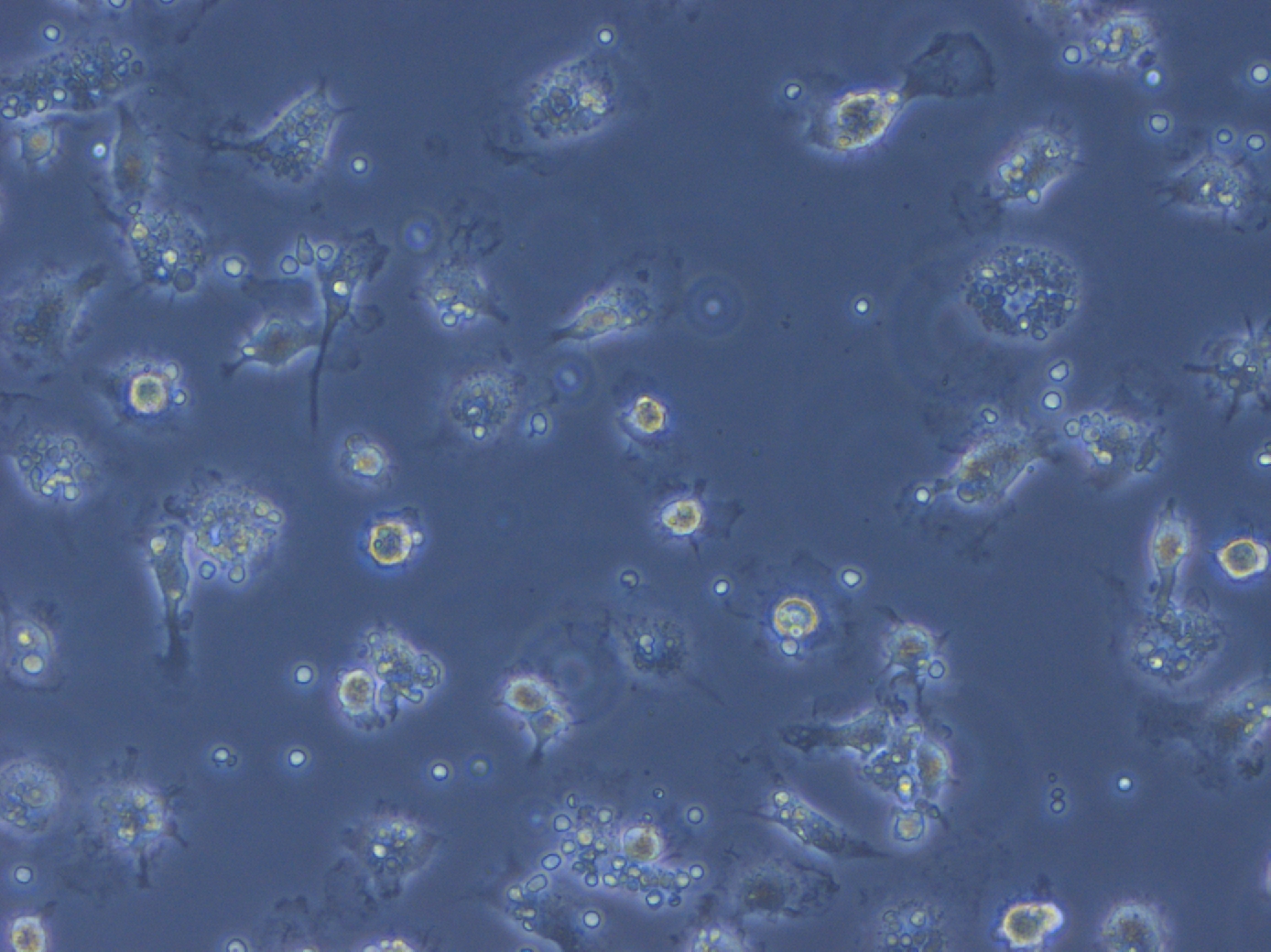

Supplement: Supplementary file 9 — Source Data for Figure 1 [file EMMM-15-e17815-s002.zip › Figure_1/1C/CTRL/bright-BMDM.jpg]

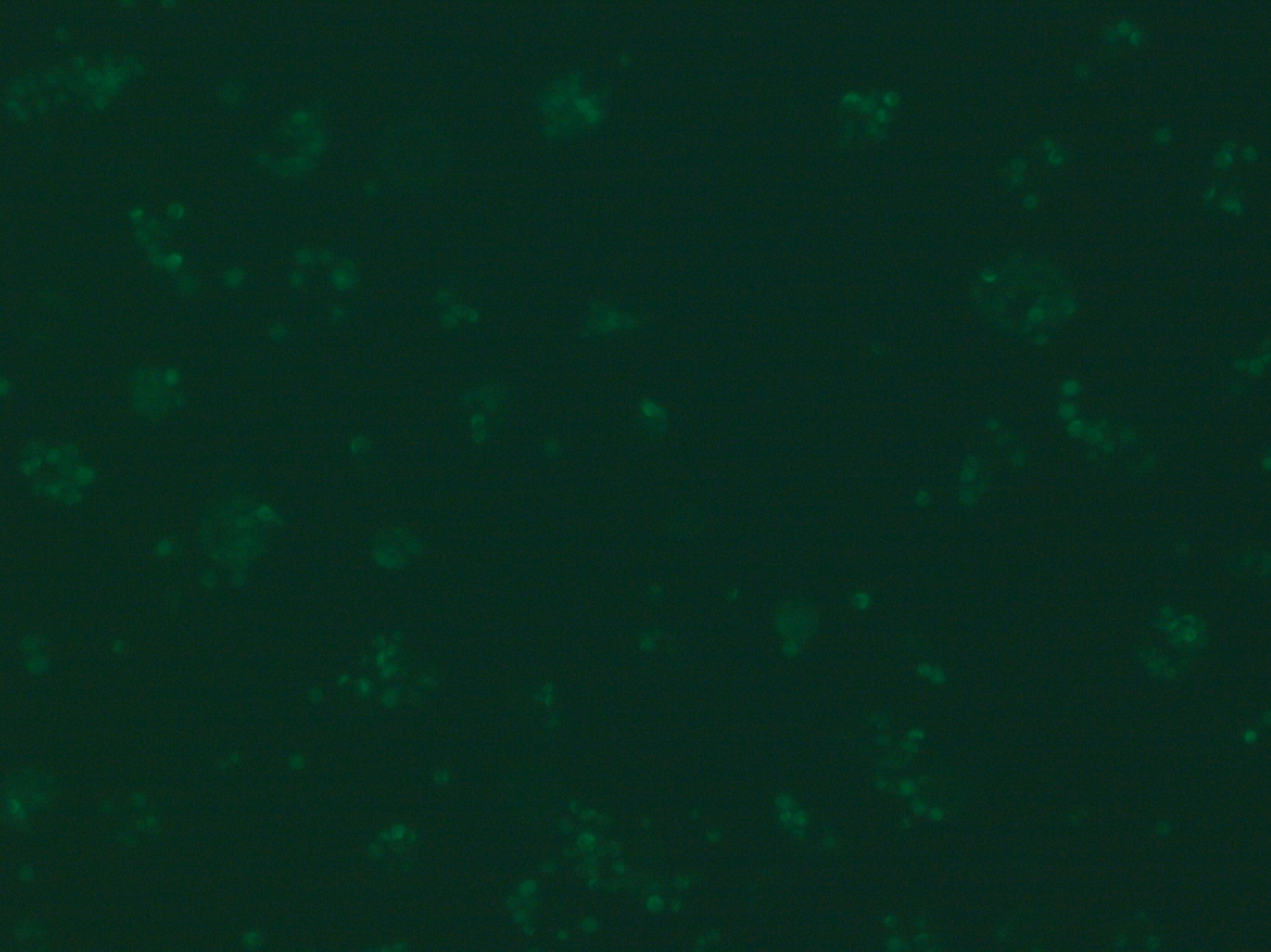

Supplement: Supplementary file 9 — Source Data for Figure 1 [file EMMM-15-e17815-s002.zip › Figure_1/1C/CTRL/Fluo._image-CMFDA-ACs.jpg]

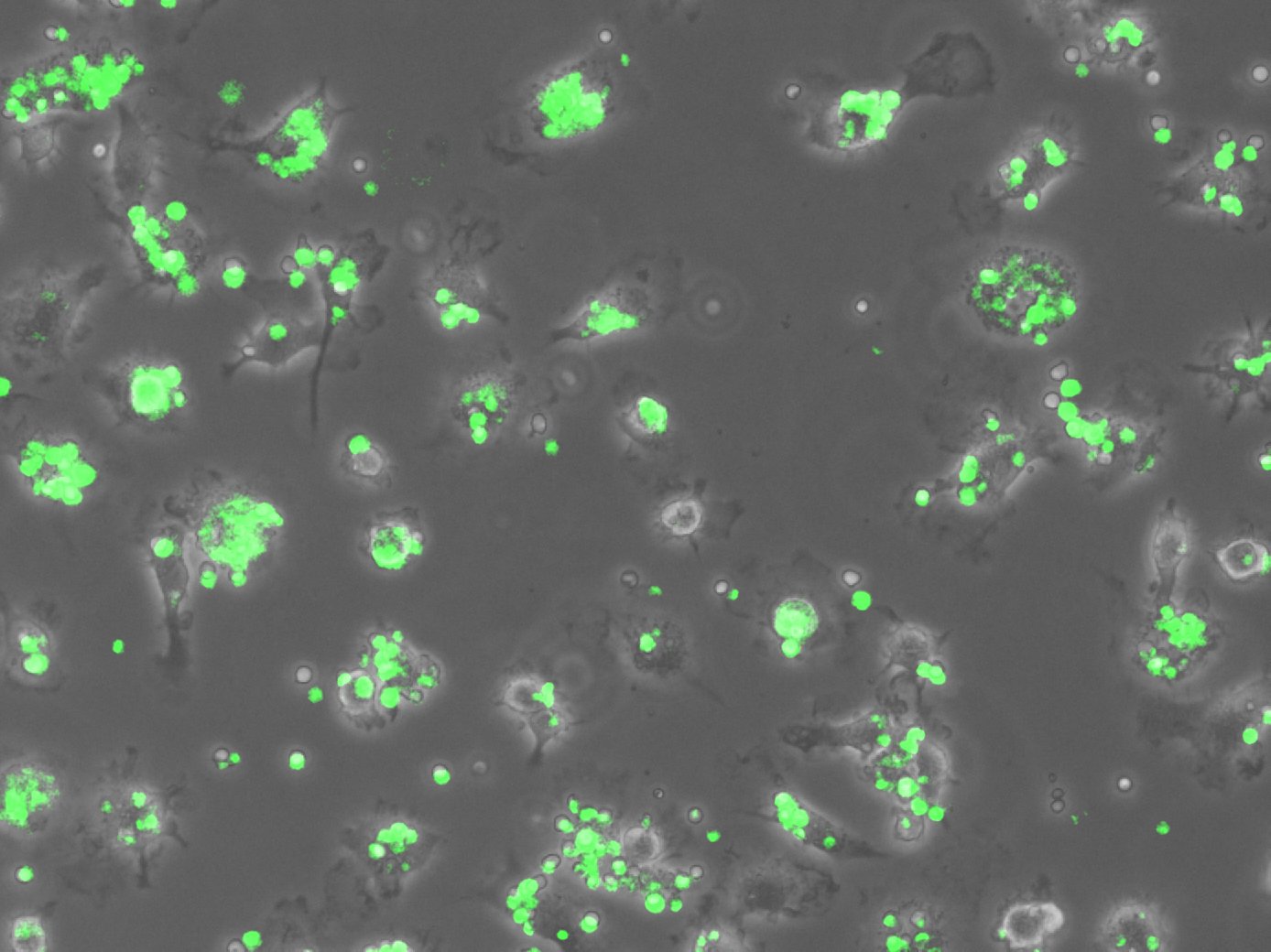

Supplement: Supplementary file 9 — Source Data for Figure 1 [file EMMM-15-e17815-s002.zip › Figure_1/1C/CTRL/Merge.jpg]

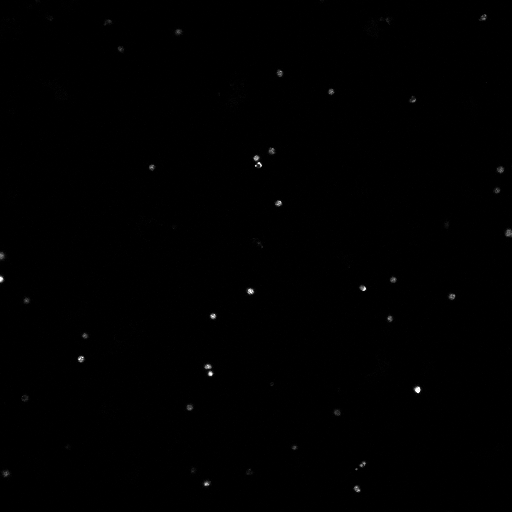

Supplement: Supplementary file 9 — Source Data for Figure 1 [file EMMM-15-e17815-s002.zip › Figure_1/1E/COL/Live_images_capture-efferocytosis/col_RAW_ch00.tif]

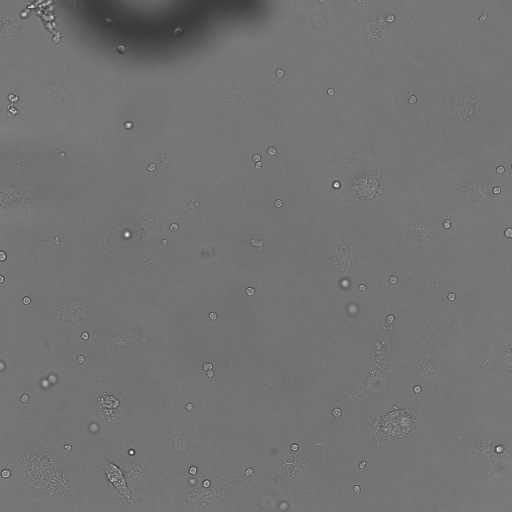

Supplement: Supplementary file 9 — Source Data for Figure 1 [file EMMM-15-e17815-s002.zip › Figure_1/1E/COL/Live_images_capture-efferocytosis/col_RAW_ch01.tif]

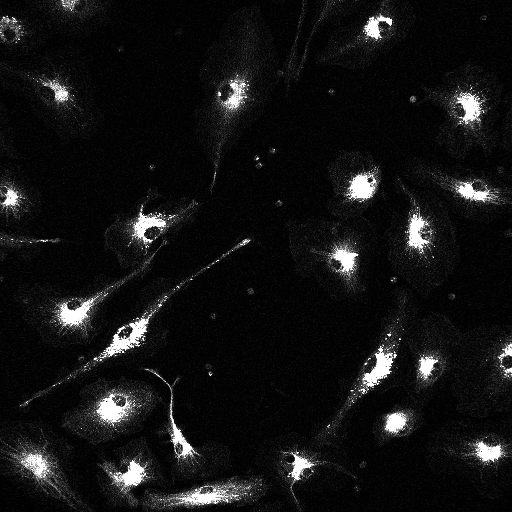

Supplement: Supplementary file 9 — Source Data for Figure 1 [file EMMM-15-e17815-s002.zip › Figure_1/1E/COL/Live_images_capture-efferocytosis/col_RAW_ch02.tif]

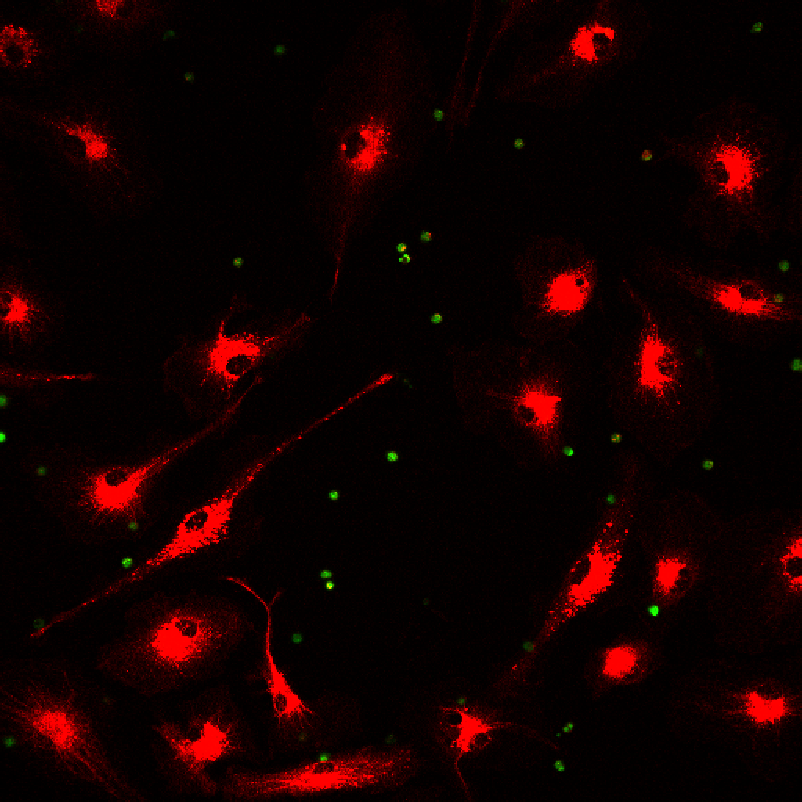

Supplement: Supplementary file 9 — Source Data for Figure 1 [file EMMM-15-e17815-s002.zip › Figure_1/1E/COL/Selected_images-efferocytosis/colSnapshot1-0_RAW_ch00.tif]

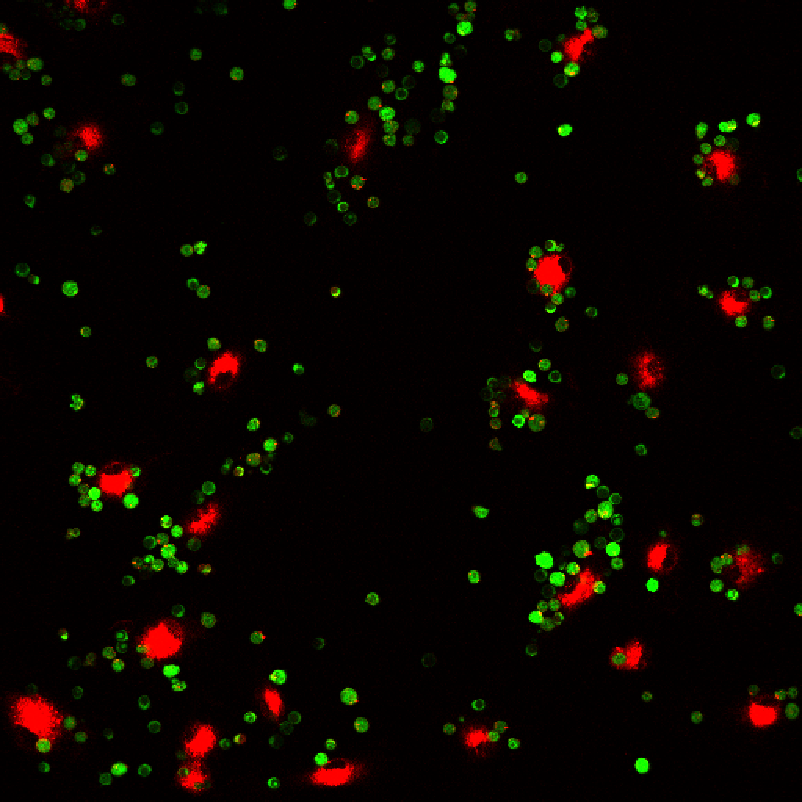

Supplement: Supplementary file 9 — Source Data for Figure 1 [file EMMM-15-e17815-s002.zip › Figure_1/1E/COL/Selected_images-efferocytosis/colSnapshot2-30_RAW_ch00.tif]

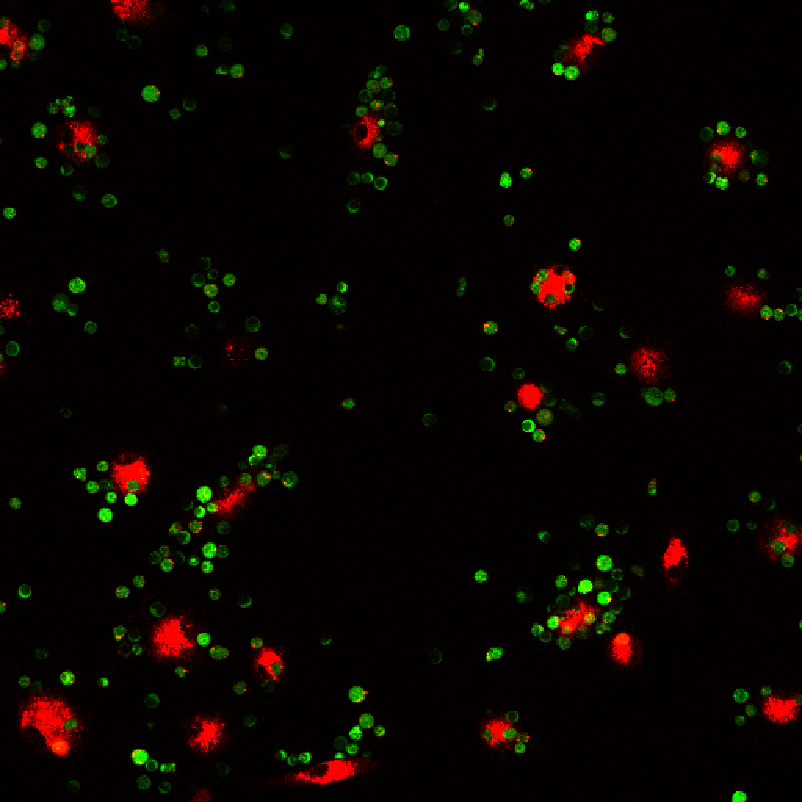

Supplement: Supplementary file 9 — Source Data for Figure 1 [file EMMM-15-e17815-s002.zip › Figure_1/1E/COL/Selected_images-efferocytosis/colSnapshot3-60_RAW_ch00.tif]

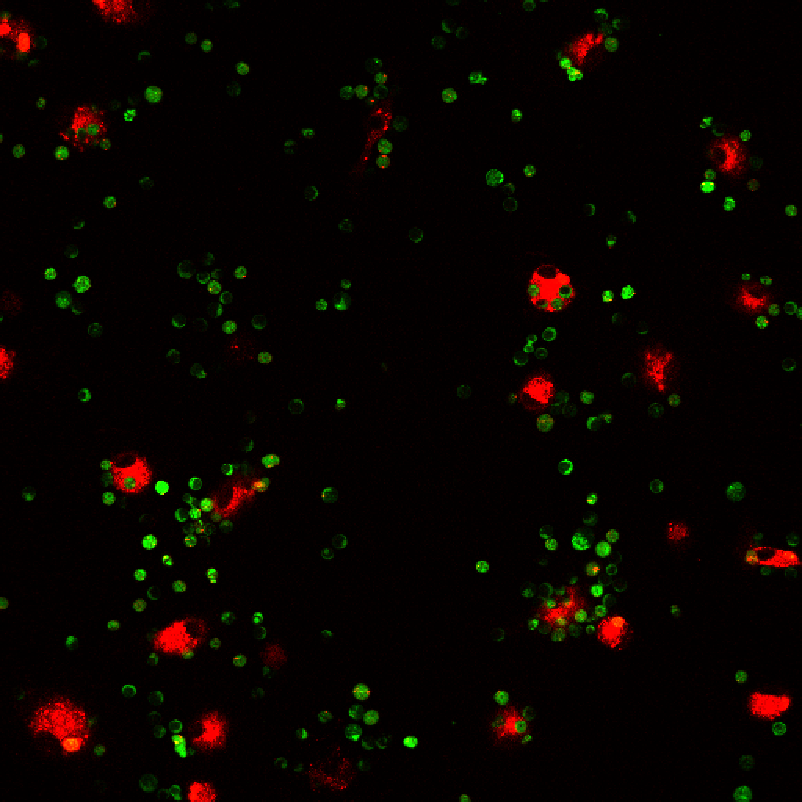

Supplement: Supplementary file 9 — Source Data for Figure 1 [file EMMM-15-e17815-s002.zip › Figure_1/1E/COL/Selected_images-efferocytosis/colSnapshot4-90_RAW_ch00.tif]

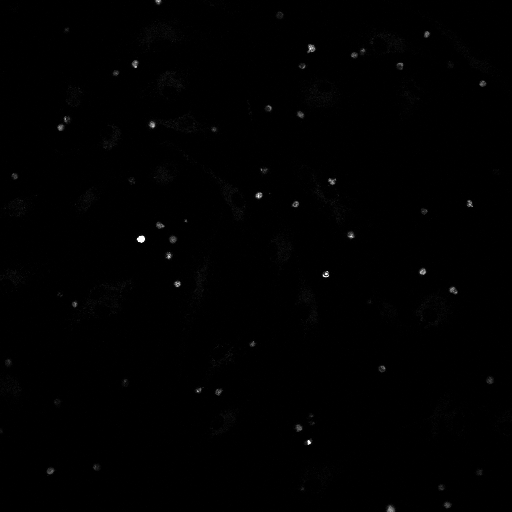

Supplement: Supplementary file 9 — Source Data for Figure 1 [file EMMM-15-e17815-s002.zip › Figure_1/1E/CTRL/Live_images_capture-efferocytosis/CTRL.lif_-_con_-_C=0.tif]

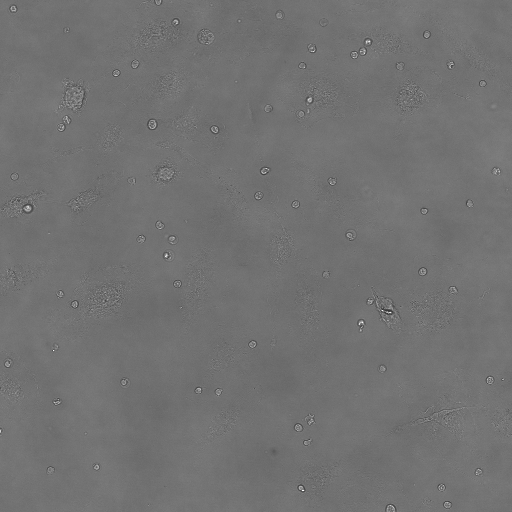

Supplement: Supplementary file 9 — Source Data for Figure 1 [file EMMM-15-e17815-s002.zip › Figure_1/1E/CTRL/Live_images_capture-efferocytosis/CTRL.lif_-_con_-_C=1.tif]

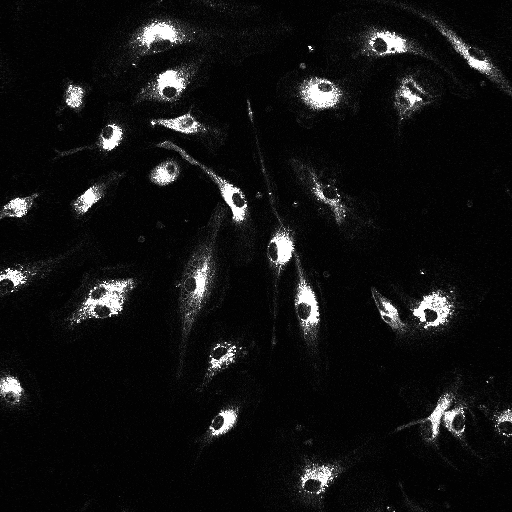

Supplement: Supplementary file 9 — Source Data for Figure 1 [file EMMM-15-e17815-s002.zip › Figure_1/1E/CTRL/Live_images_capture-efferocytosis/CTRL.lif_-_con_-_C=2.tif]

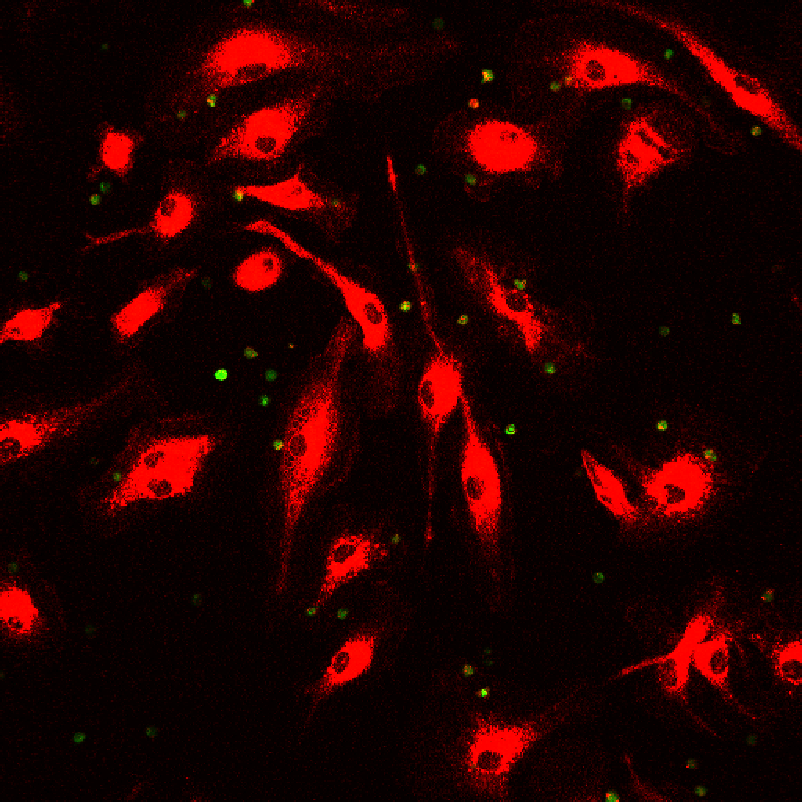

Supplement: Supplementary file 9 — Source Data for Figure 1 [file EMMM-15-e17815-s002.zip › Figure_1/1E/CTRL/Selected_images-efferocytosis/conSnapshot-0_RAW_ch00.tif]

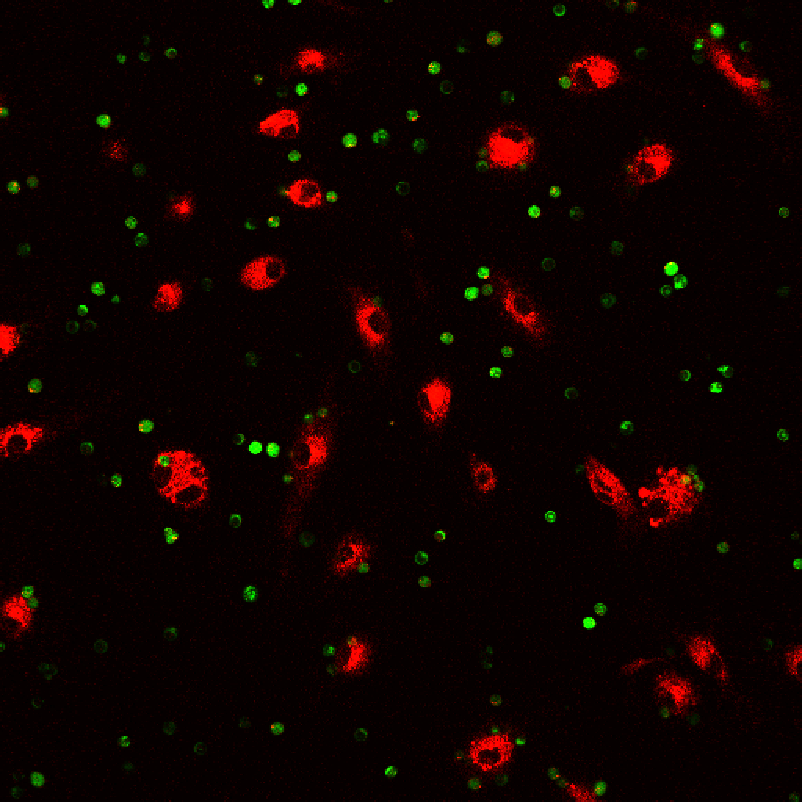

Supplement: Supplementary file 9 — Source Data for Figure 1 [file EMMM-15-e17815-s002.zip › Figure_1/1E/CTRL/Selected_images-efferocytosis/conSnapshot-30_RAW_ch00.tif]

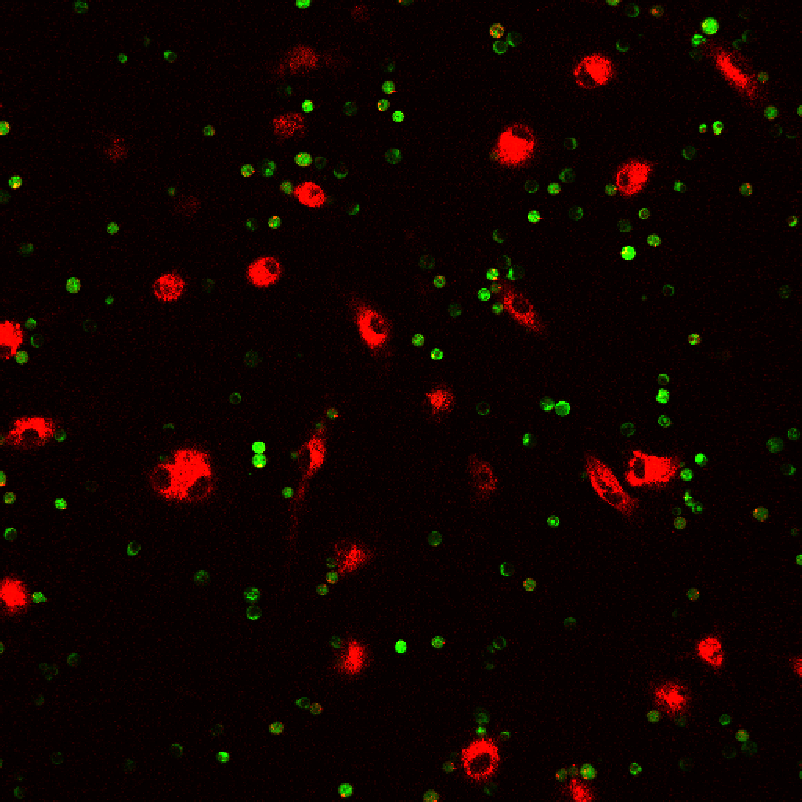

Supplement: Supplementary file 9 — Source Data for Figure 1 [file EMMM-15-e17815-s002.zip › Figure_1/1E/CTRL/Selected_images-efferocytosis/conSnapshot-60_RAW_ch00.tif]

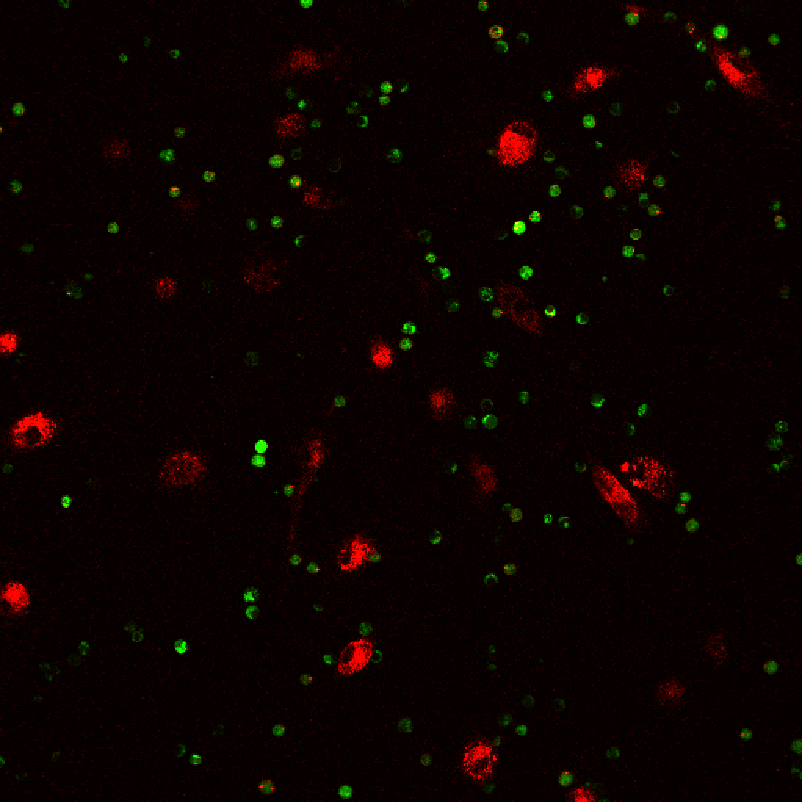

Supplement: Supplementary file 9 — Source Data for Figure 1 [file EMMM-15-e17815-s002.zip › Figure_1/1E/CTRL/Selected_images-efferocytosis/conSnapshot-90_RAW_ch00.tif]

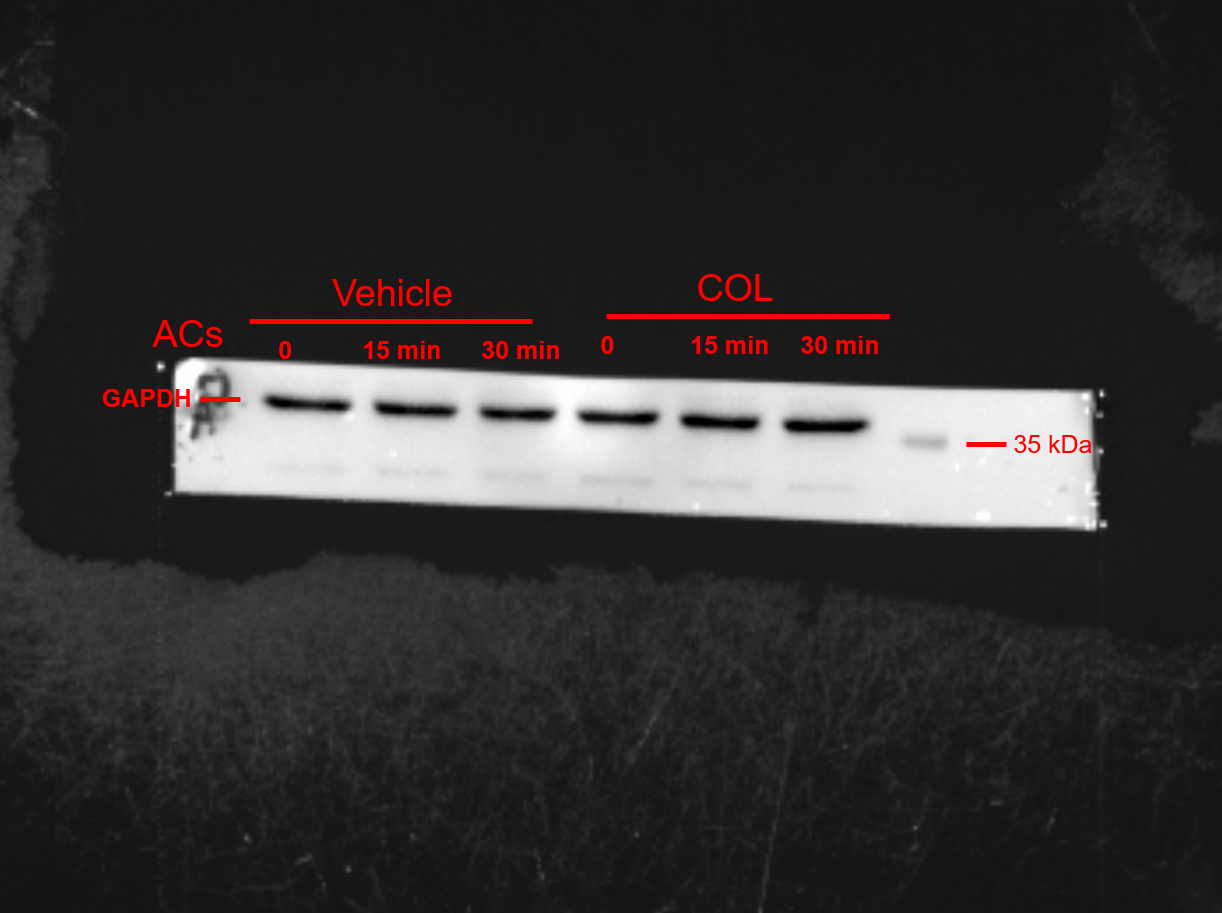

Supplement: Supplementary file 10 — Source Data for Figure 2 [file EMMM-15-e17815-s013.zip › Figure_2/2A/westernblot-gapdh.tif]

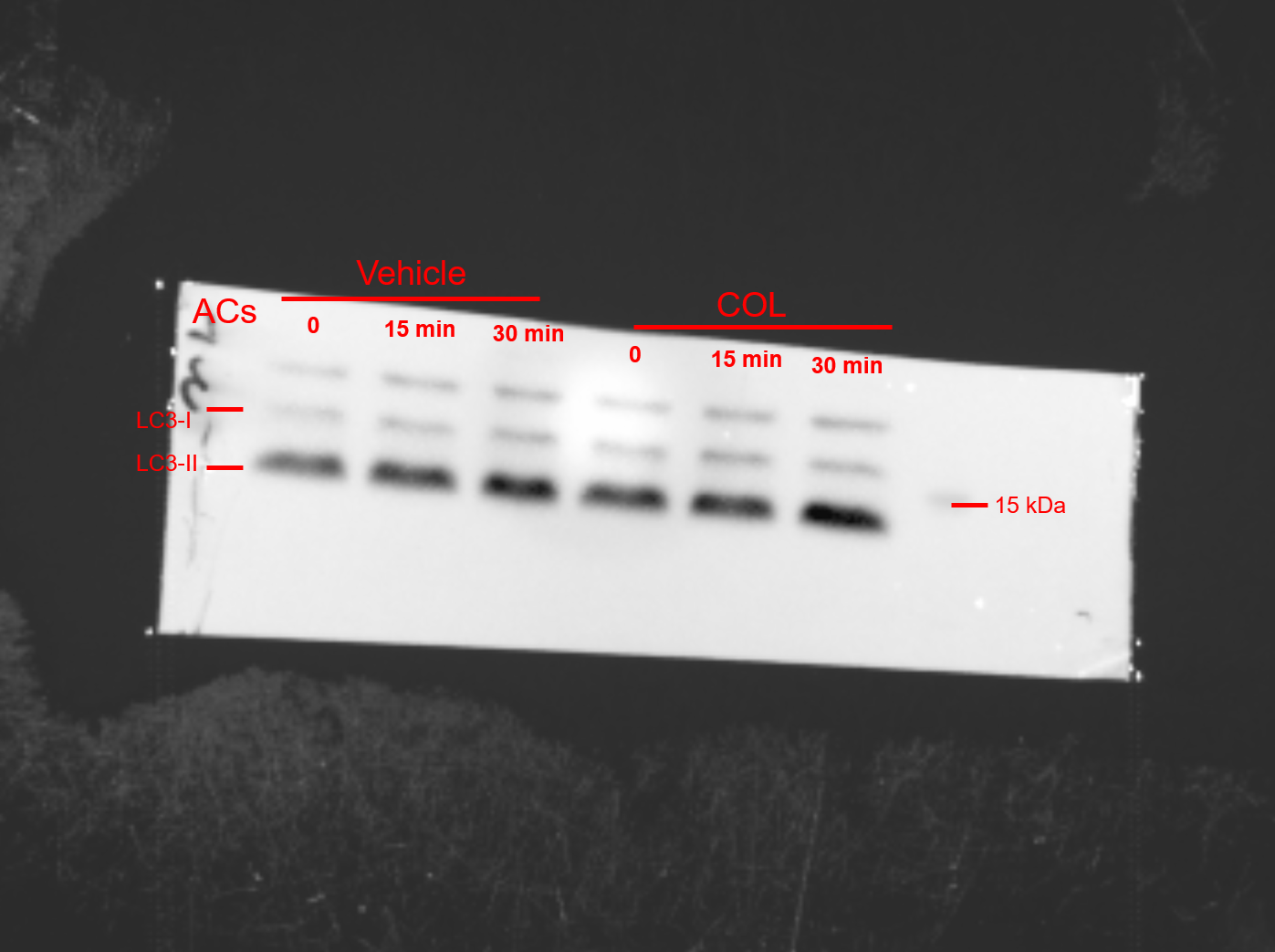

Supplement: Supplementary file 10 — Source Data for Figure 2 [file EMMM-15-e17815-s013.zip › Figure_2/2A/westernblot-LC3.tif]

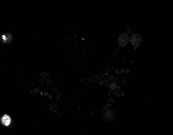

Supplement: Supplementary file 10 — Source Data for Figure 2 [file EMMM-15-e17815-s013.zip › Figure_2/2D/3-MA-COL/All_time_lapse_images/Fluo._Images-GFP-LC3-BMDMs_full_time.tif]

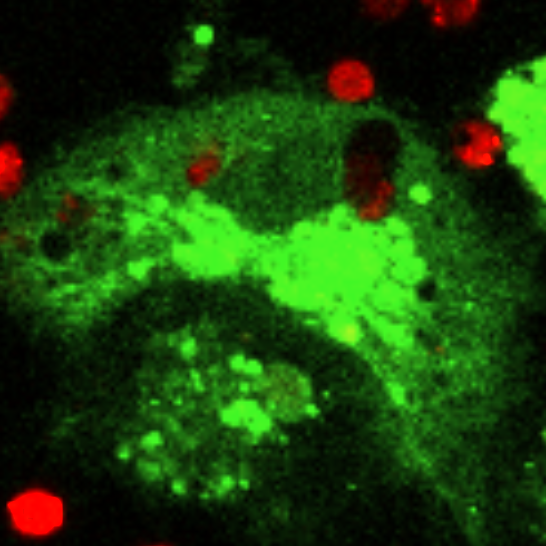

Supplement: Supplementary file 10 — Source Data for Figure 2 [file EMMM-15-e17815-s013.zip › Figure_2/2D/3-MA-COL/Images_used/Fluo._Images-GFP-LC3-BMDMs_0m.tif]

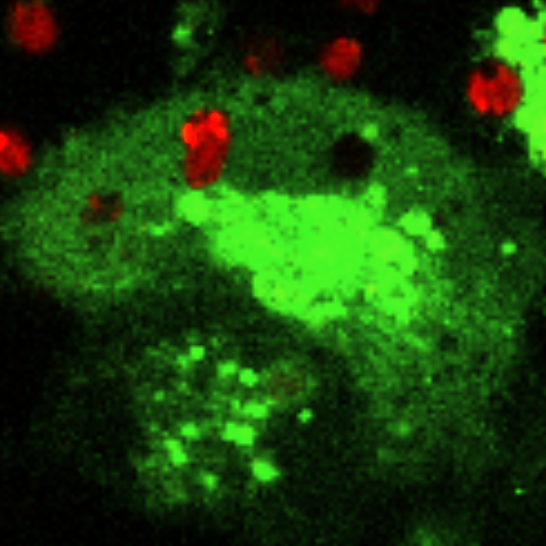

Supplement: Supplementary file 10 — Source Data for Figure 2 [file EMMM-15-e17815-s013.zip › Figure_2/2D/3-MA-COL/Images_used/Fluo._Images-GFP-LC3-BMDMs_30m.tif]

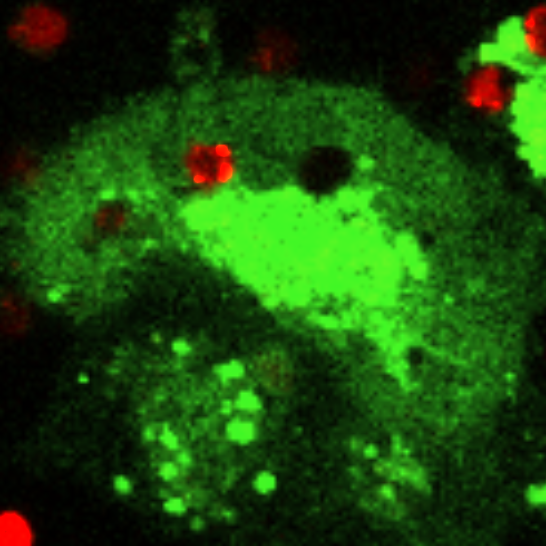

Supplement: Supplementary file 10 — Source Data for Figure 2 [file EMMM-15-e17815-s013.zip › Figure_2/2D/3-MA-COL/Images_used/Fluo._Images-GFP-LC3-BMDMs_60m.tif]

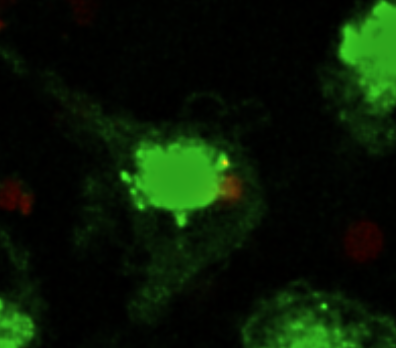

Supplement: Supplementary file 10 — Source Data for Figure 2 [file EMMM-15-e17815-s013.zip › Figure_2/2D/COL/Images_used/Fluo._Images-GFP-LC3-BMDMs_0m.tif]

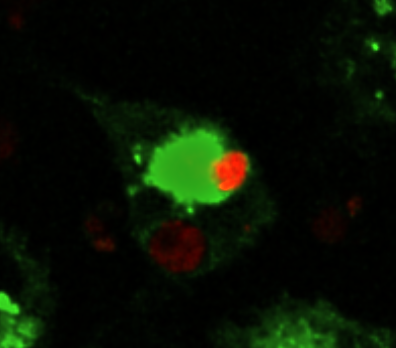

Supplement: Supplementary file 10 — Source Data for Figure 2 [file EMMM-15-e17815-s013.zip › Figure_2/2D/COL/Images_used/Fluo._Images-GFP-LC3-BMDMs_30m.tif]

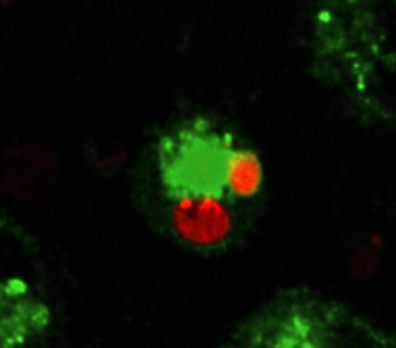

Supplement: Supplementary file 10 — Source Data for Figure 2 [file EMMM-15-e17815-s013.zip › Figure_2/2D/COL/Images_used/Fluo._Images-GFP-LC3-BMDMs_60m.tif]

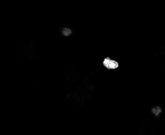

Supplement: Supplementary file 10 — Source Data for Figure 2 [file EMMM-15-e17815-s013.zip › Figure_2/2D/CTRL/All_time_lapse_images/Fluo._Images-GFP-LC3-BMDMs_full_time.tif]

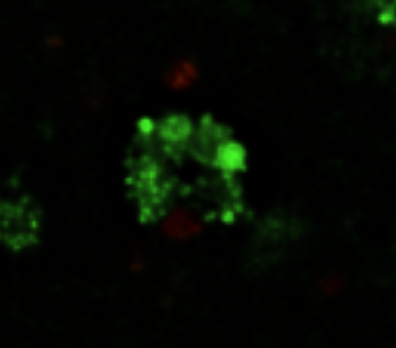

Supplement: Supplementary file 10 — Source Data for Figure 2 [file EMMM-15-e17815-s013.zip › Figure_2/2D/CTRL/Images_used/Fluo._Images-GFP-LC3-BMDMs_0m.tif]

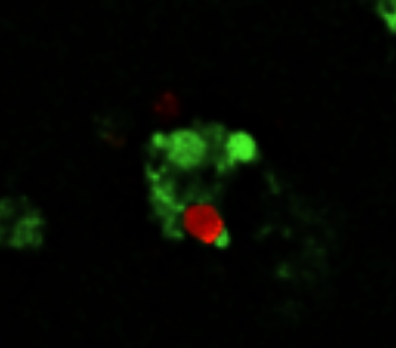

Supplement: Supplementary file 10 — Source Data for Figure 2 [file EMMM-15-e17815-s013.zip › Figure_2/2D/CTRL/Images_used/Fluo._Images-GFP-LC3-BMDMs_30m.tif]

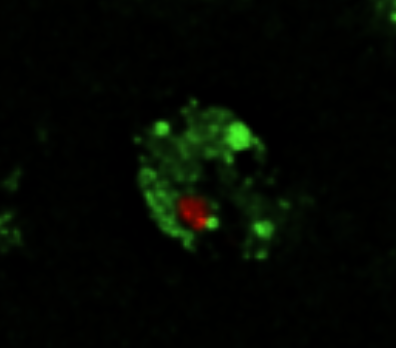

Supplement: Supplementary file 10 — Source Data for Figure 2 [file EMMM-15-e17815-s013.zip › Figure_2/2D/CTRL/Images_used/Fluo._Images-GFP-LC3-BMDMs_60m.tif]

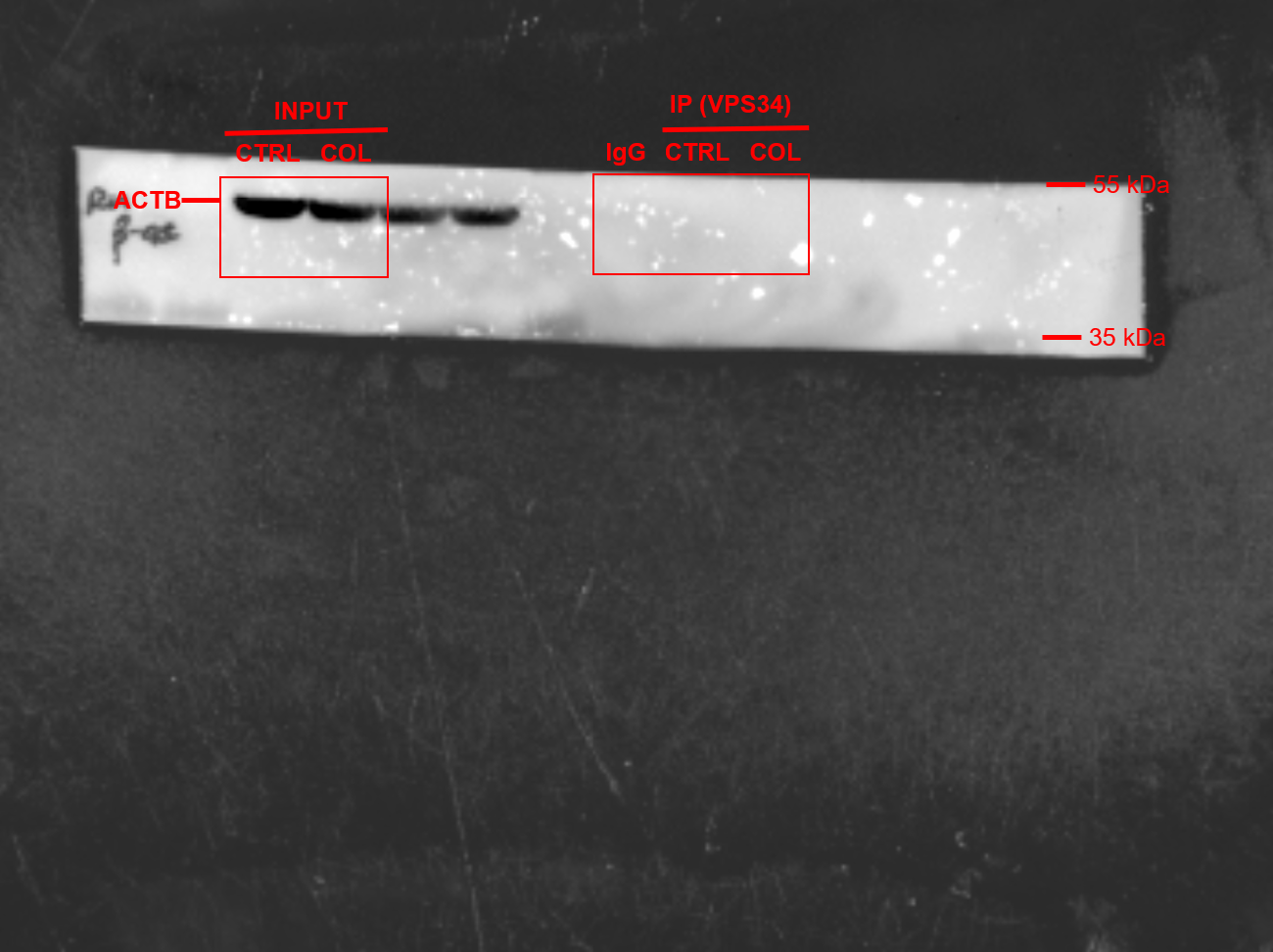

Supplement: Supplementary file 10 — Source Data for Figure 2 [file EMMM-15-e17815-s013.zip › Figure_2/2F/ACTB/westernblot-ACTIN.tif]

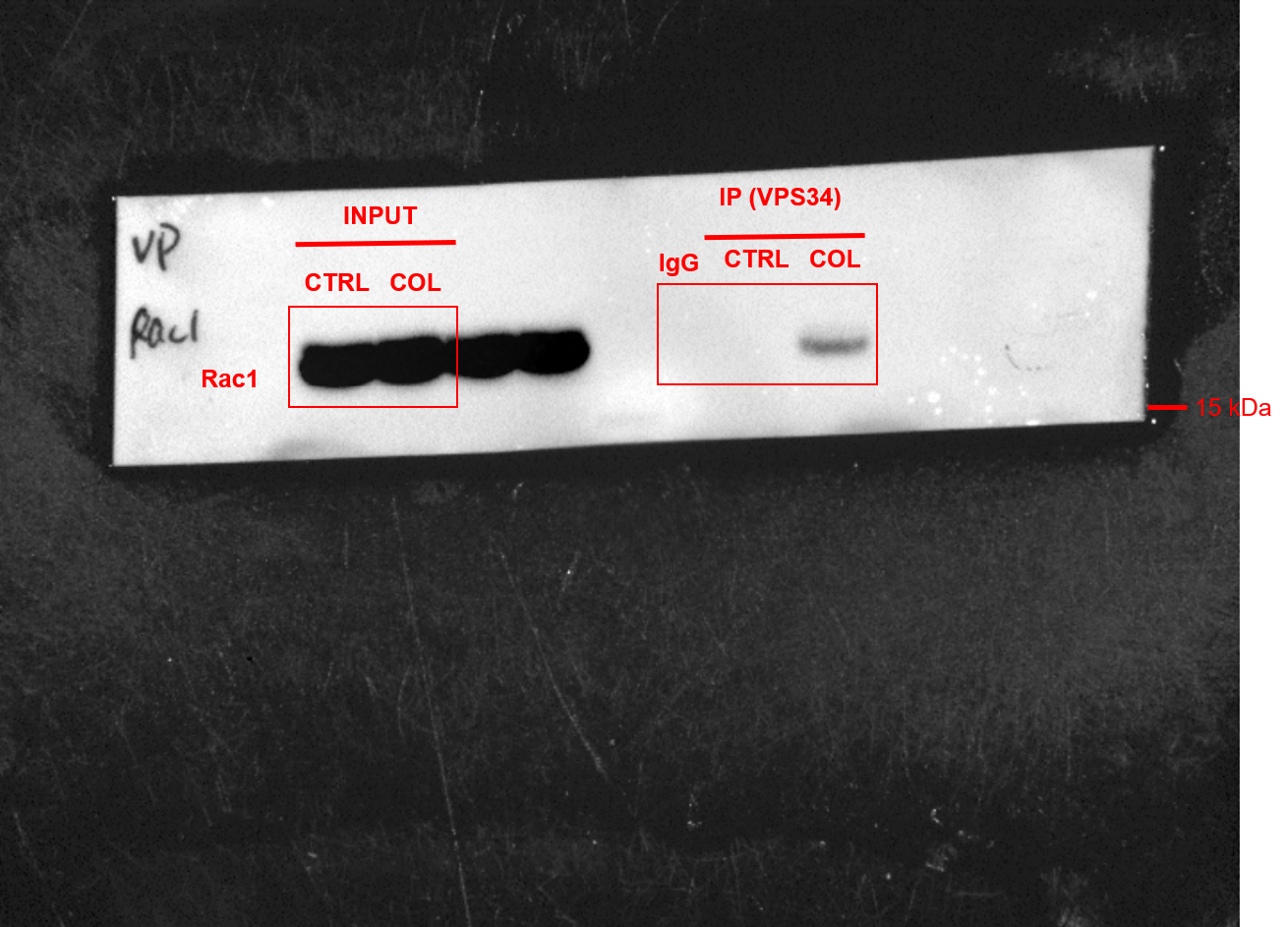

Supplement: Supplementary file 10 — Source Data for Figure 2 [file EMMM-15-e17815-s013.zip › Figure_2/2F/RAC1/Westernblot-RAC1_(long_time_expo.).tif]

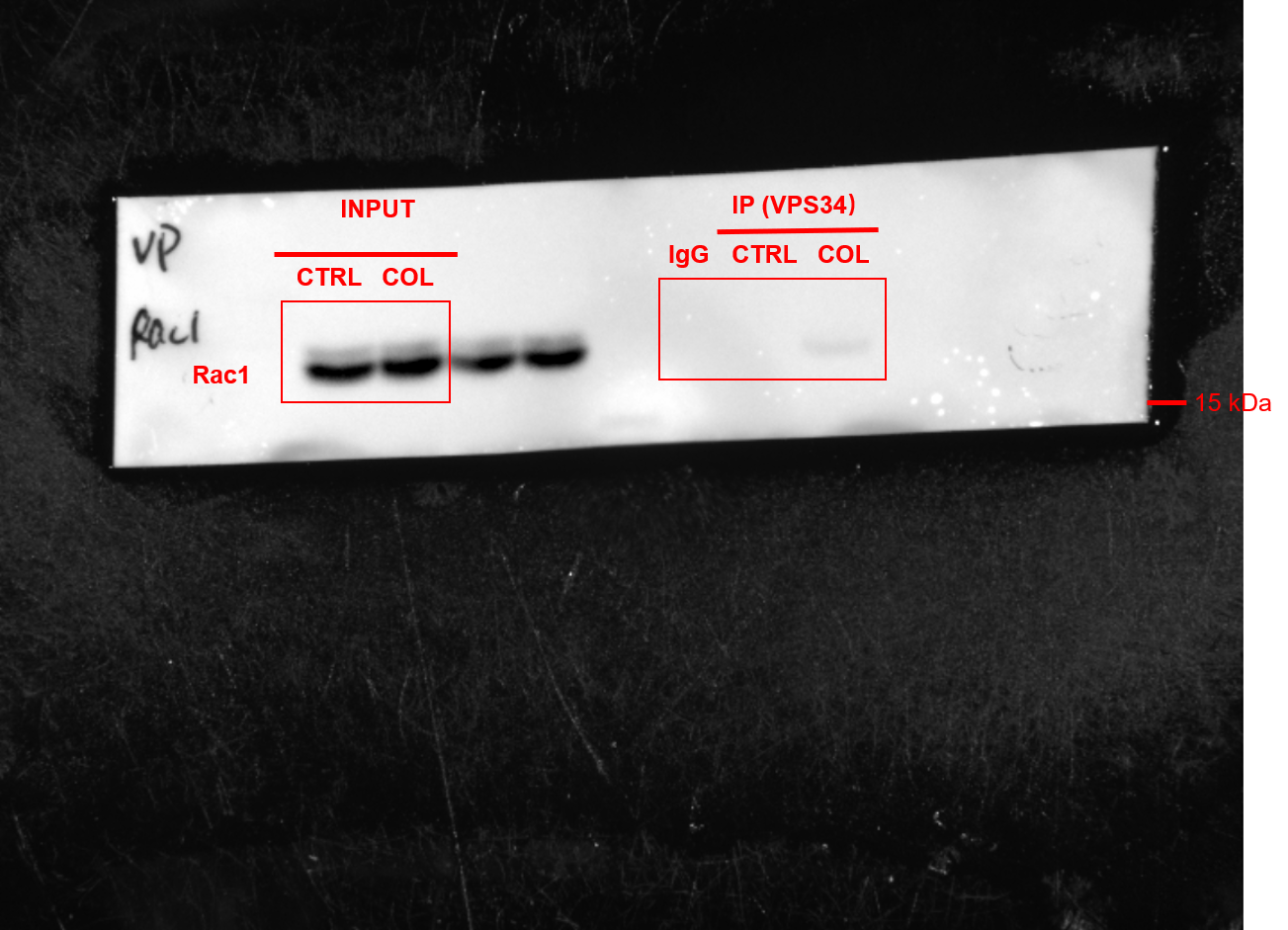

Supplement: Supplementary file 10 — Source Data for Figure 2 [file EMMM-15-e17815-s013.zip › Figure_2/2F/RAC1/Westernblot-RAC1_(short_time_expo.).tif]

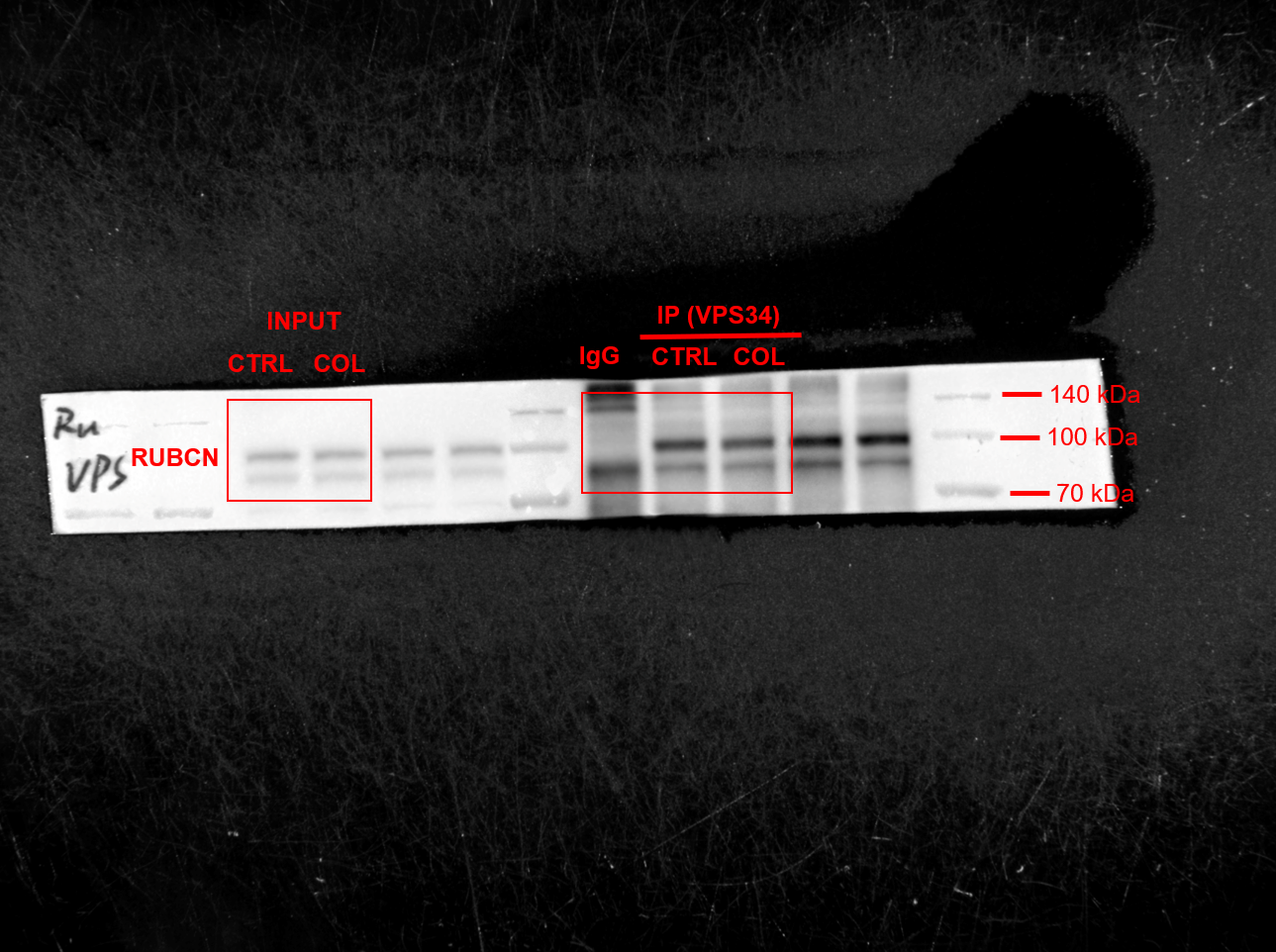

Supplement: Supplementary file 10 — Source Data for Figure 2 [file EMMM-15-e17815-s013.zip › Figure_2/2F/RUBICON/Westernblot-RUBICON.tif]

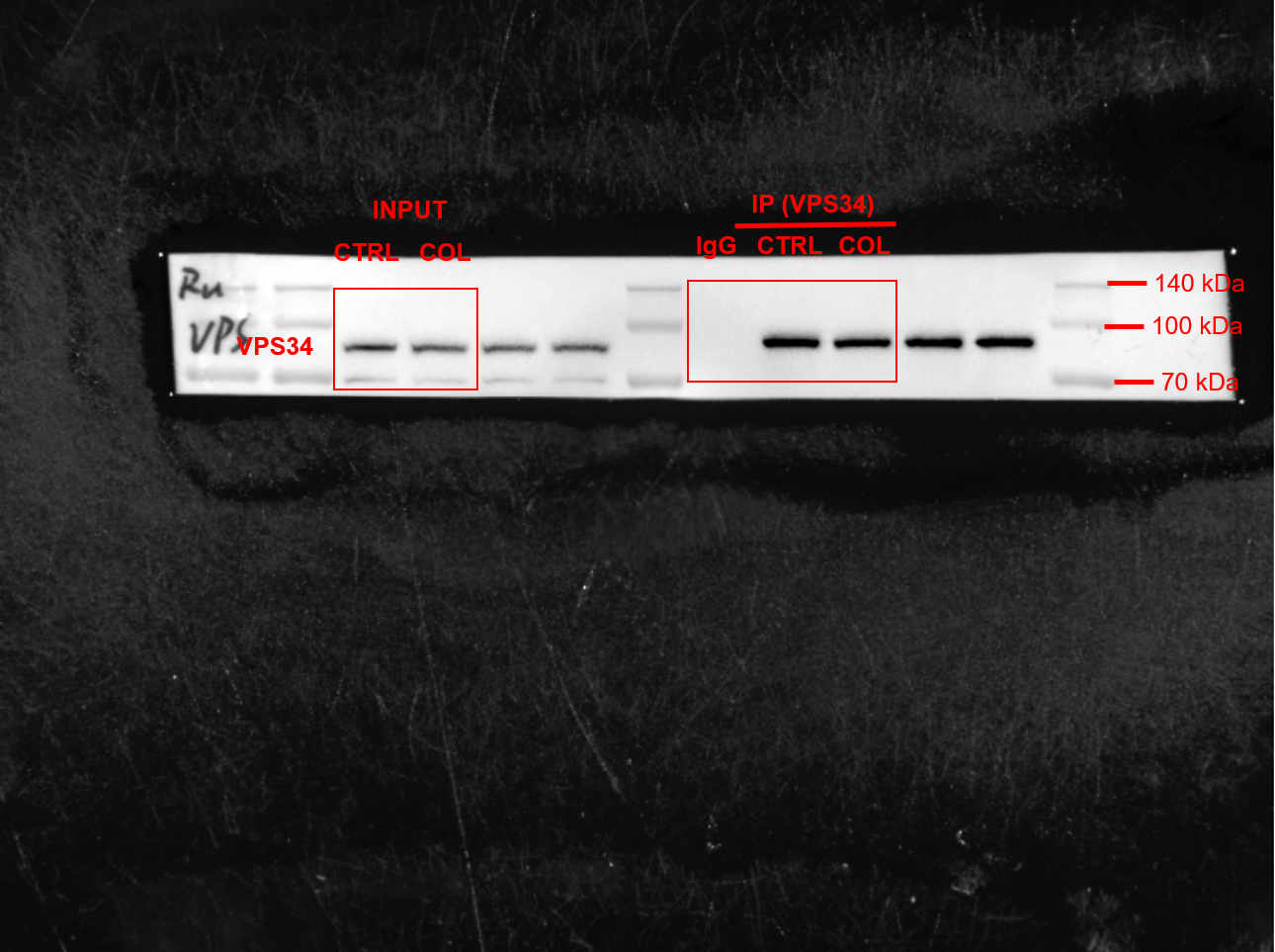

Supplement: Supplementary file 10 — Source Data for Figure 2 [file EMMM-15-e17815-s013.zip › Figure_2/2F/VPS34/Westernblot-VPS34.tif]

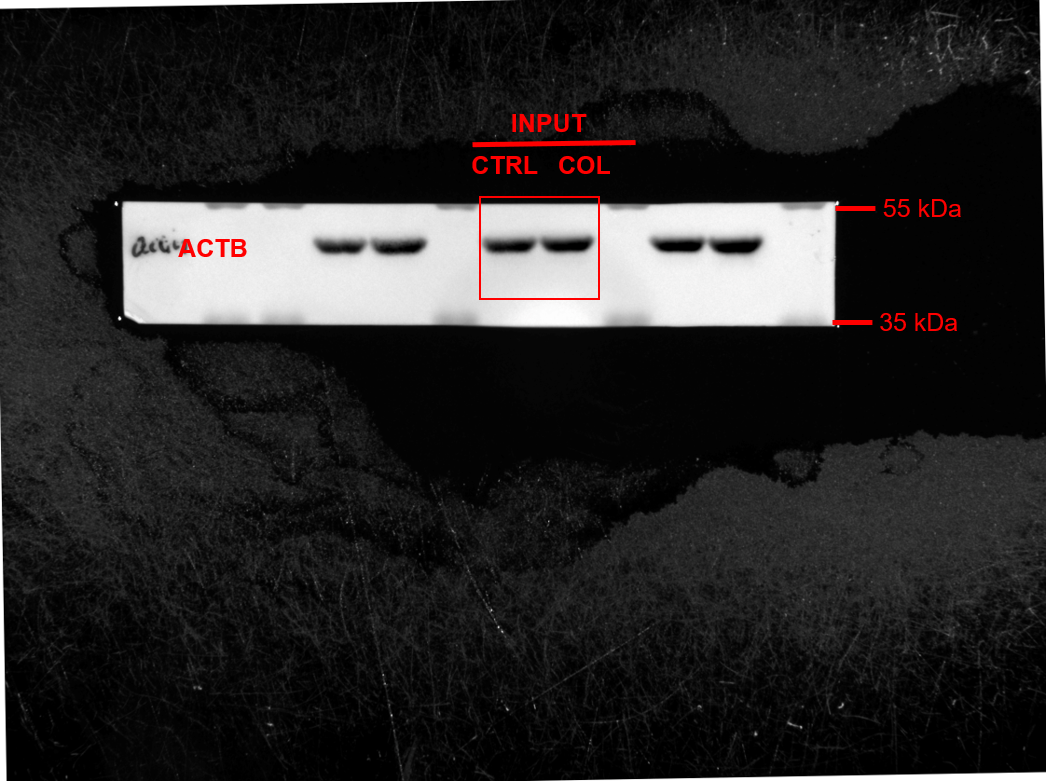

Supplement: Supplementary file 10 — Source Data for Figure 2 [file EMMM-15-e17815-s013.zip › Figure_2/2G/ACTB/Westernblot-ACTB-input.tif]

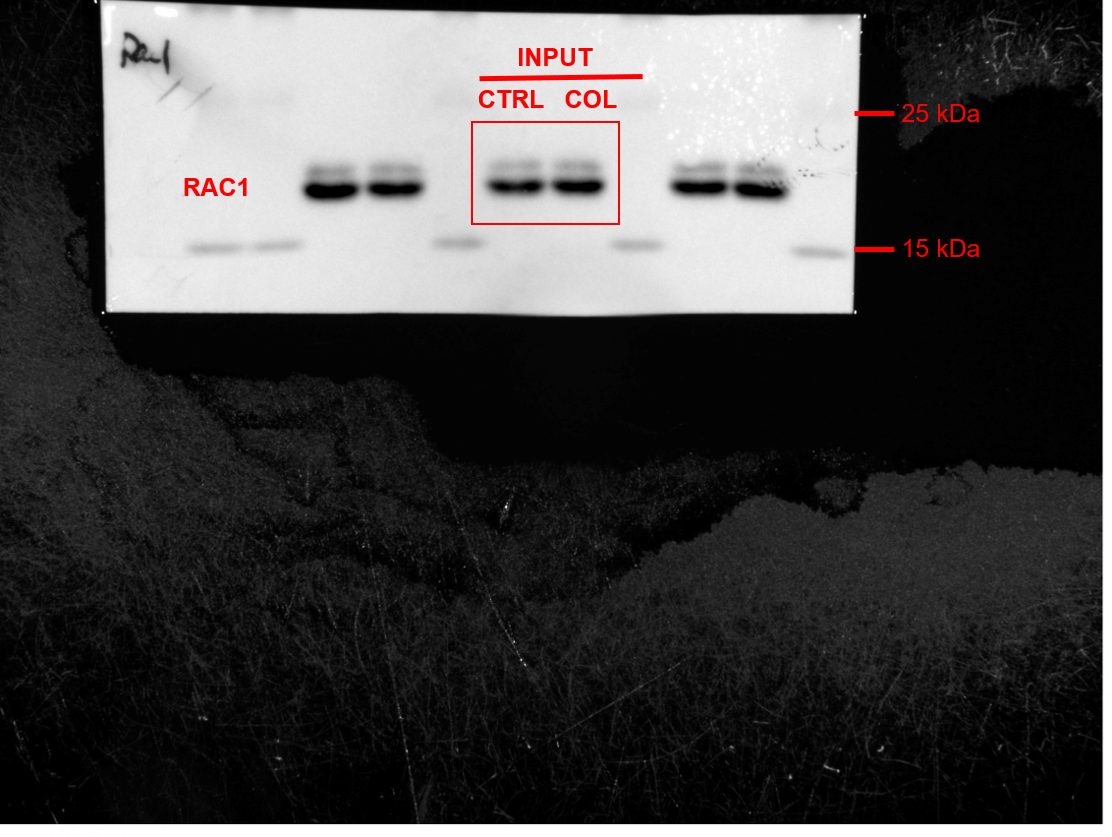

Supplement: Supplementary file 10 — Source Data for Figure 2 [file EMMM-15-e17815-s013.zip › Figure_2/2G/RAC1/Westernblot-RAC1_input.tif]

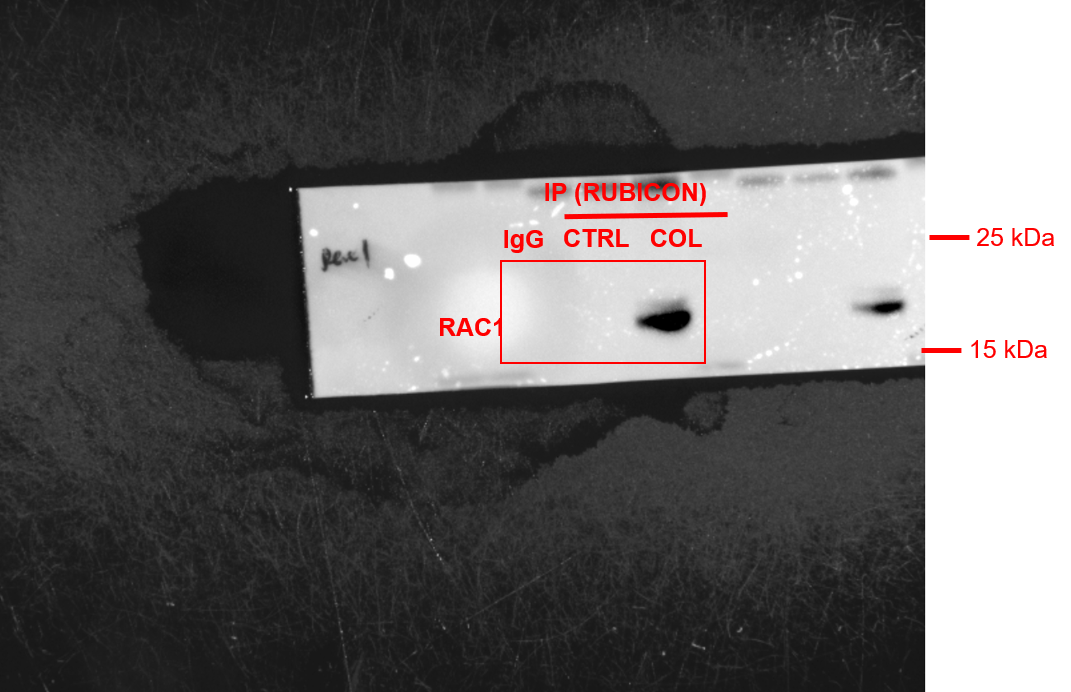

Supplement: Supplementary file 10 — Source Data for Figure 2 [file EMMM-15-e17815-s013.zip › Figure_2/2G/RAC1/Westernblot-RAC1_IP.tif]

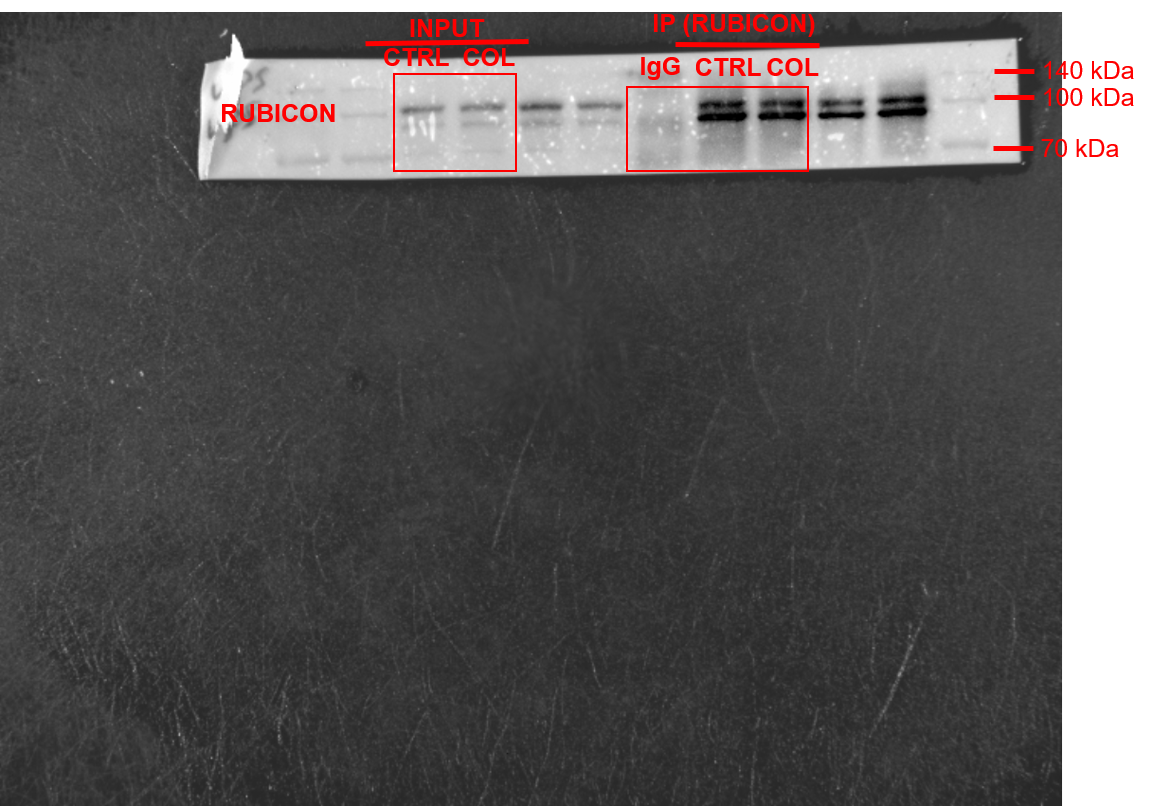

Supplement: Supplementary file 10 — Source Data for Figure 2 [file EMMM-15-e17815-s013.zip › Figure_2/2G/RUBICON/Westernblot-RUBICON.tif]

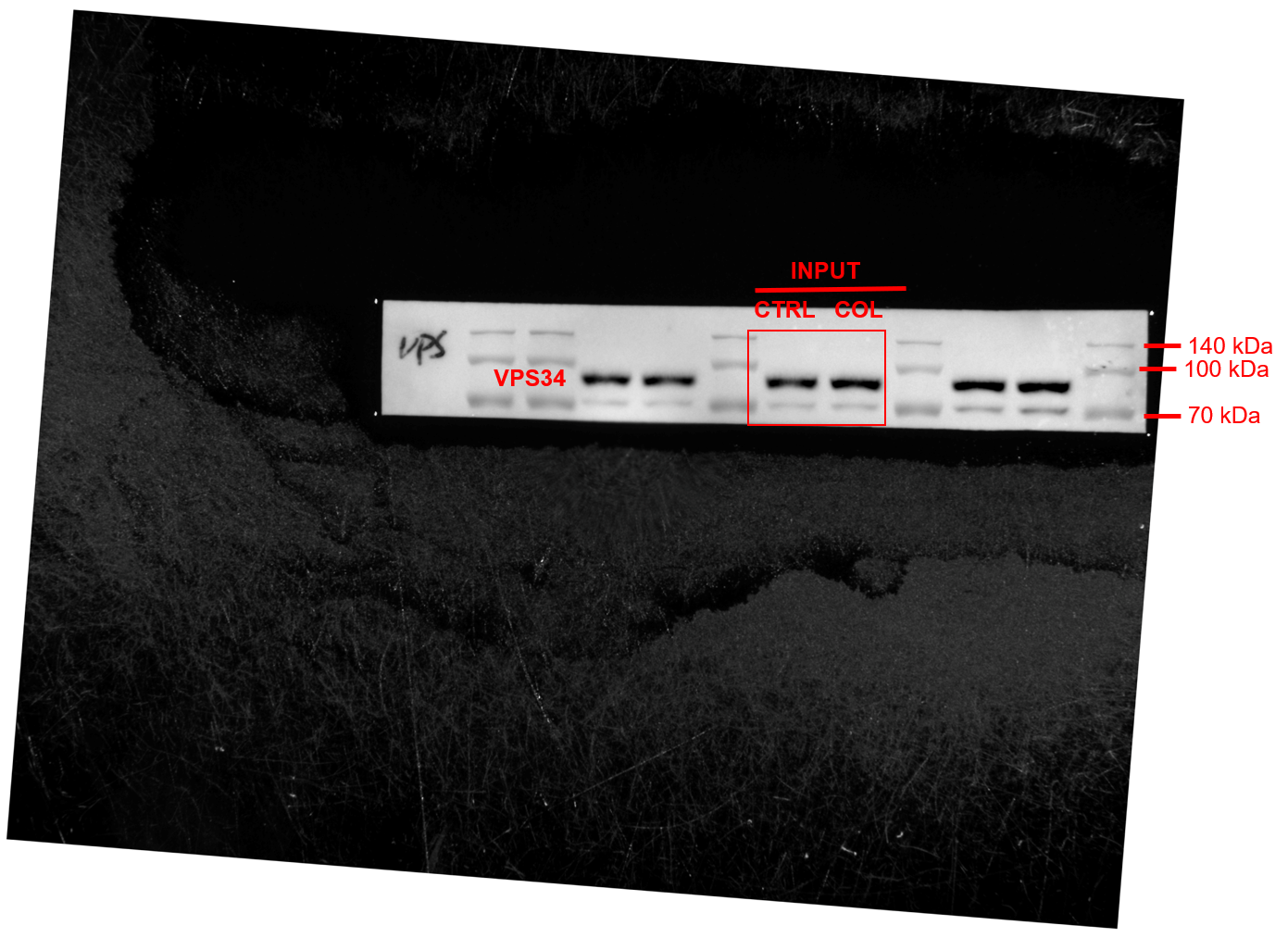

Supplement: Supplementary file 10 — Source Data for Figure 2 [file EMMM-15-e17815-s013.zip › Figure_2/2G/VPS34/Westernblot-vps34_input.tif]

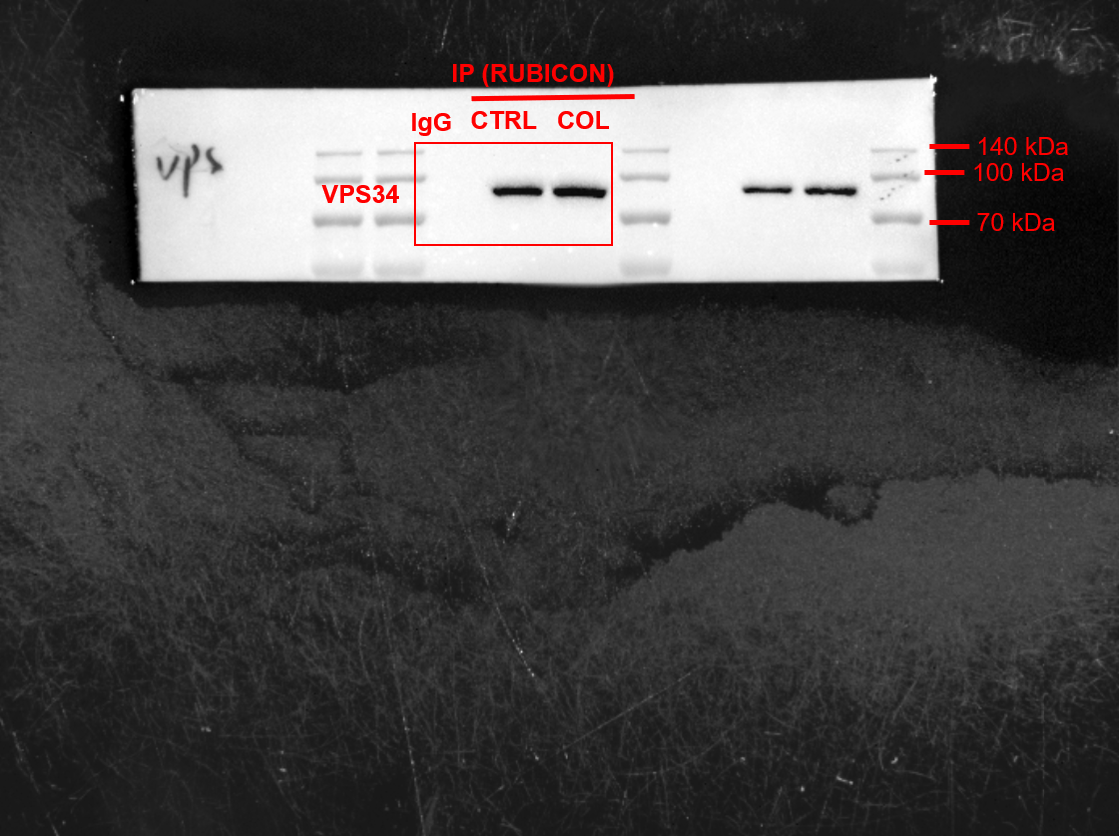

Supplement: Supplementary file 10 — Source Data for Figure 2 [file EMMM-15-e17815-s013.zip › Figure_2/2G/VPS34/Westernblot-vps34_IP.tif]

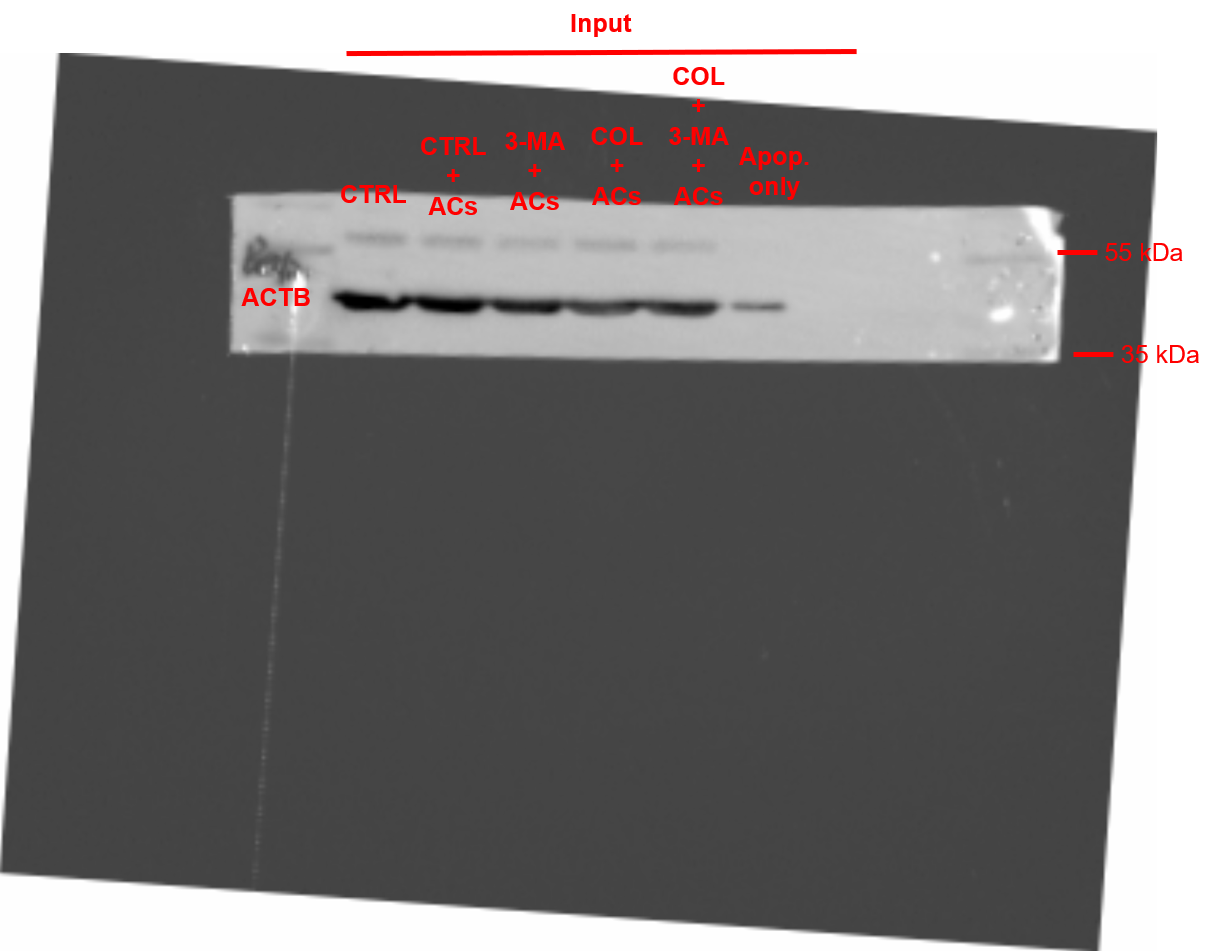

Supplement: Supplementary file 10 — Source Data for Figure 2 [file EMMM-15-e17815-s013.zip › Figure_2/2H/ACTB/Westernblot-ACTB.tif]

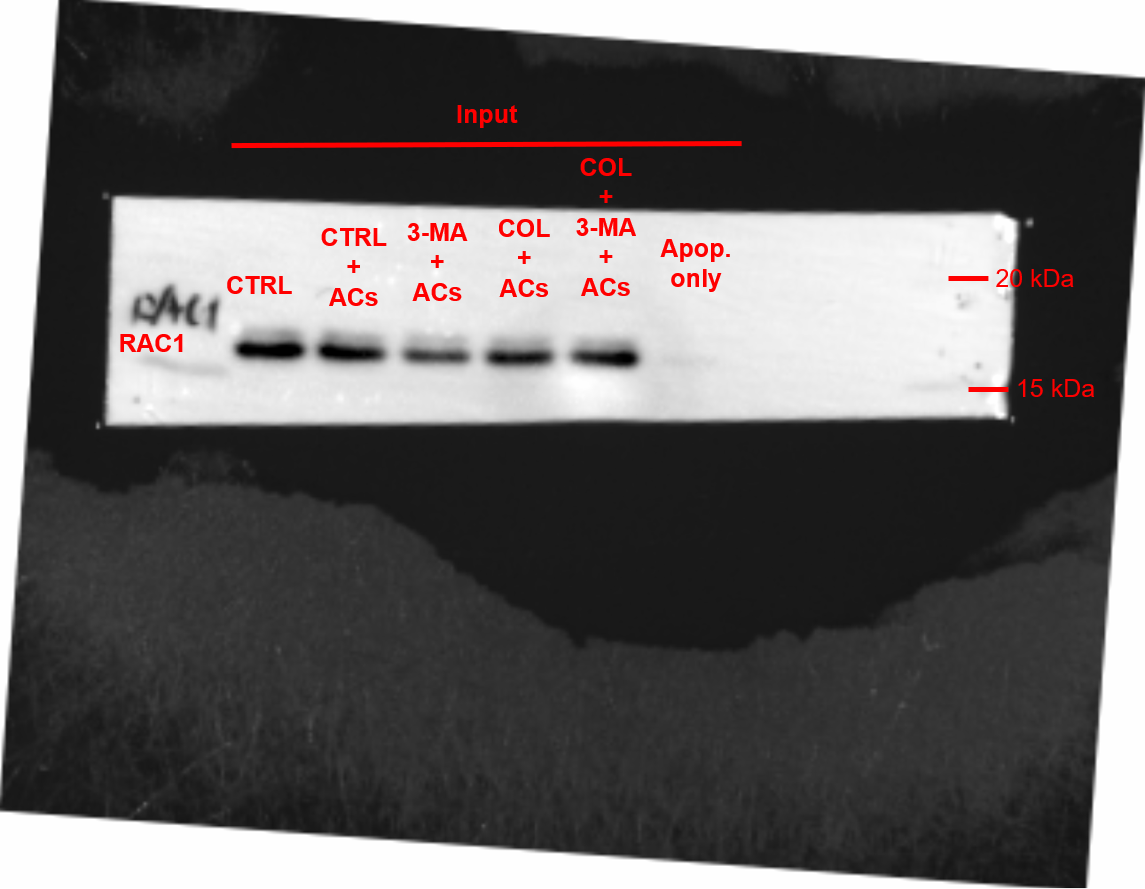

Supplement: Supplementary file 10 — Source Data for Figure 2 [file EMMM-15-e17815-s013.zip › Figure_2/2H/RAC1/Westernblot-RAC1-input.tif]

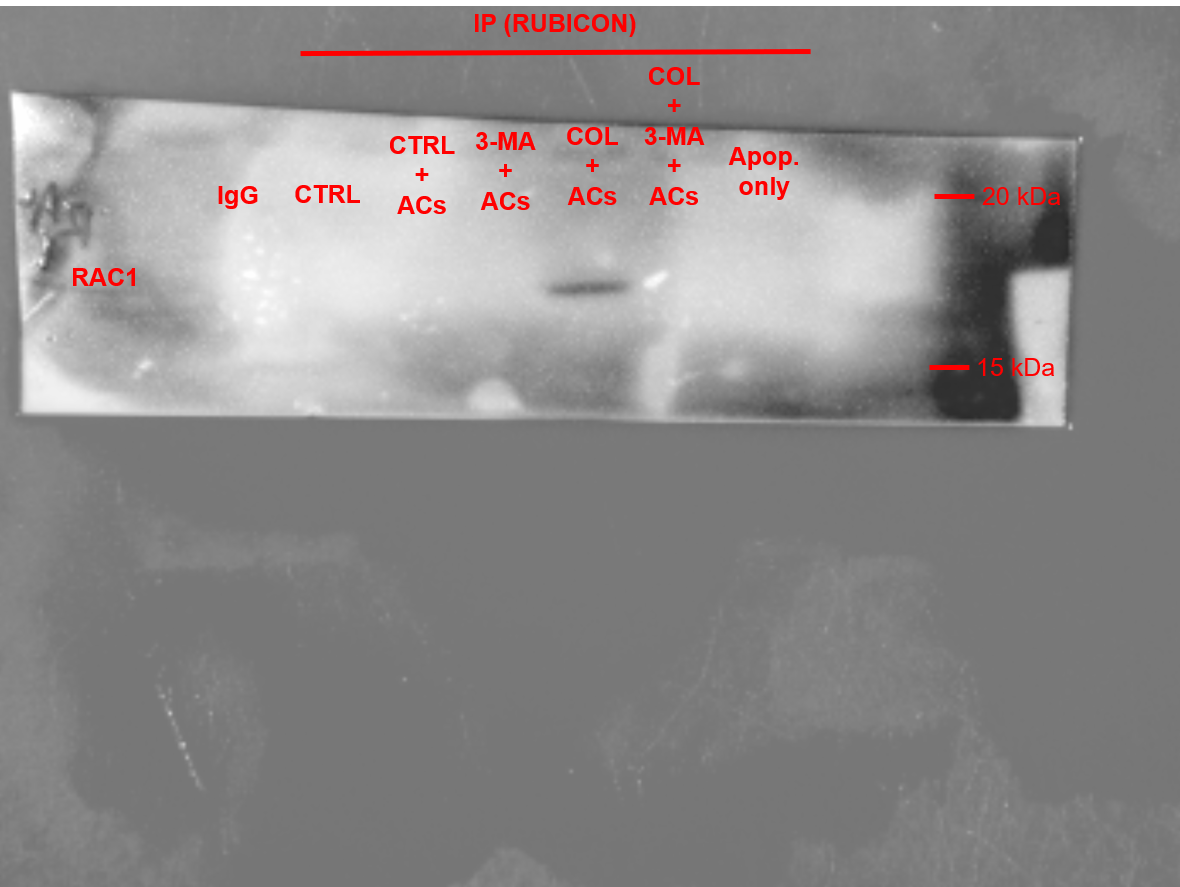

Supplement: Supplementary file 10 — Source Data for Figure 2 [file EMMM-15-e17815-s013.zip › Figure_2/2H/RAC1/Westernblot-RAC1-ip.tif]

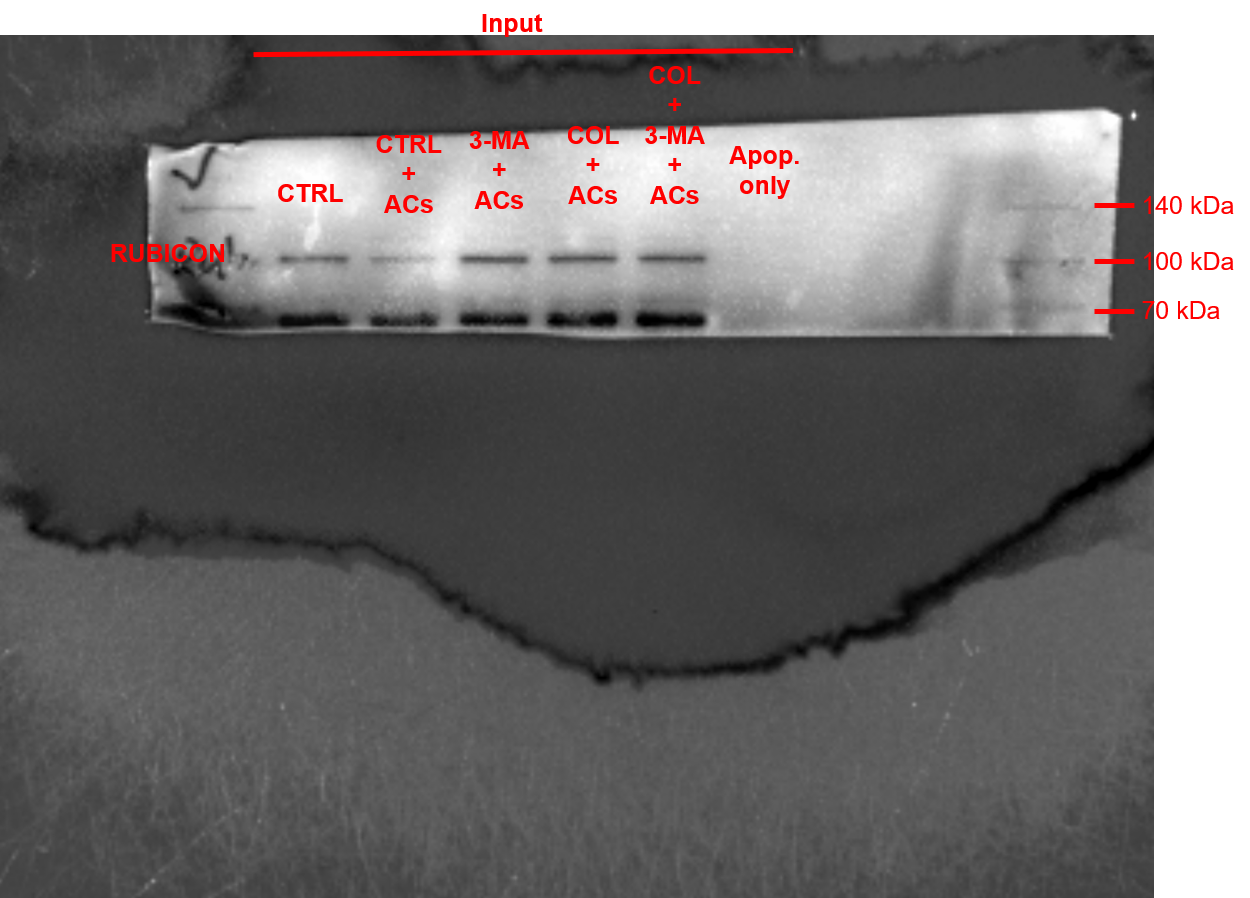

Supplement: Supplementary file 10 — Source Data for Figure 2 [file EMMM-15-e17815-s013.zip › Figure_2/2H/RUBICON/Westernblot-RUBICON_input.tif]

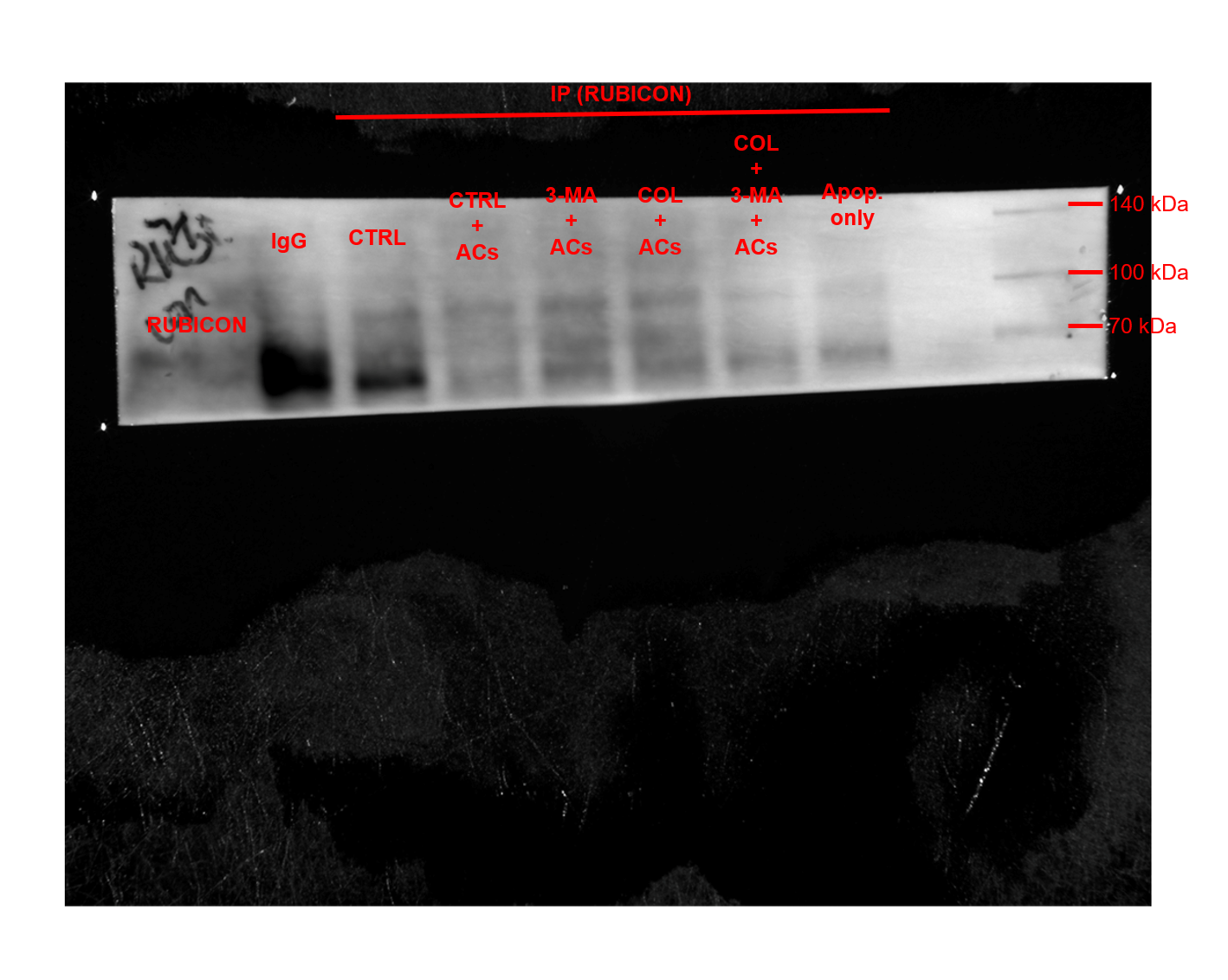

Supplement: Supplementary file 10 — Source Data for Figure 2 [file EMMM-15-e17815-s013.zip › Figure_2/2H/RUBICON/Westernblot-RUBICON_IP.tif]

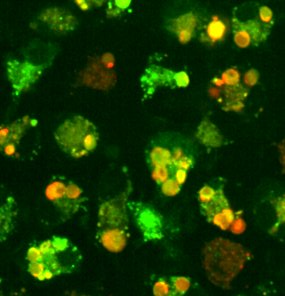

Supplement: Supplementary file 10 — Source Data for Figure 2 [file EMMM-15-e17815-s013.zip › Figure_2/2I/COL/images_micro._selected.tif]

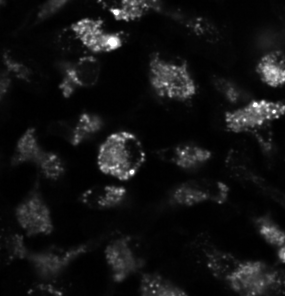

Supplement: Supplementary file 10 — Source Data for Figure 2 [file EMMM-15-e17815-s013.zip › Figure_2/2I/COL/time-lapse_images.tif]

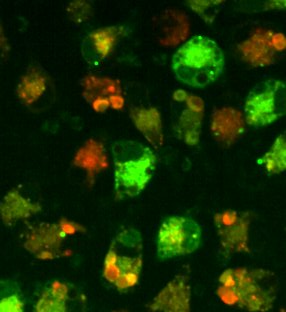

Supplement: Supplementary file 10 — Source Data for Figure 2 [file EMMM-15-e17815-s013.zip › Figure_2/2I/COL+3-MA/images_micro._selected.tif]

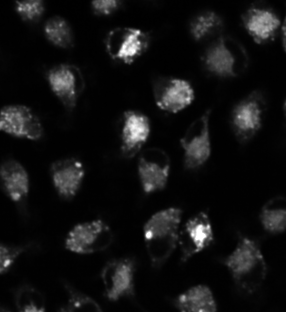

Supplement: Supplementary file 10 — Source Data for Figure 2 [file EMMM-15-e17815-s013.zip › Figure_2/2I/COL+3-MA/time-lapse_images.tif]

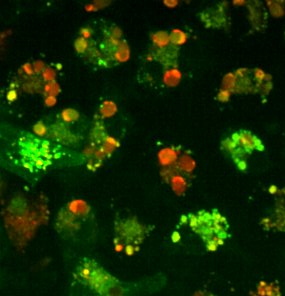

Supplement: Supplementary file 10 — Source Data for Figure 2 [file EMMM-15-e17815-s013.zip › Figure_2/2I/CTRL/images_micro._selected.tif]

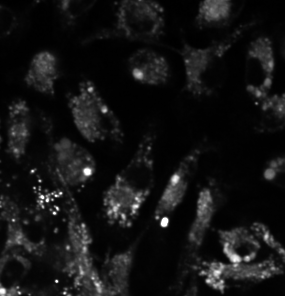

Supplement: Supplementary file 10 — Source Data for Figure 2 [file EMMM-15-e17815-s013.zip › Figure_2/2I/CTRL/time-lapse_images.tif]

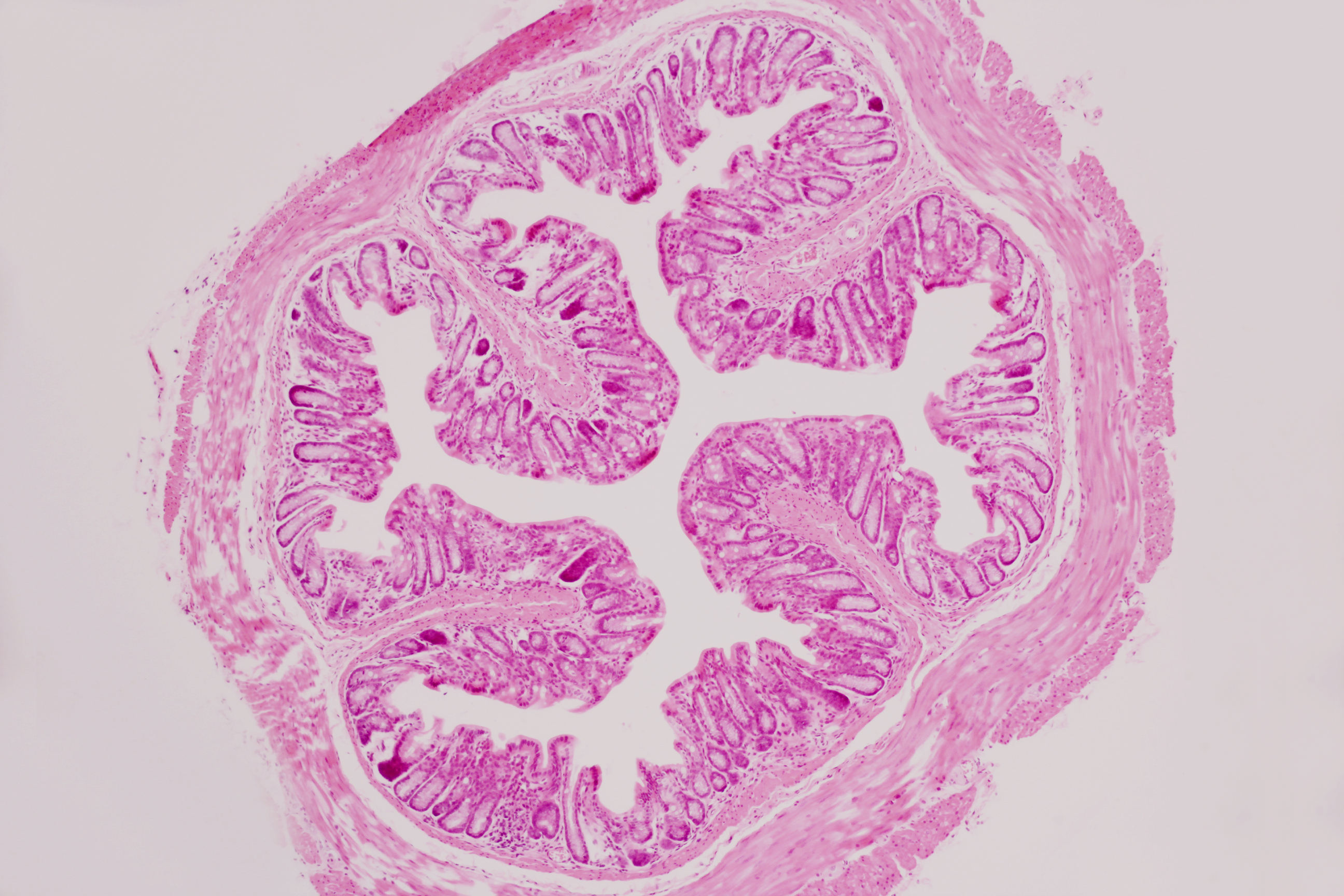

Supplement: Supplementary file 11 — Source Data for Figure 3 [file EMMM-15-e17815-s006.zip › Figure_3/3F/CTRL/IMG_6147.JPG]

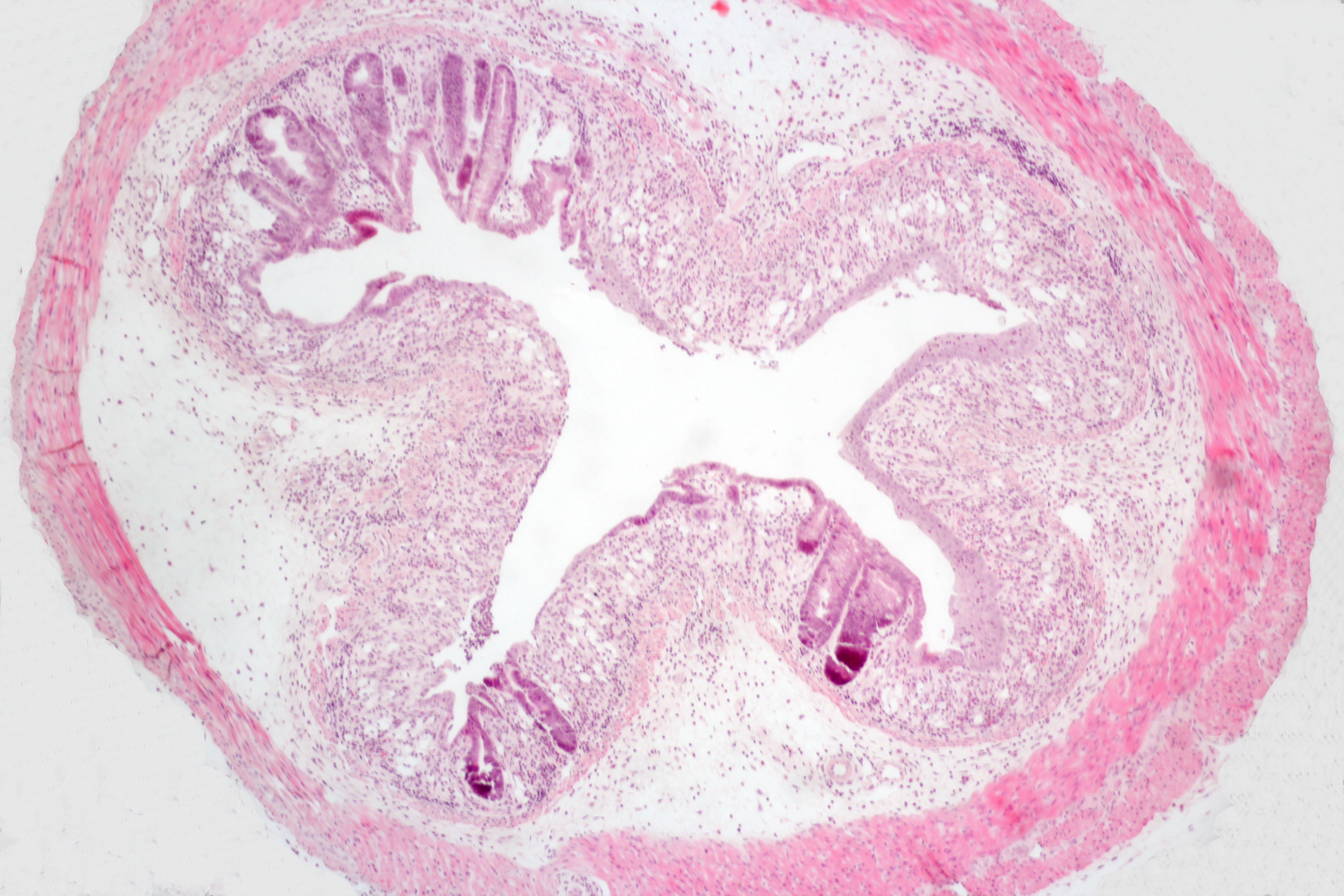

Supplement: Supplementary file 11 — Source Data for Figure 3 [file EMMM-15-e17815-s006.zip › Figure_3/3F/DSS-COL10/IMG_5919.JPG]

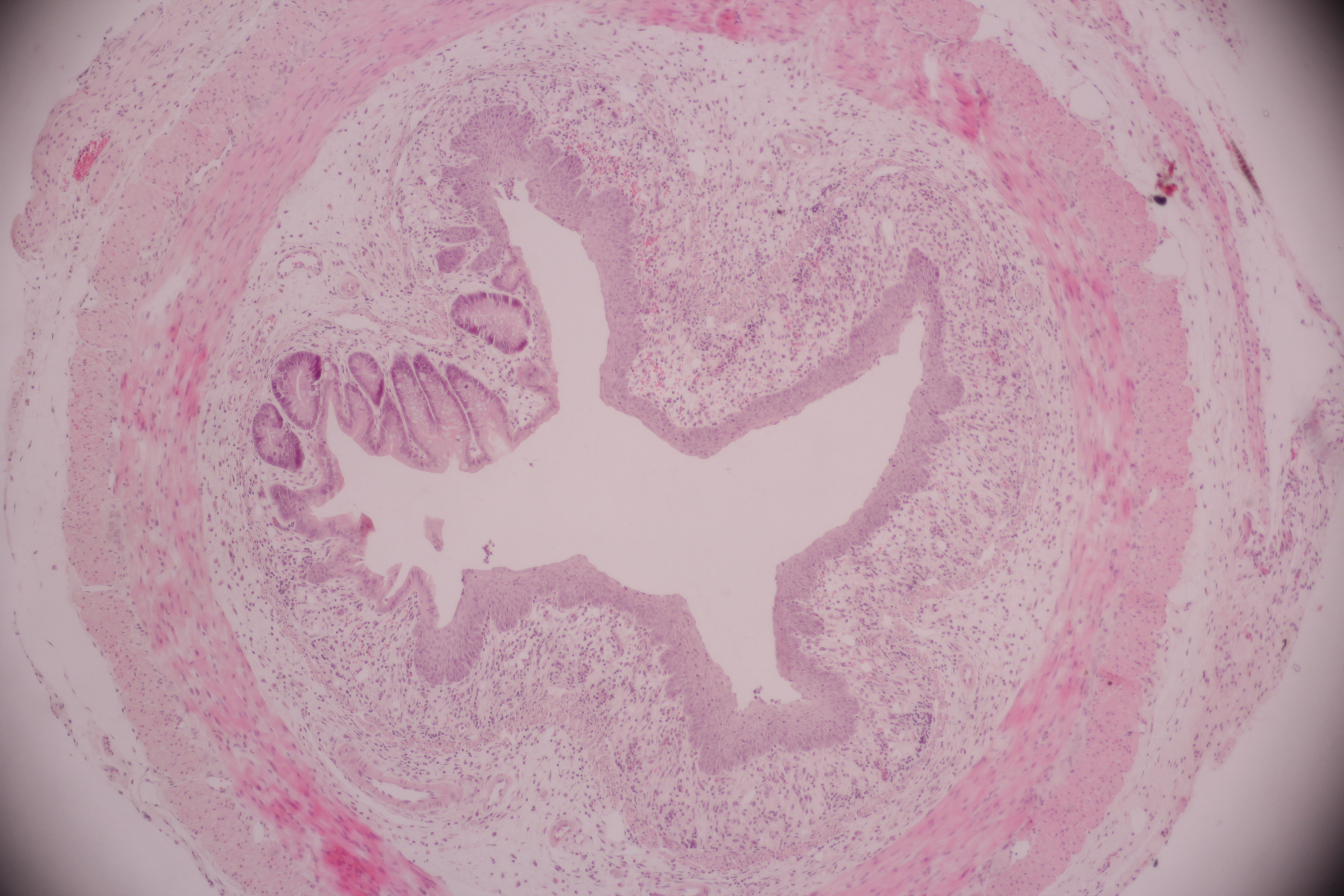

Supplement: Supplementary file 11 — Source Data for Figure 3 [file EMMM-15-e17815-s006.zip › Figure_3/3F/DSS-COL15/IMG_5720.JPG]

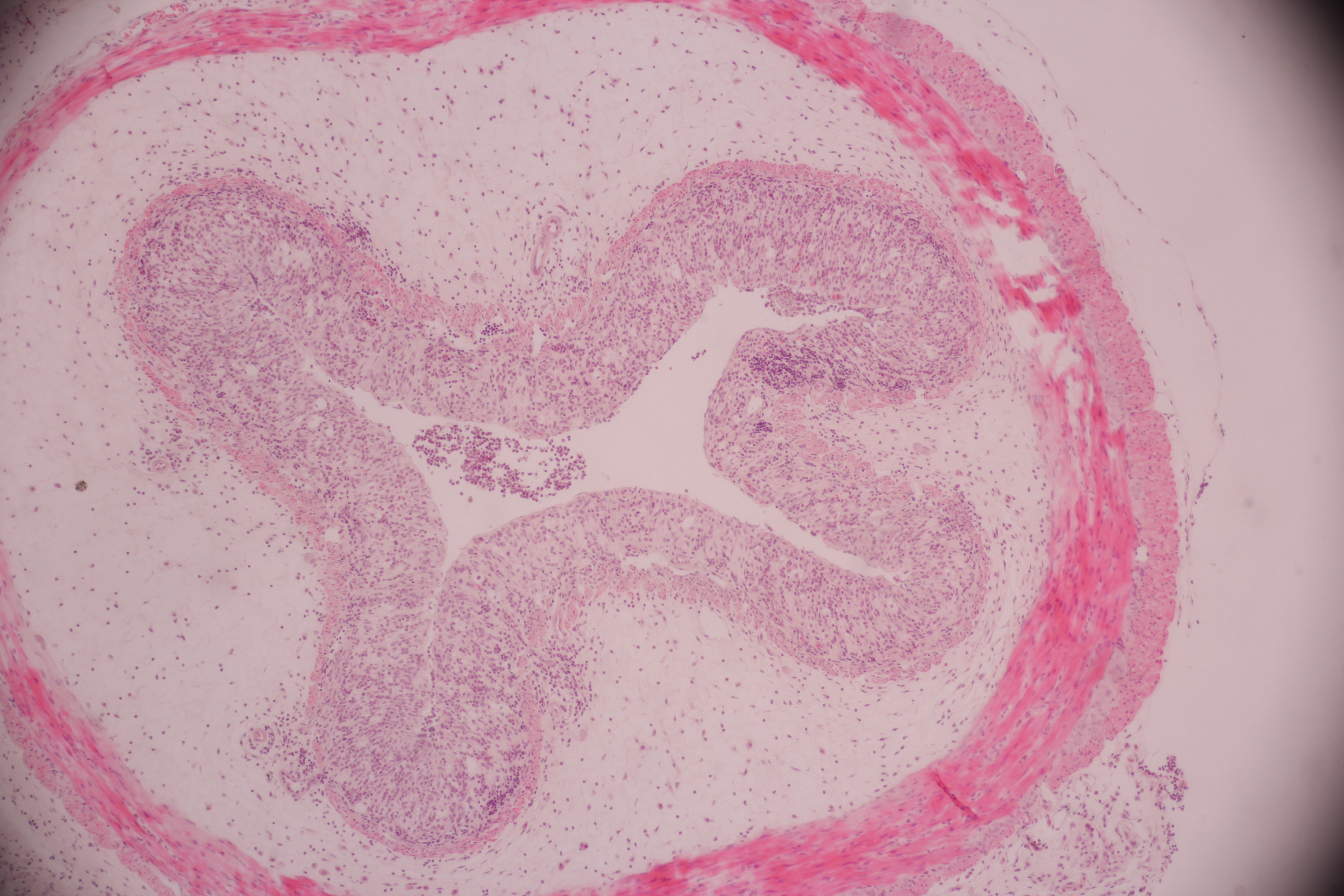

Supplement: Supplementary file 11 — Source Data for Figure 3 [file EMMM-15-e17815-s006.zip › Figure_3/3F/DSS-Vehicle/IMG_5938.JPG]

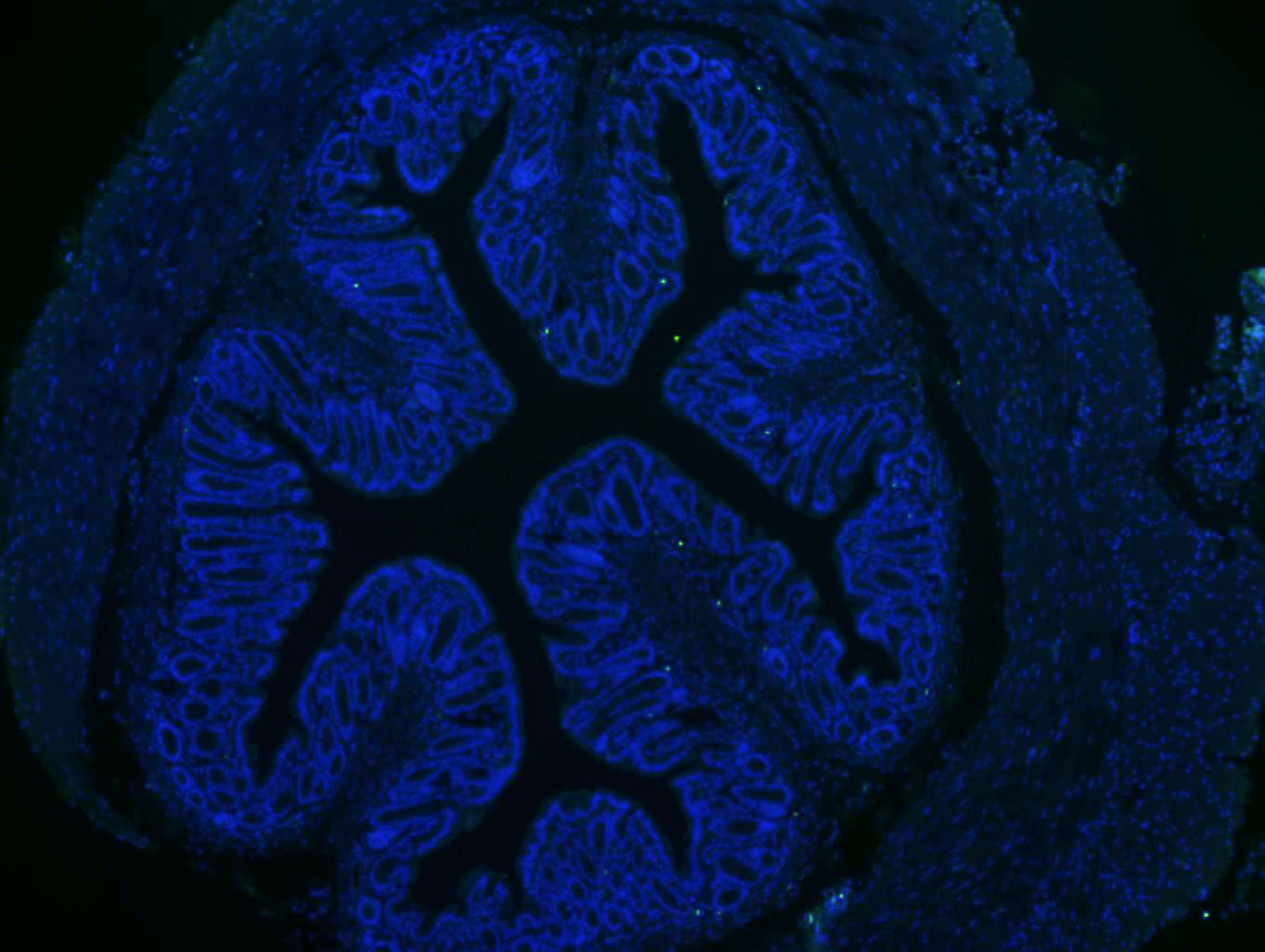

Supplement: Supplementary file 11 — Source Data for Figure 3 [file EMMM-15-e17815-s006.zip › Figure_3/3H/CTRL/MERGE.jpg]

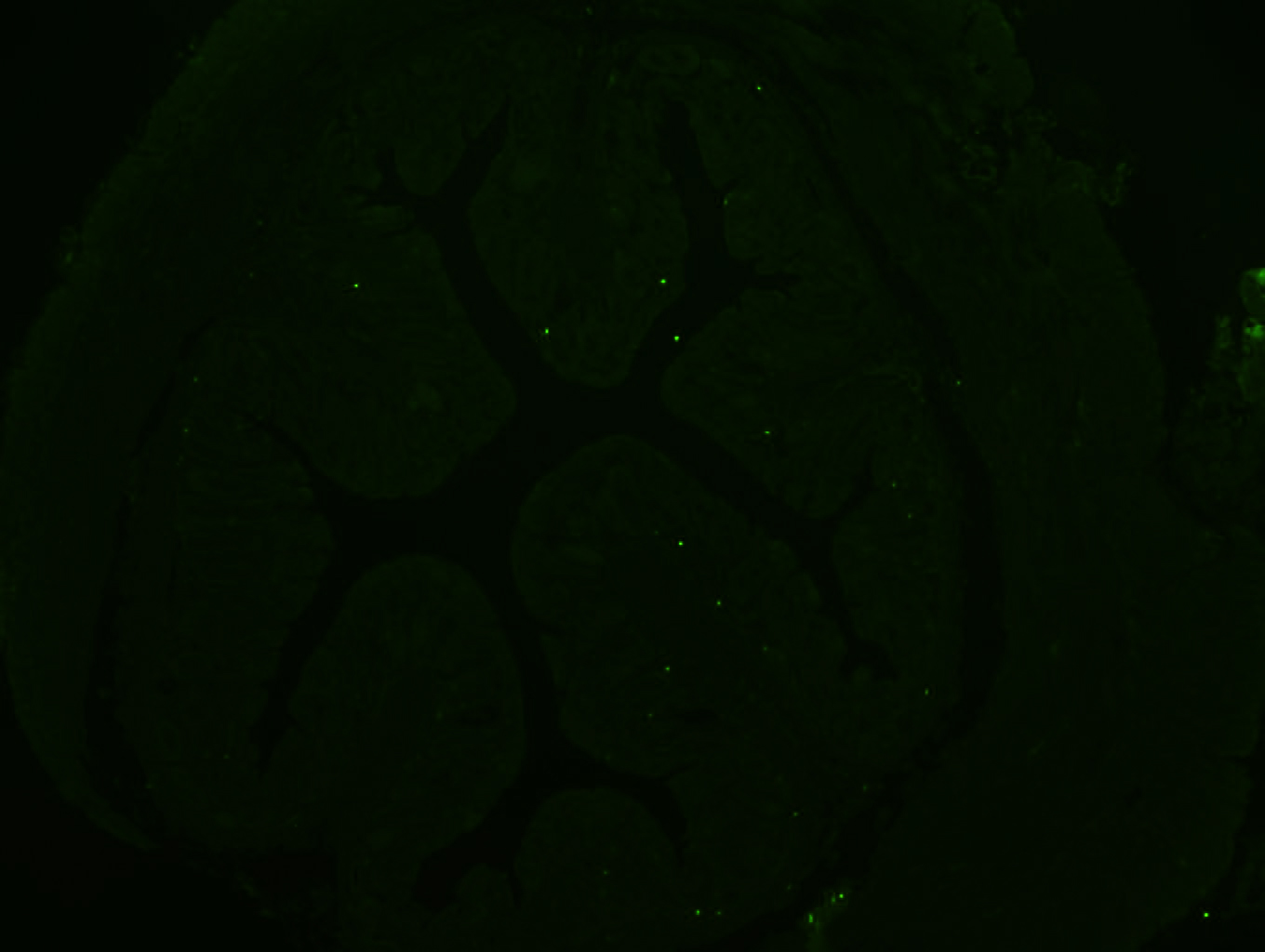

Supplement: Supplementary file 11 — Source Data for Figure 3 [file EMMM-15-e17815-s006.zip › Figure_3/3H/CTRL/TUNEL.jpg]

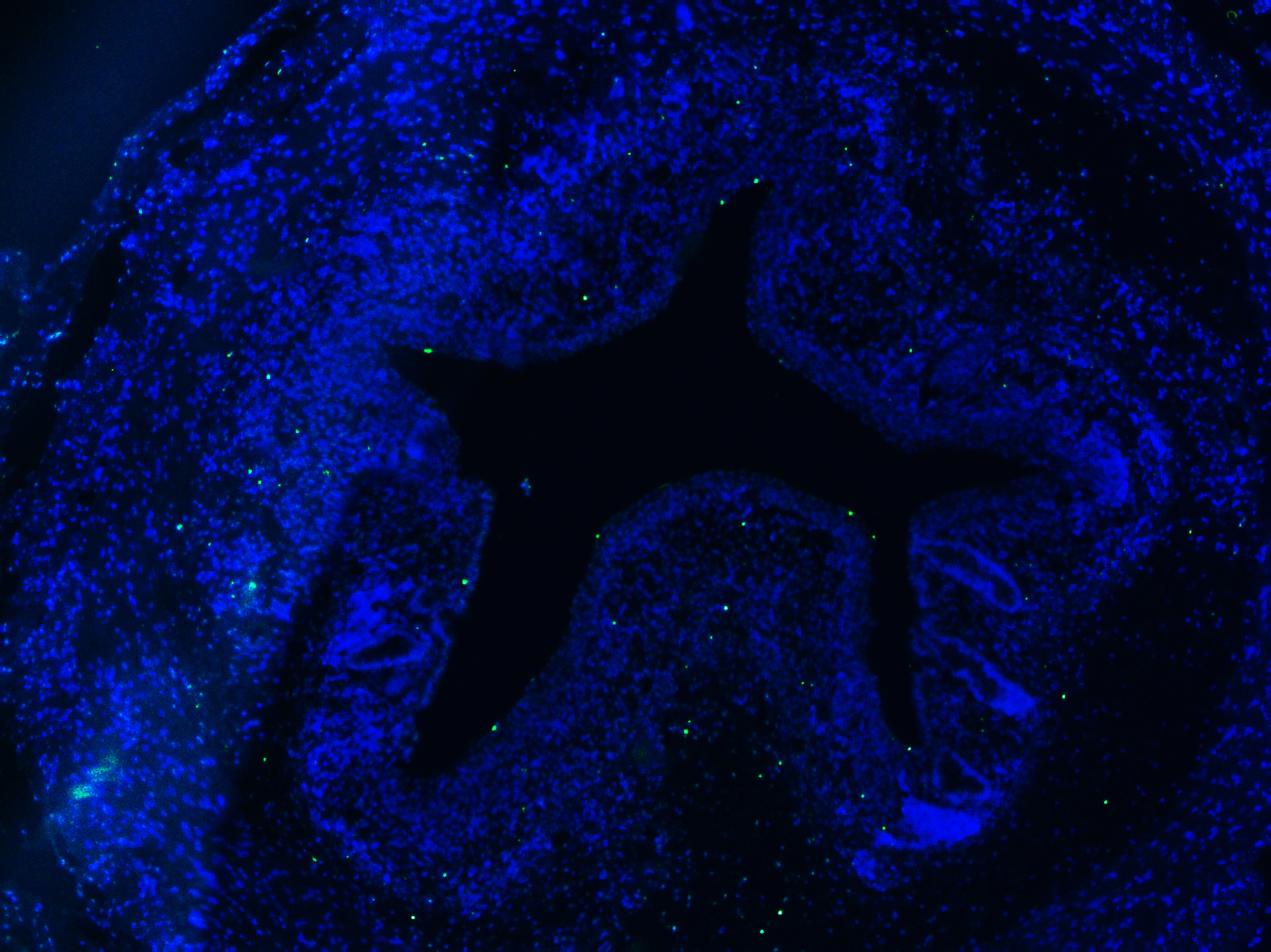

Supplement: Supplementary file 11 — Source Data for Figure 3 [file EMMM-15-e17815-s006.zip › Figure_3/3H/DSS-COL10/MERGE.jpg]

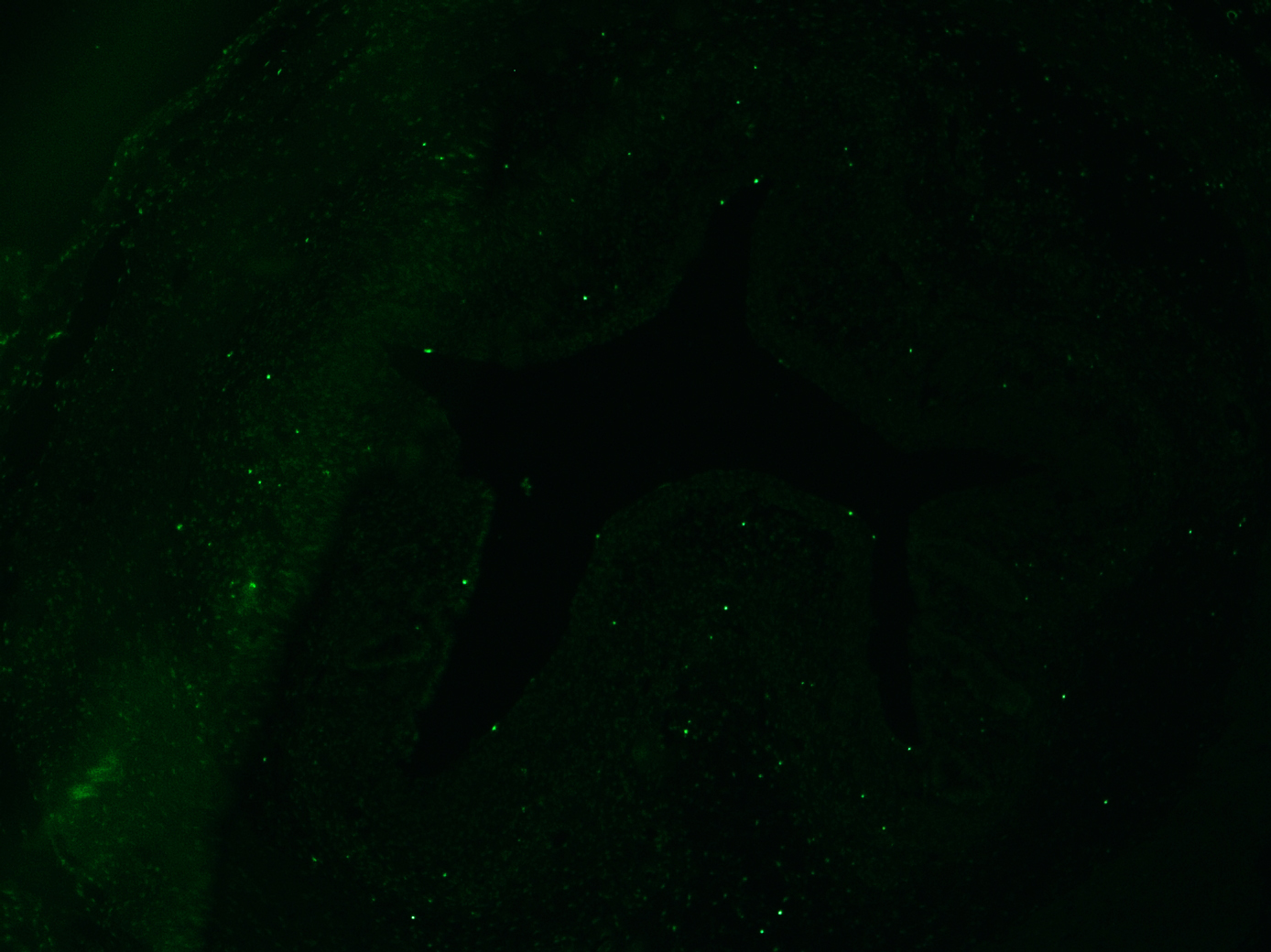

Supplement: Supplementary file 11 — Source Data for Figure 3 [file EMMM-15-e17815-s006.zip › Figure_3/3H/DSS-COL10/TUNEL.jpg]

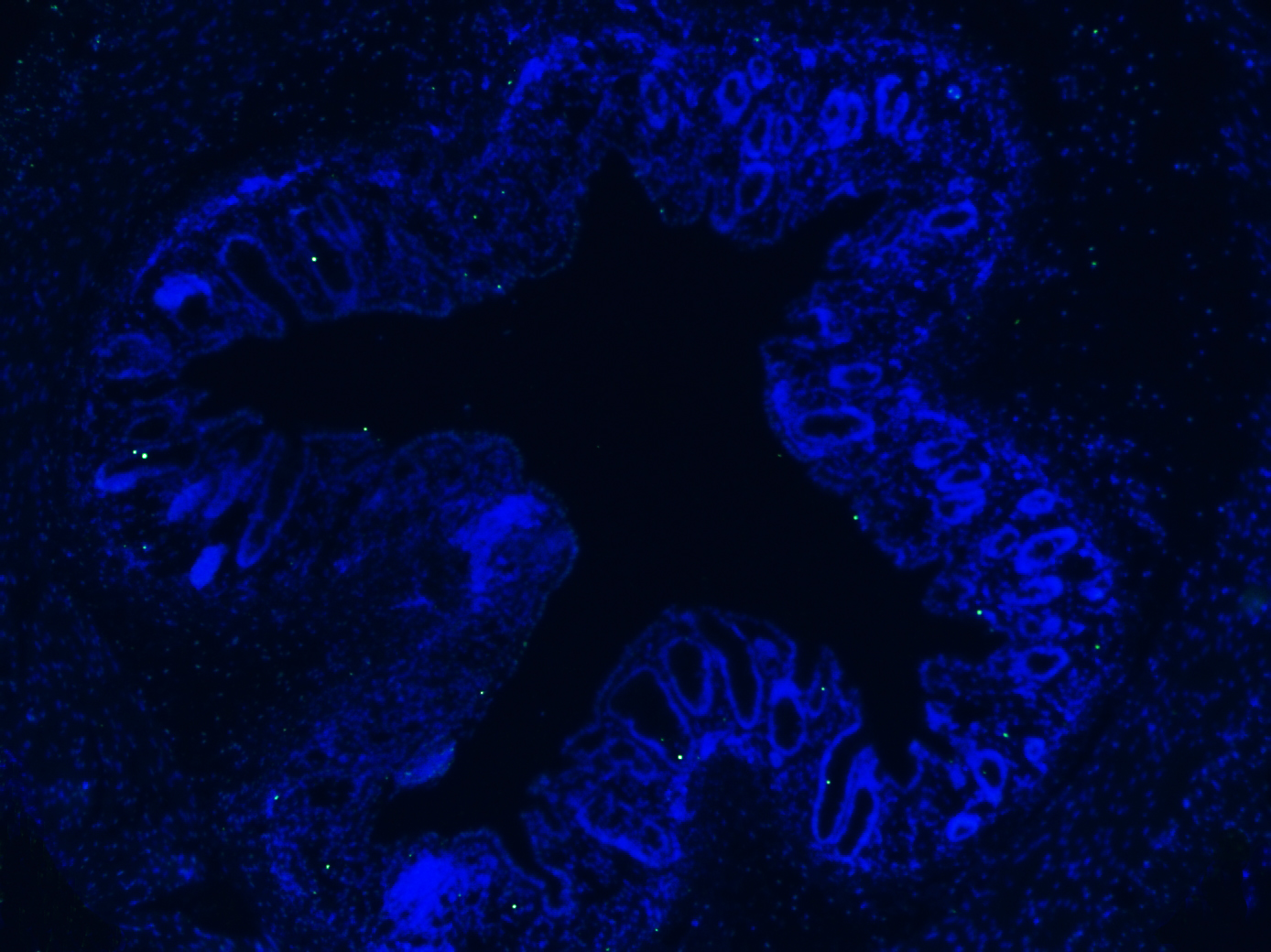

Supplement: Supplementary file 11 — Source Data for Figure 3 [file EMMM-15-e17815-s006.zip › Figure_3/3H/DSS-COL15/MERGE.jpg]

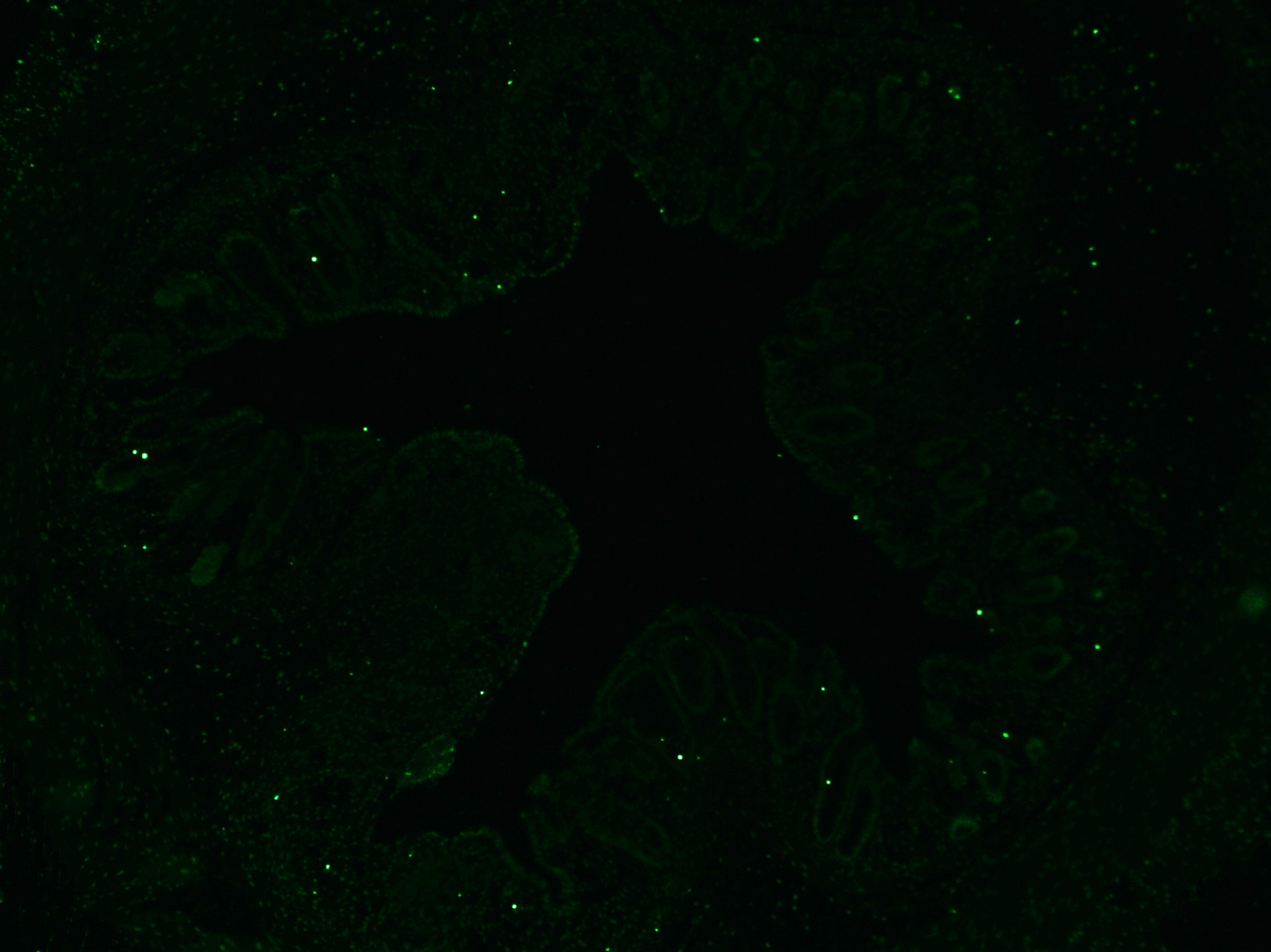

Supplement: Supplementary file 11 — Source Data for Figure 3 [file EMMM-15-e17815-s006.zip › Figure_3/3H/DSS-COL15/TUNEL.jpg]

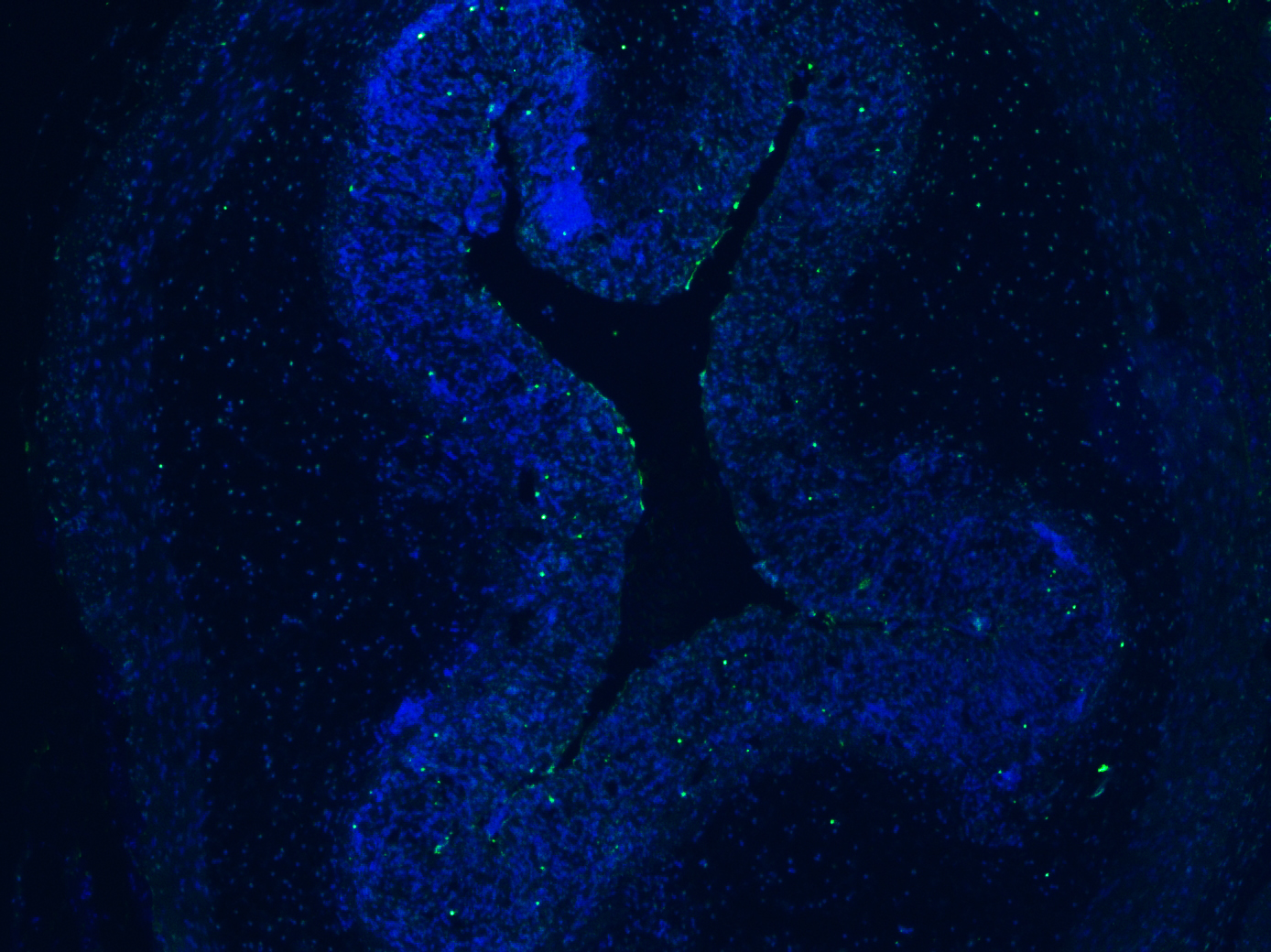

Supplement: Supplementary file 11 — Source Data for Figure 3 [file EMMM-15-e17815-s006.zip › Figure_3/3H/DSS-Vehicle/MERGE.jpg]

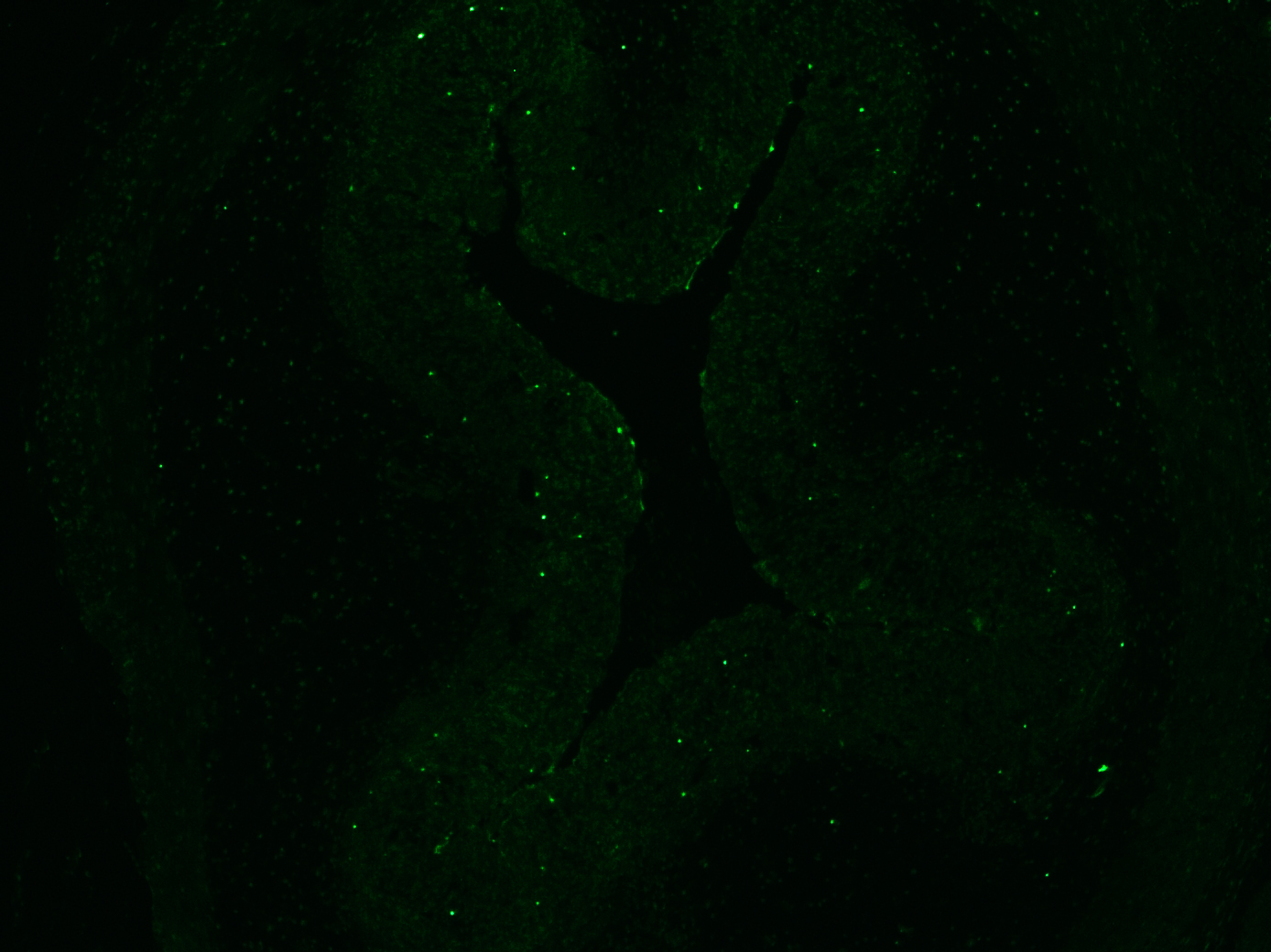

Supplement: Supplementary file 11 — Source Data for Figure 3 [file EMMM-15-e17815-s006.zip › Figure_3/3H/DSS-Vehicle/TUNEL.jpg]

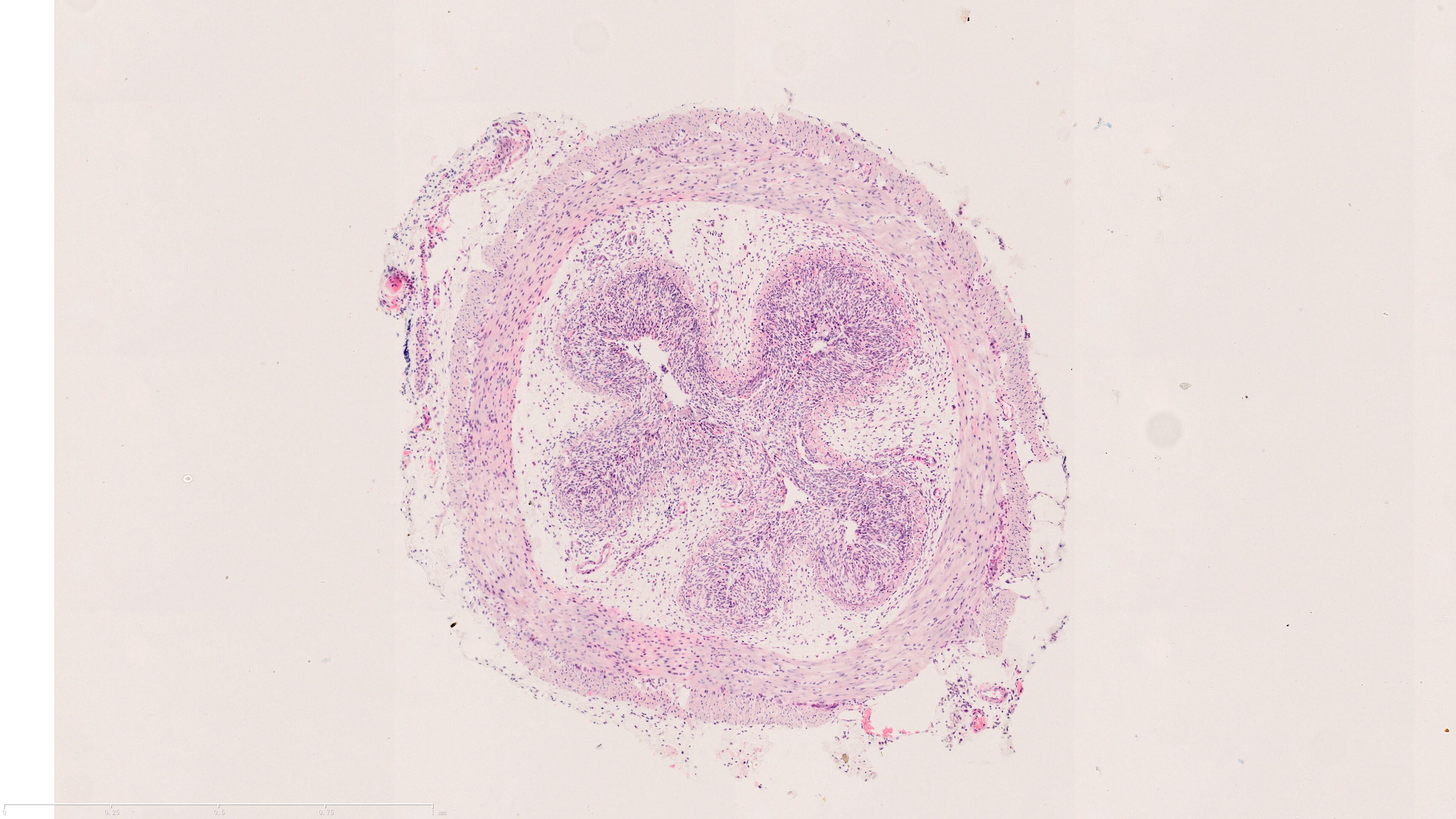

Supplement: Supplementary file 12 — Source Data for Figure 4 [file EMMM-15-e17815-s005.zip › Figure_4/4G/Clodronate-COL/CLO-COL.jpg]

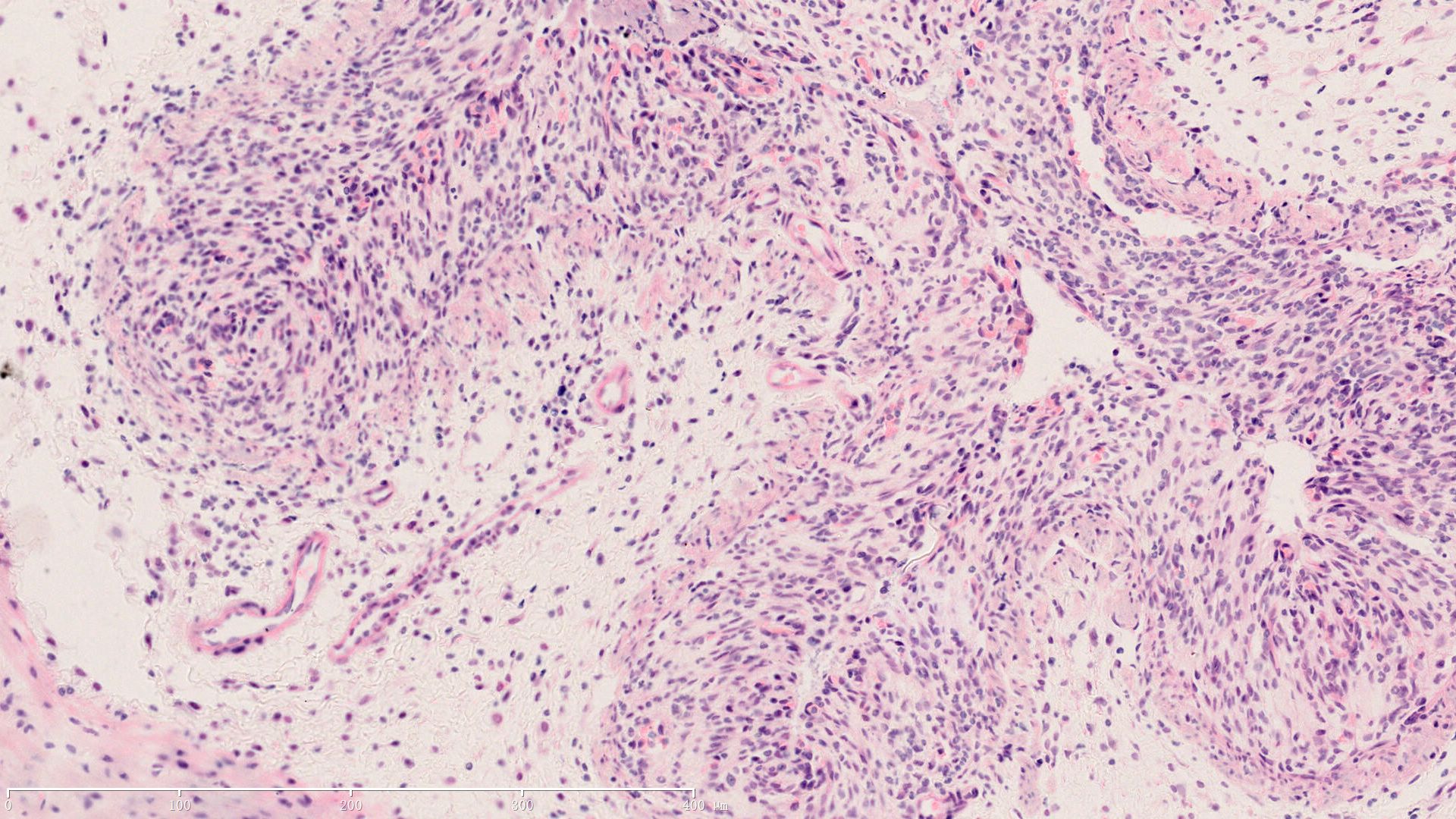

Supplement: Supplementary file 12 — Source Data for Figure 4 [file EMMM-15-e17815-s005.zip › Figure_4/4G/Clodronate-COL/CLO-COLx20.jpg]

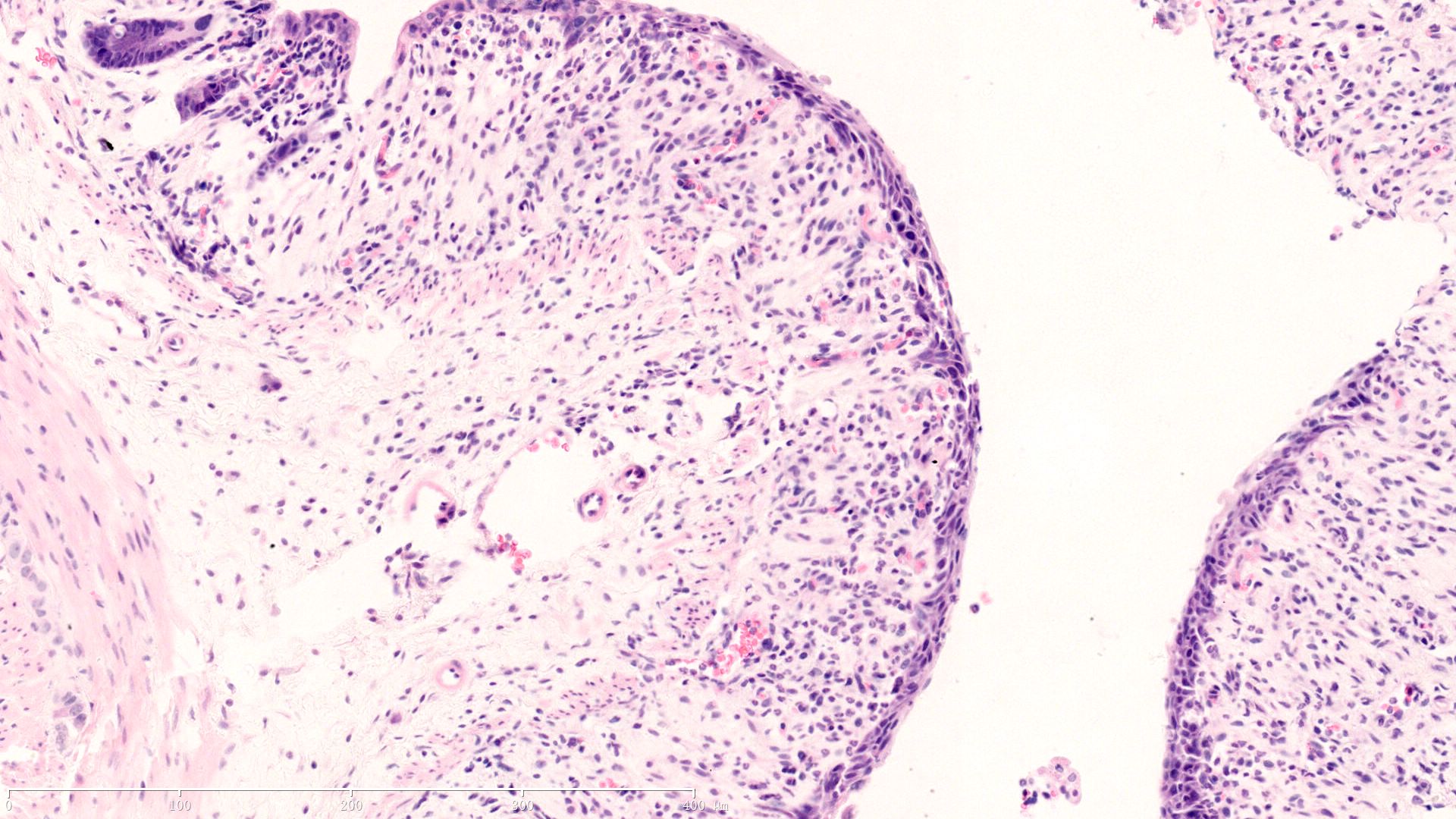

Supplement: Supplementary file 12 — Source Data for Figure 4 [file EMMM-15-e17815-s005.zip › Figure_4/4G/Clodronate-CTRL/CLO-CTRL-X20.jpg]

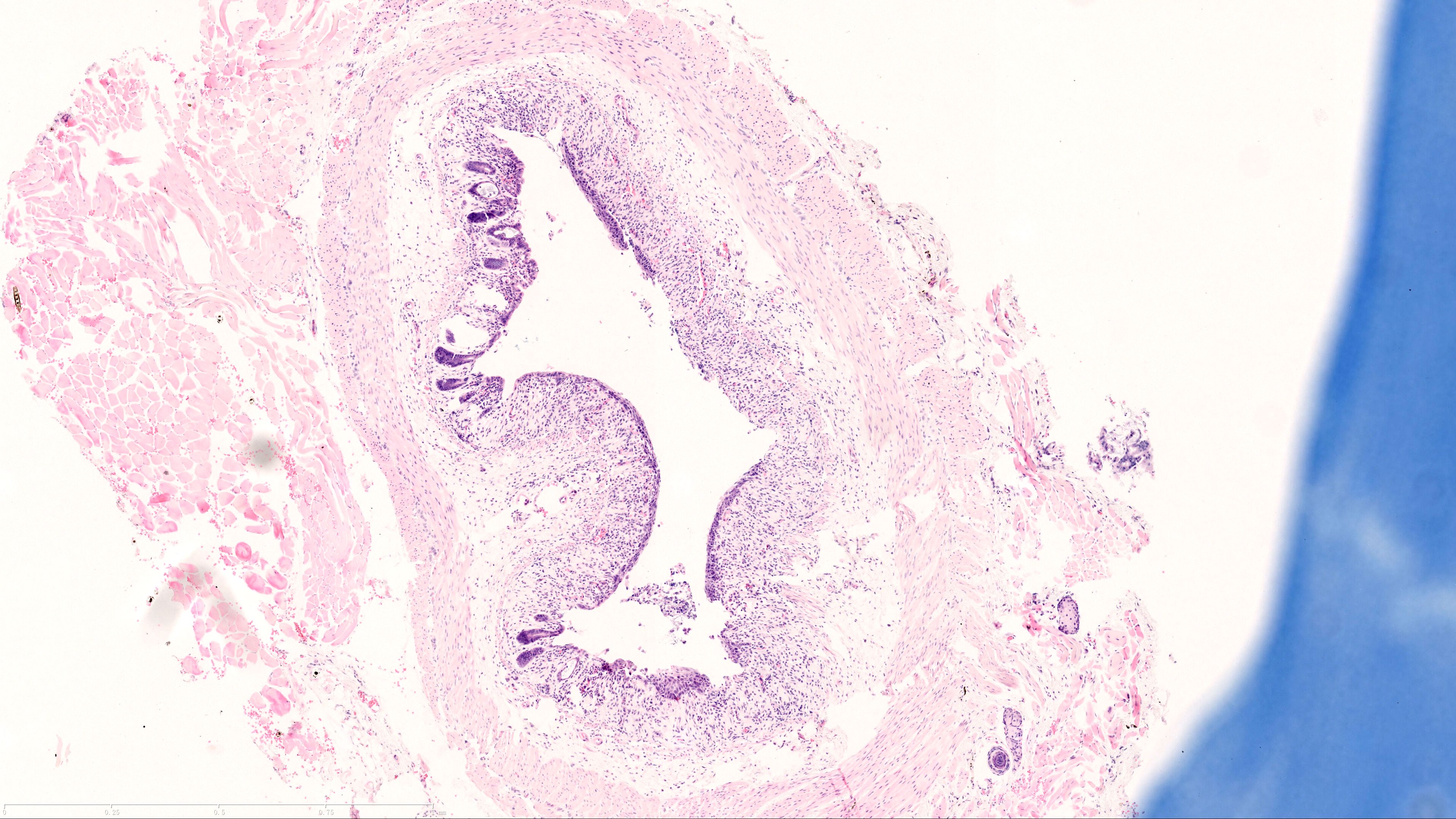

Supplement: Supplementary file 12 — Source Data for Figure 4 [file EMMM-15-e17815-s005.zip › Figure_4/4G/Clodronate-CTRL/CLO-CTRL.jpg]

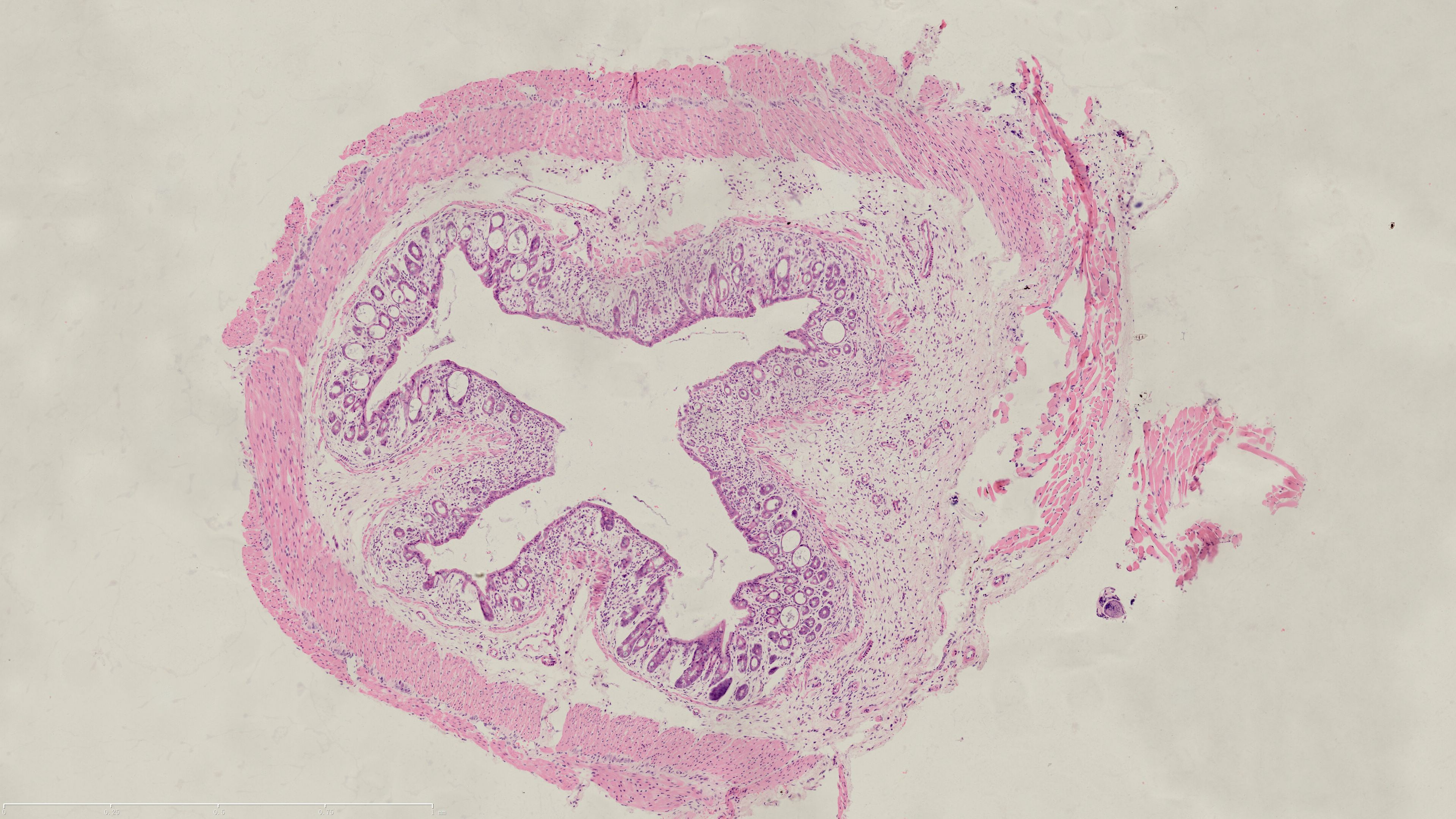

Supplement: Supplementary file 12 — Source Data for Figure 4 [file EMMM-15-e17815-s005.zip › Figure_4/4G/PBS-COL/pbs-co2l.jpg]

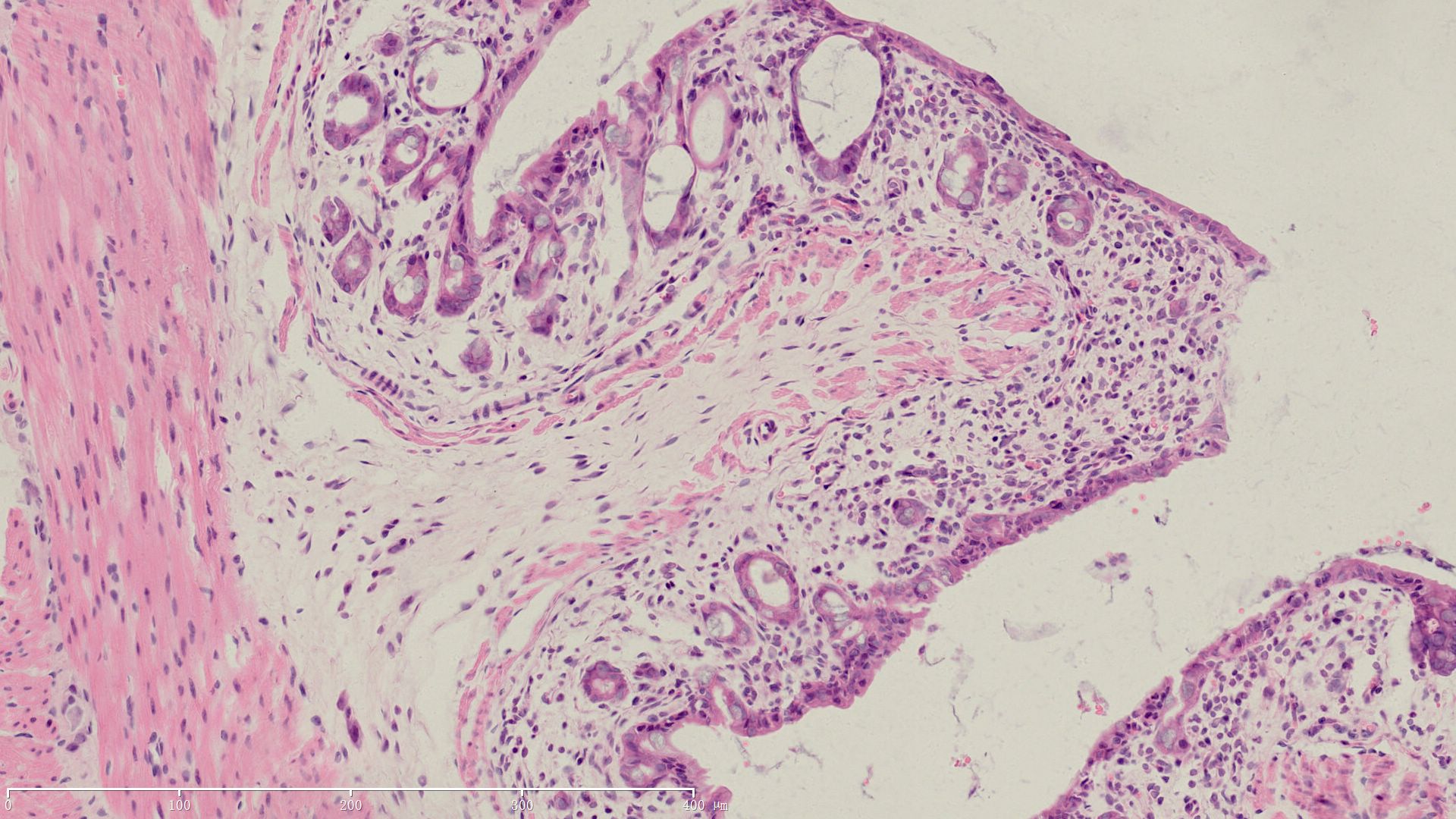

Supplement: Supplementary file 12 — Source Data for Figure 4 [file EMMM-15-e17815-s005.zip › Figure_4/4G/PBS-COL/pbs-co2lx20.jpg]

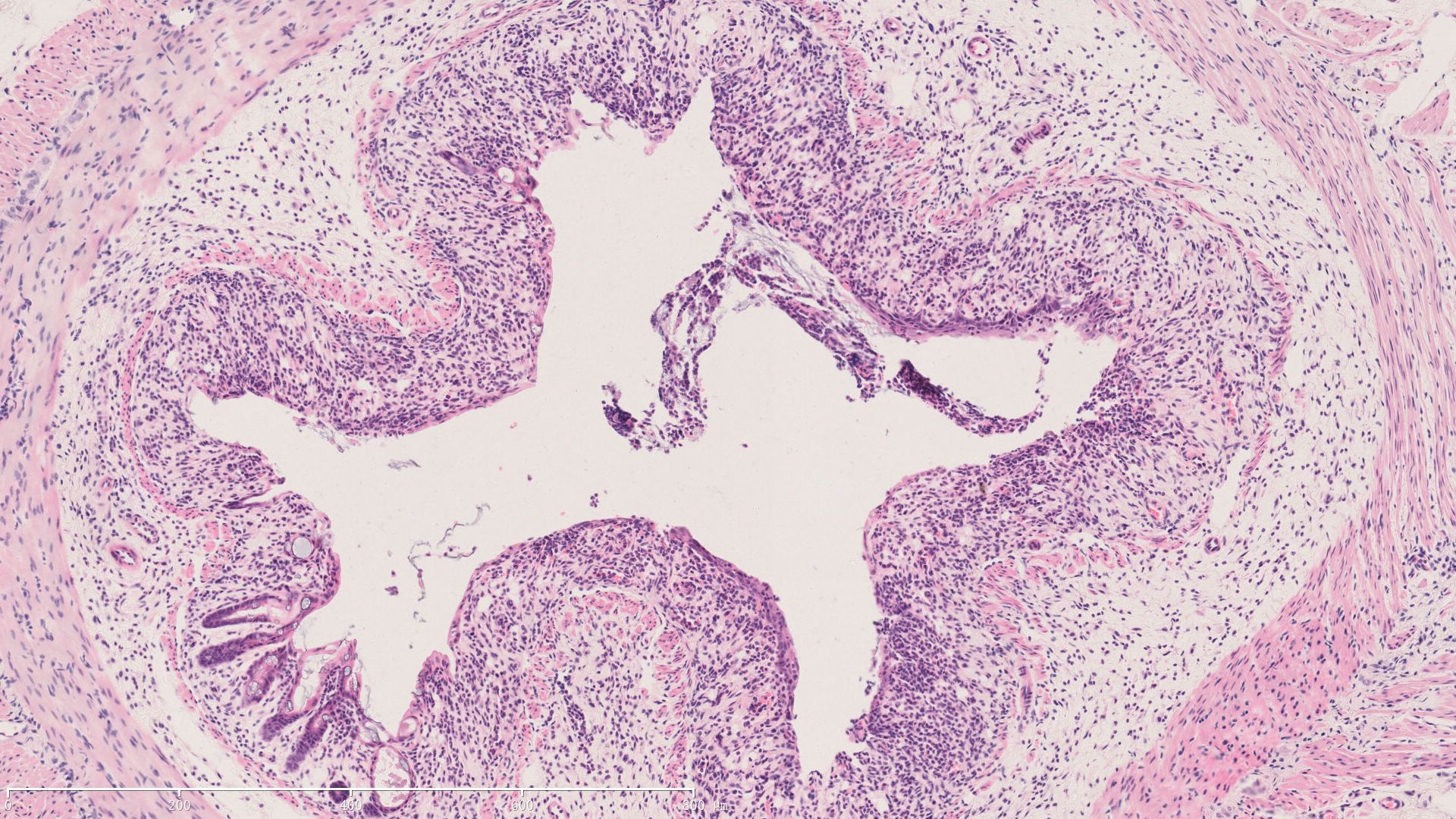

Supplement: Supplementary file 12 — Source Data for Figure 4 [file EMMM-15-e17815-s005.zip › Figure_4/4G/PBS-CTRL/pbs-ctrl.jpg]

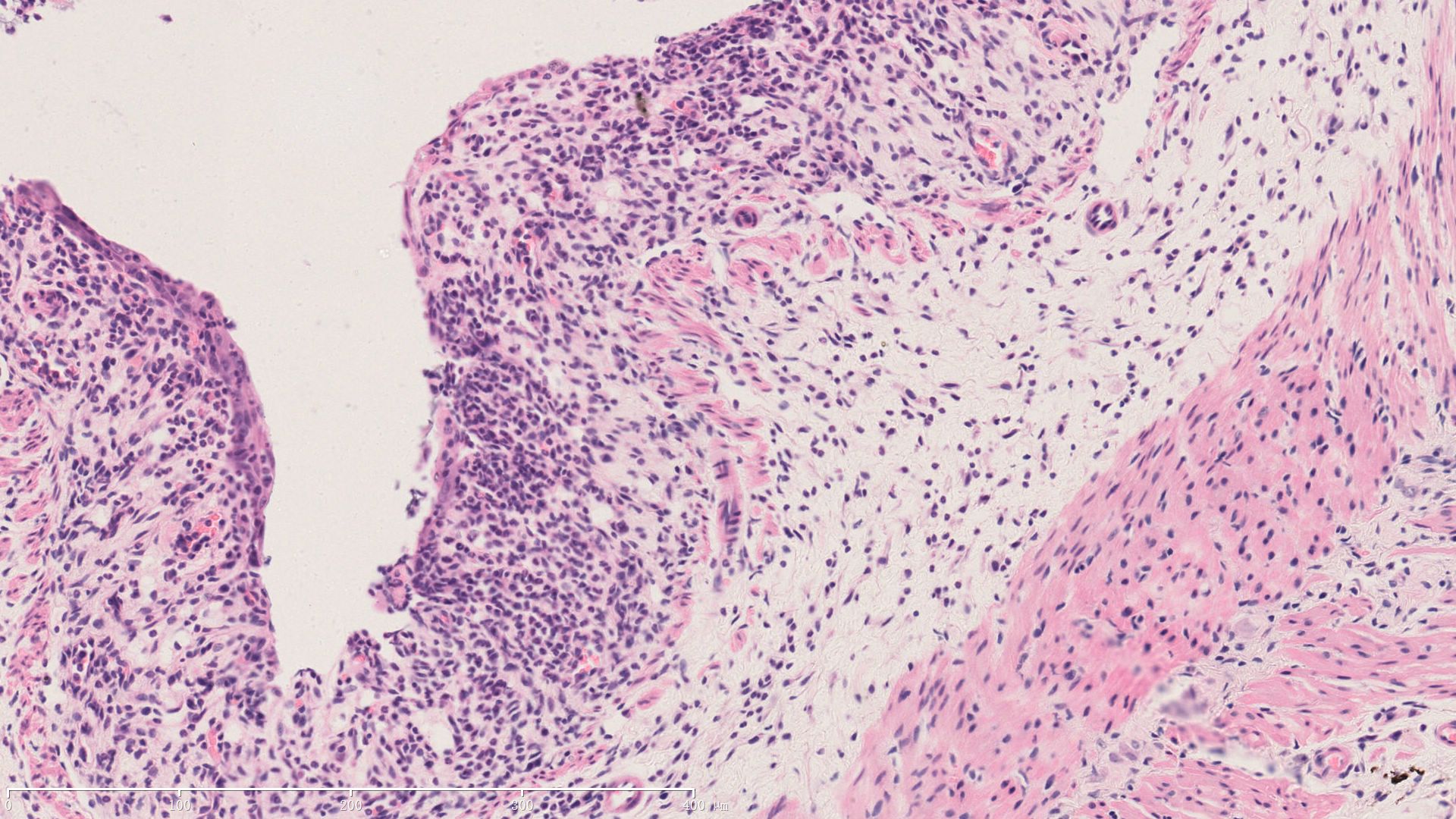

Supplement: Supplementary file 12 — Source Data for Figure 4 [file EMMM-15-e17815-s005.zip › Figure_4/4G/PBS-CTRL/pbs-ctrlx20.jpg]

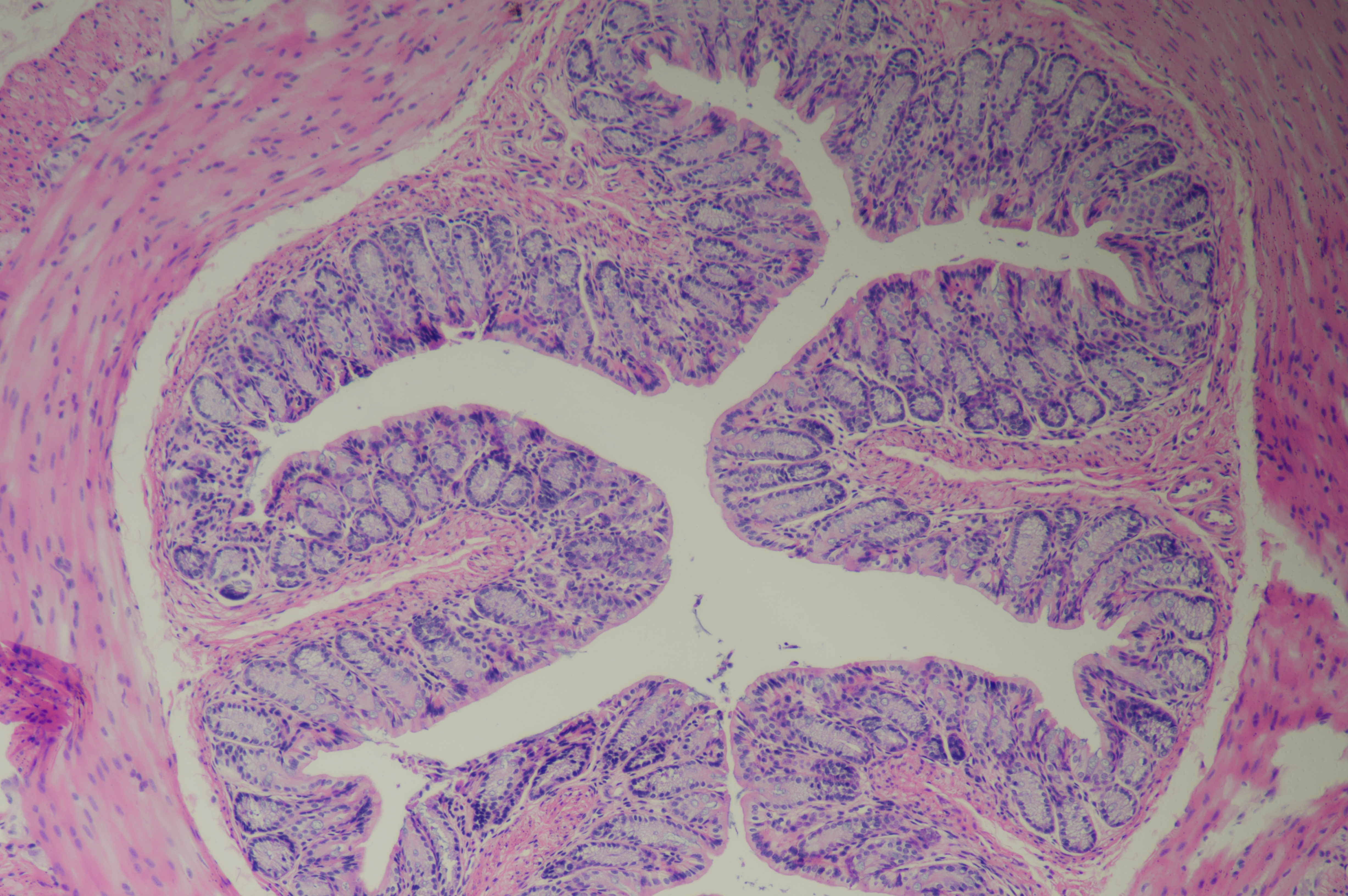

Supplement: Supplementary file 14 — Source Data for Figure 6 [file EMMM-15-e17815-s001.zip › Figure_6/6G/FPR2_KO-CTRL/HE-image.tif]

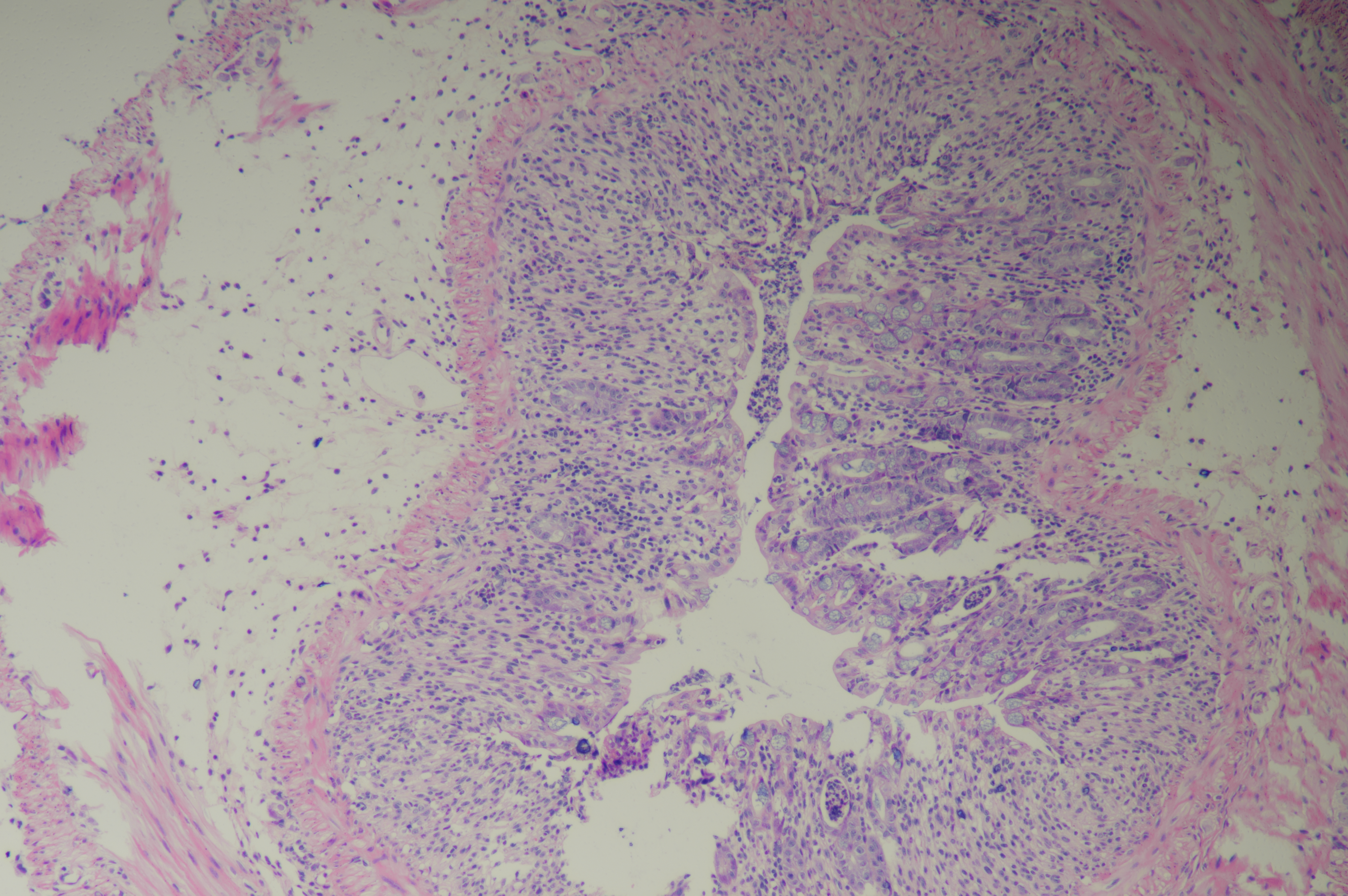

Supplement: Supplementary file 14 — Source Data for Figure 6 [file EMMM-15-e17815-s001.zip › Figure_6/6G/FPR2_KO-DSS-COL/HE_image.tif]

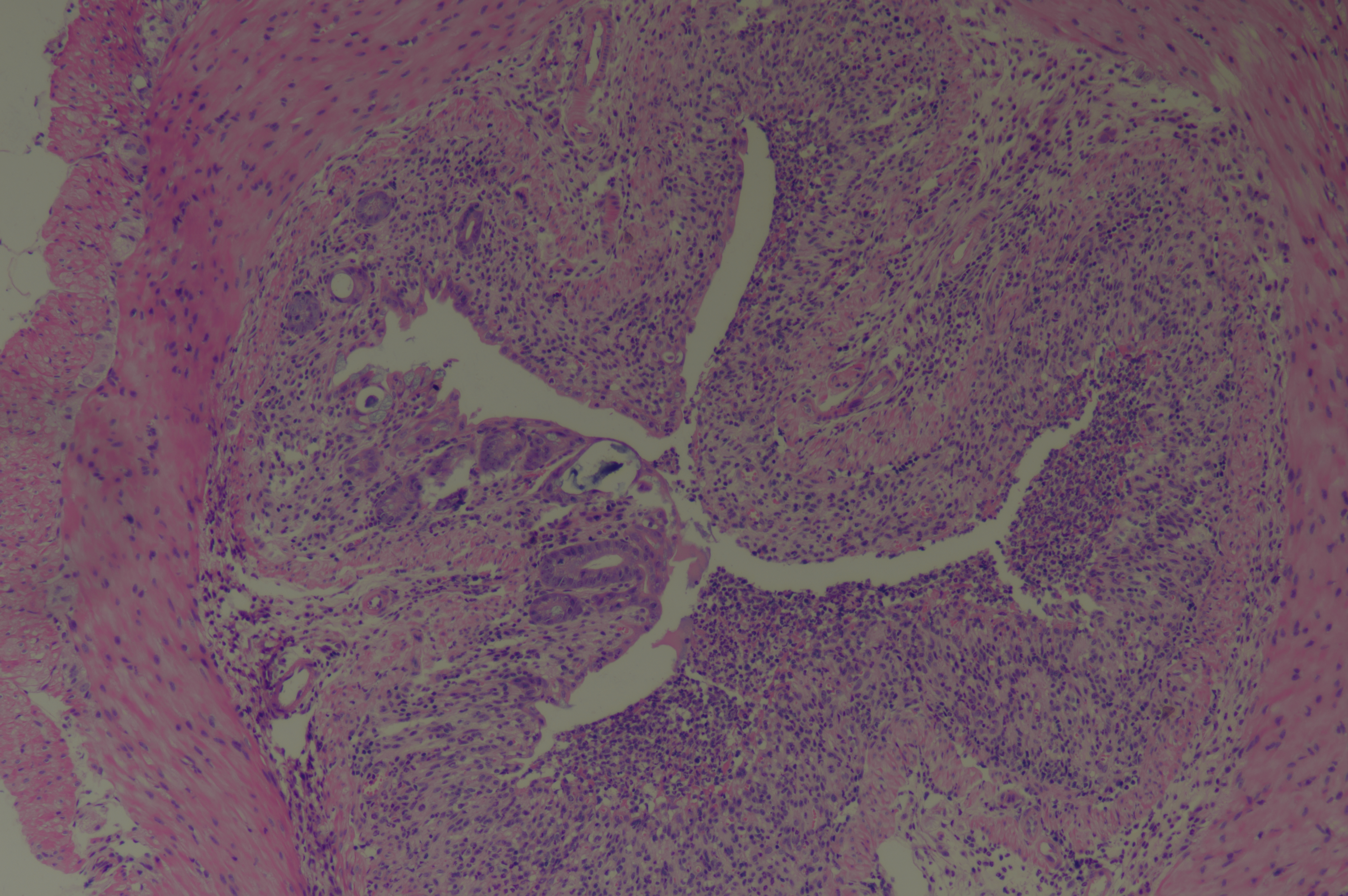

Supplement: Supplementary file 14 — Source Data for Figure 6 [file EMMM-15-e17815-s001.zip › Figure_6/6G/FPR2_KO-DSS-Vehicle/HE_image.tif]

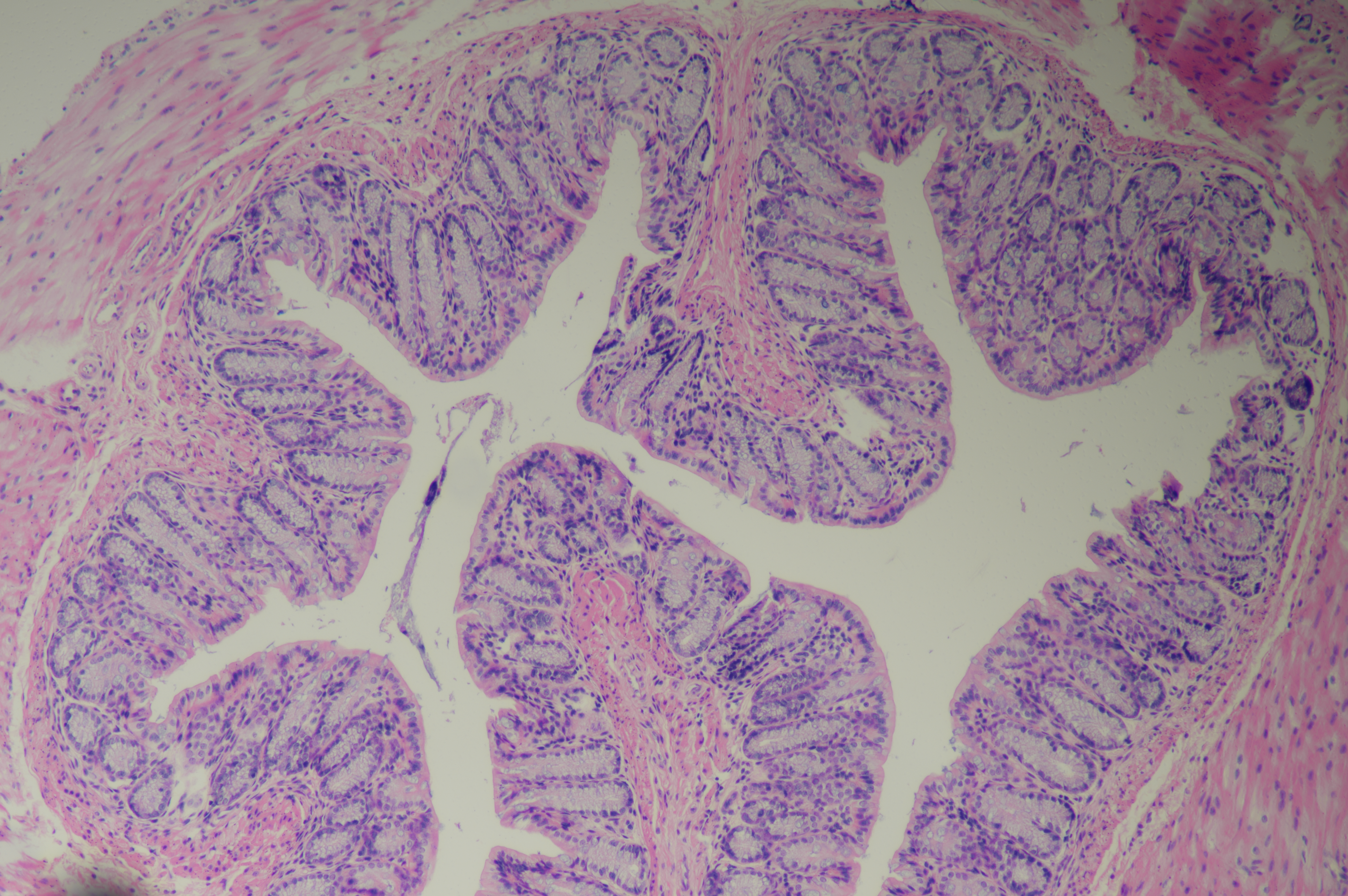

Supplement: Supplementary file 14 — Source Data for Figure 6 [file EMMM-15-e17815-s001.zip › Figure_6/6G/WT-CTRL/HE_image.tif]

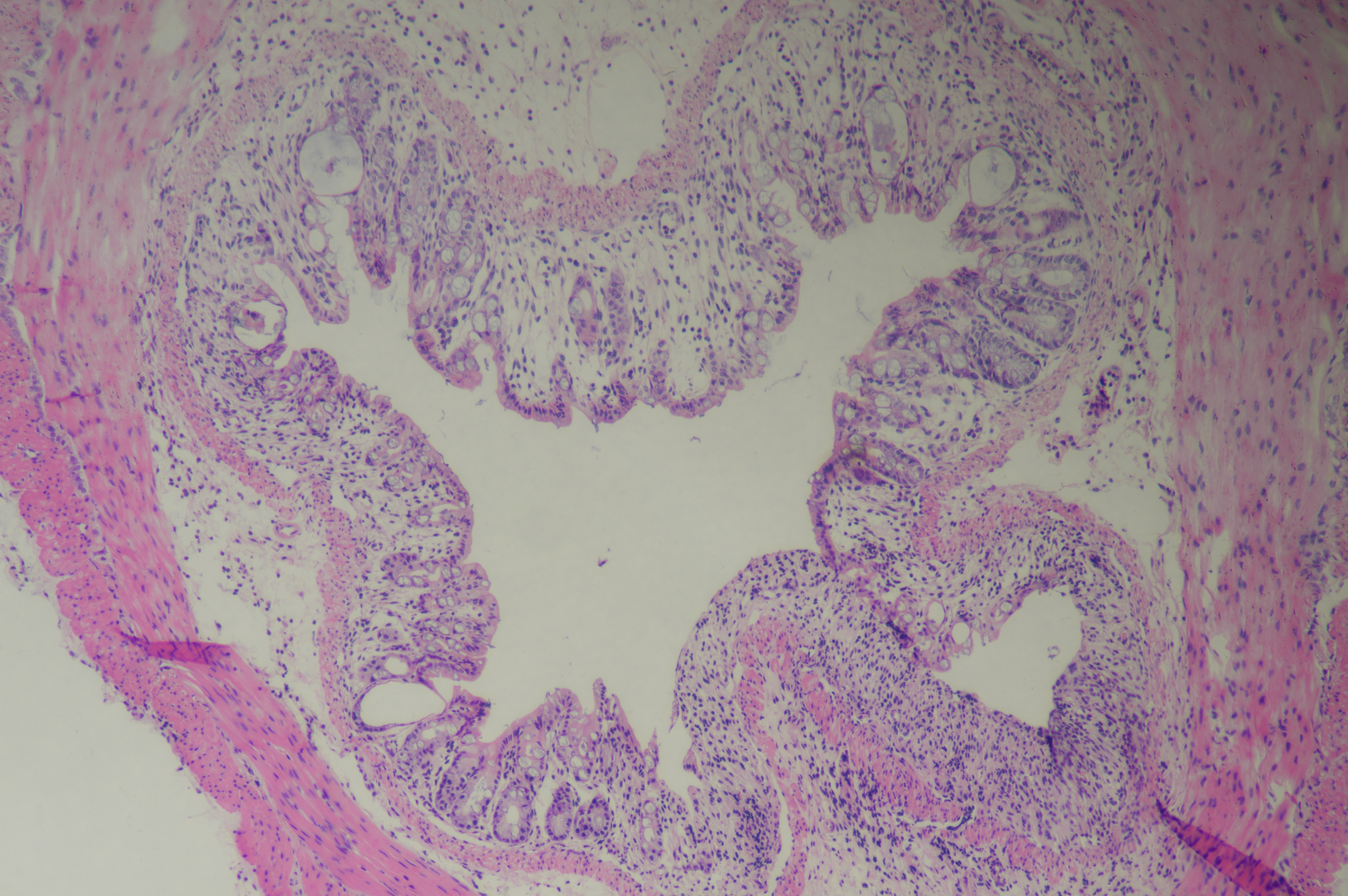

Supplement: Supplementary file 14 — Source Data for Figure 6 [file EMMM-15-e17815-s001.zip › Figure_6/6G/WT-DSS-COL/HE_image.tif]

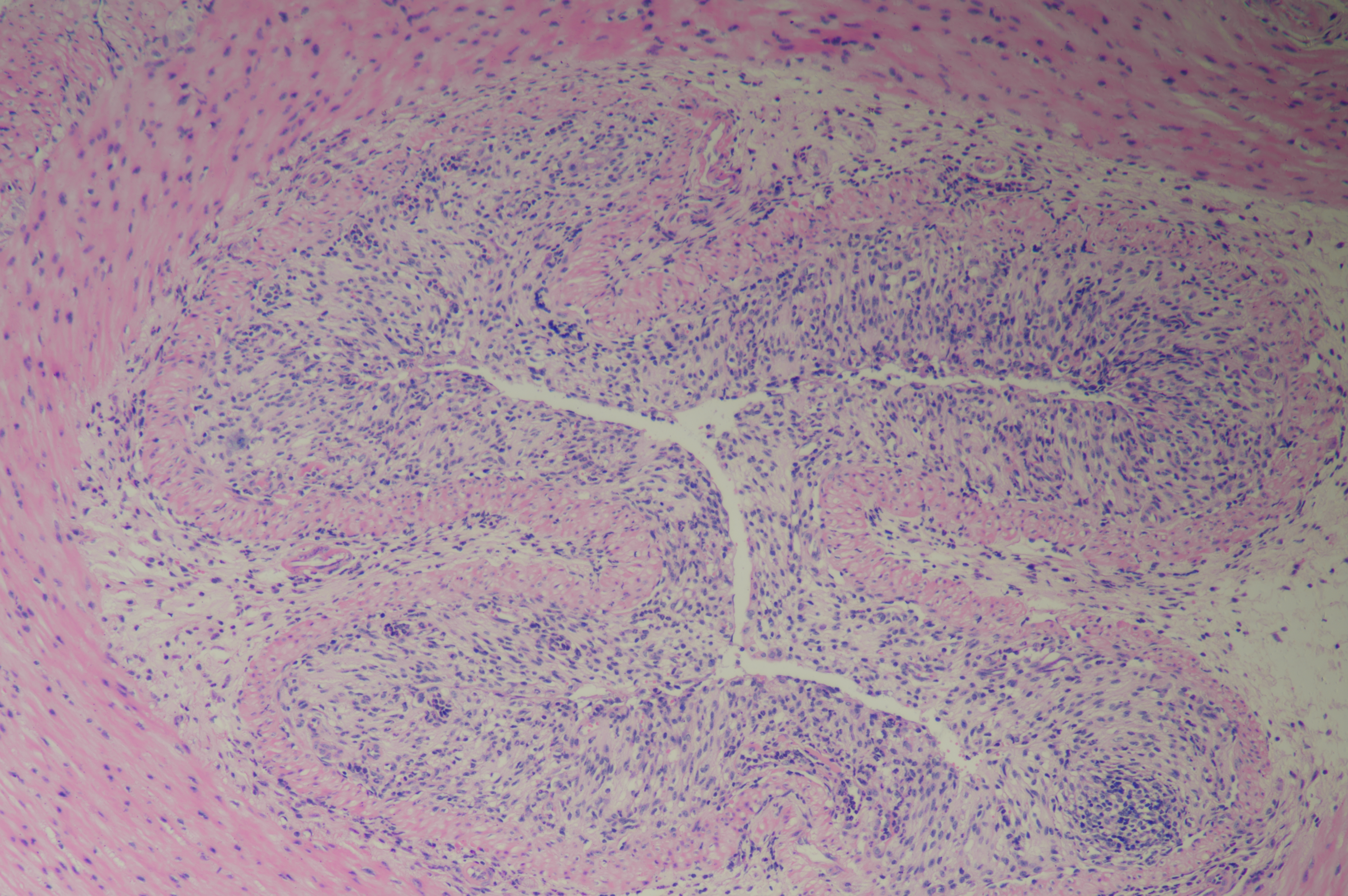

Supplement: Supplementary file 14 — Source Data for Figure 6 [file EMMM-15-e17815-s001.zip › Figure_6/6G/WT-DSS-Vehicle/HE_image.tif]

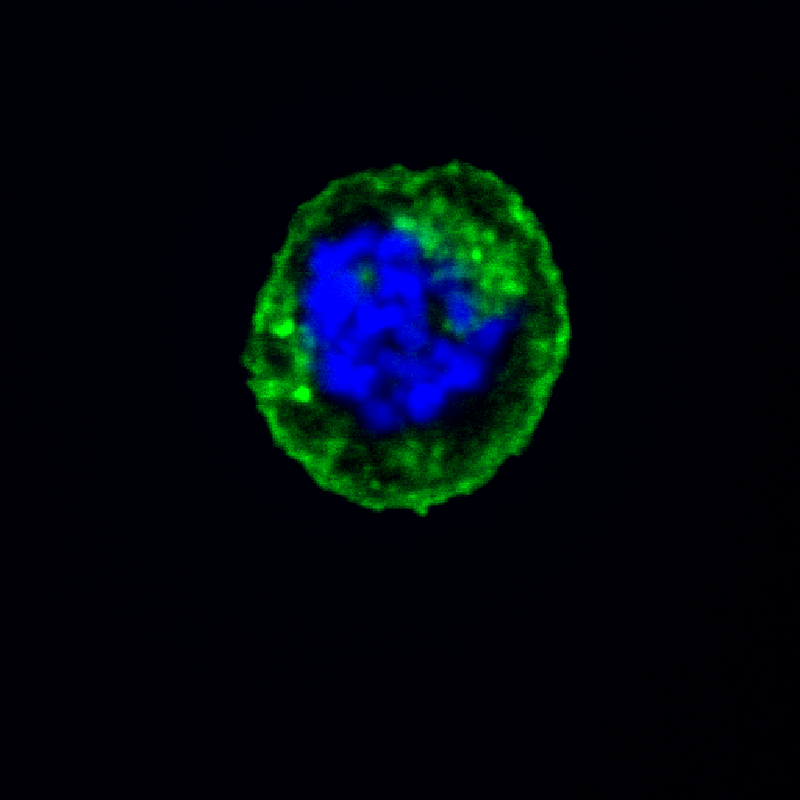

Supplement: Supplementary file 15 — Source Data for Figure 7 [file EMMM-15-e17815-s008.zip › Figure_7/7E/COL/Fluor._micro-GFP-FPR2.tif]

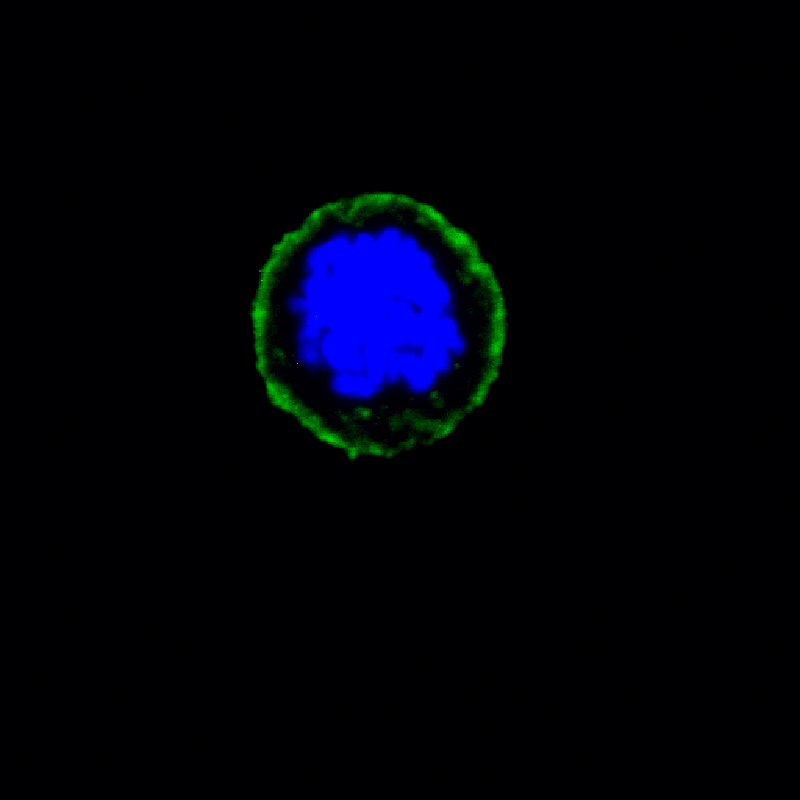

Supplement: Supplementary file 15 — Source Data for Figure 7 [file EMMM-15-e17815-s008.zip › Figure_7/7E/CTRL/Fluor._Micro-GFP-FPR2.tif]

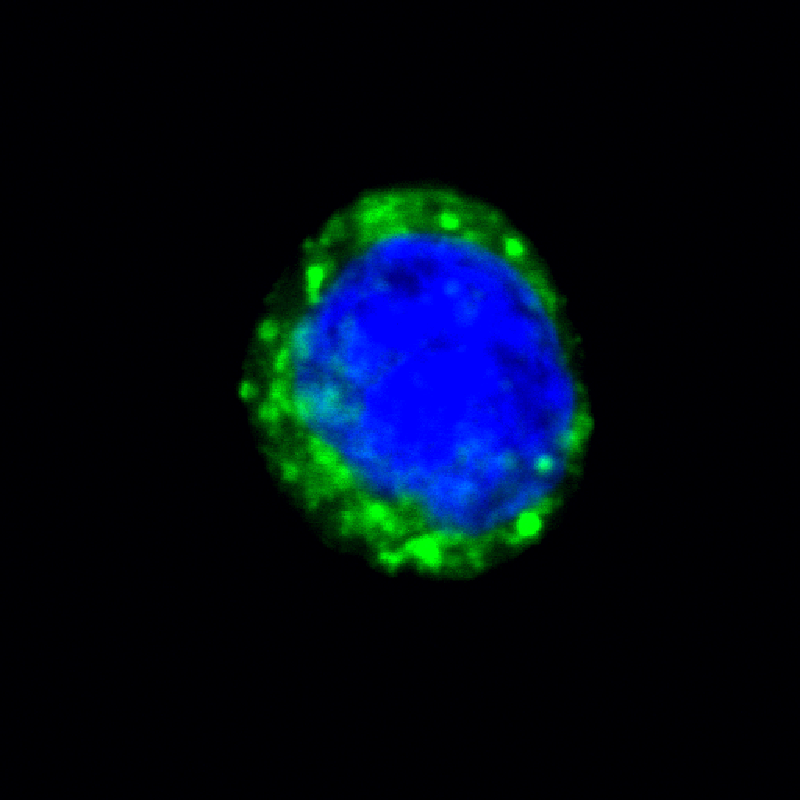

Supplement: Supplementary file 15 — Source Data for Figure 7 [file EMMM-15-e17815-s008.zip › Figure_7/7E/W-pep/Fluro._Micro-GFP-FPR2.tif]

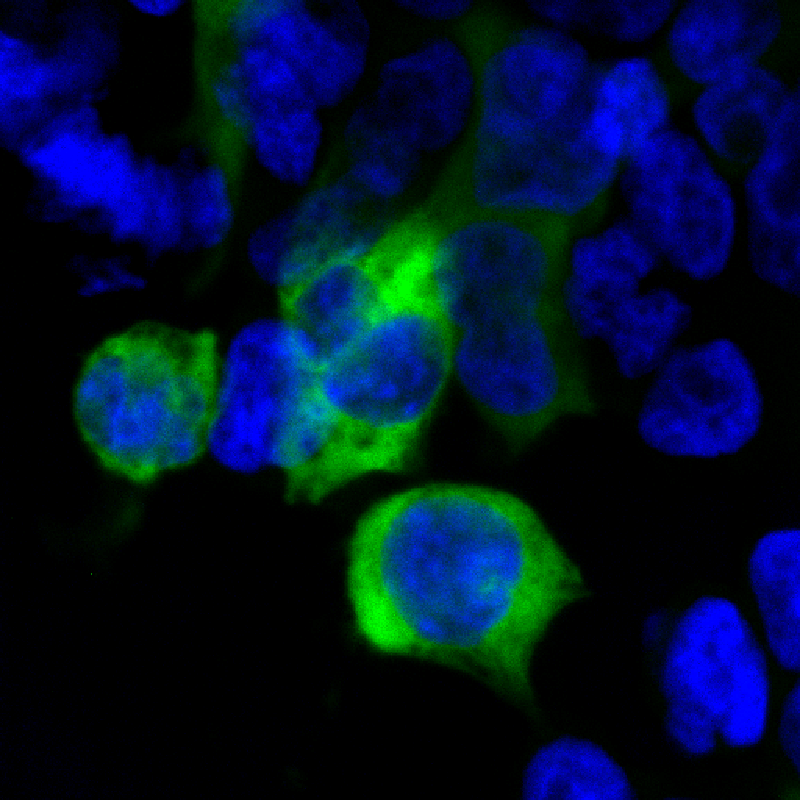

Supplement: Supplementary file 15 — Source Data for Figure 7 [file EMMM-15-e17815-s008.zip › Figure_7/7G/GFP-b-ARR2-COL1/gfp-arrestin-col1.tif]

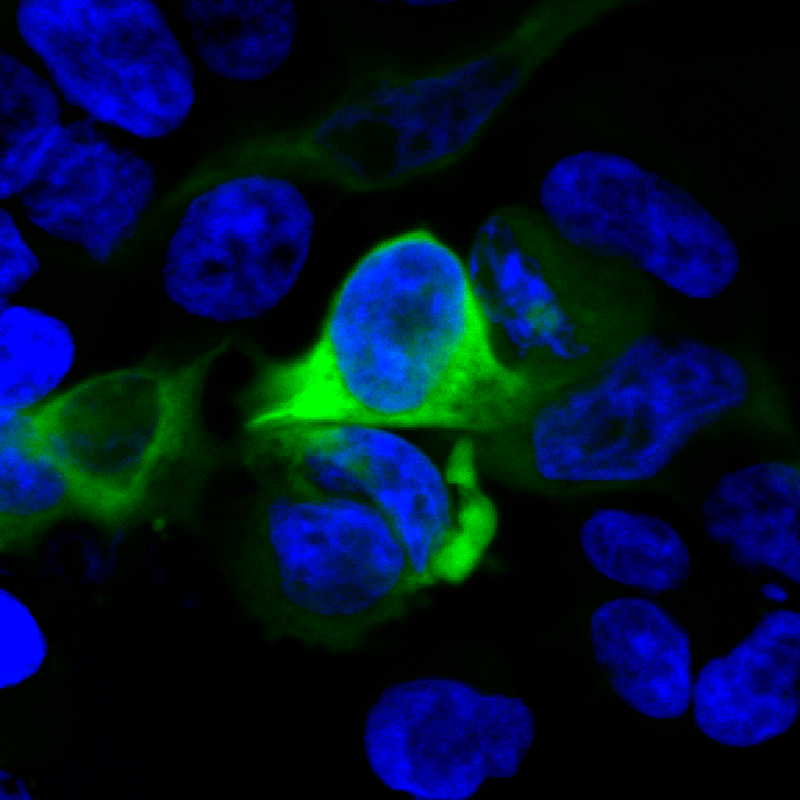

Supplement: Supplementary file 15 — Source Data for Figure 7 [file EMMM-15-e17815-s008.zip › Figure_7/7G/GFP-b-ARR2-COL10/gfp-arrestin-col10.tif]

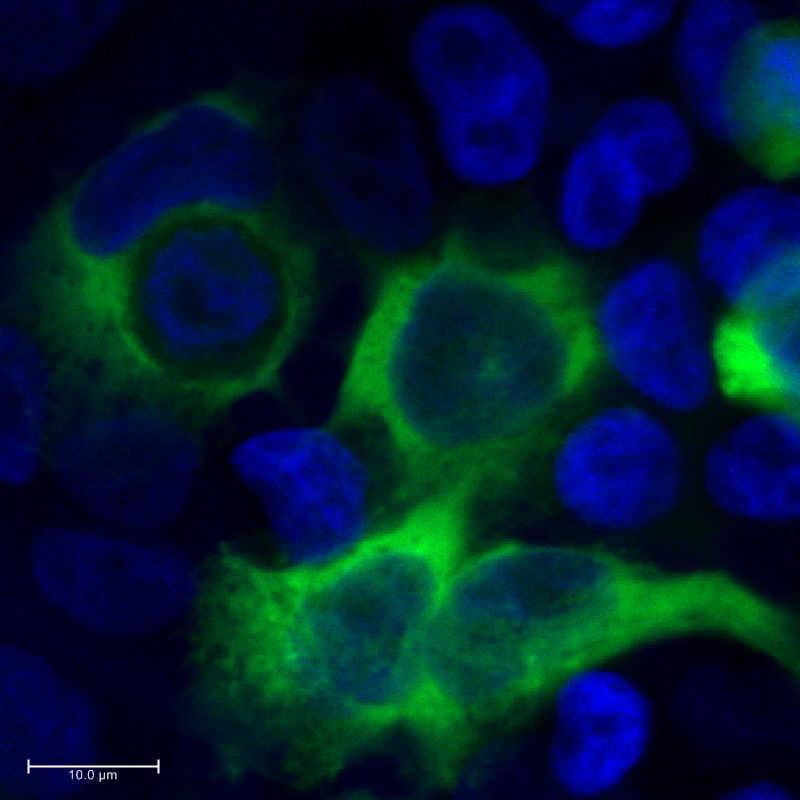

Supplement: Supplementary file 15 — Source Data for Figure 7 [file EMMM-15-e17815-s008.zip › Figure_7/7G/GFP-b-ARR2-CTRL/Arr2-GfP.tif]

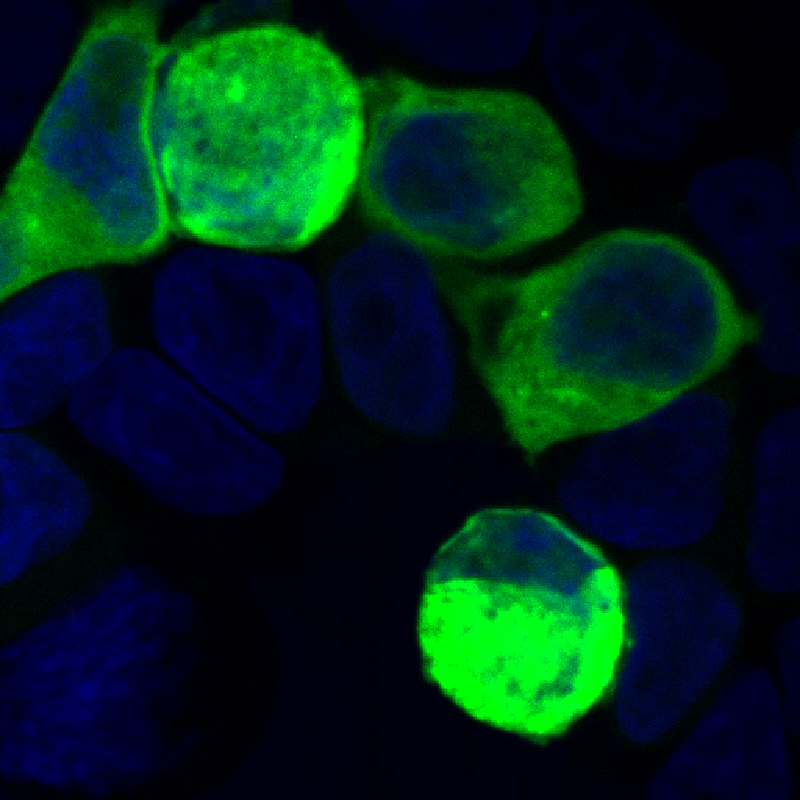

Supplement: Supplementary file 15 — Source Data for Figure 7 [file EMMM-15-e17815-s008.zip › Figure_7/7G/GFP-b-ARR2-FPR2-COL1/gfp-arr_fpr_col1.tif]

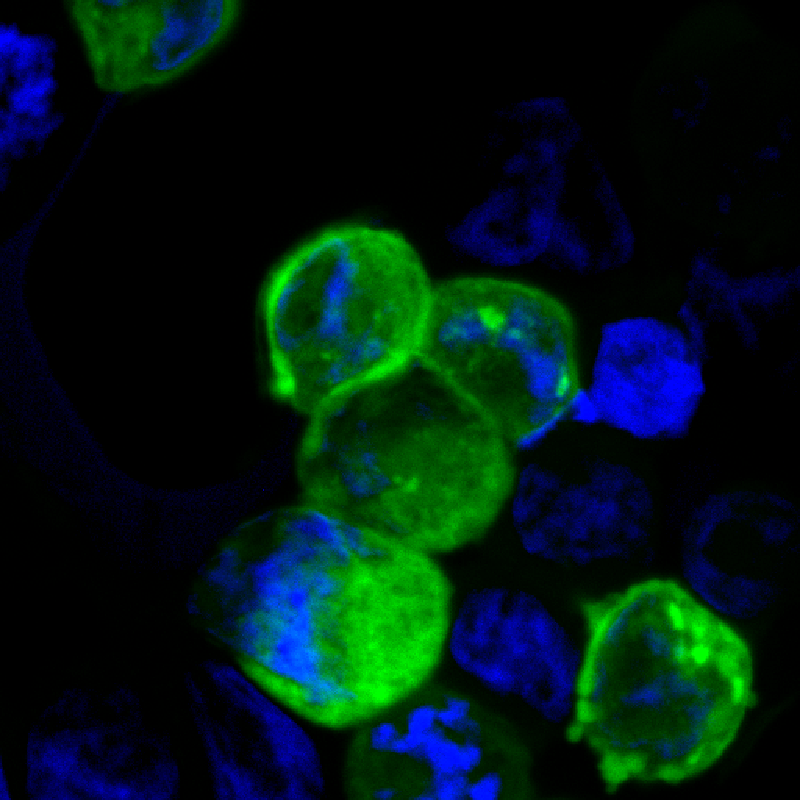

Supplement: Supplementary file 15 — Source Data for Figure 7 [file EMMM-15-e17815-s008.zip › Figure_7/7G/GFP-b-ARR2-FPR2-COL10/gfp-arr_fpr_col10.tif]

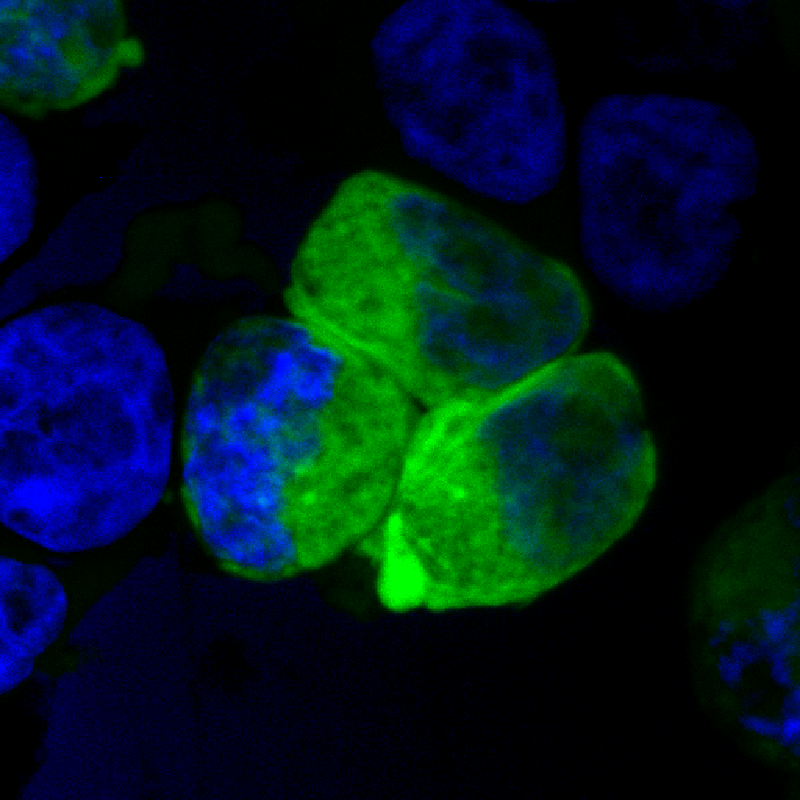

Supplement: Supplementary file 15 — Source Data for Figure 7 [file EMMM-15-e17815-s008.zip › Figure_7/7G/GFP-b-ARR2-FPR2-CTRL/gfp-arr_fpr_ctr.tif]

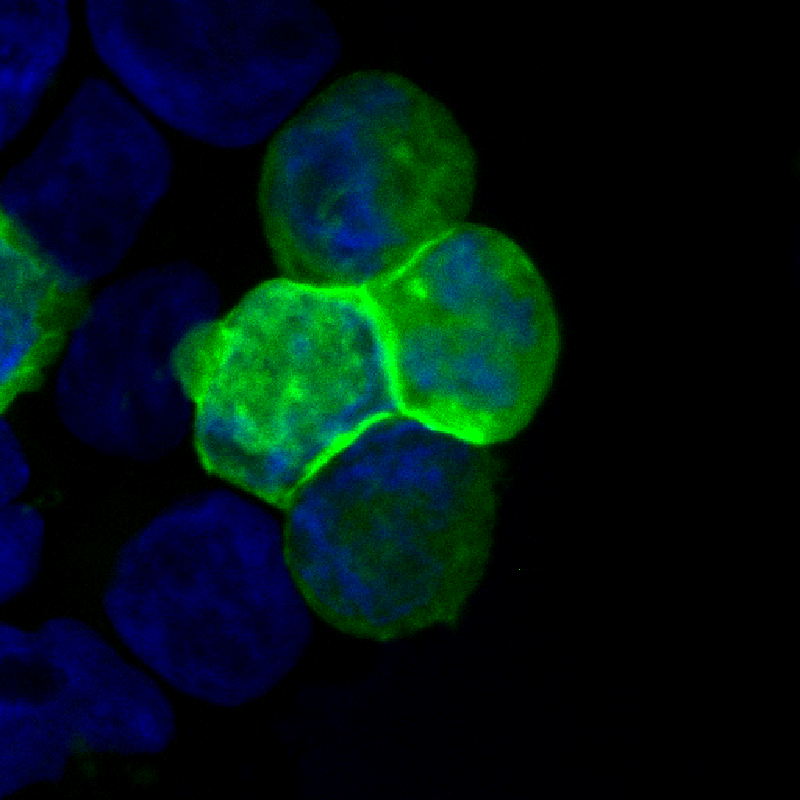

Supplement: Supplementary file 15 — Source Data for Figure 7 [file EMMM-15-e17815-s008.zip › Figure_7/7G/GFP-b-ARR2-FPR2-W-pep/gfp-arr_fpr_wp.tif]

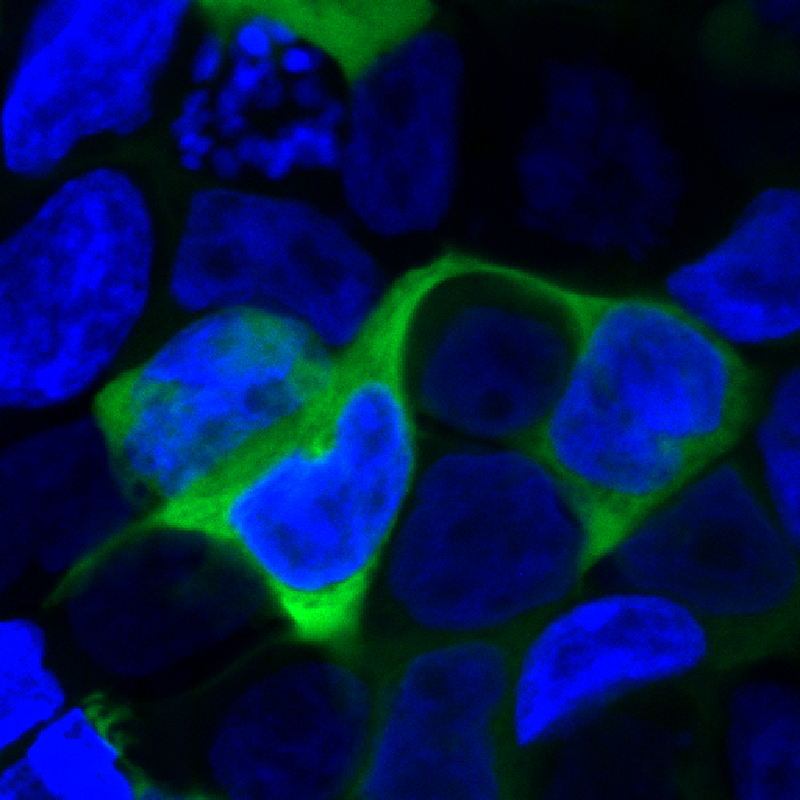

Supplement: Supplementary file 15 — Source Data for Figure 7 [file EMMM-15-e17815-s008.zip › Figure_7/7G/GFP-b-ARR2-W-pep/GFP-arrestin-w-p.tif]

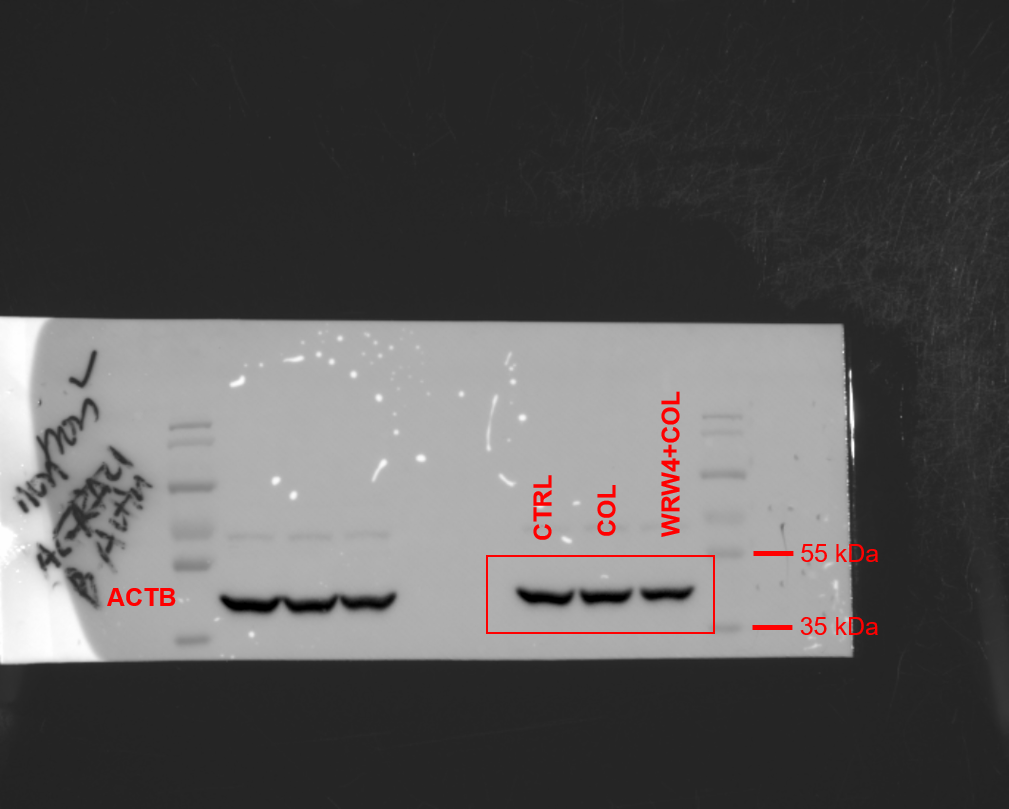

Supplement: Supplementary file 15 — Source Data for Figure 7 [file EMMM-15-e17815-s008.zip › Figure_7/7I/ACTB/ACTB.tif]
