# Supplementary figures and images for: Enhancement of efferocytosis through biased FPR2 signaling attenuates intestinal inflammation (part 2 of 2)
Source: EMBO Mol Med. 2023 Nov 22;15(12):e17815. doi: 10.15252/emmm.202317815 (PMC10701612; doi:10.15252/emmm.202317815)

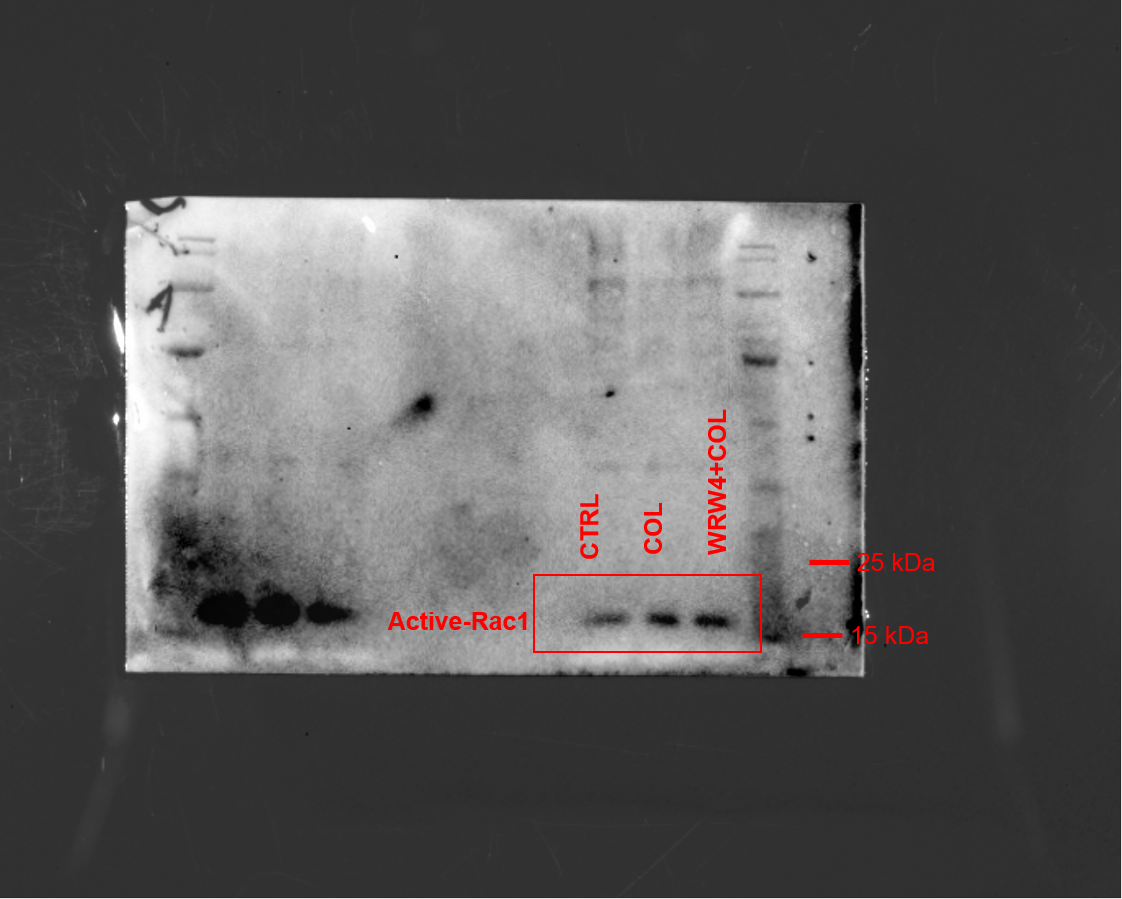

Supplement: Supplementary file 15 — Source Data for Figure 7 [file EMMM-15-e17815-s008.zip › Figure_7/7I/active-RAC1/westernblot-active-Rac1.tif]

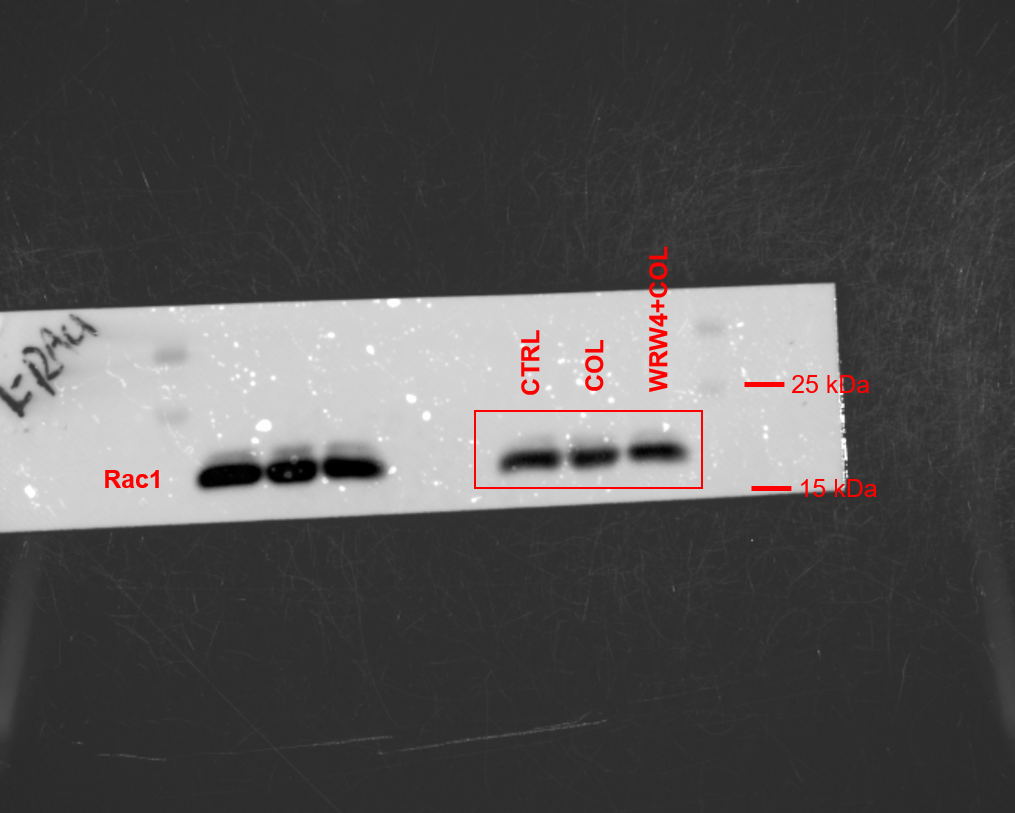

Supplement: Supplementary file 15 — Source Data for Figure 7 [file EMMM-15-e17815-s008.zip › Figure_7/7I/RAC1/westernblot-rac1.tif]

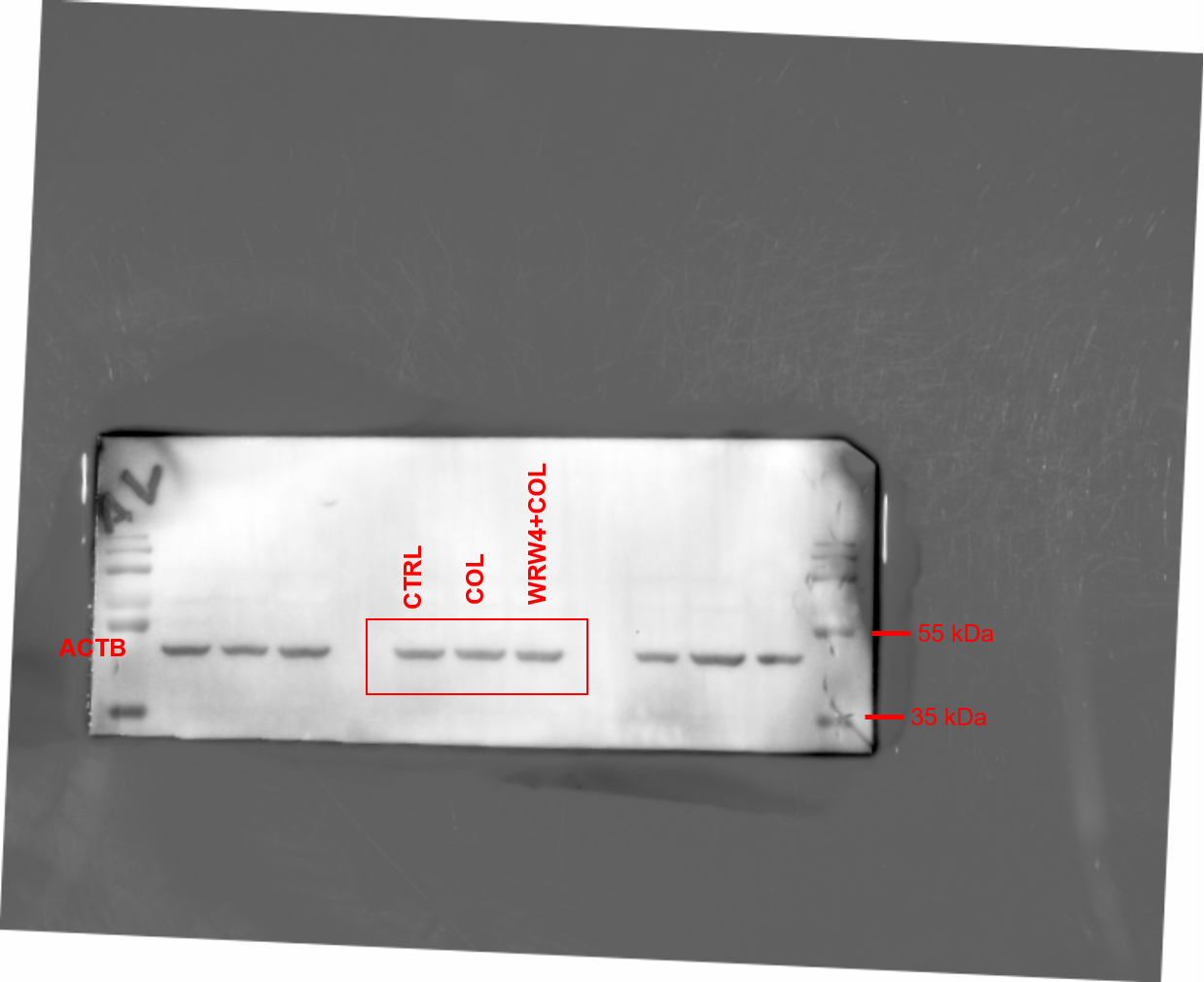

Supplement: Supplementary file 15 — Source Data for Figure 7 [file EMMM-15-e17815-s008.zip › Figure_7/7J/ACTB/westernblot-ACTB.tif]

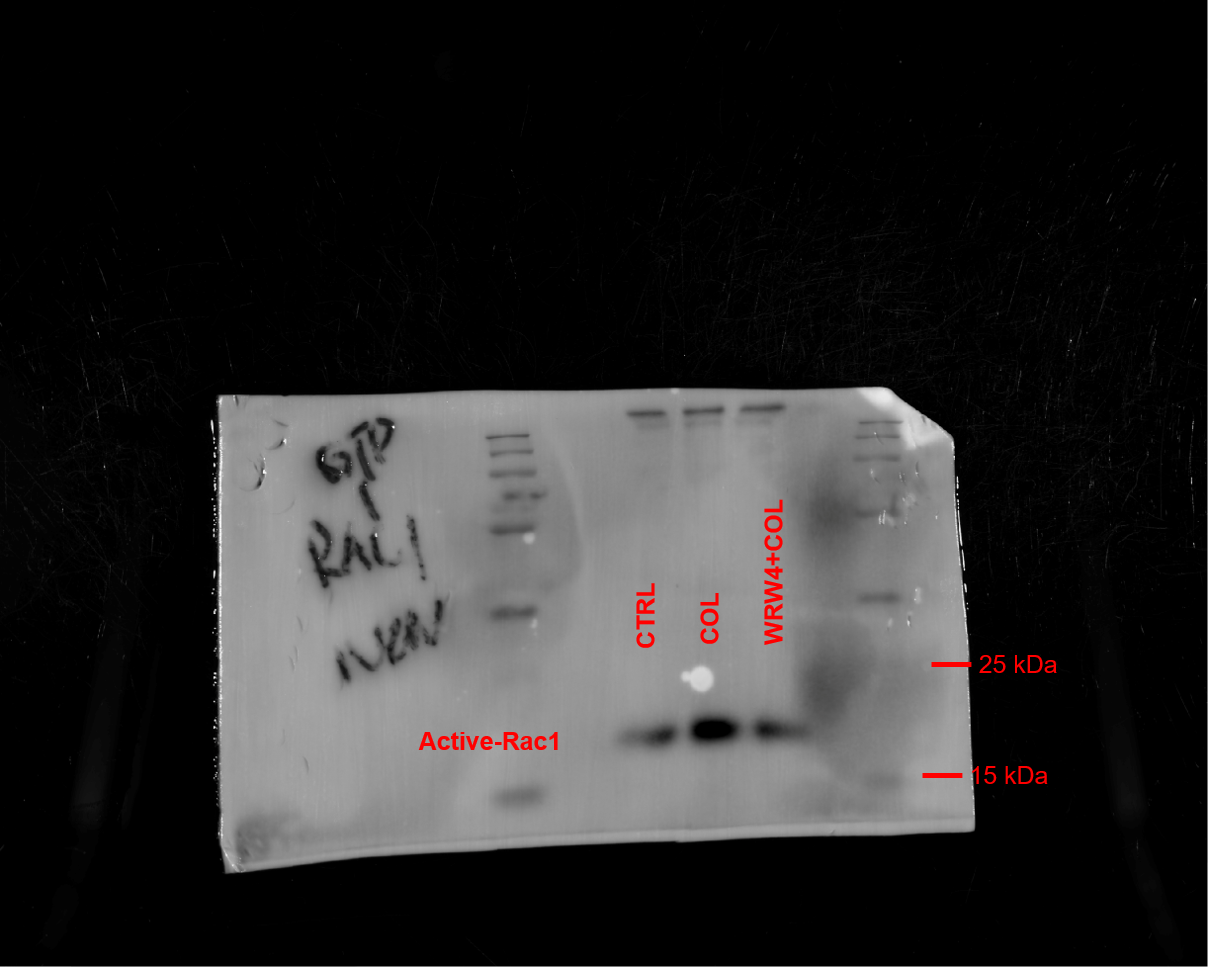

Supplement: Supplementary file 15 — Source Data for Figure 7 [file EMMM-15-e17815-s008.zip › Figure_7/7J/Active-Rac1/Westernblto-GTP-RAC1.tif]

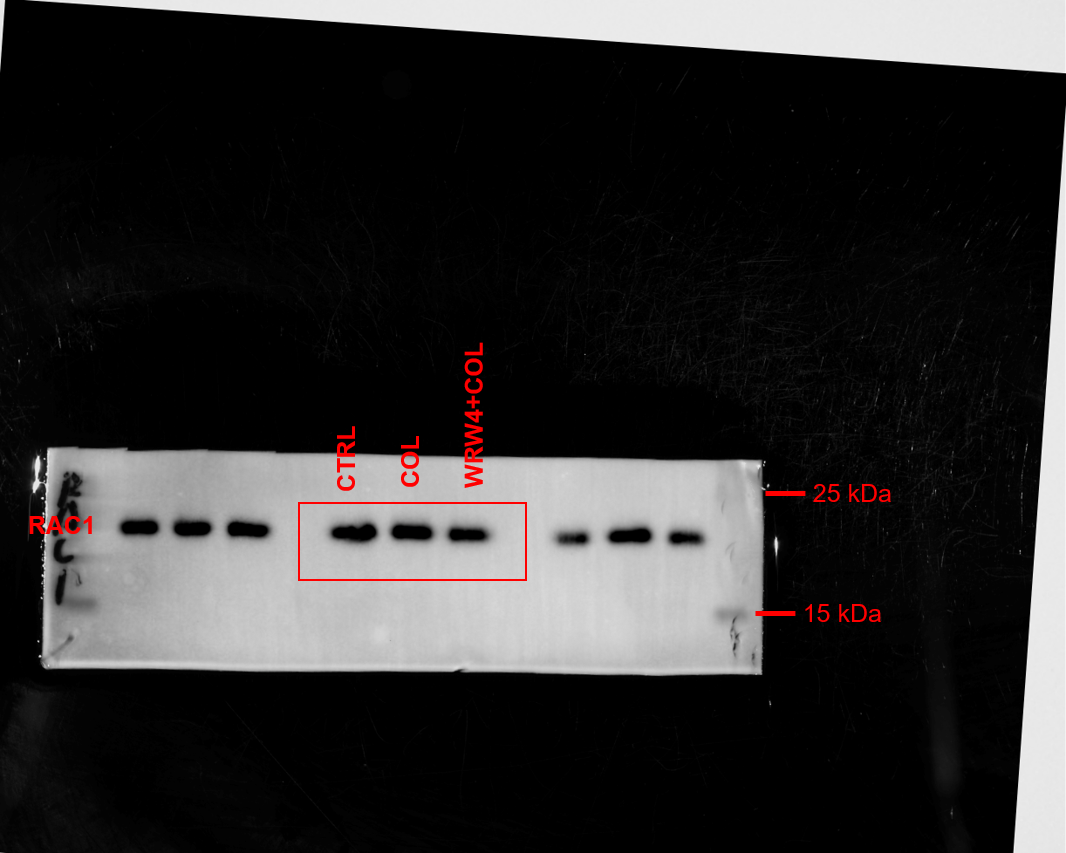

Supplement: Supplementary file 15 — Source Data for Figure 7 [file EMMM-15-e17815-s008.zip › Figure_7/7J/Rac1/Rac1.tif]

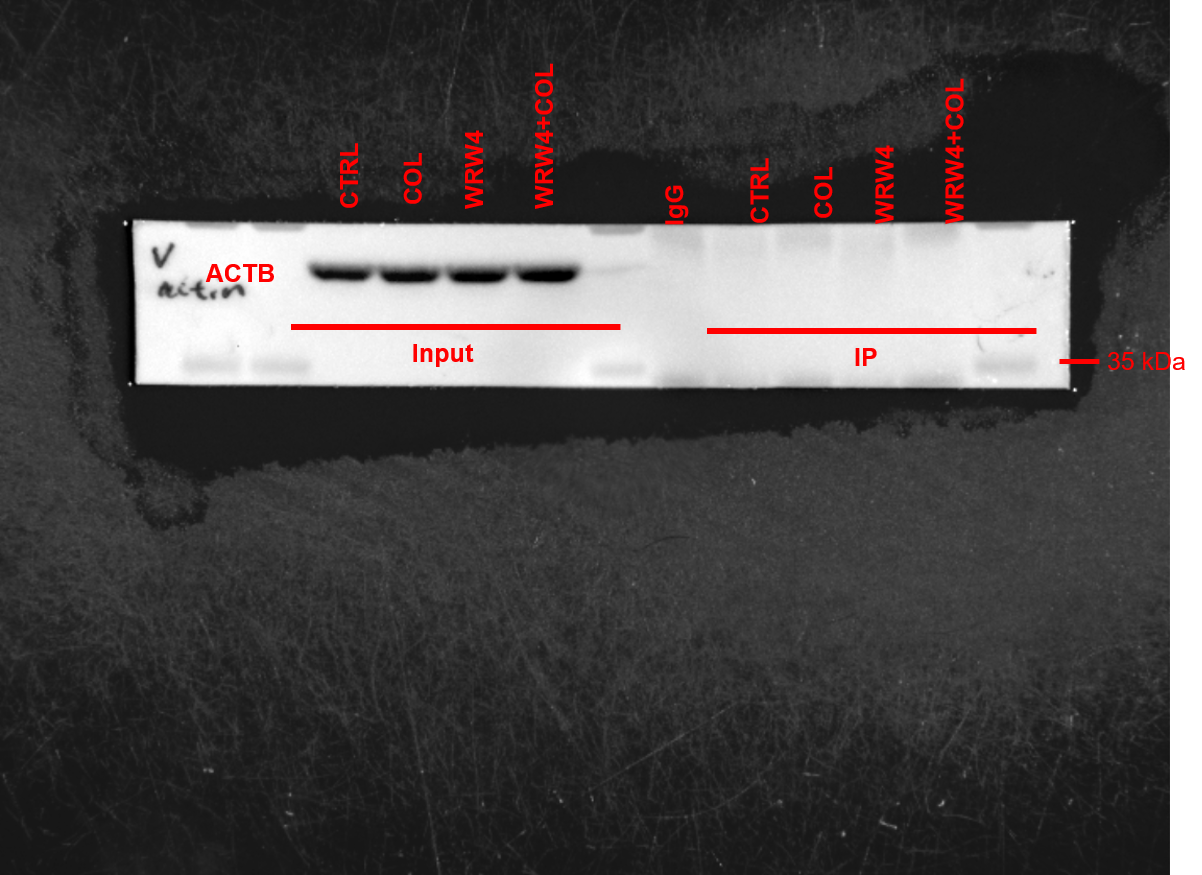

Supplement: Supplementary file 17 — Source Data for Figure 9 [file EMMM-15-e17815-s017.zip › Figure_9/9C/ACTB/westernblot-actin.tif]

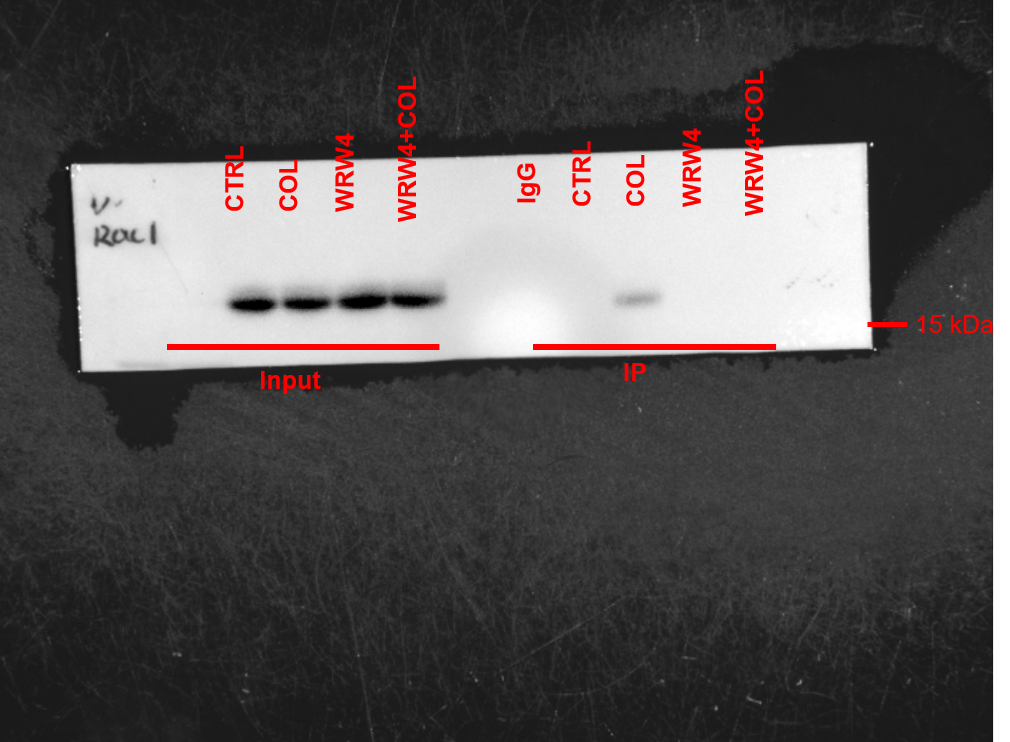

Supplement: Supplementary file 17 — Source Data for Figure 9 [file EMMM-15-e17815-s017.zip › Figure_9/9C/RAC1/westernblot-rac1_long.tif]

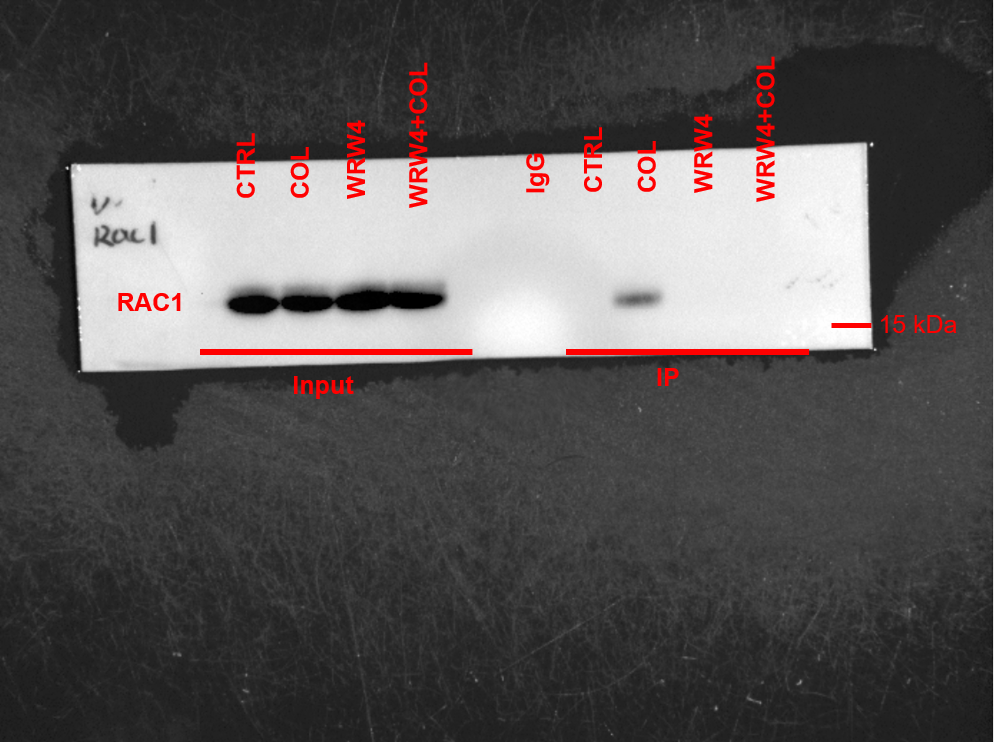

Supplement: Supplementary file 17 — Source Data for Figure 9 [file EMMM-15-e17815-s017.zip › Figure_9/9C/RAC1/westernblot-rac1_short.tif]

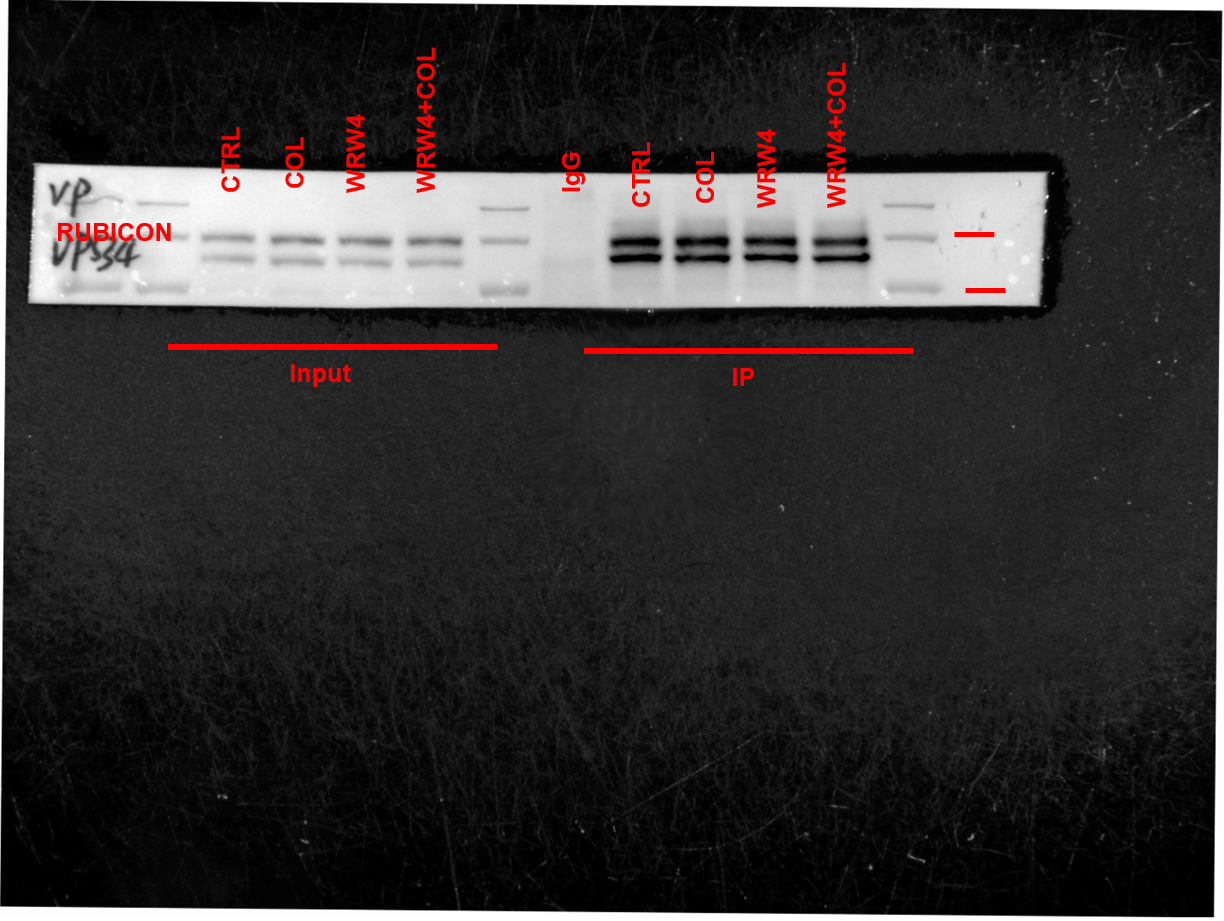

Supplement: Supplementary file 17 — Source Data for Figure 9 [file EMMM-15-e17815-s017.zip › Figure_9/9C/RUBICON/westernblot-rubicon.tif]

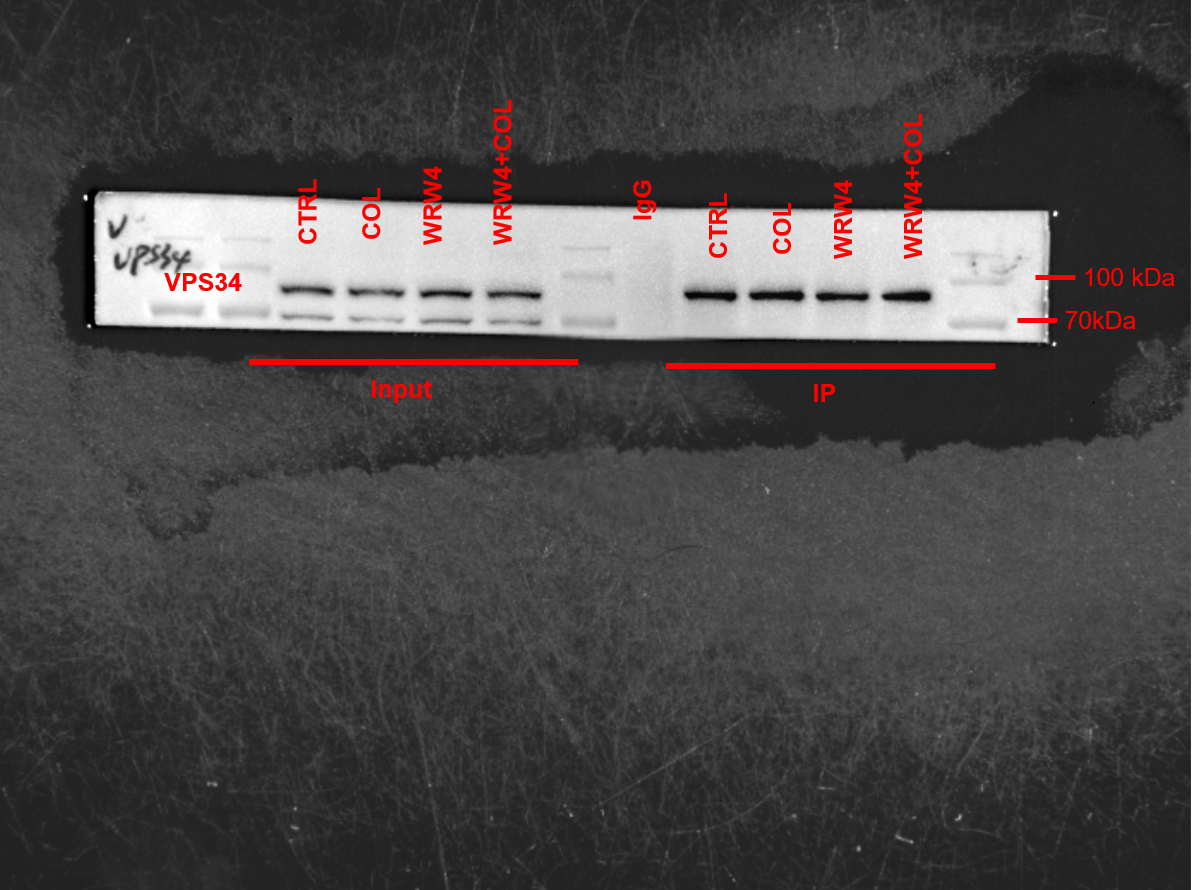

Supplement: Supplementary file 17 — Source Data for Figure 9 [file EMMM-15-e17815-s017.zip › Figure_9/9C/VPS34/westernblot-vps34.tif]

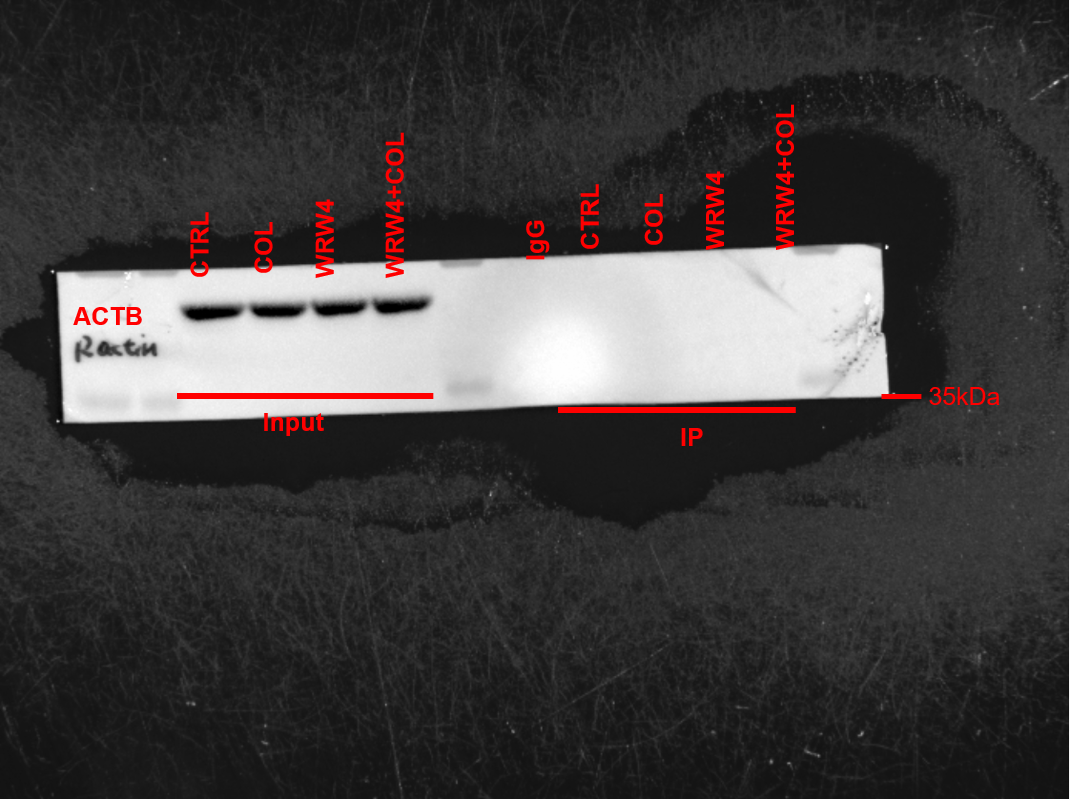

Supplement: Supplementary file 17 — Source Data for Figure 9 [file EMMM-15-e17815-s017.zip › Figure_9/9D/ACTB/ACTB.tif]

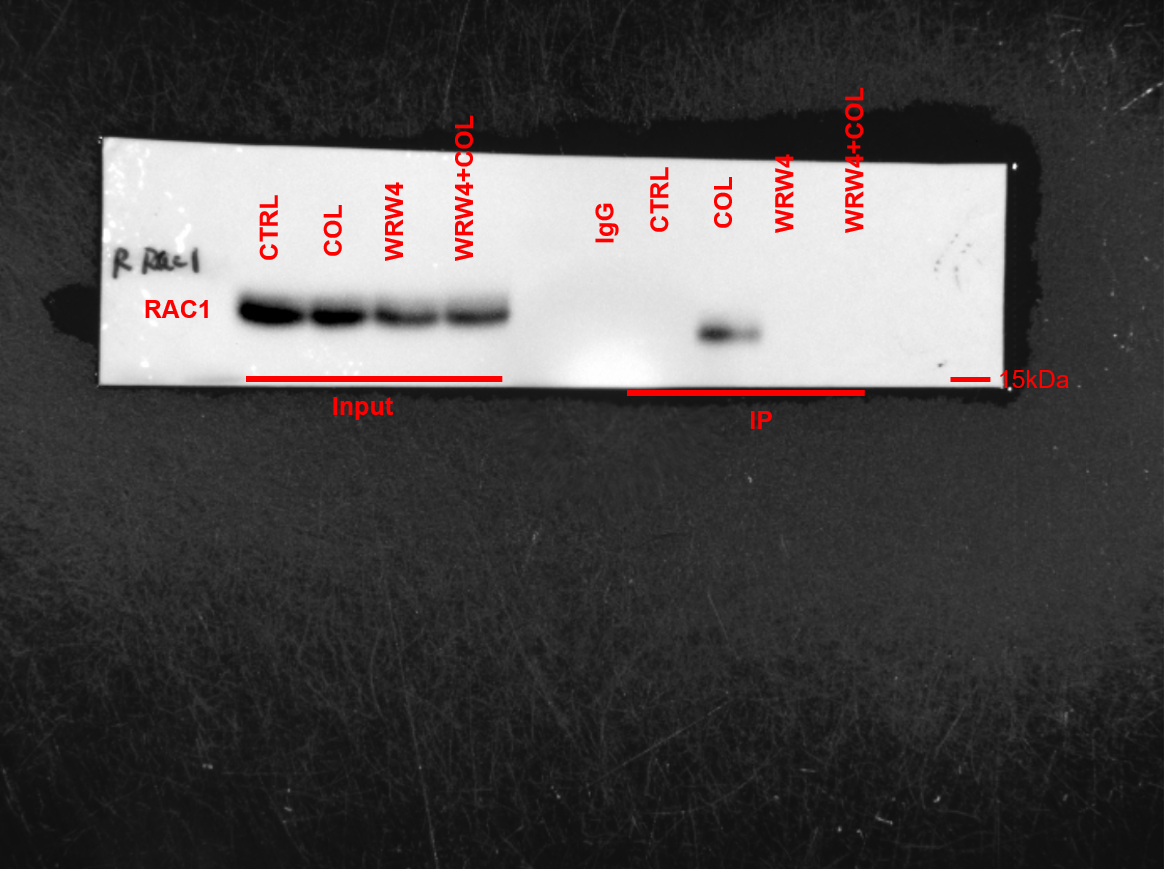

Supplement: Supplementary file 17 — Source Data for Figure 9 [file EMMM-15-e17815-s017.zip › Figure_9/9D/RAC1/RAC1.tif]

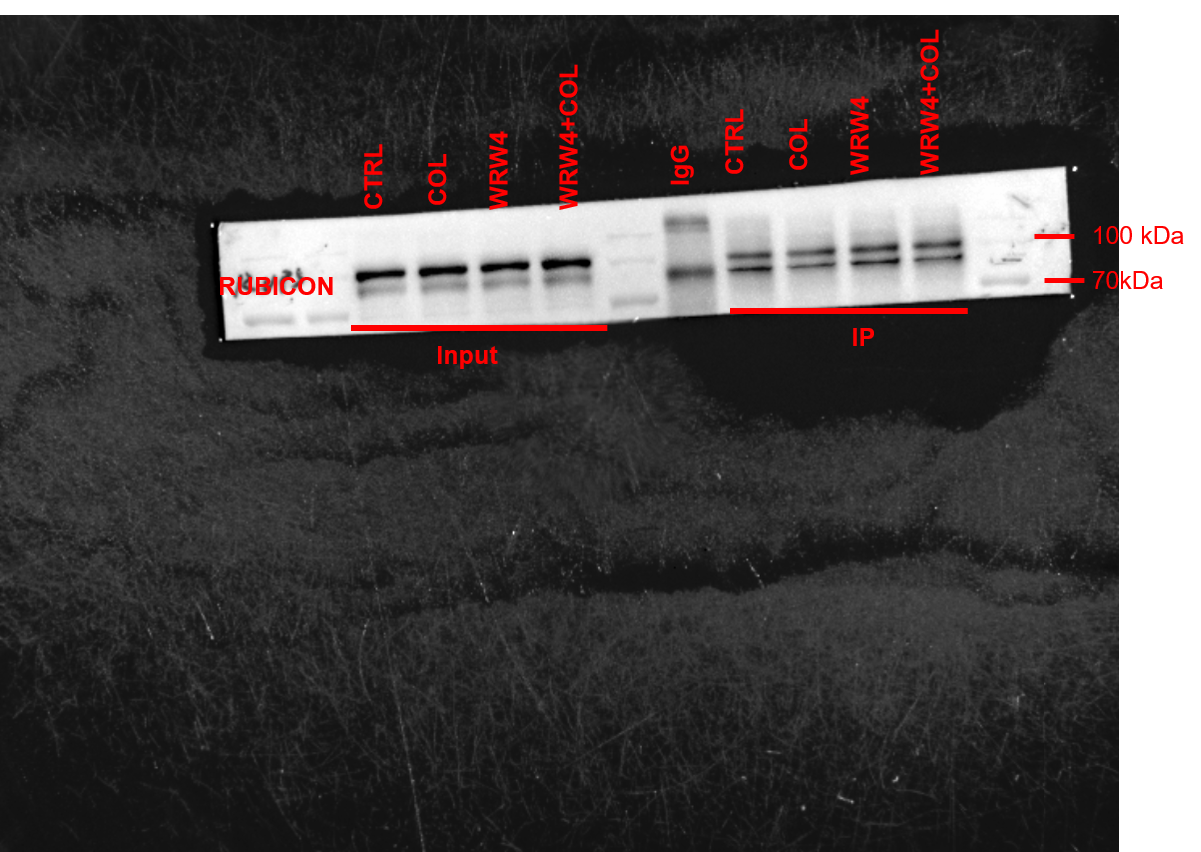

Supplement: Supplementary file 17 — Source Data for Figure 9 [file EMMM-15-e17815-s017.zip › Figure_9/9D/RUBICON/RUBICON.tif]

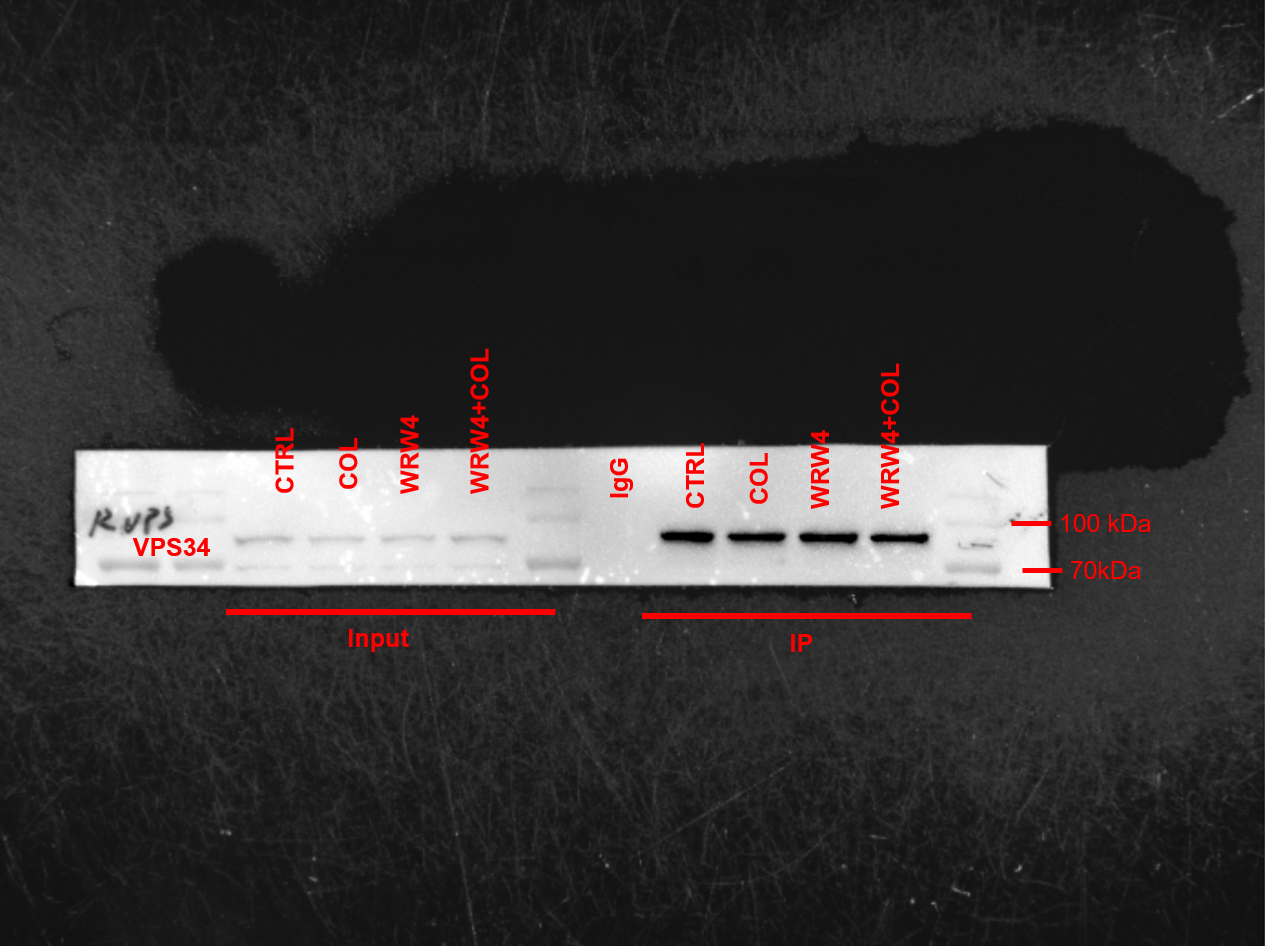

Supplement: Supplementary file 17 — Source Data for Figure 9 [file EMMM-15-e17815-s017.zip › Figure_9/9D/VPS34/westernblot-vps34.tif]

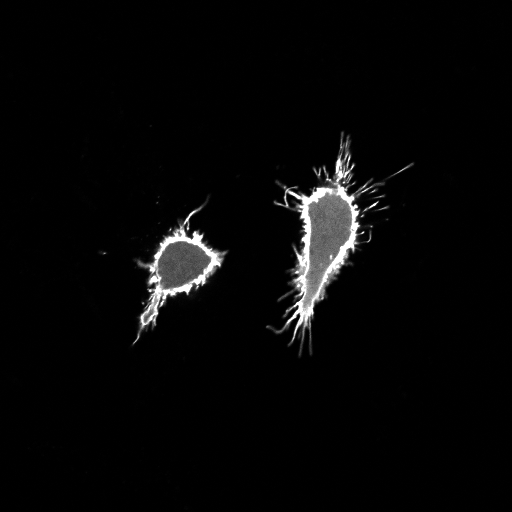

Supplement: Supplementary file 17 — Source Data for Figure 9 [file EMMM-15-e17815-s017.zip › Figure_9/9E/COL/fluor.micro.pip2.tif]

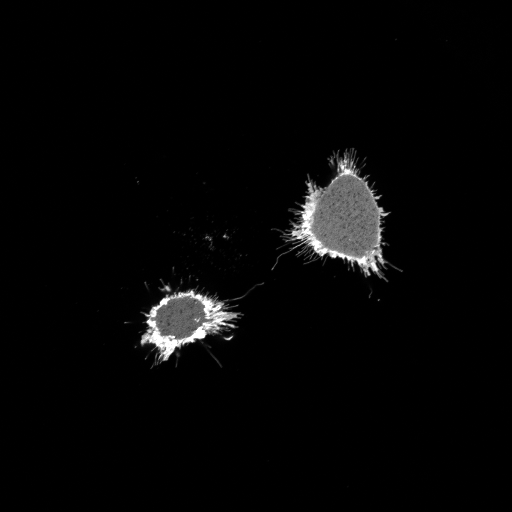

Supplement: Supplementary file 17 — Source Data for Figure 9 [file EMMM-15-e17815-s017.zip › Figure_9/9E/COL+WRW4/fluor.micro-pip2.tif]

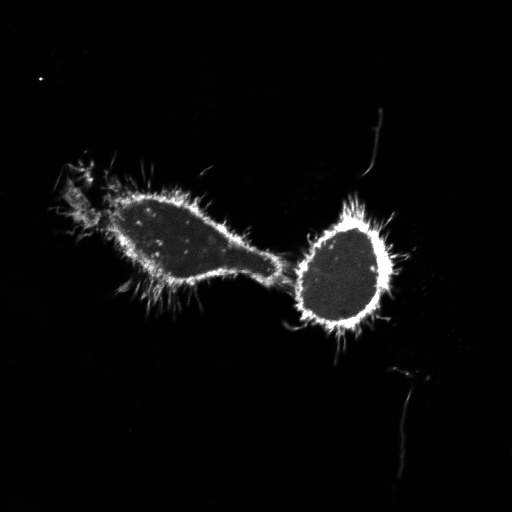

Supplement: Supplementary file 17 — Source Data for Figure 9 [file EMMM-15-e17815-s017.zip › Figure_9/9E/CTRL/fluor.micro.pip2.tif]

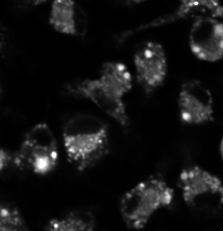

Supplement: Supplementary file 17 — Source Data for Figure 9 [file EMMM-15-e17815-s017.zip › Figure_9/9H/COL/fluro.micro.gfp-lc3-lysotracker.tif]

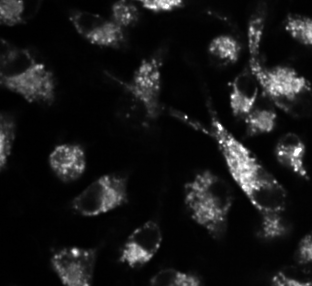

Supplement: Supplementary file 17 — Source Data for Figure 9 [file EMMM-15-e17815-s017.zip › Figure_9/9H/COL+WRW4/fluro.micro.gfp-lc3-lysotracker.tif]

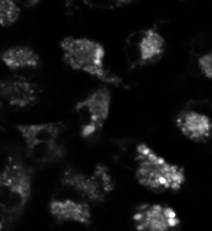

Supplement: Supplementary file 17 — Source Data for Figure 9 [file EMMM-15-e17815-s017.zip › Figure_9/9H/CTRL/fluor.micro.-gfp-lc3_plus_lysotr.tif]
