# Supplementary material for: Enhancement of efferocytosis through biased FPR2 signaling attenuates intestinal inflammation
Source: EMBO Mol Med. 2023 Nov 22;15(12):e17815. doi: 10.15252/emmm.202317815 (PMC10701612; doi:10.15252/emmm.202317815)
Supplement: Supplementary file 18 [file EMMM-15-e17815-s016.pdf]

EMBO Molecular Medicine

Enhancement of efferocytosis through biased FPR2 signaling attenuates intestinal inflammation

Jia-Hong Lu, Richard Ye, Ming-Yue Wu, Yun-Jun Ge, Er-Jin Wang, Qi-Wen Liao, Zheng-Yu Ren, Yang Yu, Guo Zhu, Chun-Ping Liu, Huanxing Su, Han-Ming Shen, Ye Chen, Lei Wang, Jin Chai, Yi-Tao Wang, Min Li, Zhaoxiang Bian, and Meng-Ni Zhang
DOI: 10.15252/emmm.202317815

Corresponding authors: Jia-Hong Lu (jiahonglu@um.edu.mo) , Richard Ye (richardye@cuhk.edu.cn)

Review Timeline:

Submission Date:	19th Apr 23
Editorial Decision:	19th May 23
Revision Received:	19th Sep 23
Editorial Decision:	29th Sep 23
Revision Received:	20th Oct 23
Accepted:	24th Oct 23

Editor: Zeljko Durdevic

Transaction Report:

(Note: With the exception of the correction of typographical or spelling errors that could be a source of ambiguity, letters and reports are not edited. Depending on transfer agreements, referee reports obtained elsewhere may or may not be included in this compilation. Referee reports are anonymous unless the Referee chooses to sign their reports.)

19th May 2023

Dear Dr. Lu,

Thank you for the submission of your manuscript to EMBO Molecular Medicine. We have now received feedback from the three reviewers who agreed to evaluate your manuscript. As you will see from the reports below, all referees recognize potential interest of the study, but also raise important concerns that should be addressed in a major revision. If you would like to discuss further the points raised by the referees, I am available to do so via email or video. Let me know if you are interested in this option.

Further consideration of a revision that addresses reviewers' concerns in full will entail a second round of review. EMBO Molecular Medicine encourages a single round of revision only and therefore, acceptance or rejection of the manuscript will depend on the completeness of your responses included in the next, final version of the manuscript. For this reason, and to save you from any frustrations in the end, I would strongly advise against returning an incomplete revision.

We would welcome the submission of a revised version within three months for further consideration. Please let us know if you require longer to complete the revision.

I look forward to receiving your revised manuscript.

Yours sincerely,

Zeljko Durdevic

Zeljko Durdevic
Editor
EMBO Molecular Medicine

*****

When submitting your revised manuscript, please carefully review the instructions that follow below. We perform an initial quality control of all revised manuscripts before re-review; failure to include requested items will delay the evaluation of your revision.

We require:

1) A .docx formatted version of the manuscript text (including legends for main figures, EV figures and tables). Please make sure that the changes are highlighted to be clearly visible.

2) Individual production quality figure files as .eps, .tif, .jpg (one file per figure). For guidance, download the 'Figure Guide PDF': (https://www.embopress.org/page/journal/17574684/authorguide#figureformat).

3) A .docx formatted letter INCLUDING the reviewers' reports and your detailed point-by-point responses to their comments. As part of the EMBO Press transparent editorial process, the point-by-point response is part of the Review Process File (RPF), which will be published alongside your paper.

4) A complete author checklist, which you can download from our author guidelines (https://www.embopress.org/page/journal/17574684/authorguide#submissionofrevisions). Please insert information in the checklist that is also reflected in the manuscript. The completed author checklist will also be part of the RPF.

5) Please note that all corresponding authors are required to supply an ORCID ID for their name upon submission of a revised manuscript.

6) It is mandatory to include a 'Data Availability' section after the Materials and Methods. Before submitting your revision, primary

datasets produced in this study need to be deposited in an appropriate public database, and the accession numbers and database listed under 'Data Availability'. Please remember to provide a reviewer password if the datasets are not yet public (see https://www.embopress.org/page/journal/17574684/authorguide#dataavailability).

In case you have no data that requires deposition in a public database, please state so in this section. Note that the Data Availability Section is restricted to new primary data that are part of this study.

7) For data quantification: please specify the name of the statistical test used to generate error bars and P values, the number (n) of independent experiments (specify technical or biological replicates) underlying each data point and the test used to calculate p-values in each figure legend. The figure legends should contain a basic description of n, P and the test applied. Graphs must include a description of the bars and the error bars (s.d., s.e.m.). See also 'Figure Legend' guidelines: https://www.embopress.org/page/journal/17574684/authorguide#figureformat

8) At EMBO Press we ask authors to provide source data for the main manuscript figures. Our source data coordinator will contact you to discuss which figure panels we would need source data for and will also provide you with helpful tips on how to upload and organize the files.

9) Our journal encourages inclusion of *data citations in the reference list* to directly cite datasets that were re-used and obtained from public databases. Data citations in the article text are distinct from normal bibliographical citations and should directly link to the database records from which the data can be accessed. In the main text, data citations are formatted as follows: "Data ref: Smith et al, 2001" or "Data ref: NCBI Sequence Read Archive PRJNA342805, 2017". In the Reference list, data citations must be labeled with "[DATASET]". A data reference must provide the database name, accession number/identifiers and a resolvable link to the landing page from which the data can be accessed at the end of the reference. Further instructions are available at .

10) We replaced Supplementary Information with Expanded View (EV) Figures and Tables that are collapsible/expandable online. A maximum of 5 EV Figures can be typeset. EV Figures should be cited as 'Figure EV1, Figure EV2" etc... in the text and their respective legends should be included in the main text after the legends of regular figures.

- For the figures that you do NOT wish to display as Expanded View figures, they should be bundled together with their legends in a single PDF file called *Appendix*, which should start with a short Table of Content. Appendix figures should be referred to in the main text as: "Appendix Figure S1, Appendix Figure S2" etc.

- Additional Tables/Datasets should be labeled and referred to as Table EV1, Dataset EV1, etc. Legends have to be provided in a separate tab in case of .xls files. Alternatively, the legend can be supplied as a separate text file (README) and zipped together with the Table/Dataset file.

See detailed instructions here:

.

11) The paper explained: EMBO Molecular Medicine articles are accompanied by a summary of the articles to emphasize the major findings in the paper and their medical implications for the non-specialist reader. Please provide a draft summary of your article highlighting

- the medical issue you are addressing,

- the results obtained and

- their clinical impact.

This may be edited to ensure that readers understand the significance and context of the research. Please refer to any of our published articles for an example.

12) For more information: There is space at the end of each article to list relevant web links for further consultation by our readers. Could you identify some relevant ones and provide such information as well? Some examples are patient associations, relevant databases, OMIM/proteins/genes links, author's websites, etc...

13) Author contributions: You will be asked to provide CRediT (Contributor Role Taxonomy) terms in the submission system. These replace a narrative author contribution section in the manuscript.

14) A Conflict of Interest statement should be provided in the main text.

15) Every published paper now includes a 'Synopsis' to further enhance discoverability. Synopses are displayed on the journal webpage and are freely accessible to all readers. They include a short stand first (maximum of 300 characters, including space) as well as 2-5 one-sentences bullet points that summarizes the paper. Please write the bullet points to summarize the key NEW findings. They should be designed to be complementary to the abstract - i.e. not repeat the same text. We encourage inclusion of key acronyms and quantitative information (maximum of 30 words / bullet point). Please use the passive voice. Please attach these in a separate file or send them by email, we will incorporate them accordingly.

Please also suggest a striking image or visual abstract to illustrate your article as a PNG file 550 px wide x 300-600 px high.

EMBO Molecular Medicine has a "scooping protection" policy, whereby similar findings that are published by others during review or revision are not a criterion for rejection. Should you decide to submit a revised version, I do ask that you get in touch after three months if you have not completed it, to update us on the status.

Please note: When submitting your revision you will be prompted to enter your funding and payment information. This will allow Wiley to send you a quote for the article processing charge (APC) in case of acceptance. This quote takes into account any reduction or fee waivers that you may be eligible for. Authors do not need to pay any fees before their manuscript is accepted and transferred to the publisher.

EMBO Press participates in many Publish and Read agreements that allow authors to publish Open Access with reduced/no publication charges. Check your eligibility: https://authorservices.wiley.com/author-resources/Journal-Authors/open-access/affiliation-policies-payments/index.html

***** Reviewer's comments *****

Referee #1 (Comments on Novelty/Model System for Author):

This is a really nice report describing the discovery of a natural compound that enhances efferocytosis and improves the outcome of DSS-induced colitis. The investigators show that this is du to effects on FPR2. This is an important discovery and links FPR2 function and efferocytosis.

Referee #1 (Remarks for Author):

The authors report studies investigating the effects of a novel natural product, columbamine (COL), on macrophage efferocytosis and show that COL enhances efferocytosis. COL also improves the outcome of DSS-induced colitis through biased agonism of FPR2, which is an interesting finding. This report would benefit from minor revisions.

Specific Comments

1. The authors show in Figure 1 that several compound enhance macrophage efferocytosis, some to a similar level as columbamine. It would be of interest to explain what those other compounds were and why COL was chosen for further analysis. Are the other active compounds structurally related to columbamine?

2. The Figure 1 legend and the text needs to include the dose of COL used to treat the mice. Was the concentration achieved in vivo similar to that used to treat macrophages in vitro? This should be provided or discussed.

3. The authors show that COL affects macrophage functions. Was there any cytotoxicity associated with this compound?

4. The authors show that COL affects FPR2 responses and suggest it is an FPR2 biased agonist. Does COL also have effects at FPR1? This should be addressed since many FPR agonists hit both receptors.

5. This study was performed on murine cells. However, it is well documented that some FPR ligands that impact mouse cells have no effect on human macrophages, and vice versus. This issue should be addressed or at least a discussion of this issue should be provided to enhance medical implications of this work. Was COL tested with any human macrophages or macrophage cell lines?

Referee #2 (Remarks for Author):

In this manuscript, Wu et al. report on the identification of columbamine (COL) as a biased FPR2 agonist that enhances efferocytosis and the efficacy of COL in attenuating intestinal inflammation in a murine colitis model. COL was first identified as a potential efferocytosis enhancers from a small library of natural products. The authors subsequently showed that COL enhances LC3-associated phagocytosis of mouse BMDMs, and they used a murine colitis model to demonstrate the anti-inflammatory

function of COL. Stemmed from these findings, the authors have identified FPR2 as the molecular target of COL in enhancing efferocytosis and promoting the resolution of inflammation. Furthermore, the authors have conducted docking and mutagenesis studies to investigate the binding mechanism of COL on FPR2 and mechanistic studies to prove the involvement of RAC1 in COL-mediated enhancement of efferocytosis. Overall, this study is of high quality. The identified novel biased FPR2 agonist as well as the mechanism of FPR2-mediated enhancement of efferocytosis may offer new therapeutic opportunities for inflammatory diseases such as colitis. The paper is expected to have a high impact on the research fields of GPCR pharmacology, inflammation, and IBD. However, there are some issues that I believe need to be addressed or at least discussed.

1. The intriguing observation that COL does not induce Ca2+ immobilization raises interesting questions. Previous research (https://doi.org/10.1038/ncomms14232) has shown that another FPR2 agonist, compound 17b, also appears to be biased away from Ca2+. Interestingly, compared to the FPR2 agonist compound 43, which induces strong Ca2+ responses, compound 17b has been shown to exhibit superior anti-inflammatory and cardioprotective effects in cardiomyocytes. To gain a better understanding of the functional role of biased signaling in efferocytosis, it may be worth investigating the W-peptide, which is typically considered a non-biased agonist of FPR2. Testing the W-peptide in efferocytosis could serve as an important control experiment to help establish a link between the biased signaling properties of COL and its functional role in efferocytosis.

2. Related to 1, is the functional role of COL in promoting efferocytosis exclusive to this compound, or can other synthetic FPR2 agonists, such as compound 43 and BMS compounds, also induce efferocytosis as effectively? Understanding the mechanism of action and efficacy of various FPR2 agonists in promoting efferocytosis could lead to the development of novel therapeutic agents.

3. Another study (https://doi.org/10.1021/acsptsci.2c00042) comparing two clinical stage FPR2 agonists, BMS-986235 and ACT-389949, showed that while both agonists induced strong receptor internalization, only BMS-986235 effectively recycled the receptors back to the plasma membrane. Does COL induce strong receptor internalization? Would prolonged treatment of COL cause receptor desensitization? This may be related to the therapeutic efficacy of COL. Investigating these questions could provide valuable insights into the mechanism of action of COL and its potential as a therapeutic agent.

4. There is a published structure of FPR2 bound to compound 43. How does the binding of COL to the receptor compare to that of compound 43?

5. Humans and mice have very different formyl peptide receptor systems (https://doi.org/10.1111/bcpt.12903). Does COL act on human FPR2? Also, for experiments using heterologous expression systems, e.g. HEK293 over-expressing FPR2, it would be helpful to specify human or mouse FPR2 in figure legend.

Referee #3 (Comments on Novelty/Model System for Author):

The authors claim columbamine could promote inflammation resolution by enhancing macrophage efferocytosis. This is an interesting area of research with immense translational implications. However, the study is limited by its lack of data on specific mechanisms (such as efferocytosis which is the central claim) and lack of PK/PD data for columbamine in in-vivo studies.

Referee #3 (Remarks for Author):

In this manuscript, Wu et al show that columbamine is a biased FPR2 agonist that increases macrophage efferocytosis in a LAP-dependent fashion. These effects are claimed to promote resolution of inflammation in a murine model of colitis. The characterization of the biased signaling on FPR2 is rigorous and well conducted. Although the cell biological and signaling findings are interesting, the data to support the central claim that columbamine-mediated increase in macrophage efferocytosis is critical for the in vivo phenotype is generally lacking.

Major comments:

Screening assay:

1) The authors use the quantification of the numbers of uncleared ACs as an indirect measure of macrophage efferocytosis efficiency. While this is a counter-intuitive but interesting strategy, the authors do not present data for validation of this screening model. For example, the robustness of screening assays is defined by Z-factor (PMID: 10838414). Such data are unavailable to test the validity and usefulness of this novel screening strategy for discovery of efferocytosis modulators.

2) Similarly, how this screening strategy performs with known macrophage efferocytosis modulators such as dexamethasone,

PMA, and Annexin A1 are not shown. These data will be critical to test the validity of the assay.

3) Lastly, screening strategies often use Z-scores to detect "hits" (For eg. PMID: 21515799). These are typically those that fall 2 or even 3 standard deviations above or below the population mean. However, such an approach was not taken here. Instead, the authors used a standard calculation of intensity to identify efferocytosis modulators. While I agree that the authors identified columbamine as an efferocytosis enhancer using this strategy, the robustness and usefulness of this approach for future use has not been well established.

Columbamine as an efferocytosis enhancer

4) Columbamine treated mice show lower numbers of remaining ACs in liver and spleen (Figure 1 H and I). The authors interpret that this is due to enhanced macrophage efferocytosis efficiency. However, an alternate interpretation would be that columbamine treatment alters the trafficking of injected ACs and therefore less of the injected ACs reach liver and spleen. It would be best if the authors directly measure efferocytosis efficiency of macrophages by analysing the percent macrophages that have engulfed an injected AC. Alternatively, the authors can conduct in-situ efferocytosis assays to monitor free and engulfed ACs (such as PMID: 30291029).

5) Similarly, in the DSS-colitis model, the decrease in the numbers of ACs could simply represent decreased intestinal cell death (instead of accelerated clearance). How do the authors exclude that possibility? As above, quantification of macrophage efferocytosis efficiency in the colonic tissue will facilitate direct interpretation of the effect of columbamine on efferocytosis.

6) The authors claim that macrophages and macrophage-mediated efferocytosis are critical for inflammation resolution in the DSS-colitis model. In this context, it is intriguing that macrophage depletion by itself did not affect colon length or disease activity (Figure 4). I would have predicted that that the mere depletion of macrophages would have made the disease worse. Can the authors explain?

7) The authors deplete macrophages and show that the protective effects of columbamine on intestinal inflammation are lost. These data argue a role for macrophages in eliciting the protective effects of columbamine. However, these do not represent evidence to support the claim that columbamine-induced efferocytosis enhancement mediates the beneficial effects. Can the authors block LAP in vivo or other such relevant pathways to show that columbamine indeed protects against intestinal inflammation via enhancement of efferocytosis? Additionally, can the authors directly test the efferocytosis efficiency of macrophages in the colonic sections?

8) It would be important to present TUNEL data for experiments in Figure 4. Can the authors also directly measure macrophage efferocytosis efficiency in the colon? Similar comment for data presented in Figure 6.

9) The authors could discuss the PK/PD of columbamine and whether they could detect columbamine or its metabolic products in their in vivo experiments. These data will be critical for translating these findings into clinical scenario.

Dear Editor:

Thank you very much for your kind consideration on our manuscript. We are also grateful to all the reviewers' insightful comments. We have revised the manuscript according to the comments and hopefully the reviewer will find our paper meet the publication standards. The main corrections have been highlighted in the paper and the point-by-point responses to the reviewers' comments are listed below:

***** Reviewer's comments *****

Referee #1 (Comments on Novelty/Model System for Author):

This is a really nice report describing the discovery of a natural compound that enhances efferocytosis and improves the outcome of DSS-induced colitis. The investigators show that this is du to effects on FPR2. This is an important discovery and links FPR2 function and efferocytosis.

Referee #1 (Remarks for Author):

The authors report studies investigating the effects of a novel natural product, columbamine (COL), on macrophage efferocytosis and show that COL enhances efferocytosis. COL also improves the outcome of DSS-induced colitis through biased agonism of FPR2, which is an interesting finding. This report would benefit from minor revisions.

Specific Comments

1. The authors show in Figure 1 that several compounds enhance macrophage efferocytosis, some to a similar level as columbamine. It would be of interest to explain what those other compounds were and why COL was chosen for further analysis. Are the other active compounds structurally related to columbamine?

Response: We thank reviewer for raising the question.

The top 10 compounds identified through the screening were shown in supplementary table 1. Comparing the structures, we didn't find any shared characteristics among them compare to COL. We select COL because of two reasons: 1, no obvious toxicity was observed at the COL working concentration; 2, COL can be isolated from herbal plant traditionally used to treat colitis and we speculate that COL may be the active compound of this plant to treat colitis. We will characterize other positive compounds in the future.

2. The Figure 1 legend and the text needs to include the dose of COL used to treat the mice. Was the concentration achieved in vivo similar to that used to treat macrophages in vitro? This should be provided or discussed.

Response: Thanks for the valuable suggestions.

We have added the dose of COL used in the Figure 1 legend (words in blue).

The PK/PD studies of columbamine has been published in a Chinese journal, “Chinese Journal of Health Laboratory Technology” *(1)*. In the study, the half-life of columbamine $t_{1/2z}$ was (10 ± 7.9) h or (7.7 ± 4.8) h after intravenous injection and (5.2 ± 2.8) h after oral administration after treating rat with columbamine (10 mg/kg). According to our results, COL at 1 μM is effective to trigger efferocytosis, indicating that the concentration of 0.338 ng/ml in blood is enough to work. Based on the medication time curve, COL can be effective for at least 12 h *in vivo*. The analysis has been added into

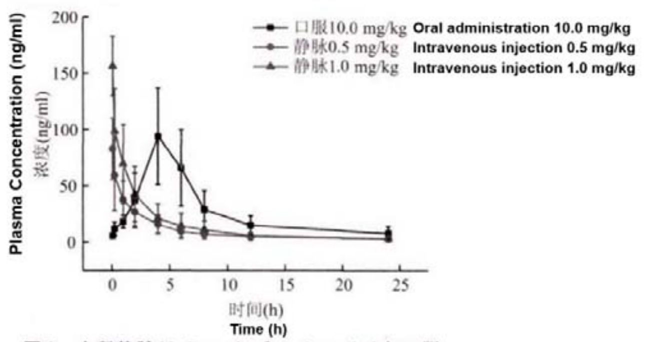

图 2 大鼠静脉(0.5 mg/kg 和 1.0 mg/kg)与口服(10.0 mg/kg)非洲防己碱后的药时曲线图

Columbamine medication time curve after oral administration or intravenous injection of columbamine

discussion part (words in blue).

3. The authors show that COL affects macrophage functions. Was there any cytotoxicity associated with this compound?

Response: Thanks for the question.

To clarify the toxicity of COL, we have tested the cell viability of macrophage after COL treatment for 24 hours using CCK-8 kit. The data

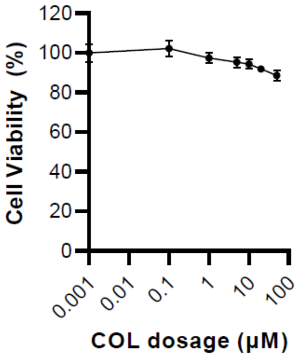

shows that COL did not trigger obvious cell toxicity at working concentration (1 μM). Actually, even at concentration up to 100 μM, macrophages still remain more than 80% viability. The data indicates that COL did not trigger obvious toxicity on macrophages. The data has been added in the Fig. S1A.

4. The authors show that COL affects FPR2 responses and suggest it is an FPR2 biased agonist. Does COL also have effects at FPR1? This should be addressed since many FPR agonists hit both receptors.

Response: We conducted additional experiments to address these questions.

In RBL-FPR1 cells, COL did not induce Ca^{2+} mobilization (A), which is consistent with data from FPR2-expressing cells (C). In cAMP inhibition assay, the EC_{50} value of COL on FPR1 (about 44.3 μM) was 10 times higher than that on FPR2 (3.4 μM). These results suggest that COL activates biased signaling both on FPR2 and FPR1, though with 10 times higher selectivity on FPR2.

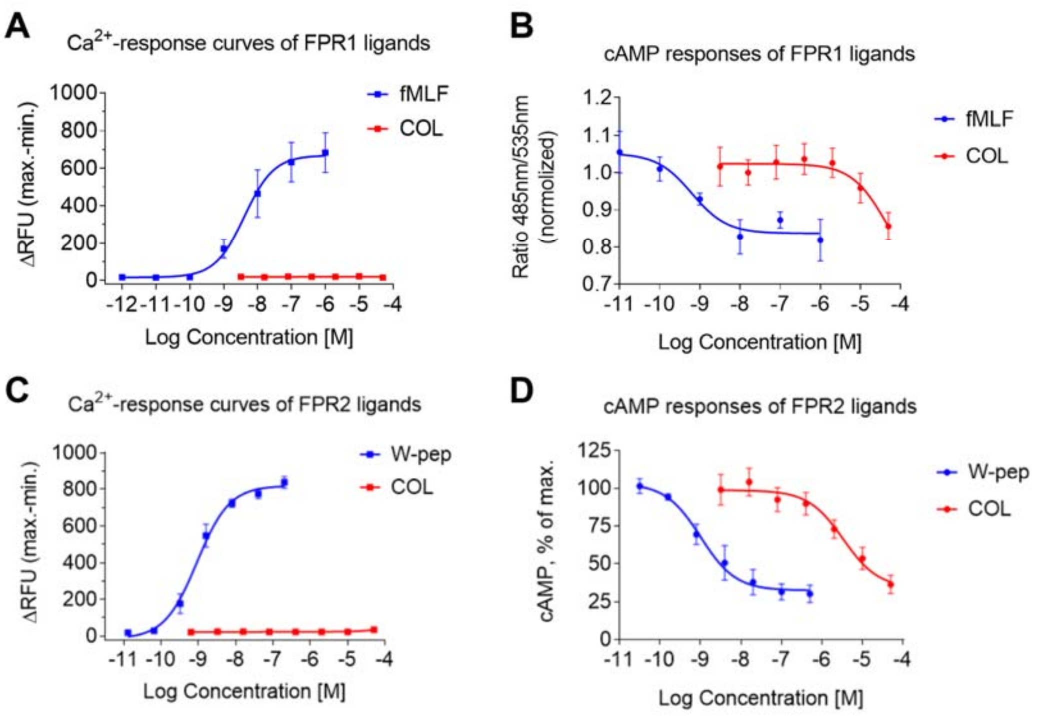

Figure legends:

Comparison of COL-induced FRP1 signaling with FPR2 signaling. (A) Cacium-response curves of COL and fMLF. Calcium responses in FPR1-expressing cells were stimulated by the ligands at the indicated concentrations (n=3). The experimental procedure was similar to that for FPR2. (B) cAMP-response curves of COL and fMLF. The inhibition of forskolin-stimulated cAMP accumulation in FPR1-expressing cells was stimulated by the ligands at the indicated concentrations (n=3). The cAMP responses of FPR1 were detected with FRET-based Epac sensors as described in a published paper (https://doi.org/10.1073/pnas.2214324120). Data of (C) and (D) were the same with those of figure 6A and 6B, respectively.

5. This study was performed on murine cells. However, it is well documented that some FPR ligands that impact mouse cells have no effect on human macrophages, and vice versus. This issue should be addressed or at least a discussion of this issue should be provided to enhance medical implications of this work. Was COL tested with any human macrophages or macrophage cell lines?

Response: Thanks for raising the important question.

Function of FPR2 in different species is a concerned point in many reports. We have evaluated FPR2 signaling on cells expressing human FPR2 (Fig. 7 and Figure S3 (B-H)), the results showed that COL activates human FPR2.

Referee #2 (Remarks for Author):

In this manuscript, Wu et al. report on the identification of columbamine (COL)

as a biased FPR2 agonist that enhances efferocytosis and the efficacy of COL in attenuating intestinal inflammation in a murine colitis model. COL was first identified as a potential efferocytosis enhancers from a small library of natural products. The authors subsequently showed that COL enhances LC3-associated phagocytosis of mouse BMDMs, and they used a murine colitis model to demonstrate the anti-inflammatory function of COL. Stemmed from these findings, the authors have identified FPR2 as the molecular target of COL in enhancing efferocytosis and promoting the resolution of inflammation. Furthermore, the authors have conducted docking and mutagenesis studies to investigate the binding mechanism of COL on FPR2 and mechanistic studies to prove the involvement of RAC1 in COL-mediated enhancement of efferocytosis. Overall, this study is of high quality. The identified novel biased FPR2 agonist as well as the mechanism of FPR2-mediated enhancement of efferocytosis may offer new therapeutic opportunities for inflammatory diseases such as colitis. The paper is expected to have a high impact on the research fields of GPCR pharmacology, inflammation, and IBD. However, there are some issues that I believe need to be addressed or at least discussed.

1. The intriguing observation that COL does not induce Ca2+ immobilization raises interesting questions. Previous research (https://doi.org/10.1038/ncomms14232) has shown that another FPR2 agonist, compound 17b, also appears to be biased away from Ca2+. Interestingly, compared to the FPR2 agonist compound 43, which induces strong Ca2+ responses, compound 17b has been shown to exhibit superior anti-inflammatory and cardioprotective effects in cardiomyocytes. To gain a better understanding of the functional role of biased signaling in efferocytosis, it may be worth investigating the W-peptide, which is typically considered a non-biased agonist of FPR2. Testing the W-peptide in efferocytosis could serve as an important control experiment to help establish a link between the biased signaling properties of COL and its functional role in efferocytosis.

Response: Thanks for the valuable suggestions.

We performed the experiment as suggested and found W-peptide at 1 μM can also induce efferocytosis. The data indicates that FPR2 agonists, either biased or unbaised, are capable of inducing efferocytosis in macrophages.

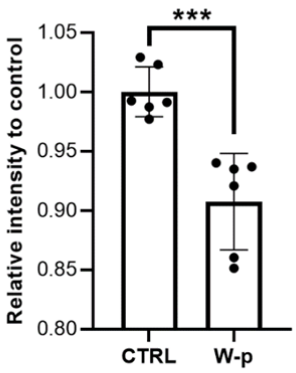

2. Related to 1, is the functional role of COL in promoting efferocytosis exclusive to this compound, or can other synthetic FPR2 agonists, such as compound 43 and BMS compounds, also induce efferocytosis as effectively? Understanding the mechanism of action and efficacy of various FPR2 agonists in promoting efferocytosis could lead to the development of novel therapeutic agents.

Response: Thanks for raising this important concern.

We have tested the efferocytosis-promotion activity of Cp43 at different concentrations. We found Cp43 can also induce efferocytosis in a dose dependent manner (data was shown as below). According to the results, it seems that efferocytosis induction is a general response of FPR2 activation.

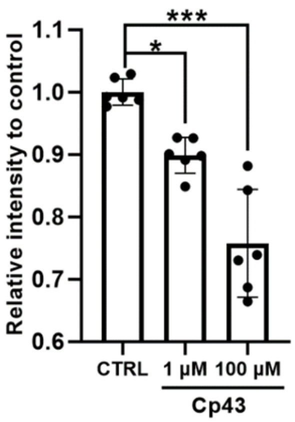

3. Another study (https://doi.org/10.1021/acsptsci.2c00042) comparing two clinical stage FPR2 agonists, BMS-986235 and ACT-389949, showed that while both agonists induced strong receptor internalization, only BMS-986235 effectively recycled the receptors back to the plasma membrane. Does COL induce strong receptor internalization? Would prolonged treatment of COL cause receptor desensitization ? This may be related to the therapeutic efficacy of COL. Investigating these questions could provide valuable insights into the mechanism of action of COL and its potential as a therapeutic agent.

Response: Thanks for raising the interesting question.

COL induced a mild and transient internalization of FPR2 compared with W-pep. The data shown in the original submission was from a 60 min-stimulation experiments (Figure 6E and 6F). We now extended the stimulation time to 120 min, but no further increase in FPR2 internalization was observed. In comparison, W-pep induced strong internalization of FPR2 after 60 min stimulation. As for receptor desensitization, since COL did not induce Ca^{2+} mobilization, the desensitization effect could not be determined. We conclude that prolonged incubation with COL did not cause significant loss of cell surface FPR2 due to receptor internalization. Since COL does not induce

Ca^{2+} mobilization at concentrations up to 50 μM, desensitization did not contribute significantly to its anti-inflammatory effect.

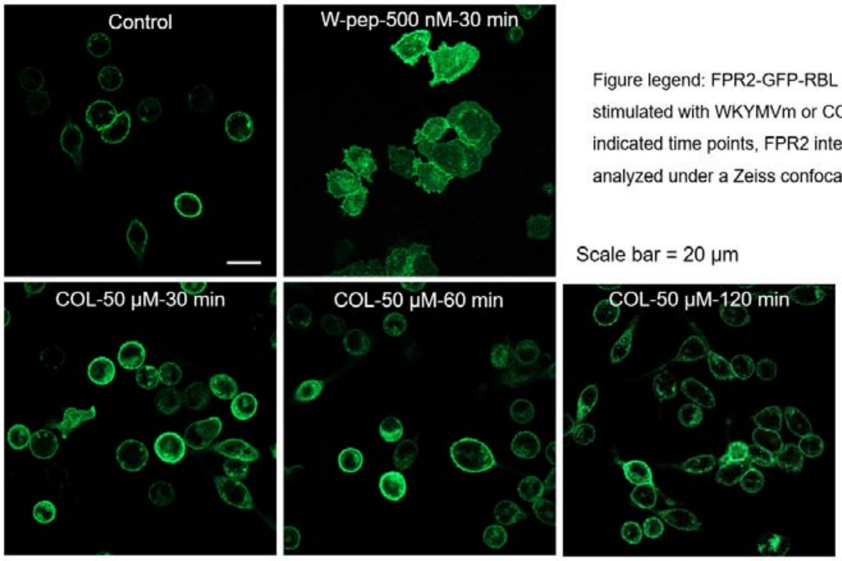

Figure legend: FPR2-GFP-RBL cells were stimulated with WKYMVm or COL. At the indicated time points, FPR2 internalization was analyzed under a Zeiss confocal microscope.

Scale bar = 20 μm

4. There is a published structure of FPR2 bound to compound 43. How does the binding of COL to the receptor compare to that of compound 43?

Response: Thanks for posing the interesting question.

We have compared the binding features of these two compounds to FPR2 (The docking figure and figure legend are shown as below).

Compound 43 (Cpd43) is a dual agonist of FPR1 and FPR2. This pharmacological property is different from COL which is a biased agonist with preference for FPR2. In docking model, Cpd43 inserts into the binding pocket wedge-like with a longitudinal angle (A in Figure below). Cpd43 interacts with V113 but not S288, in addition to key amino acids such as R201, R205 and D106. In comparison, both COL (B) and LXA4 (C) interacts with S288 but not V113. We conclude that COL is closer to LXA4 than Cpd43 in the FPR2 binding pocket.

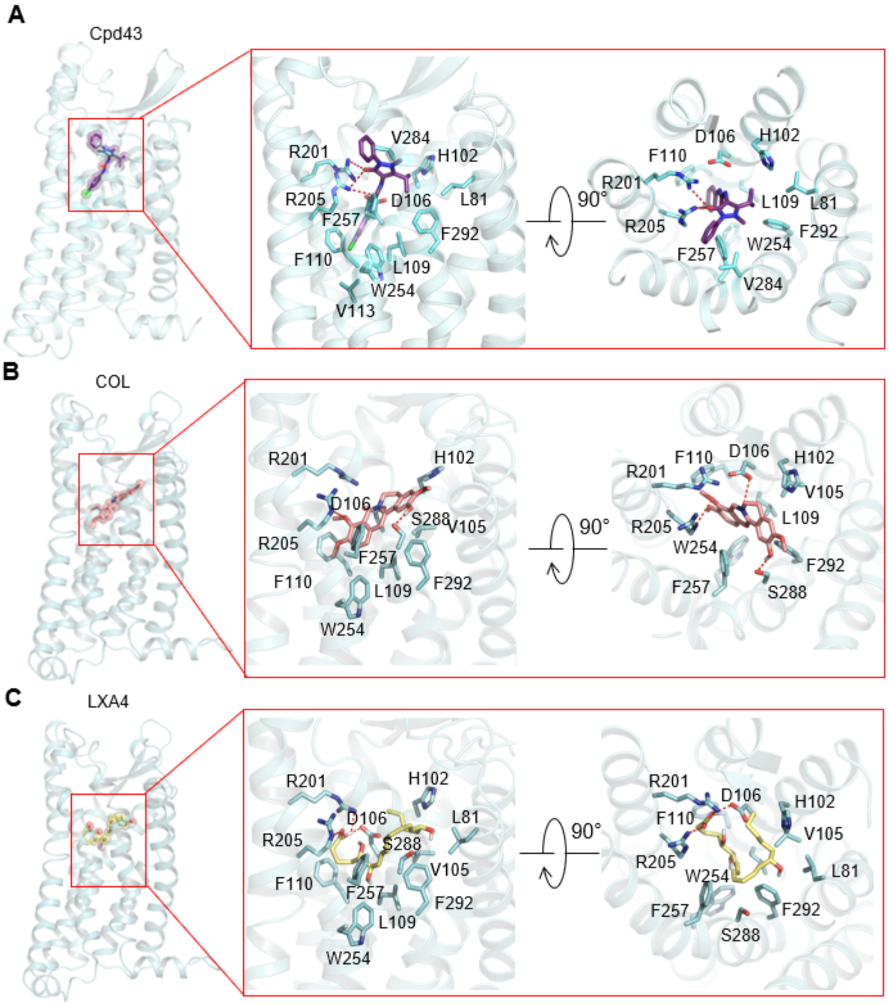

Figure legends:

Comparison of different ligands binding to FPR2 with COL. Molecular docking analysis of columbamine binding to FPR2. The chemical structure of the ligands were obtained from ChemSpider database. Docking analysis was performed by MOE software. A. Overall structure (left, PDB ID: 7T63) and interaction sites (right) of compound 43 (cpd43) binding to FPR2. The cpd43 (sphere and sticks with carbon in purple) occupied a wedge-pose with its chlorophenyl group insertion to FPR2. The polar connections between cpd43 and FPR2 are composed of R201 and R205. The hydrophobic interactions involve L81, H102, L109, F110, V113, W254, F257, V284, F292, and the carbonyl group of D106. B. Ligand binding poses in FPR2 of columbamine (COL) generated from docking analysis. The overall conformation and binding sites viewed from bundle and extracellular side is shown in the left and right panel, respectively. The polar connections between columbamine and FPR2 are composed of D106, R205, and S288. The hydrophobic interactions involve H102, V105, L109, F110, W254, F257, F292, and the guanidino group of R205. C. Docking model of FPR2 bound with lipoxin A4 (LXA4). The detialed

residues of FPR2 involved in binding pocket of lipoxin A4 (right). Hydrophobic connections between LXA4 and FPR2 are formed by L81, H102, V105, L109, F110, W254, F257, F292. Besides the hydrogen network formed by D106-R201-R205, the residue S288 of FPR2 is important in the recognition of LXA4. The polar interactions were indicated in red dash lines.

5. Humans and mice have very different formyl peptide receptor systems (https://doi.org/10.1111/bcpt.12903). Does COL act on human FPR2? Also, for experiments using heterologous expression systems, e.g. HEK293 over-expressing FPR2, it would be helpful to specify human or mouse FPR2 in figure legend.

Response: Thanks for raising the important question.

According to our observation, we found COL acted both on human and mouse FPR2. To determine the downstream of human FPR2 and mouse FPR2 after COL treatment, we have done calcium mobilization test on human or mouse FPR2 overexpressing RBL cells (Figure S3B-E). The results showed that COL did not affect calcium mobilization both on human and mouse FPR2-RBL cells. There is no obvious specie difference on FPR2 signal. In other experiments, such as cAMP response, membrane recruitment and internalization, we have used cells with human FPR2 construct expressing in Figure 7 as recommendation. These data indicates that COL acts on human FPR2. To avoid confusion, we have specified the FPR2 into hFPR2 in the revised MS.

Referee #3 (Comments on Novelty/Model System for Author):

The authors claim columbamine could promote inflammation resolution by enhancing macrophage efferocytosis. This is an interesting area of research with immense translational implications. However, the study is limited by its lack of data on specific mechanisms (such as efferocytosis which is the central claim) and lack of PK/PD data for columbamine in in-vivo studies.

Referee #3 (Remarks for Author):

In this manuscript, Wu et al show that columbamine is a biased FPR2 agonist that increases macrophage efferocytosis in a LAP-dependent fashion. These effects are claimed to promote resolution of inflammation in a murine model of colitis. The characterization of the biased signaling on FPR2 is rigorous and well conducted. Although the cell biological and signaling findings are interesting, the data to support the central claim that columbamine-mediated increase in macrophage efferocytosis is critical for the in vivo phenotype is generally lacking.

Major comments:

Screening assay:

1) The authors use the quantification of the numbers of uncleared ACs as an indirect measure of macrophage efferocytosis efficiency. While this is a counter-intuitive but interesting strategy, the authors do not present data for validation of this screening model. For example, the robustness of screening assays is defined by Z-factor (PMID: 10838414). Such data are unavailable to test the validity and usefulness of this novel screening strategy for discovery of efferocytosis modulators.

Response: Thanks for raising the concern.

As suggestion, we have repeated and re-evaluated our data by adding positive control. Dexamethasone (Dex) (1 μM) and phorbol 12-myristate 13-acetate (PMA) (100 nM) are two positive molecules to stimulate efferocytosis. 6 biological replicates were assigned for each group, and edge wells were excluded. We pretreated the BMDMs in 96-wells with Vehicles, Dex, and PMA for 24 h, followed by adding CMFDA-stained apoptotic cells for another 24 h. We finally obtained and recorded the fluorescent intensity value remained in medium in each well. The results were shown as below. There is a significant difference between vehicle and positive control groups. The data indicates that our method is a reliable measurement of macrophage efferocytosis efficiency.

However, the calculated S/N is about 2-3 and the Z factor is about -1 to -0.5 (shown as below). These data indicate that our screening strategy may not be so robust for a high throughput screening application. We speculate that the high heterogeneity of macrophages and the small difference in fluorescent intensity change make the robustness of assay not so high. Since our focus is the compound itself, we move the high content screening result to the supplementary figure (Fig. S1A-B).

	CTRL	Positive control1	Positive control2
		Dex	PMA
	6.531	5.733	5.792
	6.26	6.077	5.89
	6.333	6.048	5.743
	6.313	6.155	6.092
	6.499	6.035	6.061
	6.34	6.121	5.989
AVE	6.379	6.028166667	5.927833333
SD	0.1	0.138234603	0.130684629
S/N		2.540367305	3.454882215
S/B		1.058254306	1.016925802
Z-factor		-1.032783816	-0.530888136

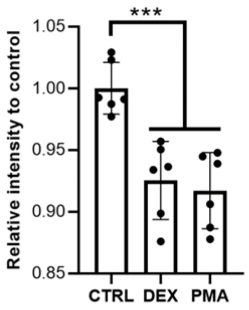

2) Similarly, how this screening strategy performs with known macrophage efferocytosis modulators such as dexamethasone, PMA, and Annexin A1 are not shown. These data will be critical to test the validity of the assay.

Response: According to reviewer's question, we have determined the efferocytosis capacities of known efferocytosis modulators by using our methods. As shown in the data below, dexamethasone, PMA and Ac2-26 (the active peptide of annexin A1) treatment reduced the apoptotic cell fluorescent intensity value significantly. This data supports the reliability of our assay in identifying efferocytosis inducers.

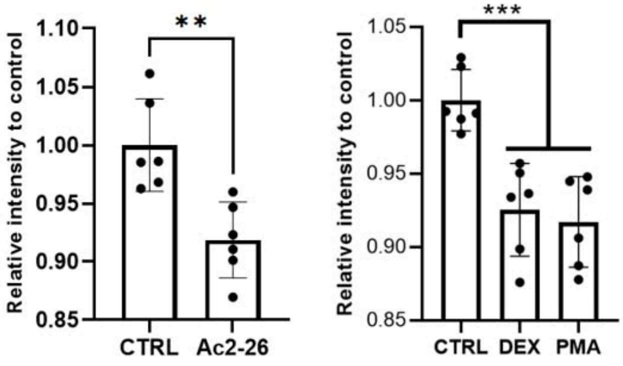

3) Lastly, screening strategies often use Z-scores to detect "hits" (For eg. PMID: 21515799). These are typically those that fall 2 or even 3 standard deviations above or below the population mean. However, such an approach was not taken here. Instead, the authors used a standard calculation of intensity to identify efferocytosis modulators. While I agree that the authors identified columbamine as an efferocytosis enhancer using this strategy, the robustness and usefulness of this approach for future use has not been well established.

Response: Thanks for providing the valuable suggestion. According to author's the suggestion, we have tried to transfer the value into Z-score. We found the graph become more intuitive to indicate the increase and decrease of efferocytosis. We have changed the Data in Fig.1B into a Z-Score analysis in new version.

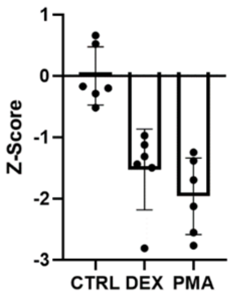

Columbamine as an efferocytosis enhancer

4) Columbamine treated mice show lower numbers of remaining ACs in liver and spleen (Figure 1 H and I). The authors interpret that this is due to enhanced macrophage efferocytosis efficiency. However, an alternate interpretation would be that columbamine treatment alters the trafficking of injected ACs and therefore less of the injected ACs reach liver and spleen. It would be best if the authors directly measure efferocytosis efficiency of macrophages by analysing the percent macrophages that have engulfed an injected AC. Alternatively, the authors can conduct in-situ efferocytosis assays to monitor free and engulfed ACs (such as PMID: 30291029).

Response: Thanks for the valuable suggestion.

We have repeated the ACs clearance experiments in vivo by injecting (i.v.) CMFDA-ACs into mice that were pretreated with COL or vehicle. Considering the efferocytosis is a quick process, we have set two time points (30 min, 12 h). We found ACs-CMFDA intensity increased quickly and accumulated more in the liver and spleen after 30 min in COL-treated mice (Fig. S1E and F). After 12 h, ACs-CMFDA intensity showed lower in COL treated group, indicating a faster clearance rate after COL treatment (Fig. S1E, F).

According to reviewer's suggestion, we have stained macrophages with F4/80 and analyzed the in-situ efferocytosis efficiency. COL treated mice also showed more F4/80-associated ACs in spleen and liver after injection of ACs for 30 min (Fig. S1G, H).

These data have been added in Figure S1E-H.

5) Similarly, in the DSS-colitis model, the decrease in the numbers of ACs could simply represent decreased intestinal cell death (instead of accelerated clearance). How do the authors exclude that possibility? As above, quantification of macrophage efferocytosis efficiency in the colonic tissue will facilitate direct interpretation of the effect of columbamine on efferocytosis.

Response: Thanks for raising the critical question. Clearance of ACs in colon

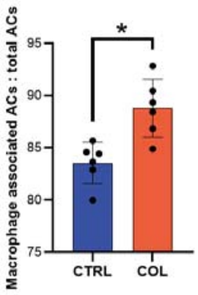

are majorly by peeling off from epithelium and by efferocytosis. According to reviewer's comments, we have determined the in situ efferocytosis efficiency in colon by counting the free TUNEL positive ACs and macrophage-associated ones. As data shown below, COL-treated mice displayed higher efferocytosis efficiency in colon as expected (as below).

6) The authors claim that macrophages and macrophage-mediated efferocytosis are critical for inflammation resolution in the DSS-colitis model. In this context, it is intriguing that macrophage depletion by itself did not affect colon length or disease activity (Figure 4). I would have predicted that the mere depletion of macrophages would have made the disease worse. Can the authors explain?

Response: Thanks for raising the important concern.

Clodronate-liposome induced macrophage depletion has been repeatedly applied in DSS-induced colitis mouse model to figure determine the role of macrophage function in IBD *(2–4)*. However, the disease severity after macrophage depletion is inconsistent in different studies.

Macrophages are regarded as a major source of cytokines that can trigger inflammatory storm and aggravate IBD syndrome *(5)*. Reduction of inflammation by depletion macrophage has been regarded as a strategy for treating IBD *(4)(6)*. On the contrary, macrophages can act as immunosuppressors and aid in tissue repairment after being polarized to M2 states *(7)*. Regulation of macrophage to anti-inflammatory state is an emerging approach for IBD treatment *(8)(9)*. We noticed that similar to our observation, several studies didn't show obvious attenuation effects in colitis model after depletion macrophage *(3)(10)*.

A possible explanation of the inconsistent results after macrophage depletion in DSS-induced colitis model may be the limitation of the macrophages depletion method. The clodronate-liposome macrophages depletion assay is a "knock-down" rather than "knock-out" strategy. In our case, clodronate-liposome depleted roughly 60% peritoneal macrophages, and the remained macrophage may still functional. While in another experiment, we applied a stronger clodronate-liposome administration protocol in which more than 70% macrophages are depleted. In this condition, a more serious damage was observed in the macrophage depletion group (shown as below). These results indicate that the macrophage depletion rates may affect the

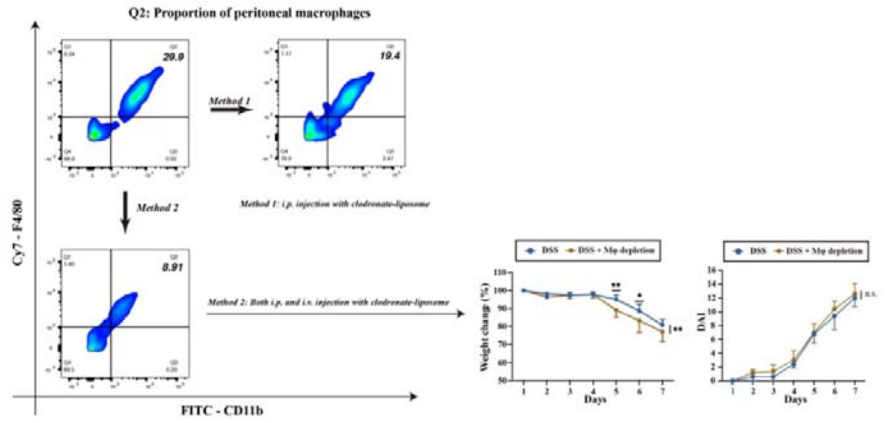

disease activity in DSS-induced colitis model.

7) The authors deplete macrophages and show that the protective effects of columbamine on intestinal inflammation are lost. These data argue a role for macrophages in eliciting the protective effects of columbamine. However, these do not represent evidence to support the claim that columbamine-induced efferocytosis enhancement mediates the beneficial effects. Can the authors block LAP in vivo or other such relevant pathways to show that columbamine indeed protects against intestinal inflammation via enhancement of efferocytosis? Additionally, can the authors directly test the efferocytosis efficiency of macrophages in the colonic sections?

Response: Thanks for the valuable suggestion. As suggested, we used 3-methyladenine (3-MA) to block LAP *in vivo (11)*, and treated mice with DSS to induce colitis mouse model. We found the protective effects of COL in colitis mouse model can be blocked by treating 3-MA (supplementary figure 3A-F). These data demonstrated that COL potentially induced a LAP-dependent protective effects on intestinal inflammation. We have also tested the in-situ efferocytosis efficiency in colon, the results showed a higher macrophage-associated ACs ratio (Figure S3G-H).

8) It would be important to present TUNEL data for experiments in Figure 4. Can the authors also directly measure macrophage efferocytosis efficiency in the colon? Similar comment for data presented in Figure 6.

Response: According to reviewer's suggestion, we have counted and analyzed the efferocytosis efficiency in colon as shown in Fig. 4 and Fig. 6. We found depletion of macrophage partially inhibited the COL-induced enhancement of efferocytosis efficiency in colon (A). COL also failed to trigger increase of efferocytosis in the mice colon without FPR2 (B). These results were consistent with our conclusion and the results have been added in the Fig. S4A and Fig. S5B in the revised MS.

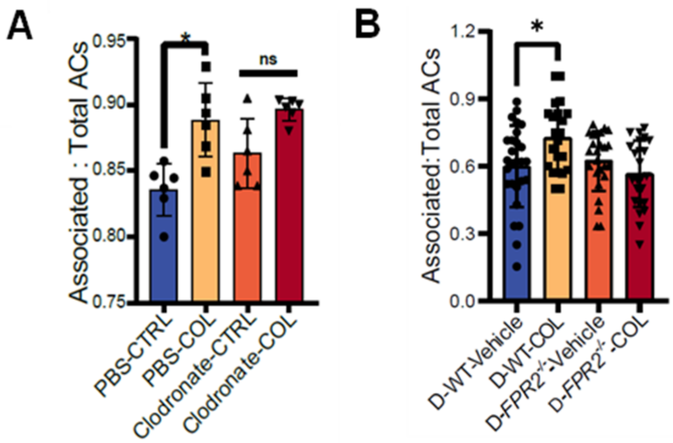

9) The authors could discuss the PK/PD of columbamine and whether they could detect columbamine or its metabolic products in their in vivo experiments. These data will be critical for translating these findings into clinical scenario.

Response: Thanks for reviewer's suggestion.

PK/PD studies of columbamine has been published in a Chinese journal, Chinese Journal of Health Laboratory Technology *(1)*. In this study, rats were oral administrated (10 mg/kg) or intravenous injected (0.5 mg/kg and 1.0 mg/kg) with columbamine, and bloods were collected after 0.08 h, 0.25 h, 1 h, 2 h, 4 h, 6 h, 8 h, 12 h, 24 h for pharmacokinetics study (the result were shown as the figure below). The absolute bioavailability of columbamine was about 15.1%. The half-life period of columbamine $t_{1/2z}$ was (10 ± 7.9) h or (7.7 ± 4.8) h after intravenous injection and (5.2 ± 2.8) h after oral administration.

Although the bioavailability isn't high, COL can still work at a low concentration level for a long period in blood. According to our results, COL at 1 μM is effective to trigger efferocytosis, indicating that the concentration of 0.338 ng/ml in blood is enough to work. Based on the medication time curve, COL can be effective for at least 12 h. Taken together, COL shows potential application value on clinical scenario.

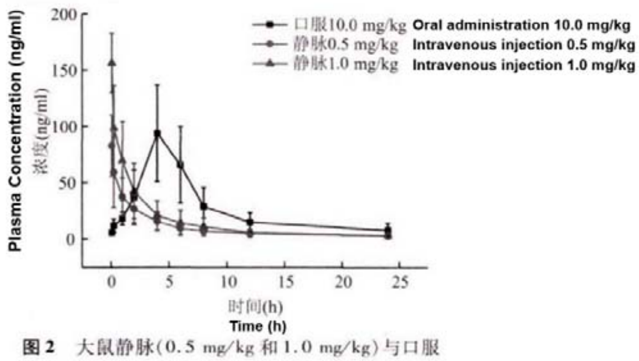

图2 大鼠静脉(0.5 mg/kg 和 1.0 mg/kg)与口服(10.0 mg/kg)非洲防己碱后的药时曲线图

Columbamine medication time curve after oral administration or intravenous injection of columbamine

29th Sep 2023

Dear Dr. Lu,

Thank you for the submission of your revised manuscript to EMBO Molecular Medicine. I am pleased to inform you that we will be able to accept your manuscript pending the following final amendments:

1) Source data:
- Please complete the Source Data Checklist.

2) In the main manuscript file, please do the following:
- Please address all comments suggested by our data editors listed below:
o Please note that the figure legend style does not comply with the journal guidelines i.e. all the figure legends are in a run-on style.
o Please note that a separate 'Data Information' section is required in the legends of all the figures.
o Please note that information related to n is missing in the legend of figures 5c-g; 7f; supplementary figures 3h; 5g-h.
o Please note that the error bars are not defined in the legend of figures 8e-f.
o Please note that the box plots need to be defined in terms of minima, maxima, centre, bounds of box and whiskers, and percentile in the legend of figures 9g.
o Please note that the scale bar needs to be defined for figures 7e, g.
- Limit keywords to max. 5.
- Add callouts for Figure 1B.
- Move main figure legends to the end of the manuscript and remove the "List of Supplementary Materials" and its content.
- Place following sections after M&M in the order: Data Availability - Acknowledgments - Disclosure Statement & Competing Interests - References - Figure Legends
- In M&M, provide the antibody dilutions that were used for each antibody.
- In M&M, statistical paragraph should reflect all information that you have filled in the Authors Checklist, especially regarding randomization, blinding, replication.
- Remove "List of Appendix material".
- Please merge "Funding" with "Acknowledgments".
- Please rename "Competing interests" to "Disclosure Statement & Competing Interests". We updated our journal's competing interests policy in January 2022 and request authors to consider both actual and perceived competing interests. Please review the policy https://www.embopress.org/competing-interests and update your competing interests if necessary.
- Author contributions: Please remove it from the manuscript and specify author contributions in our submission system. CRediT has replaced the traditional author contributions section because it offers a systematic machine-readable author contributions format that allows for more effective research assessment. You are encouraged to use the free text boxes beneath each contributing author's name to add specific details on the author's contribution. More information is available in our guide to authors:
https://www.embopress.org/page/journal/17574684/authorguide#authorshipguidelines
- Rename "Data and materials availability" to "Data availability". Please be aware that large scale data (RNA seq) need to be deposited in a public repository and made freely available. Currently, it is not clear whether you performed an RNA seq experiment as indicated in M&M section or analyzed published transcriptome data. Please clarify this and in case you only reanalyzed published data - clearly indicate it in the text, remove information about RNA seq from M&M section, and refer to the original manuscript and add data citation. Also, in that case please replace the current text with following sentence: This study includes no data deposited in external repositories.

In case you performed RNA seq experiment please deposit the raw data in an appropriate public repository and use the following format to report the accession number of your data:

The datasets produced in this study are available in the following databases:
[data type]: [full name of the resource] [accession number/identifier] ([doi or URL or identifiers.org/DATABASE:ACCESSION])

Please check "Author Guidelines" for more information.
https://www.embopress.org/page/journal/17574684/authorguide#availabilityofpublishedmaterial
3) Appendix: Please move supplementary materials and methods to M&M section in the main manuscript file. Add table of content and page numbers and correct nomenclature to "Appendix Figure S1" etc and "Appendix Table S1" etc, also in the main manuscript text.
4) Movies: Renamed the files to Movie EV1, etc. Remove their legends form the main manuscript file and zipp each legend as a readme.txt file with its corresponding movie.

5) The Paper Explained: Please provide "The Paper Explained" and add it to the main manuscript text. Please check "Author Guidelines" for more information. https://www.embopress.org/page/journal/17574684/authorguide#researcharticleguide
6) Synopsis:
- Synopsis image: Please upload the image as a high-resolution jpeg file 550 px-wide x (250-400)-px high to illustrate your article.
- Please check your synopsis text and image before submission with your revised manuscript. Please be aware that in the proof stage minor corrections only are allowed (e.g., typos).
7) For more information: This space should be used to list relevant web links for further consultation by our readers. Could you identify some relevant ones and provide such information as well? Some examples are patient associations, relevant databases, OMIM/proteins/genes links, author's websites, etc...
8) As part of the EMBO Publications transparent editorial process initiative (see our Editorial at http://embomolmed.embopress.org/content/2/9/329), EMBO Molecular Medicine will publish online a Review Process File (RPF) to accompany accepted manuscripts. This file will be published in conjunction with your paper and will include the anonymous referee reports, your point-by-point response and all pertinent correspondence relating to the manuscript. Let us know whether you agree with the publication of the RPF and as here, if you want to remove or not any figures from it prior to publication. Please note that the Authors checklist will be published at the end of the RPF.
9) Please provide a point-by-point letter INCLUDING my comments as well as the reviewer's reports and your detailed responses (as Word file).

I look forward to reading a new revised version of your manuscript as soon as possible.

Yours sincerely,

Zeljko Durdevic

Zeljko Durdevic
Editor
EMBO Molecular Medicine

*** Instructions to submit your revised manuscript ***

*** PLEASE NOTE *** As part of the EMBO Publications transparent editorial process initiative (see our Editorial at https://www.embopress.org/doi/pdf/10.1002/emmm.201000094), EMBO Molecular Medicine will publish online a Review Process File to accompany accepted manuscripts.

In the event of acceptance, this file will be published in conjunction with your paper and will include the anonymous referee reports, your point-by-point response and all pertinent correspondence relating to the manuscript. If you do NOT want this file to be published, please inform the editorial office at contact@embomolmed.org.

When submitting your revised manuscript, please include:

1) a .docx formatted version of the manuscript text (including Figure legends and tables)

2) Separate figure files*

3) supplemental information as Expanded View and/or Appendix. Please carefully check the authors guidelines for formatting Expanded view and Appendix figures and tables at https://www.embopress.org/page/journal/17574684/authorguide#expandedview

4) a letter INCLUDING the reviewer's reports and your detailed responses to their comments (as Word file).

5) The paper explained: EMBO Molecular Medicine articles are accompanied by a summary of the articles to emphasize the major findings in the paper and their medical implications for the non-specialist reader. Please provide a draft summary of your

article highlighting
- the medical issue you are addressing,
- the results obtained and
- their clinical impact.
This may be edited to ensure that readers understand the significance and context of the research.
Please refer to any of our published articles for an example.

6) For more information: There is space at the end of each article to list relevant web links for further consultation by our readers. Could you identify some relevant ones and provide such information as well? Some examples are patient associations, relevant databases, OMIM/proteins/genes links, author's websites, etc...

7) Author contributions: the contribution of every author must be detailed in a separate section.

8) EMBO Molecular Medicine now requires a complete author checklist (https://www.embopress.org/page/journal/17574684/authorguide) to be submitted with all revised manuscripts. Please use the checklist as guideline for the sort of information we need WITHIN the manuscript. The checklist should only be filled with page numbers were the information can be found. This is particularly important for animal reporting, antibody dilutions (missing) and exact values and n that should be indicted instead of a range.

9) Every published paper now includes a 'Synopsis' to further enhance discoverability. Synopses are displayed on the journal webpage and are freely accessible to all readers. They include a short stand first (maximum of 300 characters, including space) as well as 2-5 one sentence bullet points that summarise the paper. Please write the bullet points to summarise the key NEW findings. They should be designed to be complementary to the abstract - i.e. not repeat the same text. We encourage inclusion of key acronyms and quantitative information (maximum of 30 words / bullet point). Please use the passive voice. Please attach these in a separate file or send them by email, we will incorporate them accordingly.

You are also welcome to suggest a striking image or visual abstract to illustrate your article. If you do please provide a jpeg file 550 px-wide x 400-px high.

10) A Conflict of Interest statement should be provided in the main text

11) Please note that we now mandate that all corresponding authors list an ORCID digital identifier. This takes <90 seconds to complete. We encourage all authors to supply an ORCID identifier, which will be linked to their name for unambiguous name identification.

Currently, our records indicate that the ORCID for your account is 0000-0002-1147-125X.

Please click the link below to modify this ORCID:
Link Not Available

12) The system will prompt you to fill in your funding and payment information. This will allow Wiley to send you a quote for the article processing charge (APC) in case of acceptance. This quote takes into account any reduction or fee waivers that you may be eligible for. Authors do not need to pay any fees before their manuscript is accepted and transferred to our publisher.

*Additional important information regarding Figures

Each figure should be given in a separate file and should have the following resolution:
Graphs 800-1,200 DPI
Photos 400-800 DPI
Colour (only CMYK) 300-400 DPI"

Figures are not edited by the production team. All lettering should be the same size and style; figure panels should be indicated by capital letters (A, B, C etc). Gridlines are not allowed except for log plots. Figures should be numbered in the order of their appearance in the text with Arabic numerals. Each Figure must have a separate legend and a caption is needed for each panel.

*Additional important information regarding figures and illustrations can be found at https://bit.ly/EMBOPressFigurePreparationGuideline. See also figure legend preparation guidelines: https://www.embopress.org/page/journal/17574684/authorguide#figureformat

The system will prompt you to fill in your funding and payment information. This will allow Wiley to send you a quote for the article processing charge (APC) in case of acceptance. This quote takes into account any reduction or fee waivers that you may be eligible for. Authors do not need to pay any fees before their manuscript is accepted and transferred to our publisher.

***** Reviewer's comments *****

Referee #1 (Remarks for Author):

This is a really nice report describing the discovery of a natural compound that enhances efferocytosis and improves the outcome of DSS-induced colitis. The authors have addressed my concerns.

Referee #2 (Comments on Novelty/Model System for Author):

There are plenty of small molecule FPR2 agonists reported. The authors identified a natural product as a new FPR2 agonist with a unique chemical scaffold. The effects of this compound in efferocytosis is very interesting.

Referee #2 (Remarks for Author):

The authors have addressed all of my concerns on the biased signaling property, the selectivity and the binding pose of their new FPR2 agonist. I don't have further concerns.

Referee #3 (Comments on Novelty/Model System for Author):

The authors have used a relevant animal model of colitis and conducted in vivo analysis of efferocytosis

Referee #3 (Remarks for Author):

The authors have adequately addressed the major concerns.

The authors addressed the remaining editorial issues.

24th Oct 2023

Dear Dr. Lu,

We are pleased to inform you that your manuscript is accepted for publication and is now being sent to our publisher to be included in the next available issue of EMBO Molecular Medicine.

Please read below for additional IMPORTANT information regarding your article, its publication and the production process.

Congratulations on your interesting work,

Zeljko Durdevic

Zeljko Durdevic
Editor
EMBO Molecular Medicine

Follow us on Twitter @EmboMolMed
Sign up for eTOCs at embopress.org/alertsfeeds

*** *** *** IMPORTANT INFORMATION *** *** ***

SPEED OF PUBLICATION
The journal aims for rapid publication of papers, using using the advance online publication "Early View" to expedite the process: A properly copy-edited and formatted version will be published as "Early View" after the proofs have been corrected. Please help the Editors and publisher avoid delays by providing e-mail address(es), telephone and fax numbers at which author(s) can be contacted.

Should you be planning a Press Release on your article, please get in contact with embomolmed@wiley.com as early as possible, in order to coordinate publication and release dates.

LICENSE AND PAYMENT:

All articles published in EMBO Molecular Medicine are fully open access: immediately and freely available to read, download and share.

EMBO Molecular Medicine charges an article processing charge (APC) to cover the publication costs. You, as the corresponding author for this manuscript, should have already received a quote with the article processing fee separately. Please let us know in case this quote has not been received.

Once your article is at Wiley for editorial production you will receive an email from Wiley's Author Services system, which will ask you to log in and will present you with the publication license form for completion. Within the same system the publication fee can be paid by credit card, an invoice, pro forma invoice or purchase order can be requested.

Payment of the publication charge and the signed Open Access Agreement form must be received before the article can be published online.

PROOFS

You will receive the proofs by e-mail approximately 2 weeks after all relevant files have been sent o our Production Office. Please return them within 48 hours and if there should be any problems, please contact the production office at embopressproduction@wiley.com.

Please inform us if there is likely to be any difficulty in reaching you at the above address at that time. Failure to meet our

deadlines may result in a delay of publication.

All further communications concerning your paper proofs should quote reference number EMM-2023-17815-V3 and be directed to the production office at embopressproduction@wiley.com.

Thank you,

Zeljko Durdevic
Editor
EMBO Molecular Medicine

EMBO Press Author Checklist

Corresponding Author Name: Jia-Hong Lu, Richard D. Ye, Jin Chai	
Journal Submitted to: EMBO Molecular Medicine	
Manuscript Number: EMM-2023-17815	

USEFUL LINKS FOR COMPLETING THIS FORM
The EMBO Journal - Author Guidelines
EMBO Reports - Author Guidelines
Molecular Systems Biology - Author Guidelines
EMBO Molecular Medicine - Author Guidelines

Reporting Checklist for Life Science Articles (updated January
This checklist is adapted from Materials Design Analysis Reporting (MDAR) Checklist for Authors. MDAR establishes a minimum set of requirements in transparent reporting in the life sciences (see Statement of Task: 10.31222/osf.io/9sm4x). Please follow the journal's guidelines in preparing your
Please note that a copy of this checklist will be published alongside your article.

Abridged guidelines for figures
1. Data
The data shown in figures should satisfy the following conditions:
- the data were obtained and processed according to the field's best practice and are presented to reflect the results of the experiments in an accurate and unbiased manner.
- ideally, figure panels should include only measurements that are directly comparable to each other and obtained with the same assay.
- plots include clearly labeled error bars for independent experiments and sample sizes. Unless justified, error bars should not be shown for technical
- if n<5, the individual data points from each experiment should be plotted. Any statistical test employed should be justified.
- Source Data should be included to report the data underlying figures according to the guidelines set out in the authorship guidelines on Data

2. Captions
Each figure caption should contain the following information, for each panel where they are relevant:
- a specification of the experimental system investigated (eg cell line, species name).
- the assay(s) and method(s) used to carry out the reported observations and measurements.
- an explicit mention of the biological and chemical entity(ies) that are being measured.
- an explicit mention of the biological and chemical entity(ies) that are altered/varied/perturbed in a controlled manner.
- the exact sample size (n) for each experimental group/condition, given as a number, not a range;
- a description of the sample collection allowing the reader to understand whether the samples represent technical or biological replicates (including how many animals, litters, cultures, etc.).
- a statement of how many times the experiment shown was independently replicated in the laboratory.
- definitions of statistical methods and measures:
  - common tests, such as t-test (please specify whether paired vs. unpaired), simple χ2 tests, Wilcoxon and Mann-Whitney tests, can be unambiguously identified by name only, but more complex techniques should be described in the methods section;
  - are tests one-sided or two-sided?
  - are there adjustments for multiple comparisons?
  - exact statistical test results, e.g., P values = x but not P values < x;
  - definition of 'center values' as median or average;
  - definition of error bars as s.d. or s.e.m.

Please complete ALL of the questions below.
Select "Not Applicable" only when the requested information is not relevant for your study.

Materials

Newly Created Materials	Information included in the manuscript?	In which section is the information available? (Reagents and Tools Table, Materials and Methods, Figures, Data Availability Section)
New materials and reagents need to be available; do any restrictions apply?	Not Applicable	

Antibodies	Information included in the manuscript?	In which section is the information available? (Reagents and Tools Table, Materials and Methods, Figures, Data Availability Section)
For **antibodies** provide the following information:<br>- Commercial antibodies: RRID (if possible) or supplier name, catalogue number and or/clone number<br>- Non-commercial: RRID or citation	Yes	"Reagent, antibodies and plasmids" section in "materials and methods"

DNA and RNA sequences	Information included in the manuscript?	In which section is the information available? (Reagents and Tools Table, Materials and Methods, Figures, Data Availability Section)
**Short novel DNA or RNA including primers, probes**: provide the sequences.	Yes	The primers used in genotyping was shown in "Animals" section in "materials and methods"

Cell materials	Information included in the manuscript?	In which section is the information available? (Reagents and Tools Table, Materials and Methods, Figures, Data Availability Section)
**Cell lines:** Provide species information, strain. Provide accession number in repository **OR** supplier name, catalog number, clone number, and/**OR** RRID.	Yes	"Cell Culture" section in "materials and methods"
**Primary cultures:** Provide species, strain, sex of origin, genetic modification status.	Yes	"BMDM" section in "materials and methods"
Report if the cell lines were recently **authenticated** (e.g., by STR profiling) and tested for mycoplasma contamination.	Not Applicable	

Experimental animals	Information included in the manuscript?	In which section is the information available? (Reagents and Tools Table, Materials and Methods, Figures, Data Availability Section)
**Laboratory animals or Model organisms:** Provide species, strain, sex, age, genetic modification status. Provide accession number in repository **OR** supplier name, catalog number, clone number, **OR** RRID.	Yes	"Animals" section in "materials and methods"
**Animal observed in or captured from the field:** Provide species, sex, and age where possible**.**	Not Applicable	
Please detail **housing and husbandry conditions**.	Yes	"Animals" section in "materials and methods"

Plants and microbes	Information included in the manuscript?	In which section is the information available? (Reagents and Tools Table, Materials and Methods, Figures, Data Availability Section)
**Plants:** provide species and strain, ecotype and cultivar where relevant, unique accession number if available, and source (including location for collected wild specimens).	Not Applicable	
**Microbes:** provide species and strain, unique accession number if available, and source.	Not Applicable	

Human research participants	Information included in the manuscript?	In which section is the information available? (Reagents and Tools Table, Materials and Methods, Figures, Data Availability Section)
If collected and within the bounds of privacy constraints report on age, sex and gender or ethnicity for all study participants.	Not Applicable	

Core facilities	Information included in the manuscript?	In which section is the information available? (Reagents and Tools Table, Materials and Methods, Figures, Data Availability Section)
If your work benefited from core facilities, was their service mentioned in the acknowledgments section?	Yes	In the "Acknowledgments" section

Design

Study protocol	Information included in the manuscript?	In which section is the information available? (Reagents and Tools Table, Materials and Methods, Figures, Data Availability Section)
If study protocol has been **pre-registered, provide DOI in the manuscript**. For clinical trials, provide the trial registration number **OR** cite DOI.	Not Applicable	
Report the **clinical trial registration number** (at ClinicalTrials.gov or equivalent), where applicable.	Not Applicable	

Laboratory protocol	Information included in the manuscript?	In which section is the information available? (Reagents and Tools Table, Materials and Methods, Figures, Data Availability Section)
Provide DOI OR other citation details if **external detailed step-by-step protocols** are available.	Yes	some methods description in "materials and methods" section

Experimental study design and statistics	Information included in the manuscript?	In which section is the information available? (Reagents and Tools Table, Materials and Methods, Figures, Data Availability Section)
Include a statement about **sample size** estimate even if no statistical methods were used.	Yes	In the figure or figure legend
Were any steps taken to minimize the effects of subjective bias when allocating animals/samples to treatment (e.g. **randomization procedure**)? If yes, have they been described?	Yes	In the section of "statitical analysis"
Include a statement about **blinding** even if no blinding was done.	Yes	In the section of "statitical analysis"
Describe **inclusion/exclusion criteria** if samples or animals were excluded from the analysis. Were the criteria pre-established?<br><br>If sample or data points were omitted from analysis, report if this was due to attrition or intentional exclusion and provide justification.	Yes	In the section of "statitical analysis"
For every figure, are **statistical tests** justified as appropriate? Do the data meet the assumptions of the tests (e.g., normal distribution)? Describe any methods used to assess it. Is there an estimate of variation within each group of data? Is the variance similar between the groups that are being statistically compared?	Yes	In the figure legends

Sample definition and in-laboratory replication	Information included in the manuscript?	In which section is the information available? (Reagents and Tools Table, Materials and Methods, Figures, Data Availability Section)
In the figure legends: state number of times the experiment was **replicated** in laboratory.	Yes	In the figure legends
In the figure legends: define whether data describe **technical or biological replicates**.	Yes	In the figure legends

Ethics

Ethics	Information included in the manuscript?	In which section is the information available? (Reagents and Tools Table, Materials and Methods, Figures, Data Availability Section)
Studies involving **human participants**: State details of **authority granting ethics approval** (IRB or equivalent committee(s), provide reference number for approval.	Not Applicable	
Studies involving **human participants**: Include a statement confirming that **informed consent** was obtained from all subjects and that the experiments conformed to the principles set out in the WMA Declaration of Helsinki and the Department of Health and Human Services Belmont Report.	Not Applicable	
Studies involving **human participants: For** publication of **patient photos**, include a statement confirming that consent to publish was obtained.	Not Applicable	
Studies involving experimental **animals**: State details of **authority granting ethics approval** (IRB or equivalent committee(s), provide reference number for approval. Include a statement of compliance with ethical regulations.	Yes	In the "Animals" section in "materials and methods"
Studies involving **specimen and field samples:** State if relevant **permits** obtained, provide details of authority approving study; if none were required, explain why.	Not Applicable	

Dual Use Research of Concern (DURC)	Information included in the manuscript?	In which section is the information available? (Reagents and Tools Table, Materials and Methods, Figures, Data Availability Section)
Could your study fall under dual use research restrictions? Please check biosecurity documents and list of **select agents and toxins** (CDC): https://www.selectagents.gov/sat/list.htm	Not Applicable	
If you used a select agent, is the security level of the lab appropriate and reported in the manuscript?	Not Applicable	
If a study is subject to dual use research of concern regulations, is the name of the **authority granting approval and reference number** for the regulatory approval provided in the manuscript?	Not Applicable	

Reporting

The MDAR framework recommends adoption of discipline-specific guidelines, established and endorsed through community initiatives. Journals have their own policy about requiring specific guidelines and recommendations to complement MDAR.

Adherence to community standards	Information included in the manuscript?	In which section is the information available? (Reagents and Tools Table, Materials and Methods, Figures, Data Availability Section)
State if relevant guidelines or checklists (e.g., **ICMJE, MIBBI, ARRIVE, PRISMA**) have been followed or provided.	Not Applicable	
For **tumor marker prognostic studies,** we recommend that you follow the **REMARK** reporting guidelines (see link list at top right). See author guidelines, under 'Reporting Guidelines'. Please confirm you have followed these guidelines.	Not Applicable	
For **phase II and III randomized controlled trials**, please refer to the **CONSORT** flow diagram (see link list at top right) and submit the CONSORT checklist (see link list at top right) with your submission. See author guidelines, under 'Reporting Guidelines'. Please confirm you have submitted this list.	Not Applicable	

Data Availability

Data availability	Information included in the manuscript?	In which section is the information available? (Reagents and Tools Table, Materials and Methods, Figures, Data Availability Section)
Have **primary datasets** been deposited according to the journal's guidelines (see 'Data Deposition' section) and the respective accession numbers provided in the Data Availability Section?	Not Applicable	
Were **human clinical and genomic datasets** deposited in a public access-controlled repository in accordance to ethical obligations to the patients and to the applicable consent agreement?	Not Applicable	
Are **computational models** that are central and integral to a study available without restrictions in a machine-readable form? Were the relevant accession numbers or links provided?	Not Applicable	
If publicly available data were reused, provide the respective **data citations in the reference list**.	Yes	The publicly availiable clinical RNAseq data has been reused in the result section.
